# Genome-wide identification, characterization, and functional analysis of the *CHX*, *SOS*, and *RLK* genes in *Solanum lycopersicum* under salt stress

**DOI:** 10.1038/s41598-024-83221-w

**Published:** 2025-01-07

**Authors:** Amaal Maghraby, Mohamed Alzalaty

**Affiliations:** 1 Botany and Microbiology Department, Faculty of Science, Cairo University, Cairo, Egypt; 2 Department of Plant Genetic Transformation, Agricultural Genetic Engineering Research Institute (AGERI), Agricultural Research Center (ARC), Cairo, Egypt

**Keywords:** *CHX*, *SOS*, *RLK*, Genome-wide identification, Evolutionary analysis, Salt stress, Biotechnology, Computational biology and bioinformatics, Evolution, Genetics, Molecular biology, Plant sciences

## Abstract

The *cation/proton exchanger* (*CHX*), *salt overly sensitive* (*SOS*), *and receptor-like kinase* (*RLK*) genes play significant roles in the response to salt stress in plants. This study is the first to identify the *SOS* gene in *Solanum lycopersicum* (tomato) through genome-wide analysis under salt stress conditions. Quantitative reverse transcription PCR (qRT-PCR) results indicated that the expression levels of *CHX*, *SOS*, and *RLK* genes were upregulated, with fold changes of 1.83, 1.49, and 1.55, respectively, after 12 h of exposure to salt stress. Genome-wide analysis revealed 21 *CHX*, 5 *SOS*, and 86 *RLK* genes in *S. lycopersicum*. *CHX* genes were found on chromosomes 2, 3, 4, 5, 6, 7, 8, 9, 11, and 12 of *S. lycopersicum*. *SOS* genes were found on chromosomes 1, 4, 6, and 10. *RLK* genes were found on all chromosomes of *S. lycopersicum*. The Ka/Ks ratios indicate that the *CHX*, *SOS*, and *RLK* genes have been primarily influenced by purifying selection. This suggests that these genes have faced strong environmental pressures throughout their evolution. Purifying selection typically results in a decrease in genetic diversity. The estimated duplication time for *CHX* paralogous gene pairs ranged from approximately 26.965 to 245.413 million years ago (Mya), while the duplication time for *SOS* paralogous gene pairs ranged from around 116.682 to 275.631 Mya. For *RLK* paralogous gene pairs, the duplication time varied from approximately 27.689 to 239.376 Mya. Synteny analysis of the *CHX*, *SOS*, and *RLK* genes demonstrated collinear relationships with orthologous genes in *Arabidopsis thaliana*, but no collinearity orthologous relationships in *Oryza sativa* (rice). Furthermore, the analysis revealed that there were 6 orthologous *SlCHX* genes, 2 orthologous *SlSOS* genes, and 44 orthologous *SlRLK* genes paired with those in A. thaliana. The results of the present study may help to elucidate the role of the *CHX*,* SOS*, and *RLK* genes in salt stress in *S. lycopersicum*.

**Supplementary Information:**

The online version contains supplementary material available at 10.1038/s41598-024-83221-w.

## Introduction

Although seawater and ocean water are salty, any small variations in these nutrients can have dramatic effects on the water cycle and ocean circulation, which can affect the climate in which we live^1^. The results of tomato (*Solanum lycopersicum*) seed germination under different concentrations of NaCl_2_ (25, 50, 100 and 200 mM) revealed that the root length decreased under all concentrations, and a prominent delay in the seed germination rate, germination percentage and shoot length significantly influenced all the treatments with NaCl_2_^2^. Salinity is considered the main challenge to plants under osmotic stress and ionic stress. First, salinity reduces the ability of plants to absorb water, whereas second, salinity leads to the inhibition of metabolic pathways because of the accumulation of Na^+^^3,4^.

The *cation/proton exchanger* (*CHX*) gene family comprises monovalent cation transporters that play critical roles in membrane trafficking and in the development, growth and stress tolerance of plants. *CHX* genes play a role in pH homeostasis in response to environmental changes to adapt to and survive abiotic stress^5^. *A. thaliana* has 28 *CHX* genes, and the rice genome has 17 *CHX* genes^6^. *CHX* plays an important role in maintaining ion homeostasis and is upregulated under salt stress^7^.

*Salt-overly sensitive* (*SOS1*) rice play a critical role in the response to salt stress by controlling Na^+^ homeostasis to lessen the negative effects of salinity^8^. *A. thaliana sos1* mutants grew slowly under high-Na^+^ and low-K^+^ treatment. The *SOS1* gene is upregulated in response to NaCl treatment. The *SOS1* protein is predicted to have 12 domains in its N-terminus. Phylogenetic analysis has shown that *SOS1* is more closely related to microorganisms than to either plants or fungi^9^. The plasma membrane Na^+^/H^+^ transporter *SOS1* is key for Na^+^ transport. The results showed that *AtSOS1* was expressed mainly in roots in response to NaCl to protect plants from salinity^9,10^. In *Puccinellia tenuiflora*, *PtSOS1* expression was strongly upregulated in roots in response to NaCl. The expression of *PtSOS1* significantly increased by 5.8-fold under 25 mM NaCl; in contrast, the expression of *PtSOS1* increased by 1.4-fold in roots under 150 mM NaCl. The expression of *PtSOS1* in shoots was much greater and more persistent under 150 mM NaCl^11^. The results showed that the overexpression of *AjSOS1*, *CrcSOS1*,* CcSOS1* and *CmSOS1* improved the salinity tolerance of the transgenic plants^12^. The expression of *NTAP-SOS2* restored salt tolerance in *sos2-2* plants, demonstrating the fusion protein’s functionality in vivo. This finding highlights *SOS2’s* crucial role in regulating V-ATPase activity, which helps manage ion transport during salt stress and enhance salt tolerance^13^. *SOS3* is vital for enhancing salt tolerance in plants through myristoylation and calcium binding. Mutations that disrupt these processes, like G2A or *sos3-1*, increase the sensitivity of *A. thaliana* plants to NaCl stress. Preserving these mechanisms is crucial for improving crop resilience^14^.

The *LRR-RLK* gene is crucial for how *Medicago truncatula* roots respond to salt stress. The *Srlk* gene activates quickly under these conditions. RNA interference (RNAi) targeting the *Srlk* gene showed that transgenic roots had less growth inhibition in saline environments, indicating its significance for salt resilience^15^. *OsSTLK* (*O. sativa* L. *Salt-Tolerance LRR-RLK*) is essential for enhancing salt tolerance in rice. Research shows that its expression increases significantly under salt stress in both shoots and roots. Additionally, overexpressing *OsSTLK* enhances resistance to salt, making it a critical target for improving tolerance to salt stress^16^. Transgenic pigeon pea lines that overexpress *OsLec-RLK* have shown remarkable resilience under salt stress. This innovative research highlights the potential of the *OsLec-RLK* gene to significantly enhance crop productivity and increase yields in challenging saline conditions^17^. The overexpression of *PnLRR-RLK* in *A. thaliana* significantly elevates the expression of key genes linked to salt stress. This remarkable enhancement not only strengthens the plant’s tolerance to high salinity but also leads to improved seed germination rates and longer primary roots, showcasing its potential for advancing agricultural resilience^18^.

## Methods

### Identification of the *CHX*, *SOS*, and *RLK* genes in *S. lycopersicum*

The genomes of *A. thaliana*,* O. sativa*, *Solanum tuberosum*, and *S. lycopersicum* were downloaded from the Phytozome database^19^. The CHX (accession number: XP_004246149.1), SOS (accession number: NP_001234698.2) and RLK (accession number: XP_004236369.2) proteins were used as query proteins from the NCBI database (https://www.ncbi.nlm.nih.gov/)^20^ (Table S1 Online Resource SI 1) to screen for CHX, SOS and RLK protein members in the genomes of *S. lycopersicum* from the Phytozome database (https://phytozome.jgi.doe.gov)^19^ with an E-value ≤ 1e^−30^ for CHX, SOS and RLK proteins (Table S2 Online Resource SI 1).

### Characterization of the *CHX*, *SOS*, and *RLK* proteins in *S. lycopersicum*

Circoletto (http://tools.bat.infspire.org/circoletto/)^21^ visualized the sequence identity of the CHX, SOS, and RLK proteins. The physical and chemical properties of the CHX, SOS, and RLK proteins, including the molecular weight, isoelectric point, total number of negatively charged residues, total number of atoms, instability, and grand average hydropathicity (GRAVY), were computed using the ExPASy ProtParam Tool^22^.

### Phylogenetic, chromosomal distribution, evolutionary analysis, and synteny analysis of the *CHX*, *SOS*, and *RLK* genes in *S. lycopersicum*

We conducted multiple sequence alignments of the CHX, SOS, and RLK proteins from *S. lycopersicum* using the MUSCLE method. For the analysis of the CHX, SOS, and RLK proteins, we performed a molecular evolutionary genetic study using MEGA-11^23^. This included constructing a phylogenetic tree based on maximum likelihood with 1000 bootstrap replicates, utilizing the WAG model with frequency adjustments. To edit and visualize the phylogenetic tree of the CHX, SOS, and RLK proteins, we used the Itools online website^24^.

Based on the chromosomal positions of the *CHX*, *SOS*, and *RLK* genes, a karyotype map was created using TBtools^25^. The resulting image illustrates the locations of all the *CHX*, *SOS*, and *RLK* genes on the chromosome.

 The rates of synonymous substitutions (Ks) and nonsynonymous substitutions (Ka) for the *CHX*, *SOS*, and *RLK* genes were calculated using TBtools^25^ to investigate selection pressure^26^. The divergence time of the gene pairs was estimated using the synonymous mutation rate measured in substitutions per synonymous site per million years (Mya). The formula employed for this estimation is as follows: “${\mathrm{T}}\, = \,{\mathrm{Ks}}/{\mathrm{2}}\lambda \, \times \,{\mathrm{1}}0\,^{{ - {\mathrm{6}}}} {\text{ }}\left( {\lambda \, = \,{\mathrm{6}}.{\text{56 }} \times {\text{ 1}}0^{{ - {\mathrm{9}}}} } \right)$”^27^. The duplicated genes *CHX*, *SOS*, and *RLK* were categorized as paralogous if the sequence alignment covered at least 70% of the longer gene and if the identity of the aligned region was also at least 70%^28^. Additionally, gene duplication was identified using the gene duplication wizard in MEGA-11^23^. Collinearity analysis of paralogous gene pairs (both tandem and segmental) for *CHX*, *SOS*, and *RLK* was visualized using a Circos plot through TBtools^25^. TBtools^25^ was used to determine the syntenic relationships of the *CHX*, *SOS*, and *RLK* genes in *S. lycopersicum* compared to *S. tuberosum*, *O. sativa* and *A. thaliana*.

### Conserved domain, conserved motif, gene structure analyses, and promoters of the *CHX*, *SOS*, and *RLK* genes in *S. lycopersicum*

The NCBI Conserved Domain Tool^29^ was used to search the Pfam v34.0 PSSM database for CHX, SOS, and RLK proteins. Additionally, the InterPro tool^30^ was employed to analyze the domains present in CHX, SOS, and RLK proteins.

MEME 5.5.5^31^ was utilized to compute the conserved motifs of the CHX, SOS, and RLK proteins. Pfam^32^ was employed for motif description.

The gene structures of *CHX*, *SOS*, and *RLK* proteins sourced from the GFF file were downloaded from the Phytozome database^19^ of the *S. lycopersicum* genome and subsequently illustrated using TBtools^25^.

The promoter sequences of the *CHX*, *SOS*, and *RLK* genes in *S. lycopersicum* were obtained from the genome sequence file, specifically 1500 bp upstream of the transcription start site (TSS) for each gene. These sequences were downloaded from the Phytozome database^19^. Additionally, the cis-regulatory elements (CREs) associated with the *CHX*, *SOS*, and *RLK* genes were analyzed using PlantCARE^33^. A graphical representation of the CRE elements including in the promoter region of the *CHX*, *SOS*, and *RLK* genes was generated via TBTool^25^.

### Subcellular localization, nuclear localization signal, transmembrane helices, and phosphorylation sites of the *CHX*, *SOS*, and *RLK* genes in *S. lycopersicum*

The subcellular localization predictor WoLF PSORT (https://wolfpsort.hgc.jp/)^34^ was utilized to determine the subcellular localization of the CHX, SOS, and RLK proteins. Additionally, TBtools^25^ was employed to visualize the results. For identifying nuclear localization signals (NLSs) in the *CHX*, *SOS*, and *RLK* genes, NLSDB^35^ was used.

The TMHMM server version 2.0^36^ confirmed the presence of transmembrane helical domains (TMs) in the CHX, SOS, and RLK proteins. The NetPhos 3.1 server^37^ was used to predict the phosphorylation sites of the CHX, SOS, and RLK proteins.

### Three-dimensional (3-D) structure prediction and functional interaction network analysis of the *CHX*, *SOS*, and *RLK* proteins in *S. lycopersicum*

The I-TASSER program^38^ and Swiss model^39^ predict the three-dimensional (3-D) structure of the CHX, SOS, and RLK proteins. The STRING database was utilized to analyze the physical interaction network among CHX, SOS, and RLK proteins^40^.

### Prediction of miRNAs targeting the *CHX*, *SOS*, and *RLK* genes in *S. lycopersicum*

The psRNATarget database^41^ and miRBase^42^ were utilized to predict miRNAs of the *CHX*, *SOS*, and *RLK* genes. IPKnot^43^ was employed to predict RNA secondary structures, including pseudoknots, for the CHX, *SOS*, and *RLK* genes.

### Gene Ontology enrichment and functional relationship analysis of the *CHX*,* SOS*, and *RLK* genes in *S. lycopersicum*

ShinyGO 0.77^44^ was utilized for Gene Ontology enrichment analysis. We conducted a gene ontology (GO) annotation analysis by submitting all the gene sequences for *CHX*, *SOS*, and *RLK* to the eggNOG database^45^ and the Phytozome database^19^. The GO annotation data were then processed in SRPLOT^46^ to create a gene ontology chord that illustrates the functional relationships among the *CHX*, *SOS*, and *RLK* genes.

### Tomato plant growth and salinity treatment

The experiments were conducted in the Botany and Microbiology Department at the Faculty of Science, Cairo University. The hybrid F1 tomato seedlings used in this study were generously provided by the Agricultural Research Center (ARC) in Giza, Egypt. A total of thirty seeds were subjected to salt stress by adding 300 mM NaCl to Hoagland’s solution in the potting mix, while another thirty seeds were planted in Hoagland’s solution without NaCl as a control. Both the control and treated plants were harvested 12 h after the salt treatment. Leaves were collected for RNA extraction, gene expression analyses, and sequencing.

### RNA isolation, qRT-PCR expression analysis, and sequencing

This study identified the *CHX* (left primer: ATTCTCTTCCTTGGCCTCGC and right primer: TCGATTCGCCTTCTGGAGTC), *SOS* (left primer: TGATGATTCTGGTGGCGAGG and right primer: AGCGCTTCTTTTTCGCAATACAT) and *RLK* genes (left primer: ACTCGGCTATGTGTAGGCAA and right primer: GTTGTGCATACTCAGGGGCT). “Total RNA was isolated from the leaves of 15-day-old *S. lycopersicum* plants using a GeneTireX kit. The residual DNA was removed with RNase-free recombinant DNase I (Thermo Scientific, Lithuania). First-strand cDNA was synthesized in a 20 µL reaction mixture using a Grisp reverse transcription kit, incorporating approximately two micrograms of DNA-free total RNA from each sample. Quantitative real-time PCR (qRT-PCR) was conducted to quantify the relative transcription levels of the *CHX*, *SOS*, and *RLK* genes expressed in the leaves. The qPCR was performed with a CFX Connect Real-Time PCR System (Bio-Rad, Singapore) under the following conditions: an initial denaturation at 94 °C for 5 min; followed by 40 cycles of denaturation at 94 °C for 10 s, annealing at 58 °C for 20 s, and extension at 72 °C for 30 s. A plate read was taken, followed by a melt curve analysis from 65 to 95 °C with a 0.5 °C increment for 10 seconds, and subsequent sequencing. The Ct (cycle threshold) value was employed as a measure of the initial copy number of the target gene^47^. The relative gene expression level was calculated using the 2-ΔΔCT method^48^. TIP 41 (TAP42-interacting protein) served as the internal reference gene. The forward primer used was ATGGAGTTTTTGAGTCTTCTGC, and the reverse primer was GCTGCGTTTCTGGCTTAGG”.

## Results

### Identification of the *CHX*, *SOS*, and *RLK* genes in *S. lycopersicum*

A total of 21 *CHX*, 5 *SOS*, and 86 *RLK* candidate genes were retrieved from the *S. lycopersicum* genome and were named according to their chromosomal positions from SlCHX-1 to SlCHX-21, SlSOS-1 to SlSOS-5 and SlRLK-1 to SlRLK-86 for the *CHX*, *SOS* and *RLK* genes, respectively (Table S2 Online Resource SI 1).

### Characterization of the *CHX*, *SOS*, and *RLK* proteins in *S. lycopersicum*

**Fig. 1:**
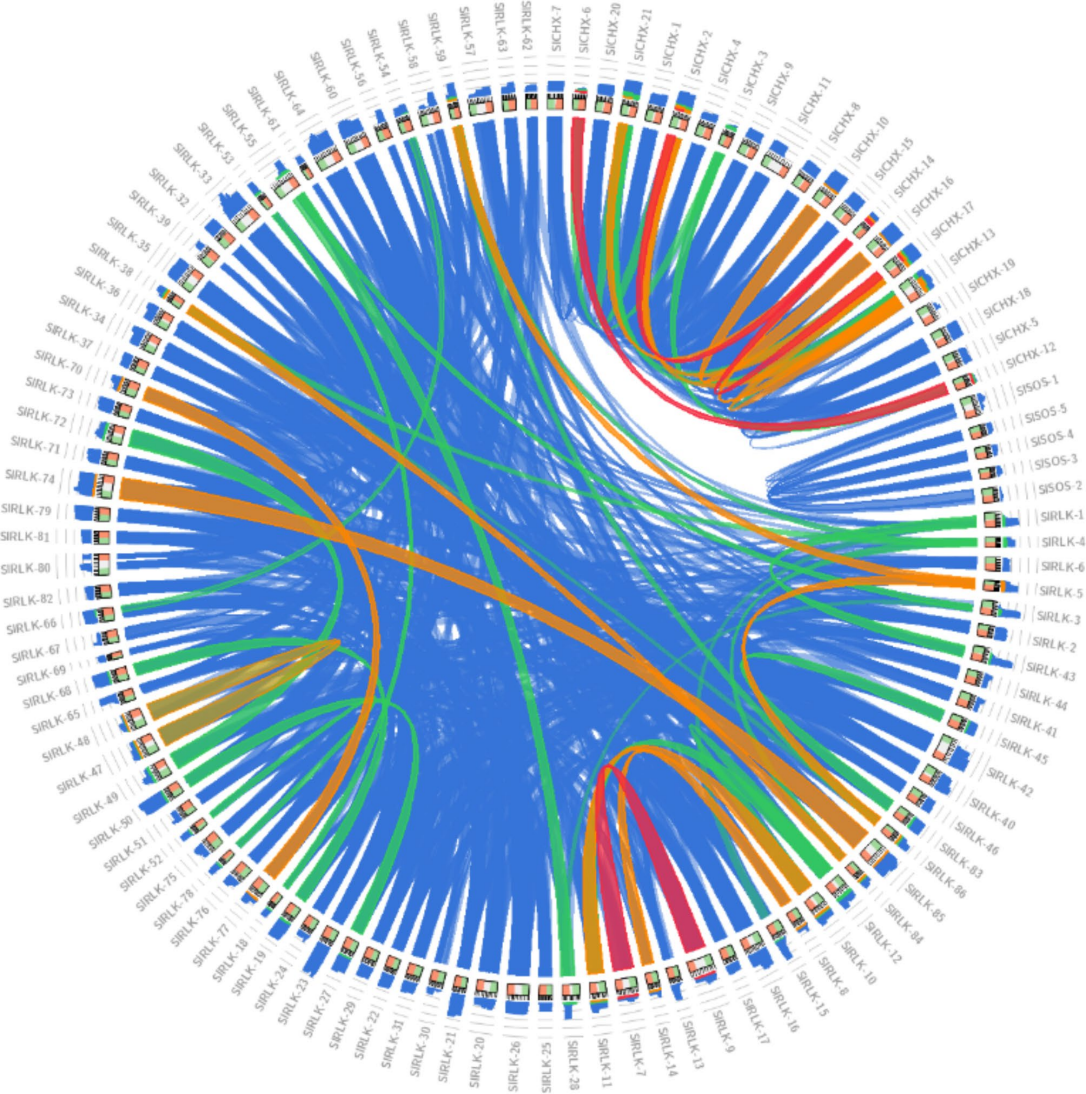
Sequence identity of the 21 CHX, 5 SOS, and 86 RLK proteins.

The sequence identities of 21 *CHX*, 5 *SOS*, and 86 RLK proteins are shown by the color-by-E-value ratio (blue, ≤ 60%; green, ≤ 80%; orange, ≤ 90%; red otherwise), as shown in Fig. 1. Analysis of protein physical and chemical properties revealed that the length of the CHX family of amino acids in S. lycopersicum ranged from 487 (SlCHX-14) to 1359 (SlCHX-11). The length of the SOS family amino acids ranged from 522 (SlSOS-2) to 1019 (SlSOS-1). The length of the RLK family amino acids ranged from 304 (RLK-69) to 1253 (RLK-42). The molecular weights (MWs) of CHX ranged from 52394.64 (SlCHX-14) to 149526.32 (SlCHX-11). The molecular weights of the SOSs ranged from 57577.43 (SlSOS-2) to 112152.85 (SlSOS-1). The molecular weights of the RLKs ranged from 33927.03 (RLK-69) to 139023.34 (RLK-42). The isoelectric point (PI) of CHX ranged from 4.75 (SlCHX-19) to 9.45 (SlCHX-14). The isoelectric point of SOS ranged from 5.63 (SlSOS-2) to 7.24 (SlSOS-5). The isoelectric point (PI) of RLK ranged from 5.24 (RLK-67) to 9.35 (RLK-78). The total number of atoms in CHX ranged from 7573 (SlCHX-14) to 21,077 (SlCHX-11). The total number of atoms in the SOS ranged from 8090 (SlSOS-2) to 15,890 (SlSOS-1). The total number of atoms in the RLK ranged from 4783 (RLK-69) to 19,524 (RLK-42). The average hydropathicity value (GRAVY) of CHX ranged from 0.036 (SlCHX-19) to 0.718 (SlCHX-14). The average hydropathicity value of the SOS ranged from 0.092 (SlSOS-1) to 0.549 (SlSOS-5). The average hydropathicity value of RLK ranged from − 0.59 (SlRLK-52) to − 0.103 (RLK-20) (Table S2 Online Resource SI 1).

### Phylogenetic, chromosomal distribution, evolutionary analysis, and synteny analysis of the *CHX*, *SOS*, and *RLK* genes in *S. lycopersicum*

**Fig. 2:**
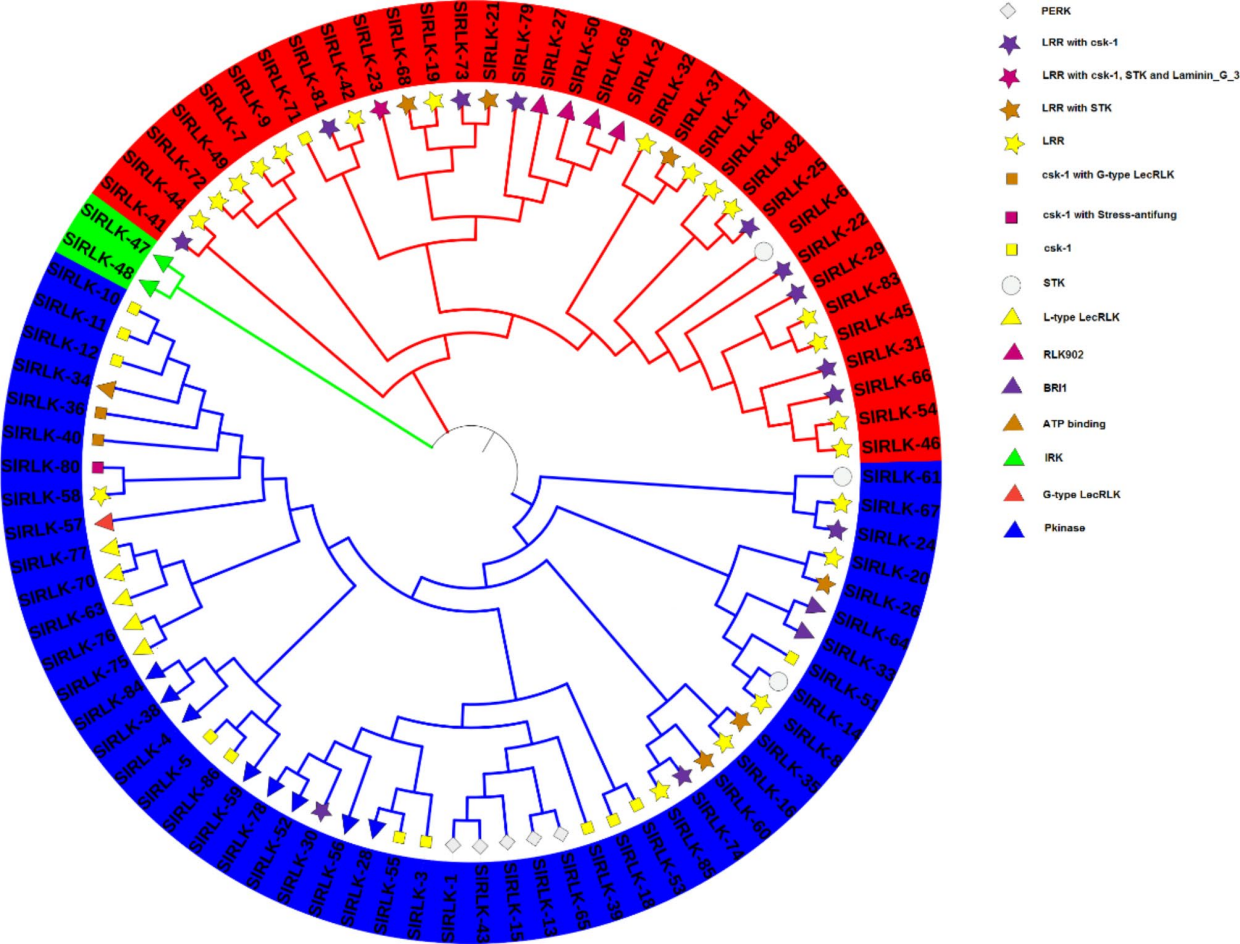
Maximum likelihood phylogenetic tree of the RLK protein family in *S. lycopersicum*.

A phylogenetic tree was constructed using maximum likelihood with 1,000 bootstrap replicates. The CHX, SOS, and RLK protein sequences were analyzed to investigate their potential evolutionary history. In the resulting phylogenetic tree, the CHX, SOS (Fig. S1 Online Resource SI 2), and RLK proteins (Fig. 2) were classified into three distinct clades. A phylogenetic tree categorized the SlRLK protein family by domain function based on the Phytozome-13 website^19^ against the InterPro Description. A summary of the SlRLK proteins is as follows: The SlRLK protein family comprises a variety of proteins distinguished by specific structural domains. Notably, one SlRLK protein features a leucine-rich repeat (LRR) alongside a serine-threonine/tyrosine-protein kinase catalytic domain (csk-1), a serine/threonine/dual specificity protein kinase (STK), and a concanavalin A-like lectin/glucanase domain (Laminin_G_3). In total, 21 SlRLK proteins possess leucine-rich repeats (LRRs). Among these: 12 SlRLK proteins have serine-threonine/tyrosine-protein kinase catalytic domains (csk-1). 6 SlRLK proteins include serine/threonine/dual specificity protein kinases (STKs). − 5 SlRLK proteins contain proline-rich receptor (PERK). 2 SlRLK proteins have serine-threonine/tyrosine-protein kinase catalytic domains (csk-1) combined with an S-locus glycoprotein domain (G-type LecRLKs). 1 SlRLK protein features a serine-threonine/tyrosine-protein kinase catalytic domain (csk-1) associated with salt stress response/antifungal activity. 3 SlRLK proteins contain serine/threonine/dual specificity protein kinases (STK). 5 SlRLK proteins have a lectin domain (L-type LecRLKs). 4 SlRLK proteins are related to the inactive receptor kinase rlk902 (RLK902). 2 SlRLK proteins include a BRI1-like receptor kinase (BRl1). 1 SlRLK protein contains an ATP binding/protein kinase-related domain. 2 SlRLK proteins feature an inflorescence and root apices receptor-like kinase (IRK). 1 SlRLK protein contains an S-locus glycoprotein domain (G-type LecRLKs). 8 SlRLK proteins have a protein kinase domain (Pkinase). This overview highlights the complexity and variety within the SlRLK protein family (Fig. 2).

**Fig. 3:**
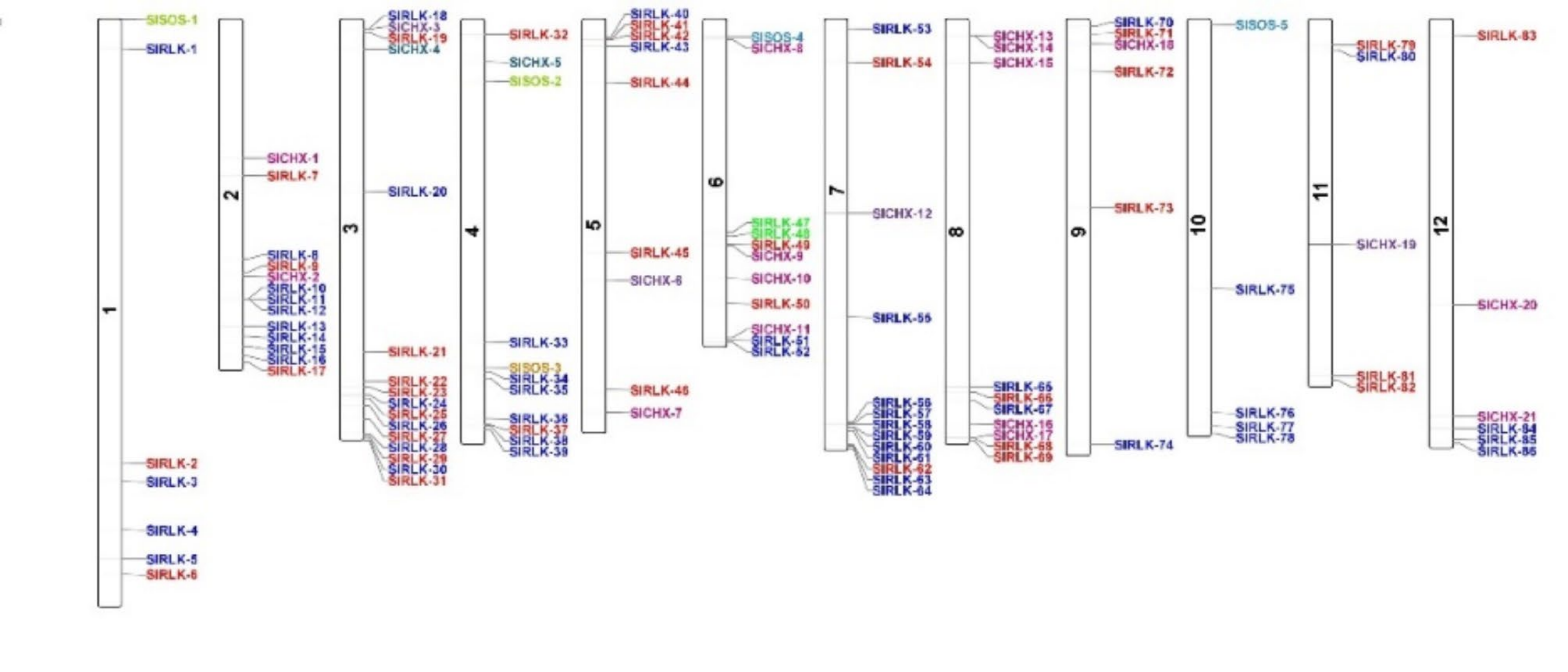
Distribution of the *CHX*, *SOS*, and *RLK* genes on *S. lycopersicum* chromosomes.

Based on the information available on the Phytozome-13 website^19^, the *CHX*, *SOS*, and *RLK* genes were physically drawn on the chromosomes in the *S. lycopersicum* genome. *CHX* genes were found on chromosomes 2, 3, 4, 5, 6, 7, 8, 9, 11 and 12 of *S. lycopersicum*. *SOS* genes were found on chromosomes 1, 4, 6, and 10. *RLK* genes were found on all chromosomes of *S. lycopersicum* (Fig. 3).

The selective pressure on the *CHX*, *SOS*, and *RLK* genes was investigated by calculating the nonsynonymous/synonymous ratio (*Ka*/*Ks*). A *Ka/Ks* ratio < 1 or Ka < Ks suggested purifying selection, a *Ka*/*Ks* ratio = 1 or Ka = Ks indicated neutral selection, and a *Ka*/*Ks* ratio > 1 or Ka > Ks suggested positive selection^49^. In the current study, the *Ka*/*Ks* ratios of the *CHX*, *SOS*, and *RLK* paralogous pairs were < 1, which indicates that the *CHX*, *SOS*, and *RLK* genes were influenced primarily by purifying selection, these results suggest that the *CHX*, *SOS*, and *RLK* genes received strong environmental pressure during evolution (Table 1). Purifying selection leads to a decrease in genetic diversity^50^ by a decrease in species diversity and reduced evolutionary change between species compared to neutral sites^51^.

**Fig. 4:**
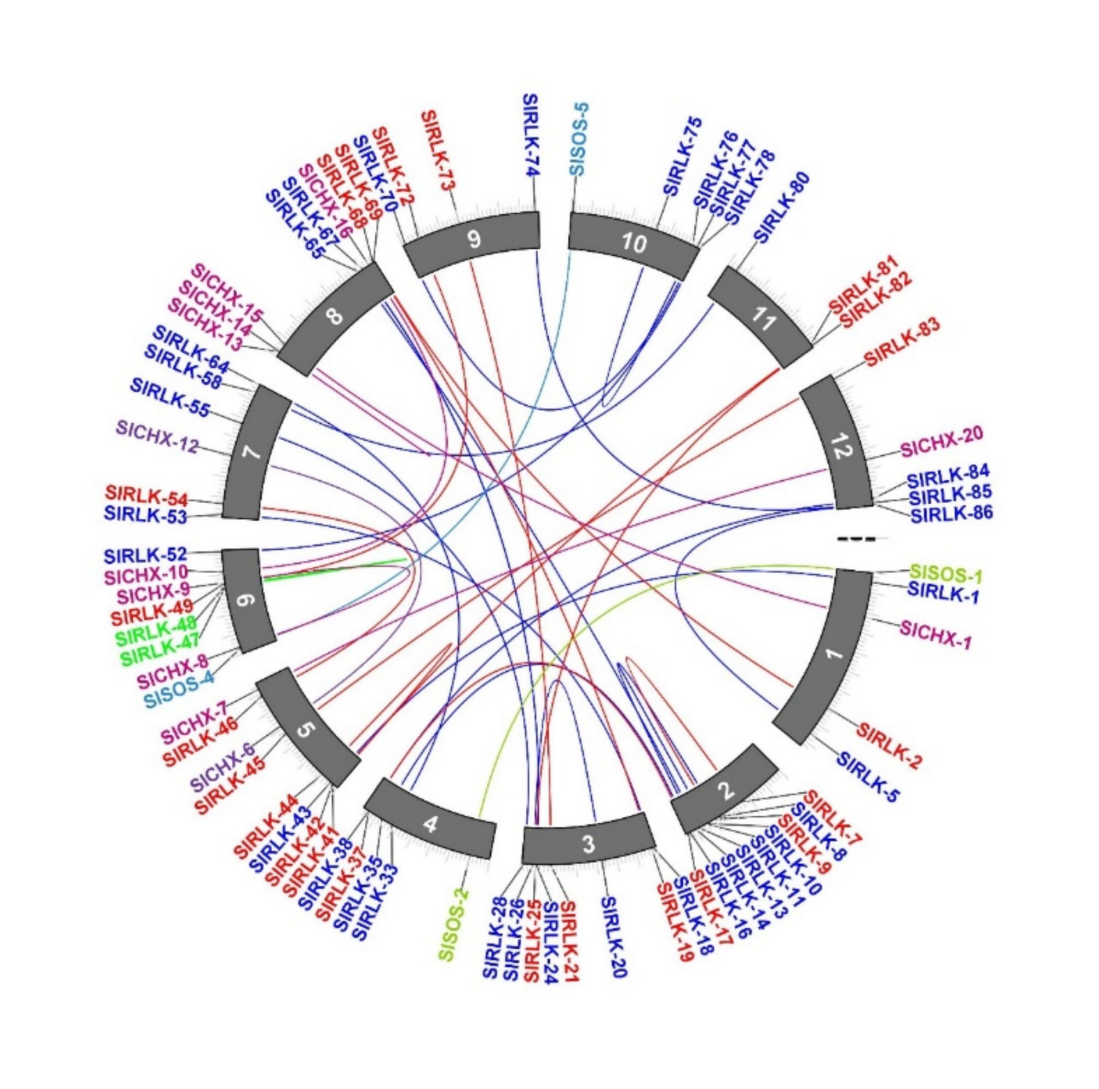
Segmental and tandem duplications of *CHX*, *SOS*, and *RLK* among the *S. lycopersicum* chromosomes.

The duplication time of the *CHX* paralogous gene pairs in *S. lycopersicum* ranged from approximately 26.965 to 245.413 Mya. The duplication time of the *SOS* paralogous gene pairs ranged from approximately 116.682 to 275.631 Mya. The duplication time of the *RLK* paralogous gene pairs ranged from approximately 27.689 to 239.376 Mya (Table 1; Fig. 4).

**Table 1:** Paralogous pairs of *CHX*, *SOS* and *RLK* genes and the *Ka*/*Ks* ratio.

locus 1	locus 2	Ka	Ks	Ka_Ks	Time (Mya)
*SlCHX-6*	*SlCHX-12*	0.08140903	0.565064848	0.144070242	43.0689671
*SlCHX-13*	*SlCHX-14*	0.093164207	0.35378587	0.263335014	26.96538645
*SlCHX-15*	*SlCHX-1*	0.559876525	1.961245152	0.285469935	149.4851488
*SlCHX-16*	*SlCHX-10*	0.164523365	0.831884763	0.197771822	63.40585084
*SlCHX-20*	*SlCHX-7*	0.584661825	2.223616882	0.262932805	169.4829941
*SlCHX-8*	*SlCHX-9*	0.596005622	3.219828003	0.185104801	245.4137197
*SlSOS-1*	*SlSOS-2*	0.469247446	1.530870221	0.306523336	116.6821815
*SlSOS-4*	*SlSOS-5*	0.342621954	3.616284419	0.094744194	275.6314343
*SlRLK-47*	*SlRLK-48*	0.129435915	0.727753899	0.177856711	55.46904716
*SlRLK-41*	*SlRLK-44*	0.51813339	2.276296996	0.227621172	173.4982467
*SlRLK-49*	*SlRLK-72*	0.233086231	1.627697126	0.143200001	124.0622809
*SlRLK-7*	*SlRLK-9*	0.103879031	0.721766877	0.143923246	55.0127193
*SlRLK-81*	*SlRLK-42*	0.49946405	2.499837185	0.199798632	190.5363708
*SlRLK-19*	*SlRLK-68*	0.306000758	2.037369231	0.150194061	155.287289
*SlRLK-73*	*SlRLK-21*	0.291568551	3.10571349	0.093881342	236.7159672
*SlRLK-2*	*SlRLK-69*	0.44919416	1.81742754	0.247159323	138.5234405
*SlRLK-17*	*SlRLK-37*	0.341975042	3.14061592	0.10888789	239.3762134
*SlRLK-25*	*SlRLK-82*	0.466624923	2.276143936	0.205006773	173.4865805
*SlRLK-83*	*SlRLK-45*	0.240439037	1.238328672	0.194164152	94.38480733
*SlRLK-54*	*SlRLK-46*	0.292222702	1.490059925	0.196114732	113.5716406
*SlRLK-20*	*SlRLK-26*	0.7317167	2.694697415	0.271539467	205.3885225
*SlRLK-64*	*SlRLK-33*	0.482745332	2.90363316	0.16625562	221.3135031
*SlRLK-14*	*SlRLK-8*	0.146582737	0.649730629	0.225605398	49.5221516
*SlRLK-74*	*SlRLK-85*	0.16042137	0.612717693	0.261819386	46.70104368
*SlRLK-53*	*SlRLK-18*	0.329472776	1.281116438	0.257176293	97.64606996
*SlRLK-65*	*SlRLK-13*	0.503439631	1.888616668	0.26656528	143.9494411
*SlRLK-43*	*SlRLK-1*	0.254174179	0.623750884	0.407493096	47.54198813
*SlRLK-55*	*SlRLK-28*	0.355765098	3.034274418	0.117248821	231.270916
*SlRLK-86*	*SlRLK-5*	0.176236048	1.898918305	0.092808652	144.7346269
*SlRLK-38*	*SlRLK-84*	0.120019716	0.78214511	0.153449423	59.61471877
*SlRLK-70*	*SlRLK-77*	0.124287078	0.877997707	0.141557406	66.9205569
*SlRLK-10*	*SlRLK-11*	0.150575001	0.363281132	0.414486158	27.68911065

**Fig. 5:**
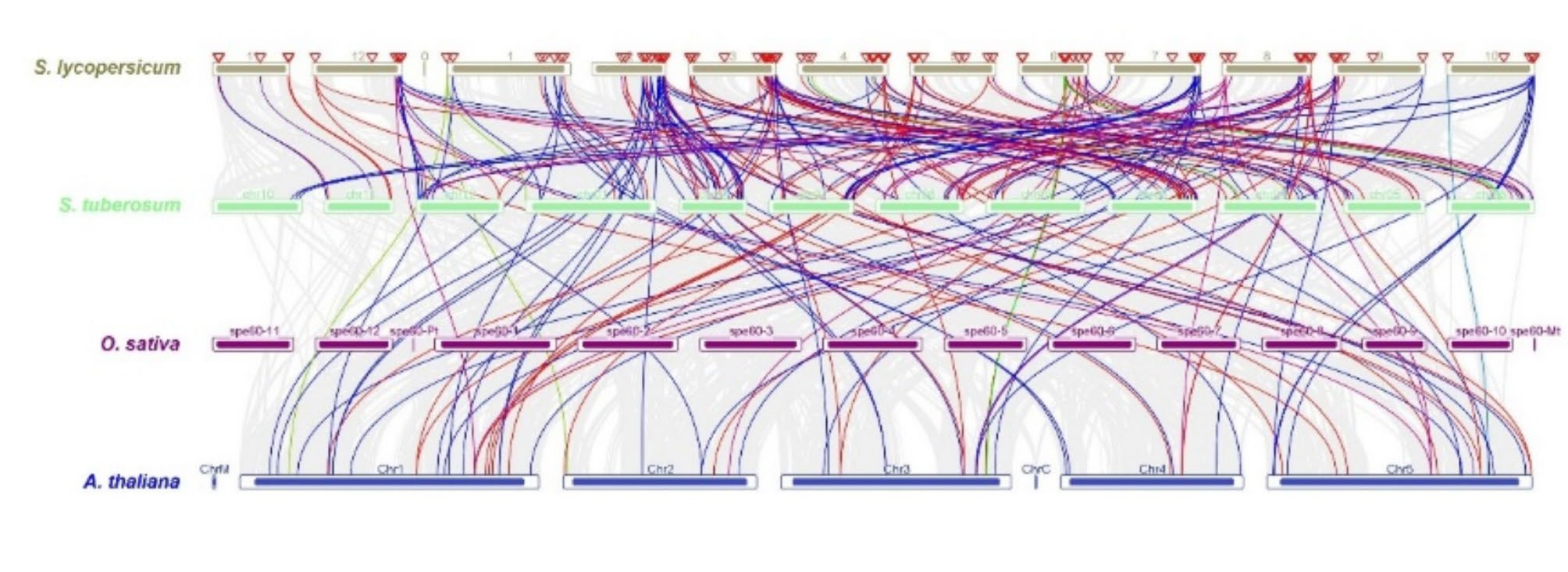
The collinear relationships of the *CHX*, *SOS*, and *RLK* genes are shown as colored lines in the phylogenetic tree.

The *CHX*, *SOS*, and *RLK* genes were analyzed for interspecies collinearity to determine the orthologous relationships of *S. lycopersicum* against *S. tuberosum*,* O. sativa*, and *A. thaliana*. Collinearity analysis revealed robust orthologs of the *CHX*, *SOS*, and *RLK* genes among *S. lycopersicum* compared with those of the other three plant species, the *CHX*, *SOS*, and *RLK* genes revealed collinearity orthologous relationships in *A. thaliana* but no collinearity orthologous relationships in *O. sativa*. In addition, collinearity analysis revealed that 6 orthologous *SlCHX* genes, 2 orthologous *SlSOS* genes, and 44 orthologous *SlRLK* genes were paired with those in *A. thaliana*. Also, collinearity analysis revealed that 24 orthologous *SlCHX* genes, 4 orthologous *SlSOS* genes, and 105 orthologous *SlRLK* genes were paired with those in *S. tuberosum* (Fig. 5 and Table S3 Online Resource SI 1).

### Conserved domain, conserved motif, gene structure, and promoter analyses of the *CHX*,* SOS*, and *RLK* genes in *S. lycopersicum*

**Fig. 6:**
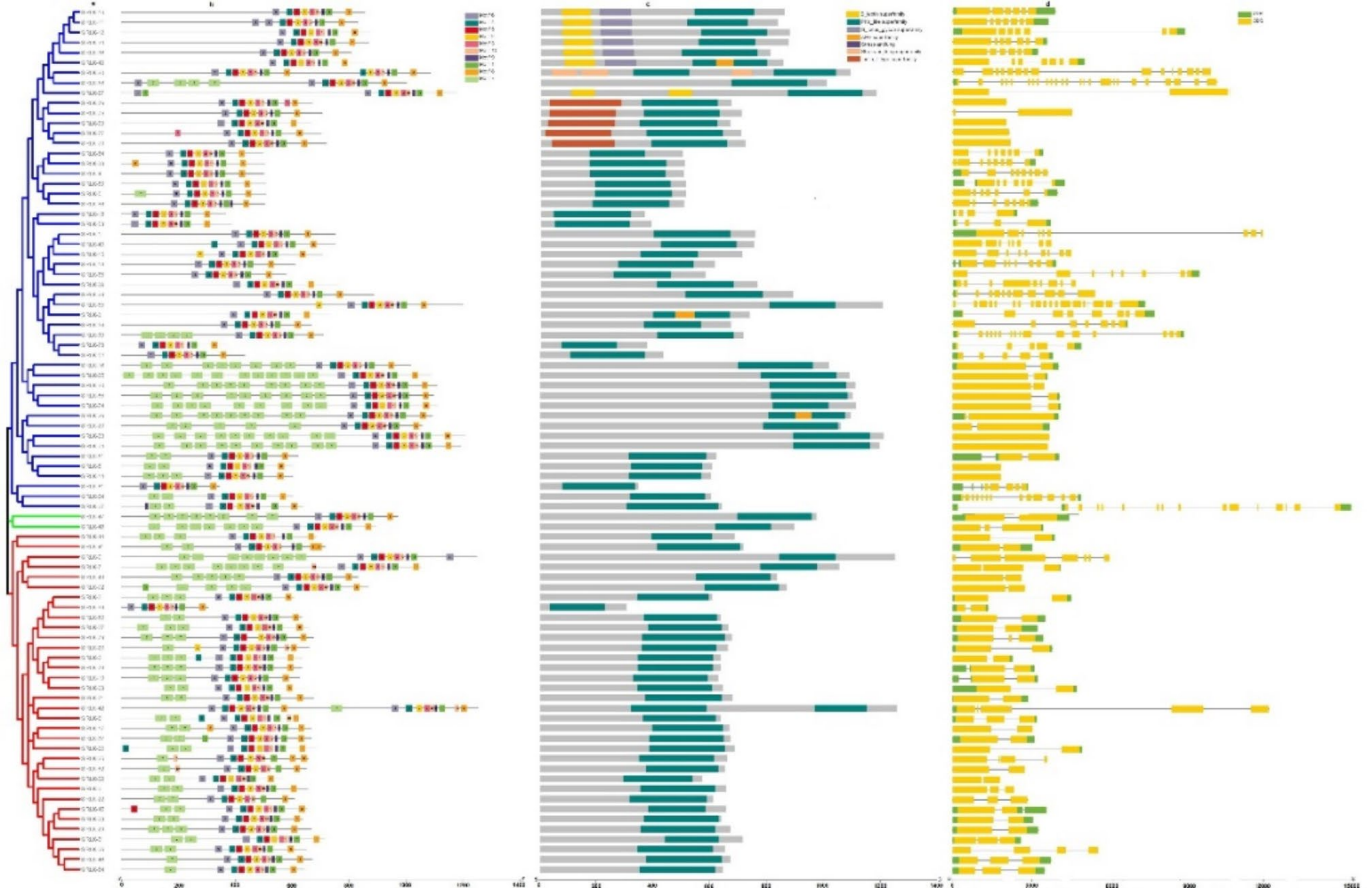
RLK proteins. (**a**) Rectangular phylogenetic tree. (**b**) Conserved motifs were predicted using MEME. (**c**) Protein domains. (**d**) Gene structure.

Domain analysis for 21 CHX, 5 SOS, and 86 RLK proteins confirmed the presence of the Na_H_Exchanger superfamily domain (Fig. S2, Online Resource SI 2), the Na_H_Exchanger superfamily domain (Fig. S3 Online Resource SI 2), and the PKc_like superfamily domain (Fig. 6), respectively.

Motif analysis for 21 CHX, 5 SOS, and 86 RLK proteins confirmed that the phylogenetic relationships were similar to the conserved motif distributions within the clade, with few differences. For instance, the CHX motif distributions for the SlCHX-9, SlCHX-8, SlCHX-11, SlCHX-7, SlCHX-20, SlCHX-10, SlCHX-16, SlCHX-1, SlCHX-15, SlCHX-18, SlCHX-21, SlCHX-2, and SlCHX-17 proteins revealed conserved motif numbers of 1, 2, 3, 4, 5, 6, 7, 8, 9, and 10 (Fig. S2 Online Resource SI 2 and Table S4 Online Resource SI 1). The SOS motif distributions for the SlSOS-5 and SlSOS-4 proteins revealed conserved motif numbers of 1, 2, 3, 4, 5, 6, and 9. The motif distributions for the SlSOS-1 and SlSOS-2 proteins revealed conserved motif numbers 3, 4, 5, 7, 8, and 10 (Fig. S3 Online Resource SI 2 and Table S5 Online Resource SI 1). The RLK proteins presented conserved motif numbers of 1, 2, 3, 4, 5, 6, 8, and 9 (Fig. 6 and Table S6 Online Resource SI 1).

The exon-intron structure is an important source of plant biodiversity and gene family evolution. The gene structure results revealed that 21 *CHX*, and 5 *SOS* genes had introns (Fig. S2 and Fig. S3 Online Resource SI 2). The gene structure results revealed that 86 *RLK* genes had introns, while *SlRLK-76*, *SlRLK-63*, *SlRLK-77*, *SlRLK-70*, *SlRLK-33*, *SlRLK-64*, *SlRLK-14*, and *SlRLK-8* did not have introns (Fig. 6).

**Fig. 7:**
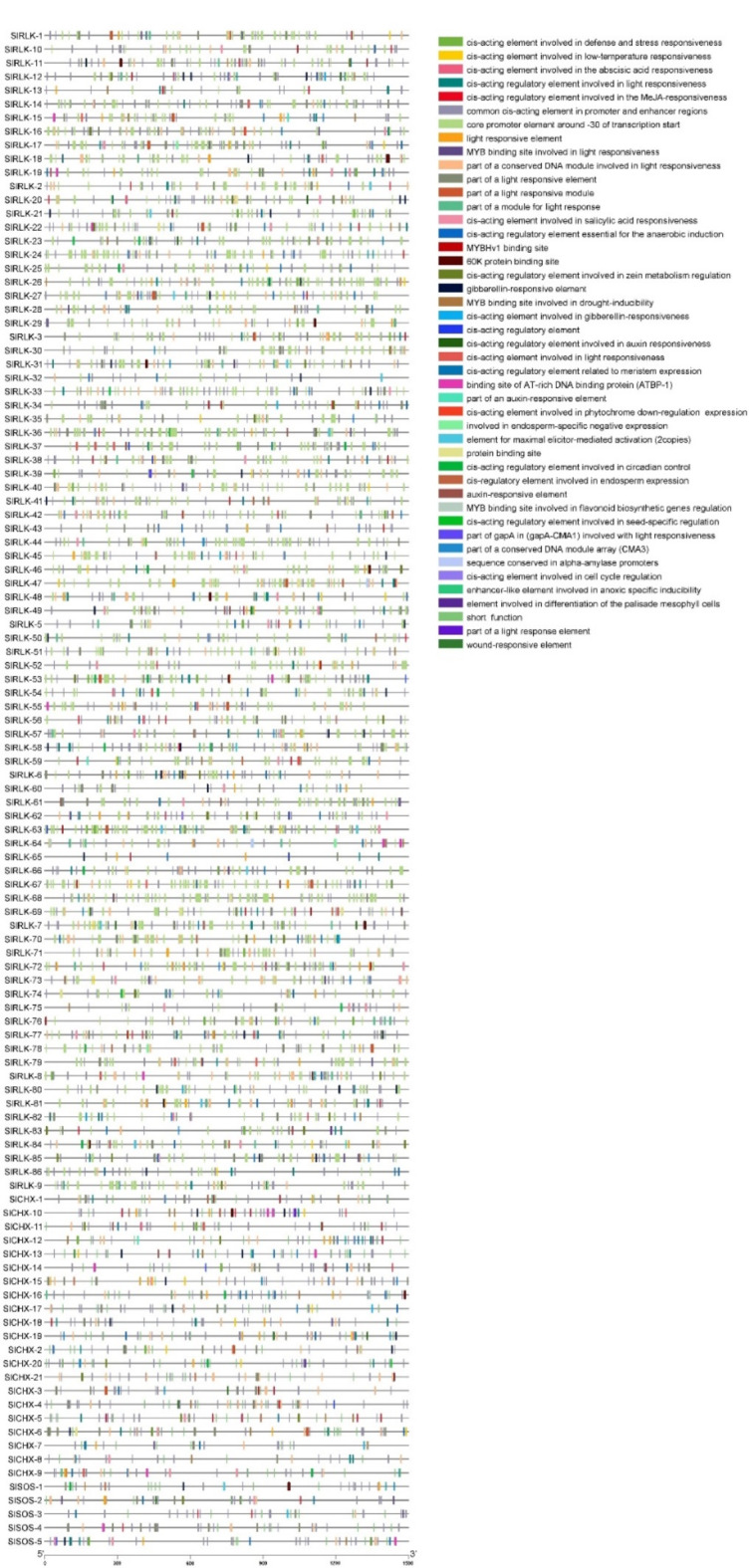
Cis-acting elements in the promoter regions (1500 bp upstream of the start codon) of the 21 *CHX*, 5 *SOS*, and 86 *RLK* genes.

The 21 *CHX*, 5 *SOS*, and 86 *RLK* gene sequences (1500 bp upstream of the start codon) (Table S2 Online Resource SI 1) were selected for cis-element analysis using the PlantCARE web tool to identify their biological functions (stress response, growth, and development). The promoter regions of the 21 *CHX*, 5 *SOS*, and 86 *RLK* genes in *S. lycopersicum* contain a large number of plant hormone response elements and stress responses. Most 21 CHX, 5 SOS, and 86 RLK proteins contain defense and stress response elements, abscisic acid-responsive elements, methyl jasmonate (MeJA)-responsive elements, salylic acid, and the MYB binding site (MBS) elements, which are involved in the salinity response (Fig. 7).

### Subcellular localization, nuclear localization signal, transmembrane helices, and phosphorylation sites of the CHX, SOS, and RLK proteins in *S. lycopersicum*

Subcellular localization analysis revealed that all 21 CHX and 5 SOS proteins were located in the plasma membrane, whereas 86 RLK proteins were located in the plasma membrane, except SlRLK-9, SlRLK-24, SlRLK-39, and SlRLK-61 proteins were located in the chloroplasts, and SlRLK-18, SlRLK-78, and SlRLK-69 proteins were located in the cytoplasm (Fig. S4 Online Resource SI 2 and Table S7 Online Resource SI 1).

Four putative nuclear localization signals (NLSs) were predicted for 5 SlCHX proteins, 13 putative nuclear localization signals (NLSs) were predicted for 13 SlRLK proteins, and no nuclear localization signals (NLSs) were predicted for SlSOS proteins (Table S8 Online Resource SI 1).

The TMHMM results predicted the presence of transmembrane helices in all 21 CHX, 5 SOS and 86 RLK proteins, except for SlRLK-18, SlRLK-39, SlRLK-52, SlRLK-53, SlRLK-69, and SlRLK-78, which are nontransmembrane hydrophobic helices (Fig. S5: Fig. S23 Online Resource SI 2 and Table S9 Online Resource SI 1).

The phosphorylation site prediction results for the 21 CHX, 5 SOS and 86 RLK proteins for kinases (ATM, CKI, CKII, CaM-II, DNAPK, EGFR, GSK3, INSR, PKA, PKB, PKC, PKG, RSK, SRC, cdc2, cdk5 and p38MAPK) are shown in Fig. S24: Fig. S42 Online Resource SI 2 and Table S10 Online Resource SI 1.

### Three-dimensional (3-D) structure prediction, and functional interaction network analysis of the CHX, SOS, and RLK proteins in *S. lycopersicum*

**Fig. 8:**
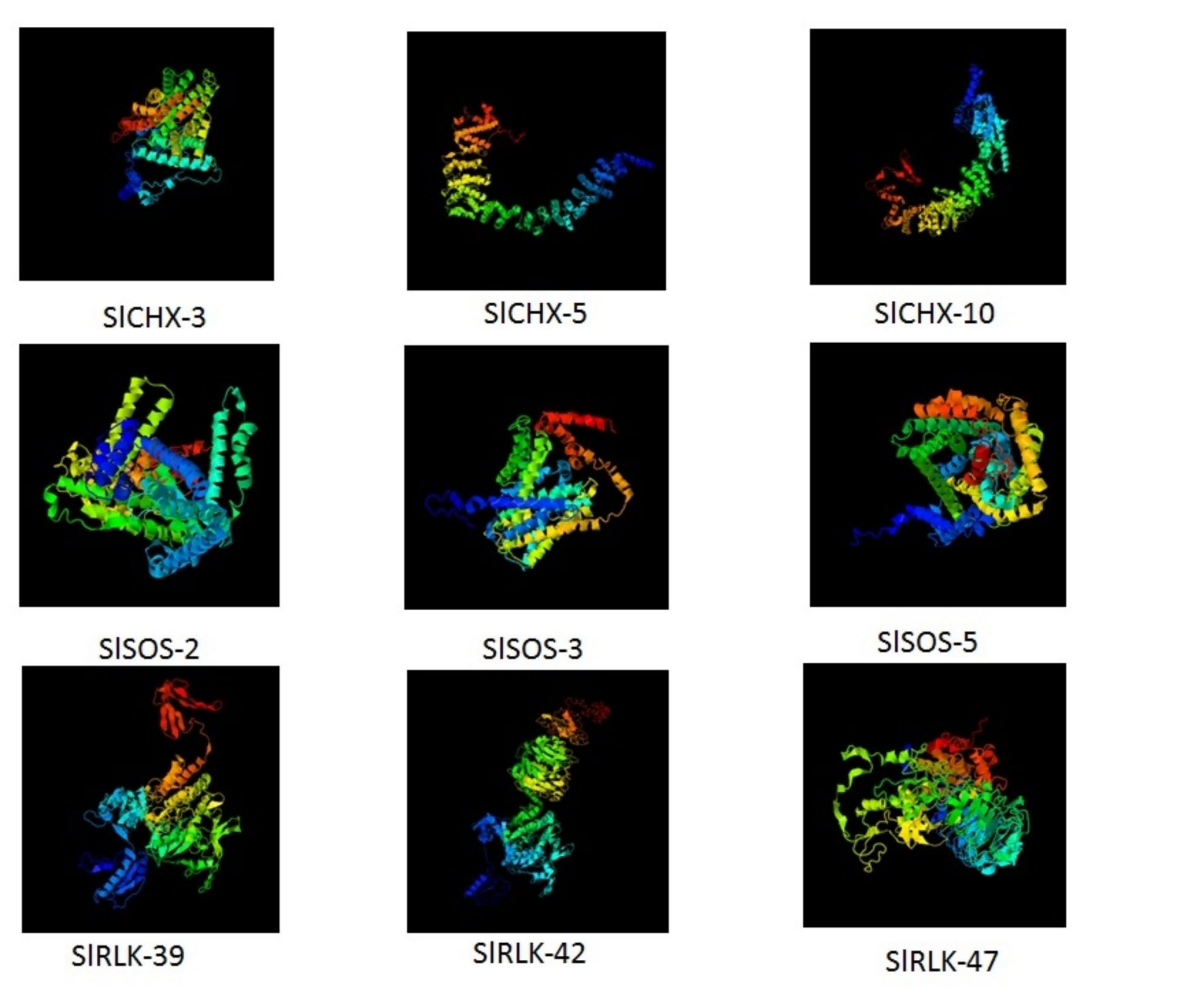
Structural analysis of the SlCHX-3, SlCHX-5, SlCHX-10, SlSOS-2, SlSOS-3, SlSOS-5, SlRLK-39, SlRLK-42, and SlRLK-47 proteins.

To study the putative functions of the CHX, SOS, and RLK proteins in S. lycopersicum, we selected a protein from each clade. The SlCHX-3, SlCHX-5, SlCHX-10, SlSOS-2, SlSOS-3, SlSOS-5, SlRLK-39, SlRLK-42 and SlRLK-47 proteins were modeled with I-TASSER software to construct 3-D structures. The 3-D structures were generated according to similar crystal structures and structural templates obtained from the Protein Data Bank (Fig. 8). Due to their structural similarity, proteins that are structurally similar to the target in the PDB often have similar functions. C-scores were used to estimate the degree of prediction confidenc*e* of the generated protein model for the SlCHX-3, SlCHX-5, SlCHX-10, SlSOS-2, SlSOS-3, SlSOS-5, SlRLK-39, SlRLK-42, and SlRLK-47 proteins as shown in Table 2. The C-scores indicated that the structures of the CHX, SOS, and RLK proteins were generated with high accuracy. The remaining proteins were modeled with the Swiss model (Fig. S43: Fig. S61 Online Resource SI 2).

To thoroughly investigate the significant roles of CHX, SOS, and RLK in protein-protein interactions (PPIs) with other proteins, we confidently constructed a detailed protein-protein interaction (PPI) network using the Search Tool for the Retrieval of Interacting Genes/Proteins (STRING) database. This proactive approach will provide essential insights into these critical interactions and their implications. CHX proteins are hypothesized to interact with several other proteins. For example, SlCHX-3 is hypothesized to interact with the following: F-actin-capping protein subunit alpha (A0A3Q7ENK3), TPT domain-containing protein (A0A3Q7F1T7), TPT domain-containing protein (A0A3Q7FAX7), and an additional F-actin-capping protein subunit alpha (A0A3Q7J0C0). SlCHX-16 is hypothesized to interact with the following: Usp domain-containing protein (A0A3Q7G7V0), Usp domain-containing protein (A0A3Q7EZD3), and Usp domain-containing protein (A0A3Q7JYU7). SlCHX-18 is hypothesized to interact with the following: Lipase_GDSL domain-containing protein (A0A3Q7IV68), Na_H_Exchanger domain-containing protein (A0A3Q7IB11), F-actin-capping protein subunit alpha (A0A3Q7ENK3), F-actin-capping protein subunit alpha (A0A3Q7J0C0), RCK N-terminal domain-containing protein (A0A3Q7EKS0), CitMHS domain-containing protein (A0A3Q7GUH1), and CitMHS domain-containing protein (A0A3Q7FEL4), as illustrated in Fig. S62: Fig. S64 of Online Resource SI 2.

SOS proteins are hypothesized to interact with several other proteins. For example, the SlSOS1 protein (A0A3Q7E704) is hypothesized to interact with various putative proteins, including Endonuclease III homolog (MLOC_61349.1, NTH1), APX (Q52QQ4), CBL10 (G4XMX1), CNGC15 (A0A3Q7FNS3), HKT1;2 (A0A3Q7H8N9), LKT1 (A0A3Q7J386), NHX1 (A0A3Q7GSL5), NHX3 (A0A3Q7EGG6), PRO2 (Q96480), SOS2 (A0A3Q7J468), and SlCBL1 (G5EM33). SlSOS3 protein (A0A3Q7E704) is hypothesized to interact with various putative proteins, including NADH-cytochrome b5 reductase (A0A3Q7IM46), Na_H_Exchanger domain-containing protein (A0A3Q7FFK2), A0A3Q7FRU0, Hop-interacting protein THI141 (G8Z288), DNA_mis_repair domain-containing protein (A0A3Q7G2Q3), A0A3Q7EET5 and A0A3Q7IJ47. SlSOS4 protein (A0A3Q7E704) is hypothesized to interact with various putative proteins, including Plasma membrane ATPase 2 (P23980), A0A3Q7H717, A0A3Q7H8N9, Delta-1-pyrroline-5-carboxylate synthase (Q96480), Ascorbate peroxidase (Q52QQ4), Calcineurin B-like molecule (G4XMX1), Non-specific serine/threonine protein kinase (A0A3Q7J468), Calcineurin B-like protein 1 (G5EM33), A0A3Q7J386, and A0A3Q7FV11, as illustrated in Fig. S65 and Fig. S66 of Online Resource SI 2.

RLK proteins are hypothesized to interact with several other proteins. For example, the SlRLK-1 is hypothesized to interact with various putative proteins, including Protein kinase domain-containing protein (A0A3Q7G4Z1), Protein kinase domain-containing protein (A0A3Q7ESK3), Protein kinase domain-containing protein (A0A3Q7FY35), Protein kinase domain-containing protein (K4CEZ6), Protein kinase domain-containing protein (A0A3Q7FK54), Protein kinase domain-containing protein (A0A3Q7EU17), Protein kinase domain-containing protein (A0A3Q7IZP0), Protein kinase domain-containing protein (A0A3Q7GR61), A0A3Q7GZ94, and Protein kinase domain-containing protein (A0A3Q7EQ44). SlRLK-12 is hypothesized to interact with various putative proteins, including A0A3Q7F6S0, A0A3Q7JW86, A0A3Q7GI92, A0A3Q7J864, Protein kinase domain-containing protein (A0A3Q7ESK3), Protein kinase domain-containing protein (K4B6S3), Protein kinase domain-containing protein (A0A3Q7EZC6), Protein kinase domain-containing protein (A0A3Q7FJB6), ZZ-type domain-containing protein (A0A3Q7HUM2), and A0A3Q7FQ22. SlRLK-86 is hypothesized to interact with various putative proteins, including Protein kinase domain-containing protein (A0A3Q7IZP0), Protein kinase domain-containing protein (A0A3Q7EZC6), Protein kinase domain-containing protein (K4B6S3), Protein kinase domain-containing protein (K4B6S2), A0A3Q7E900, Protein kinase domain-containing protein (A0A3Q7GR61), A0A3Q7GZ94, A0A3Q7IZ15, Protein kinase domain-containing protein (A0A3Q7HSB8), and Protein kinase domain-containing protein (A0A3Q7I7W0), as illustrated in Fig. S67: Fig. S94 in Online Resource SI 2.

**Table 2:** Modeling parameters for the CHX, SOS, and RLK proteins.

Protein	C-Score	TM-Score	RMSD (Å)	Best identified structural analogs in PDB				
				PDB Hit	TM-Score ^a^	RMSD ^a^	IDEN ^a^	Cov
SlCHX-3	− 1.76	0.50 ± 0.15	11.9 ± 4.5	4bwzA	0.666	0.33	0.206	0.667
SlCHX-5	− 0.88	0.60 ± 0.14	10.4 ± 4.6	7yxxA	0.932	2.10	0.077	0.965
SlCHX-10	− 1.95	0.48 ± 0.15	13.3 ± 4.1	7yxxA	0.894	2.52	0.083	0.931
SlSOS-2	0.50	0.78 ± 0.10	7.0 ± 4.1	7y3eA	0.932	0.65	0.433	0.937
SlSOS-3	− 0.27	0.68 ± 0.12	8.0 ± 4.4	7x2uA	0.905	1.49	0.229	0.929
SlSOS-5	0.05	0.72 ± 0.11	7.5 ± 4.3	7x2uA	0.889	1.58	0.231	0.906
SlRLK-39	− 0.95	0.59 ± 0.14	10.5 ± 4.6	6xr4A	0.891	1.81	0.148	0.912
SlRLK-42	− 3.01	0.37 ± 0.13	17.4 ± 2.6	6xr4A	0.682	1.74	0.136	0.693
SlRLK-47	− 1.39	0.54 ± 0.15	12.2 ± 4.4	7lhtA	0.868	2.97	0.196	0.912

### Prediction of miRNAs targeting the *CHX*, *SOS*, and *RLK* genes in *S. lycopersicum*

Using a cut-off threshold of 5 for the search parameters, 91 microRNAs were predicted to target the genes in 20 *CHX*, 40 microRNAs were predicted to target the proteins in 5 *SOS*, and 202 microRNAs were predicted to target the genes in 83 *RLK*. To reduce the number of false positive predictions, we included microRNA/target site pairs with an expectation score and a cut-off threshold of less than 5 to 4. This resulted in 45 microRNAs predicted to target the genes in 18 *CHX*, 9 microRNAs predicted to target the proteins in 3 *SOS*, and 152 microRNAs predicted to target the genes in 76 *RLK*. Additionally, this resulted in 3 microRNAs predicted to target the genes in 3 *CHX*, 1 microRNAs predicted to target the proteins in 1 *SOS*, and 51 microRNAs were predicted to target the genes in 28 *RLK* and had an expectation score of equal and less than 3.5, they can be considered more reliable. The microRNA targeting relationships for the *CHX*, *SOS*, and *RLK* genes are presented in Table S11 of Online Resource SI 1.

The results from the prediction of RNA secondary structures with pseudoknots for the *CHX* (*SlCHX-3*, *SlCHX-5*, and *SlCHX-10*), *SOS* (*SlSOS-2*, *SlSOS-3*, and *SlSOS-5*), and *RLK* (*SlRLK-1*, *SlRLK-2*, and *SlRLK-3*) proteins are shown in Fig. S95: Fig. S103 Online Resource SI 2.

### Gene Ontology enrichment and functional relationship analysis of the *CHX*,* SOS*, and *RLK* genes in *S. lycopersicum*

To better define the roles of the *CHX*, *SOS*, and *RLK* genes, we conducted gene ontology (GO) analysis and enrichment analysis focused on their molecular functions and biological processes. GO terms allow us to determine the functions of these genes at the molecular level (Fig. S104, Fig. S105, and Fig. S106 Online Resource SI 2). Gene Ontology enrichment analysis identified *CHX*, *SOS*, and *RLK* as stress-responsive genes (Fig. S107, Fig. S108, and Fig. S109 Online Resource SI 2).

In this study, qRT-PCR analysis demonstrated that the CHX, SOS, and RLK proteins were expressed in the leaves. It was found that salinity increased the expression levels of the *CHX*, *SOS* and *RLK* genes by 1.83, 1.49, and 1.55 times, respectively, after 12 h of salinity stress (Table S1 in Online Resource SI 1). Additionally, analyses of the domain structure, promoter regions, and gene ontology enrichment confirmed the functional roles of the CHX, SOS, and RLK proteins in stress responses.

## Discussion

The *CHX*^52^, *SOS*^53^, and *RLK*^54^;^55^ genes play important roles in the response to salt stress. To date, no comprehensive investigation of the *SOS* gene in *S. lycopersicum* under salt stress has been reported via genome-wide analysis. In this study, the genome-wide analysis revealed 21 *CHX*, 5 *SOS*, and 86 RLK genes in *S. lycopersicum*. Eighty-six SlRLK proteins were classified by domain to 1 SlRLK protein containing LRR with csk-1, STK, and Laminin_G_3. 21 SlRLK proteins contain LRR, 12 SlRLK proteins contain LRR with csk-1, 6 SlRLK proteins contain LRR with STK. 5 SlRLK proteins contain PERK, 12 SlRLK proteins contain csk-1, 2 SlRLK proteins contain csk-1 with G-type LecRLKs, 1 SlRLK protein contains csk-1 with stress-antifung, 3 SlRLK proteins contain STK, 5 SlRLK proteins contains L-type LecRLKs, 4 SlRLK proteins contains RLK902, 2 SlRLK proteins contains BRl1, 1 SlRLK proteins contains ATP binding, 2 SlRLK proteins contains IRK, 1 SlRLK proteins contain G-type LecRLK and 8 SlRLK proteins contains Pkinase. Hussain et al.^52^ identified 18 *CHX* genes in *S. lycopersicum*, and Wei et al.^56^ identified 234 from subfamily LRR-RLKs in the genome of *S. lycopersicum* ‘Heinz 1706’. Our qRT‒PCR results indicated that CHX, SOS, and *RLK* were upregulated in leaves, with fold changes of 1.83, 1.49, and 1.55, respectively, after 12 h of salt stress. Hussain et al.^52^ reported that *CHX* was upregulated after salinity stress, and Ali et al.^57^ reported that the *SlSOS1* gene was upregulated in the leaves of both the TA1648 and IL 2–3 genotypes under NaCl stress. In the present study, domain analysis confirmed the presence of the Na_H_Exchanger superfamily domain^52^, the Na_H_Exchanger superfamily domain, and the PKc_like superfamily domain in the CHX, SOS, and RLK proteins, respectively. Motif analyses indicated that genes with closer phylogenetic relationships exhibited more similar genetic structures. The gene structure results revealed that all 21 *CHX* and 5 *SOS* genes had introns, while *RLK* genes had introns, while *SlRLK-76*, *SlRLK-63*, *SlRLK-77*, *SlRLK-70*, *SlRLK-33*, *SlRLK-64*, *SlRLK-14*, and *SlRLK-8* did not have introns. The promoter regions of the *CHX*, *SOS*, and *RLK* genes contain defense and stress response elements, abscisic acid-responsive elements, methyl jasmonate (MeJA)-responsive elements, salylic acid, and the MYB binding site (MBS) element, which are involved in the salinity response. *CHX* genes were found on chromosomes 2, 3, 4, 5, 6, 7, 8, 9, 11 and 12 of *S. lycopersicum*. *SOS* genes were found on chromosomes 1, 4, 6, and 10. *RLK* genes were found on all chromosomes of *S. lycopersicum*. Purifying selection results in decreased genetic diversity^50^, leading to reduced species diversity and diminished evolutionary changes between species compared to neutral sites^51^. The duplication time of the *CHX* paralogous gene pairs ranged from approximately 26.965 to 245.413 Mya. The duplication time of the *SOS* paralogous gene pair ranged from approximately 116.682 to 275.631 Mya. The duplication time of the *RLK* paralogous gene pairs ranged from approximately 27.689 to 239.376 Mya. Synteny analysis of the *CHX*, *SOS*, and *RLK* genes revealed collinearity orthologous relationships in *A. thaliana* but no collinearity orthologous relationships in *O. sativa*. In addition, collinearity analysis revealed that 6 orthologous *SlCHX* genes, 2 orthologous *SlSOS* genes, and 44 orthologous *SlRLK* genes were paired with those in *A. thaliana*. Among the 21 CHX proteins^52^, 5 SOS proteins were located in the plasma membrane, whereas 86 RLK proteins were located in the plasma membrane; SlRLK-9, SlRLK-24, SlRLK-39, and SlRLK-61 proteins were located in the chloroplasts, and SlRLK-18, SlRLK-78, and SlRLK-69 proteins were located in the cytoplasm. Four putative nuclear localization signals (NLSs) were predicted for 5 SlCHX proteins, 13 putative nuclear localization signals (NLSs) were predicted for 13 SlRLK proteins, and no nuclear localization signals (NLSs) were predicted for SlSOS proteins. A total of 113 microRNAs were predicted to target the 21 *CHX* genes, 47 microRNAs were predicted to target 5 *SOS* genes, and 250 microRNAs were predicted to target 86 *RLK* genes. Gene Ontology enrichment analysis confirmed the functional role of *CHX*, *SOS*, and *RLK* in stress response.

## Conclusion

This study is the first to identify the *SOS* gene in *S. lycopersicum* through comprehensive genome-wide analysis. Our investigations into domain structure, promoter elements, and gene ontology enrichment have decisively confirmed the critical roles of CHX, SOS, and RLK proteins in mediating stress responses. The insights gained from this research are poised to enhance our understanding of how the *CHX*, *SOS*, and *RLK* genes can effectively alleviate salt stress in *S. lycopersicum*, paving the way for innovative strategies in the genetic improvement of this essential crop.

## Electronic Supplementary Material

Below is the link to the electronic supplementary material.

Supplementary Material 1

Supplementary Material 2

## Data Availability

Availability of data and materialsThe datasets supporting the conclusions of this article are included within the article and its additional files.

## Abbreviations

CHX: Cation/proton exchanger
SOS: Salt overly sensitive
RLK: Receptor-like kinase
Ka/Ks: Ratio of nonsynonymous/synonymous
RMSD: Root mean square deviation
