# Correction: Genome-wide identification, characterization, and functional analysis of the *CHX*, *SOS*, and *RLK* genes in *Solanum lycopersicum* under salt stress

**DOI:** 10.1038/s41598-026-36169-y

**Published:** 2026-01-15

**Authors:** Amaal Maghraby, Mohamed Alzalaty

**Affiliations:** 1 Botany and Microbiology Department, Faculty of Science, Cairo University, Cairo, Egypt; 2 Department of Plant Genetic Transformation, Agricultural Genetic Engineering Research Institute (AGERI), Agricultural Research Center (ARC), Cairo, Egypt

Correction to: *Scientific Reports* 10.1038/s41598-024-83221-w, published online 07 January 2025

The original version of this Article contained an error in the Methods section, under the subheading ‘Phylogenetic, chromosomal distribution, evolutionary analysis, and synteny analysis of the *CHX*, *SOS*, and *RLK* genes in *S. lycopersicum*’.

“The formula employed for this estimation is as follows: ‘${\mathrm{T}}={\mathrm{Ks}}/{2}\lambda \times {1}0{}^{-{6}} \, (\lambda ={6}.\text{5 }\times \text{ 1}{0}^{-{9}})$’^27^”.

now reads:

“The formula employed for this estimation is as follows: ‘${\mathrm{T}}={\mathrm{Ks}}/{2}\lambda \times {1}0{}^{-{6}} \, (\lambda ={6}.\text{56 }\times \text{ 1}{0}^{-{9}})$’^27^”.

In addition, Reference 27, “Yuan, S. et al. Comprehensive analysis of CCCH-type zinc finger family genes facilitates functional gene discovery and reflects recent allopolyploidization event in tetraploid switchgrass. *BMC Genom.* **16**, 1–16 (2015)” was incorrectly cited in the Article and listed in the Reference list. This reference has now been removed and replaced with the correct citation of Reference 27: “He, Y. et al. Genome-Wide Identification and Expression Analysis of Two-Component System Genes in Tomato. *Int. J. Mol. Sci.* **2016**, *17*, 1204. 10.3390/ijms17081204”.

The original Article has been corrected.

