# Supplementary material for: Genome-wide identification, characterization, and functional analysis of the CHX, SOS, and RLK genes in Solanum lycopersicum under salt stress
Source: Sci Rep. 2025 Jan 7;15:1142. doi: 10.1038/s41598-024-83221-w (PMC11707246; doi:10.1038/s41598-024-83221-w)
Supplement: Supplementary file 2 — Supplementary Material 2 [file 41598_2024_83221_MOESM2_ESM.pdf]

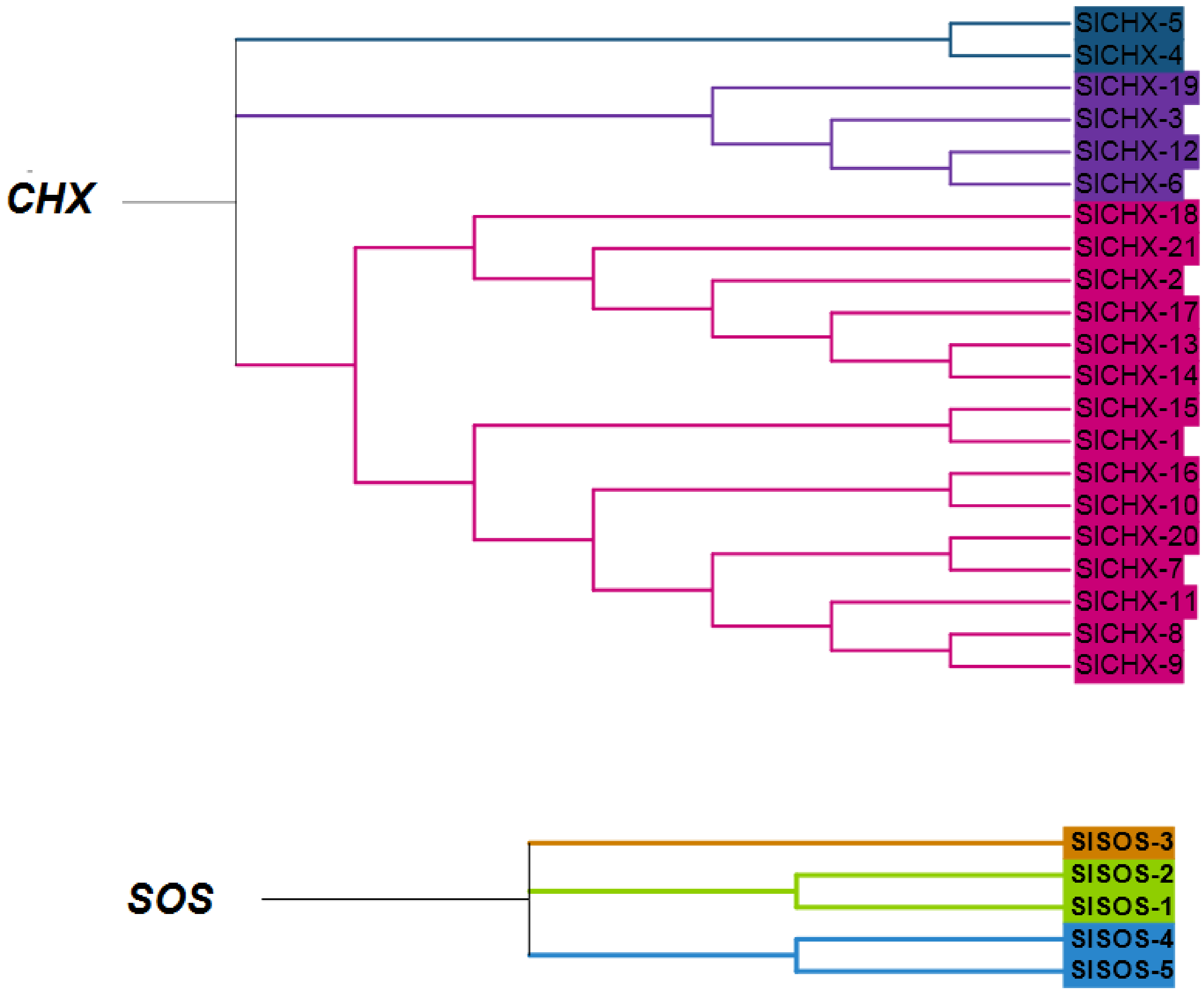

Fig.S1. Maximum likelihood phylogenetic tree of the SlCHX and SlSOS protein family in S. lycopersicum.

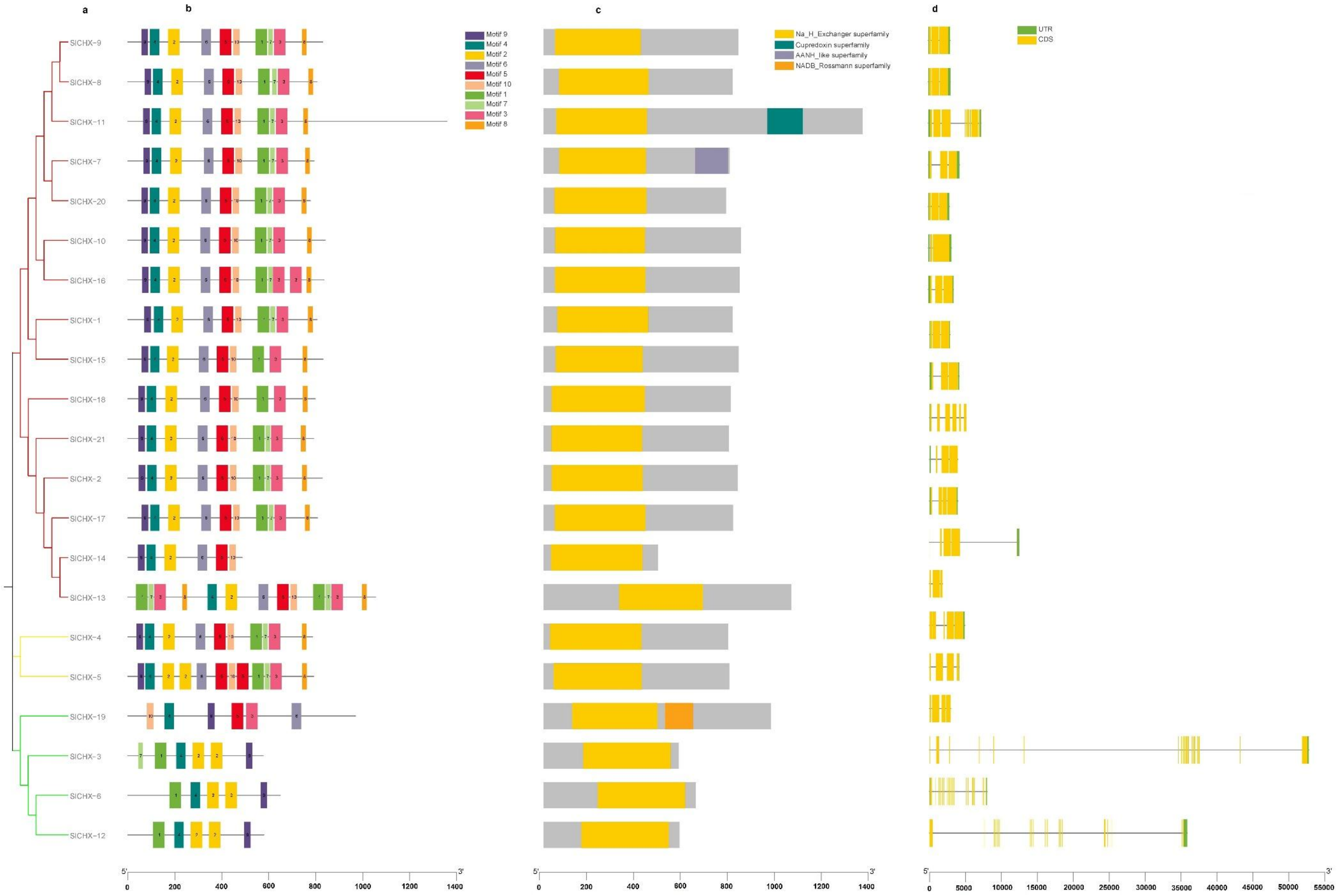

Fig. S2. CHX proteins (a) the rectangular phylogenetic tree. (b) Conserved motifs were predicted using MEME. (c) Protein domains. (d) Gene structure.

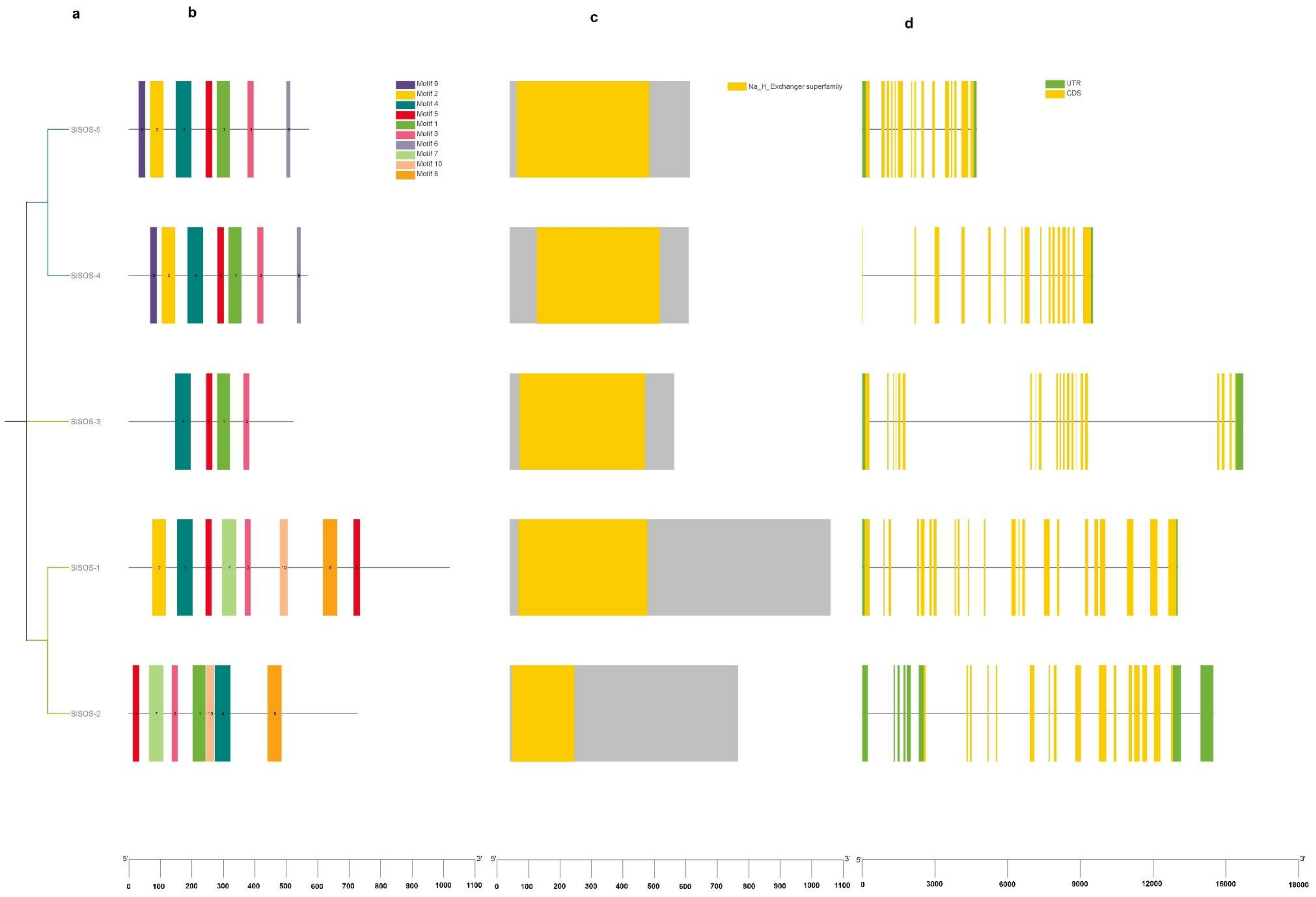

Fig. S3. SOS proteins (a) the rectangular phylogenetic tree. (b) Conserved motifs were predicted using MEME. (c) Protein domains. (d) Gene structure.

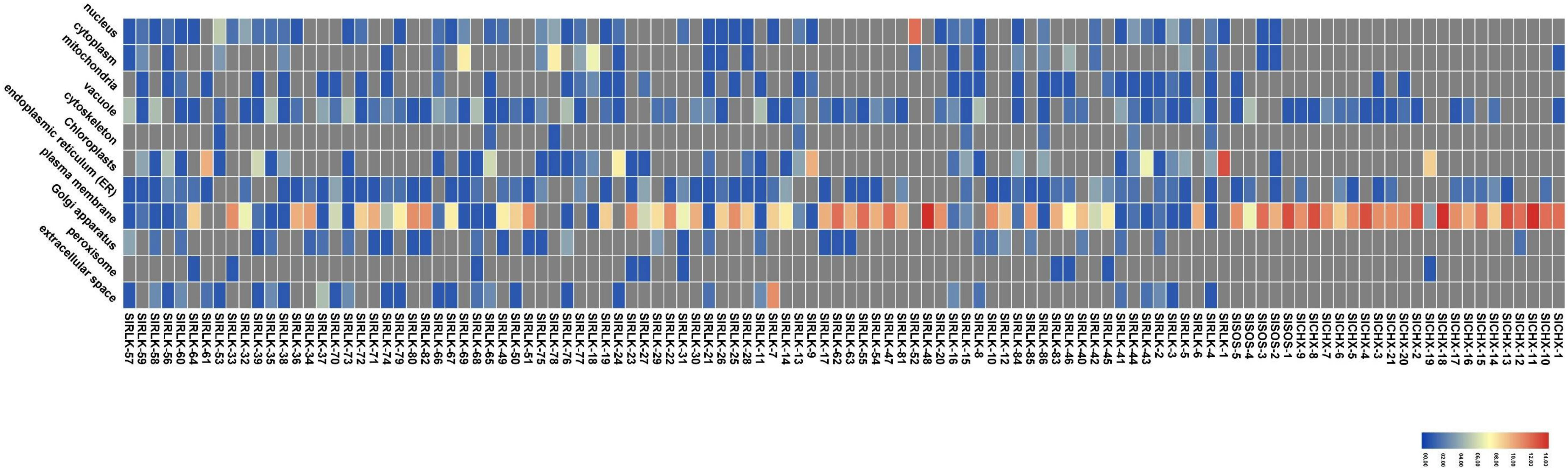

Fig. S4. Heatmap showing 21 CHX, 5 SOS and 86 RLK proteins localization prediction in deferent organelle

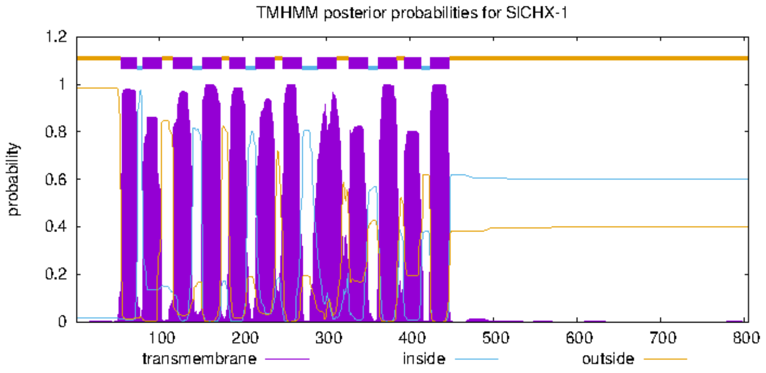

SlCHX-1

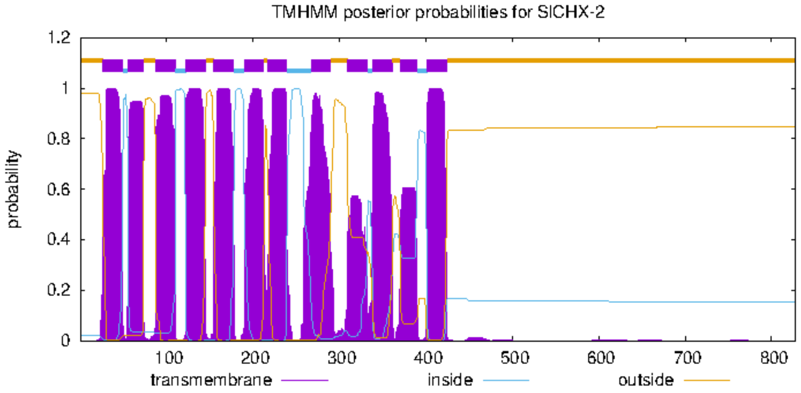

SlCHX-2

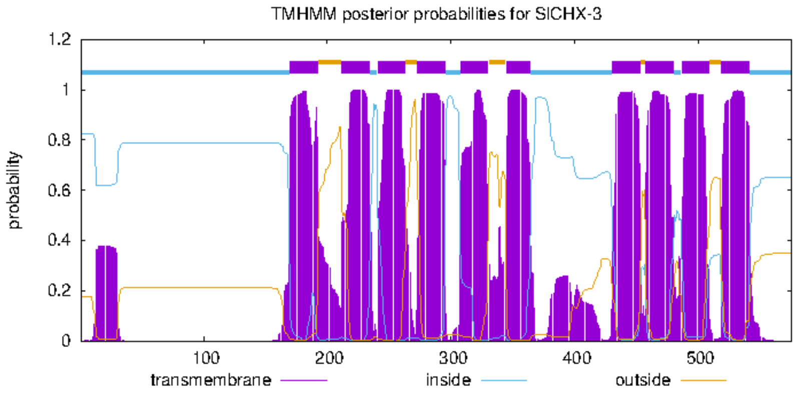

SlCHX-3

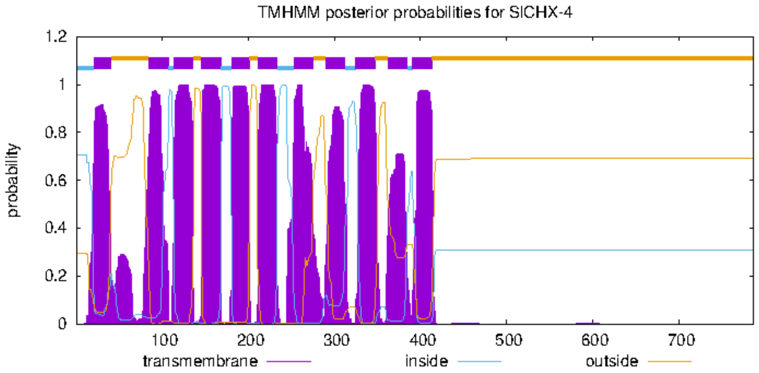

SlCHX-4

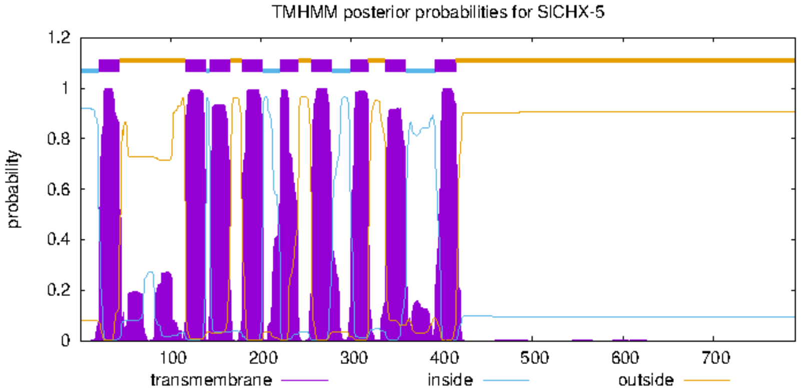

SlCHX-5

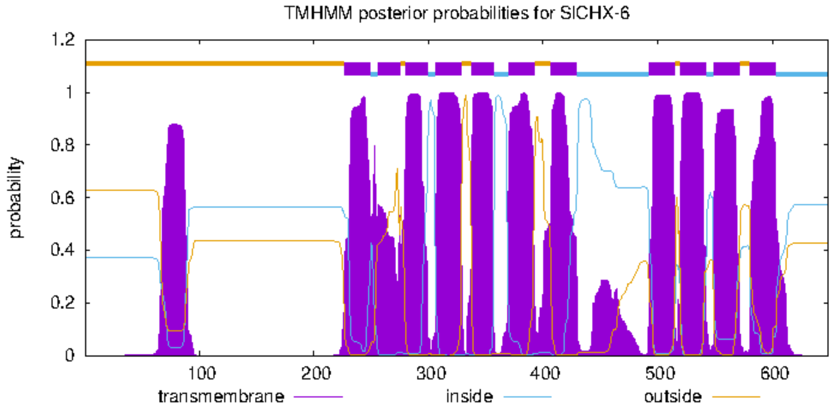

SlCHX-6

Fig. S5. The TMHMM results predicted the transmembrane helices

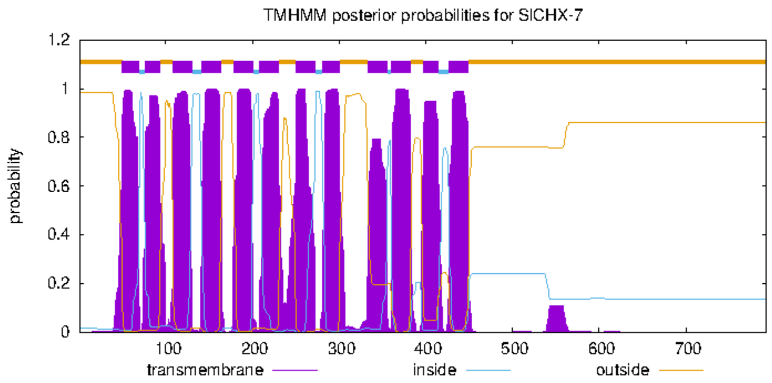

SlCHX-7

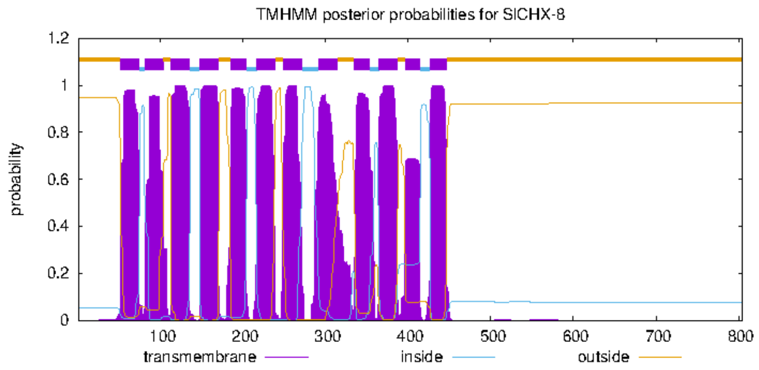

SlCHX-8

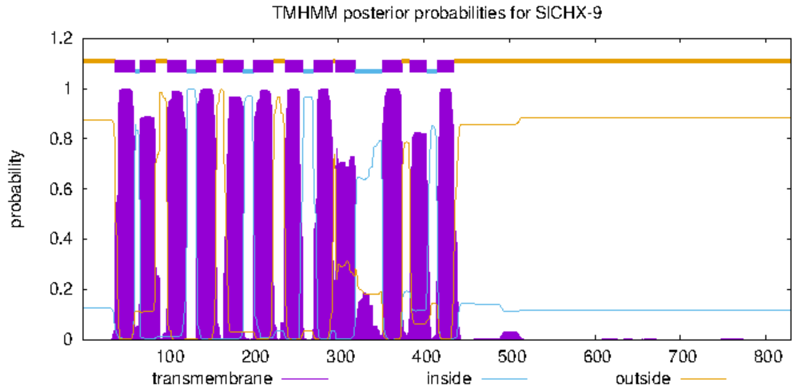

SlCHX-9

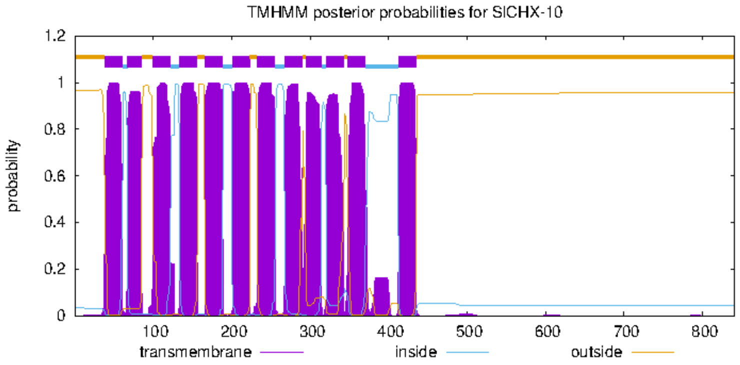

SlCHX-10

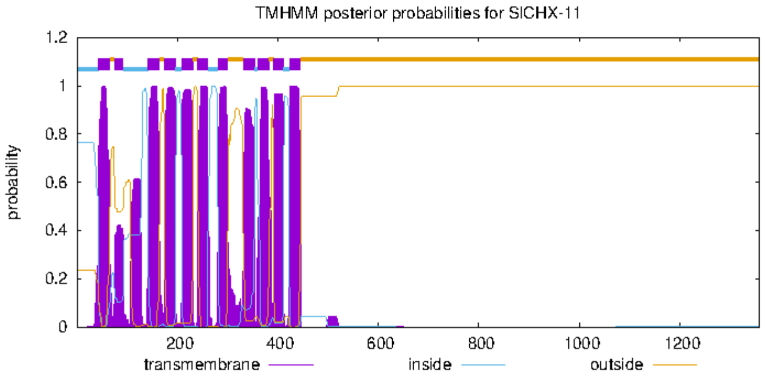

SlCHX-11

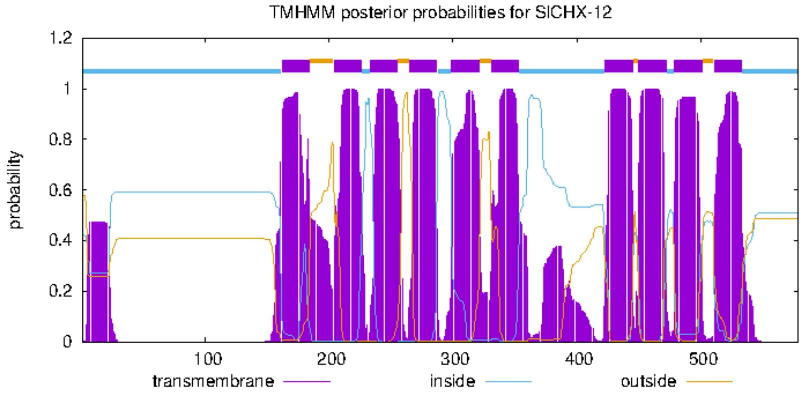

SlCHX-12

Fig. S6. The TMHMM results predicted the transmembrane helices

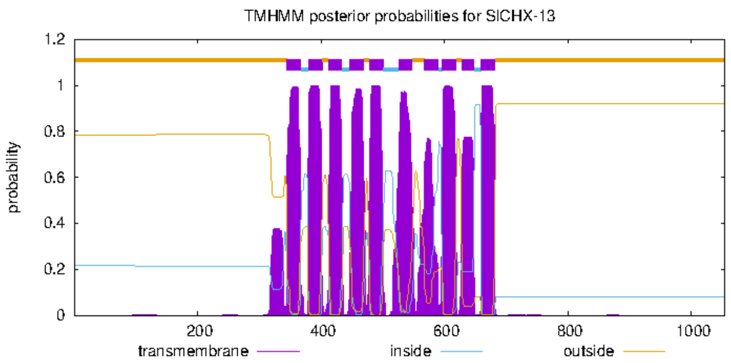

SlCHX-13

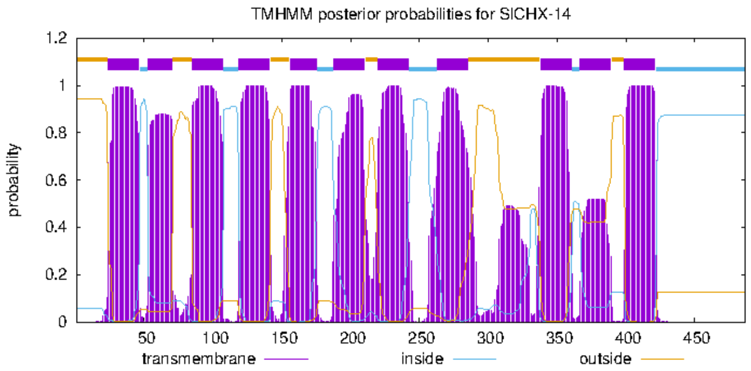

SlCHX-14

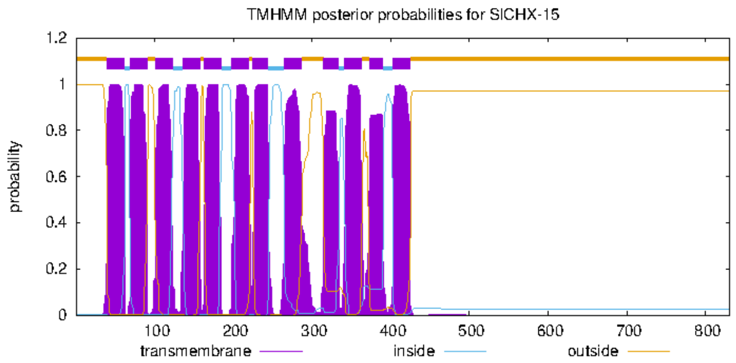

SlCHX-15

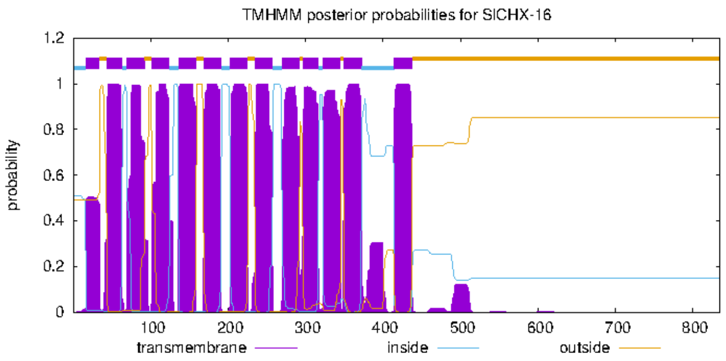

SlCHX-16

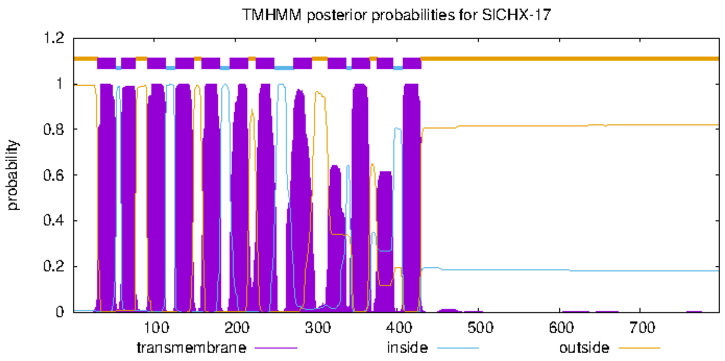

SlCHX-17

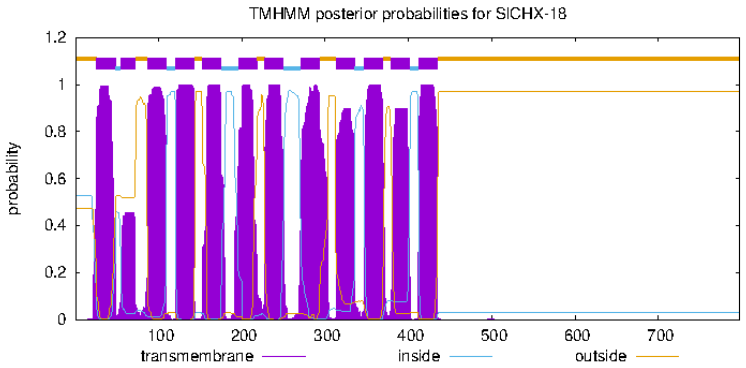

SlCHX-18

Fig. S7. The TMHMM results predicted the transmembrane helices

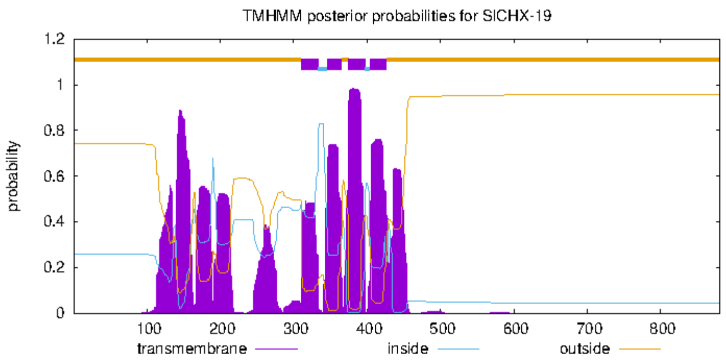

SlCHX-19

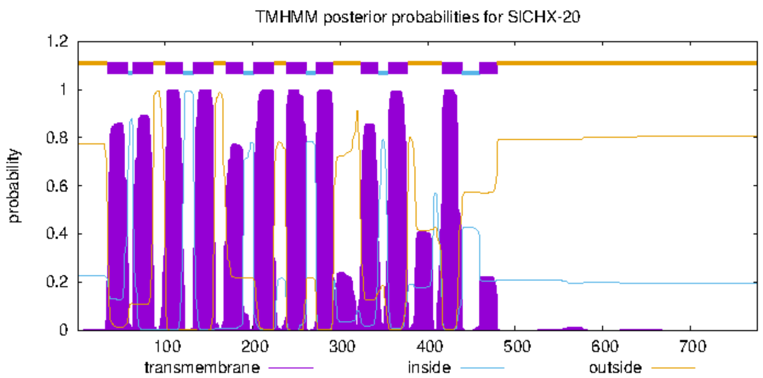

SlCHX-20

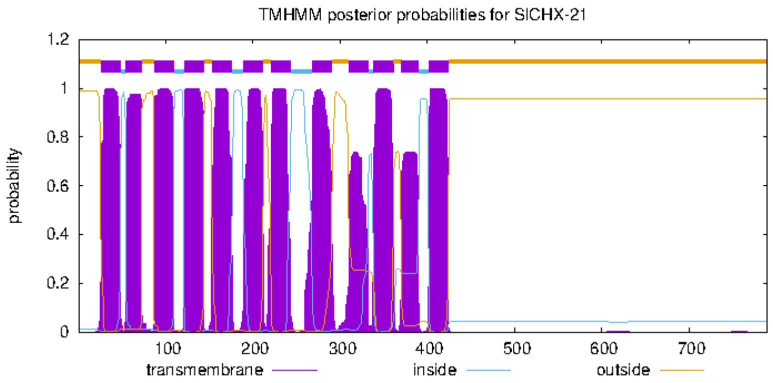

SlCHX-21

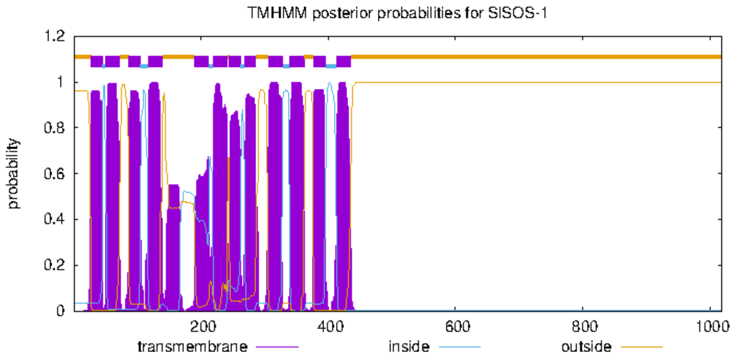

SlSOS-1

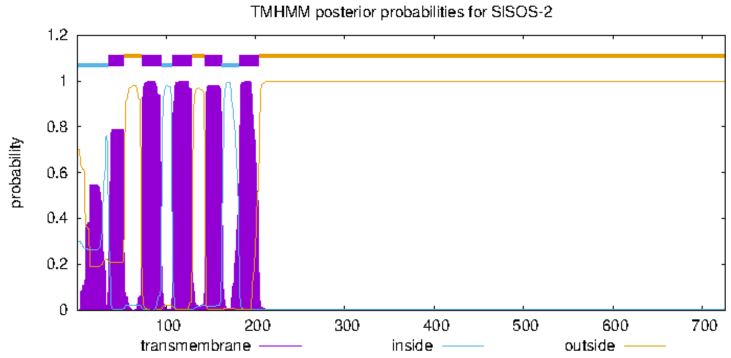

SlSOS-2

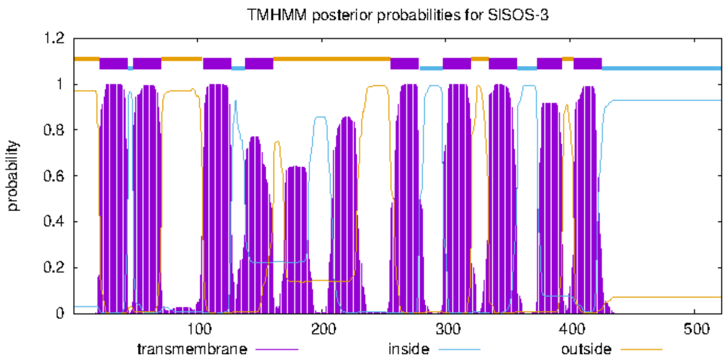

SlSOS-3

Fig. S8. The TMHMM results predicted the transmembrane helices

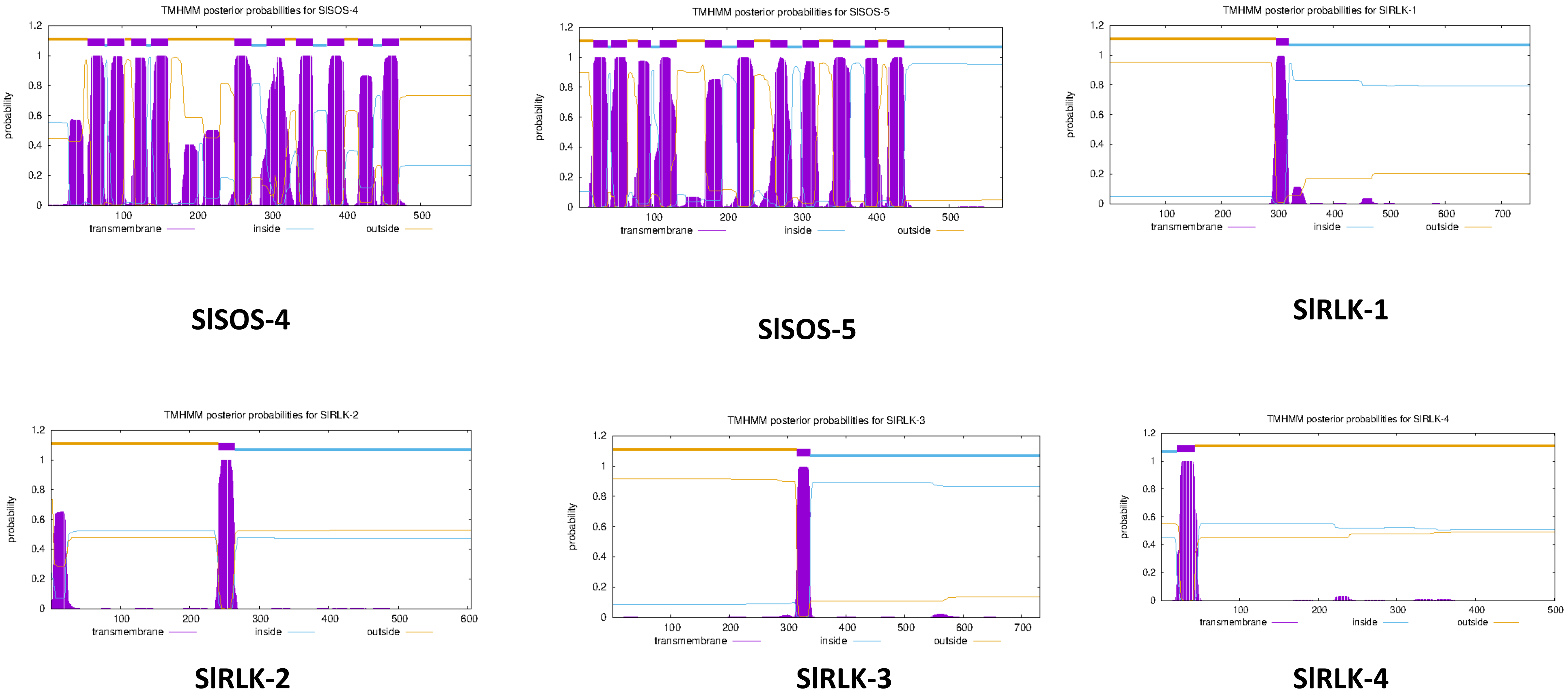

Fig. S9. The TMHMM results predicted the transmembrane helices

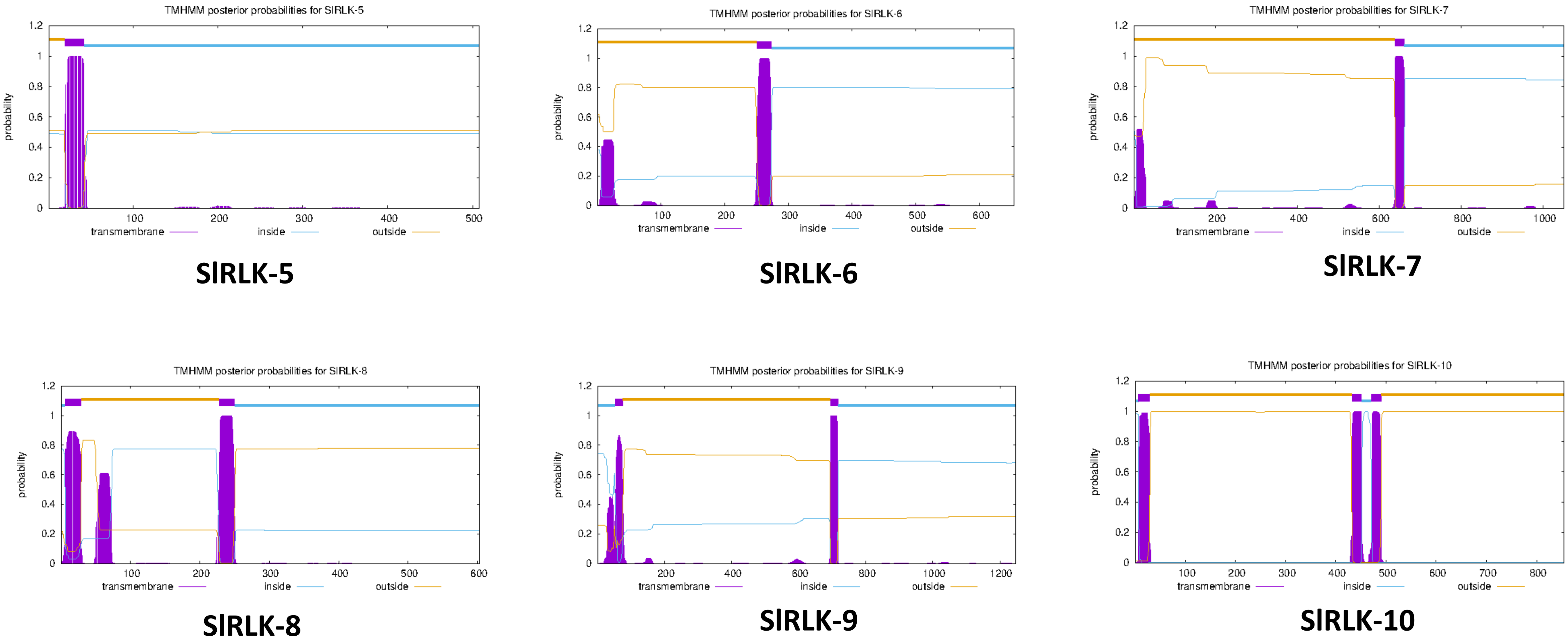

Fig. S10. The TMHMM results predicted the transmembrane helices

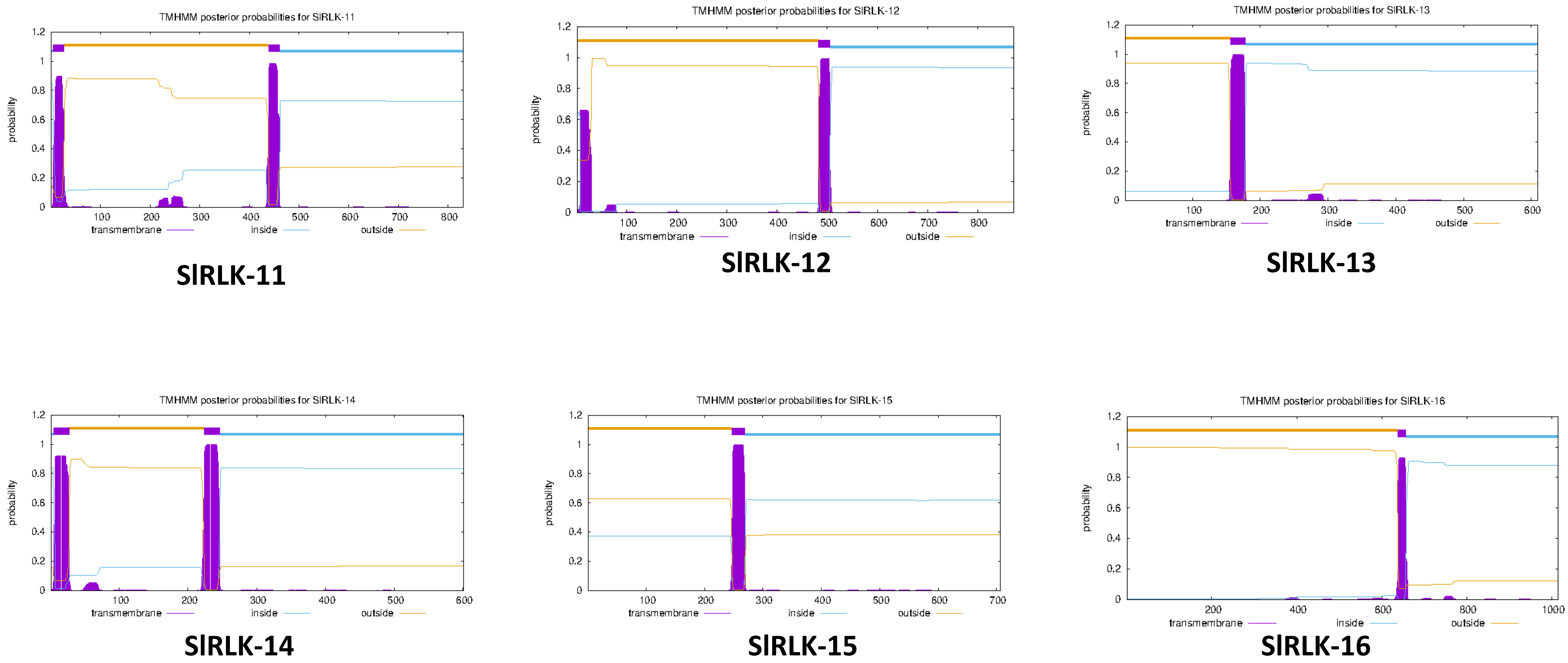

Fig. S11. The TMHMM results predicted the transmembrane helices

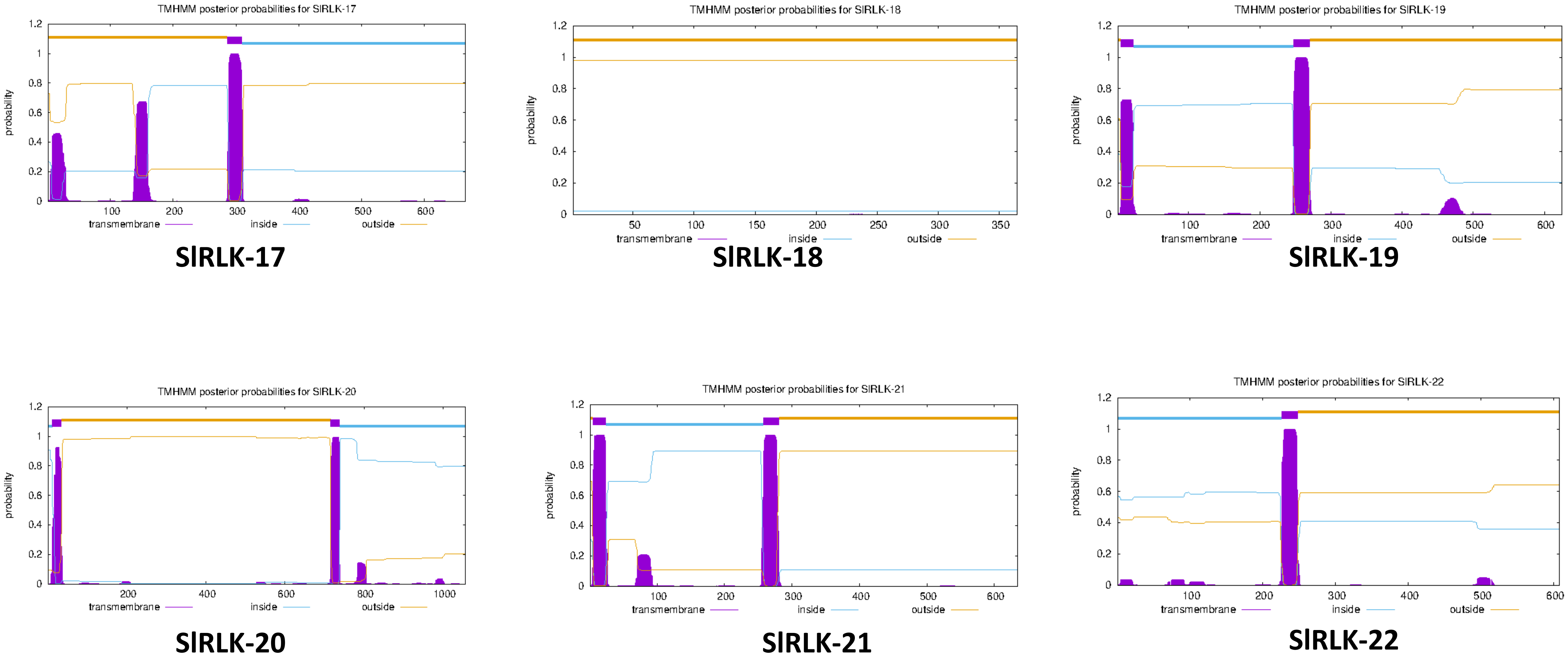

Fig. S12. The TMHMM results predicted the transmembrane helices

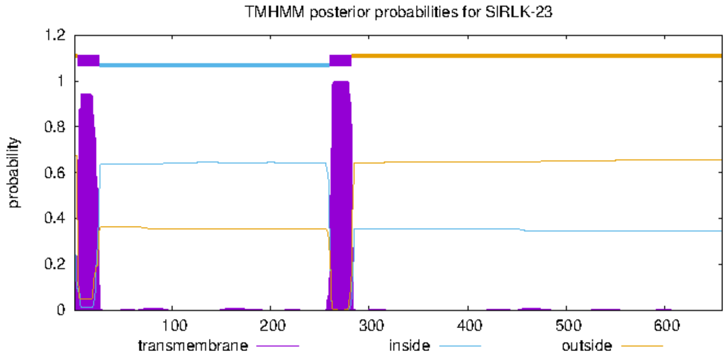

SlRLK-23

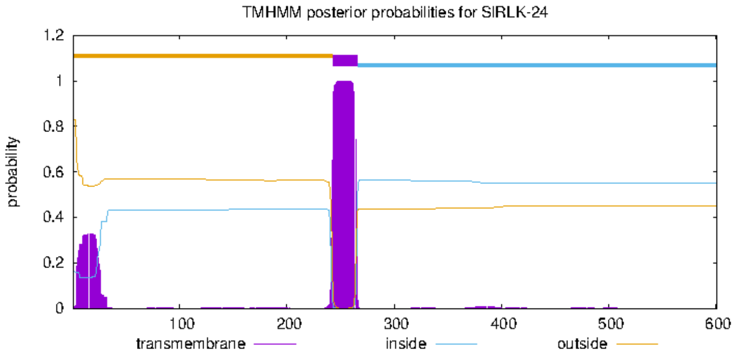

SlRLK-24

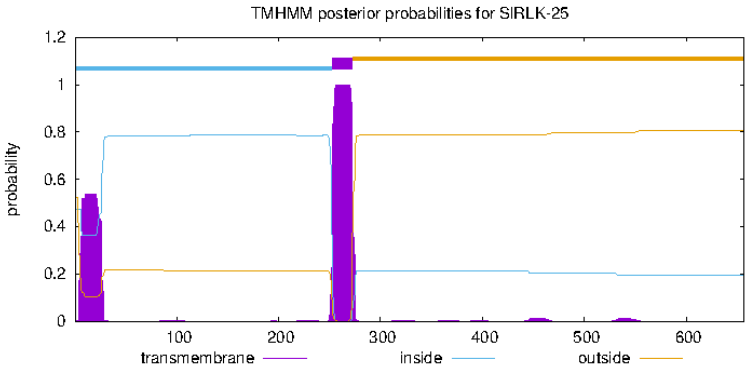

SlRLK-25

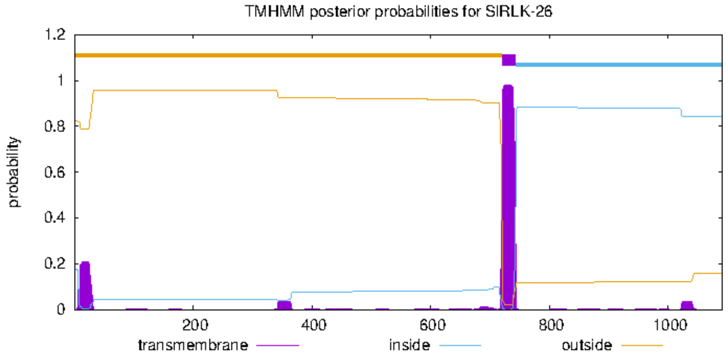

SlRLK-26

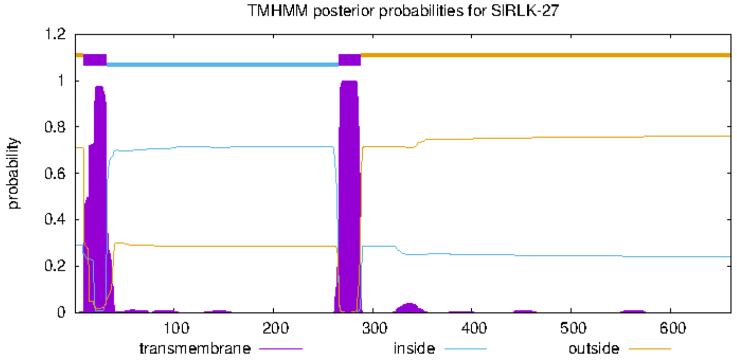

SlRLK-27

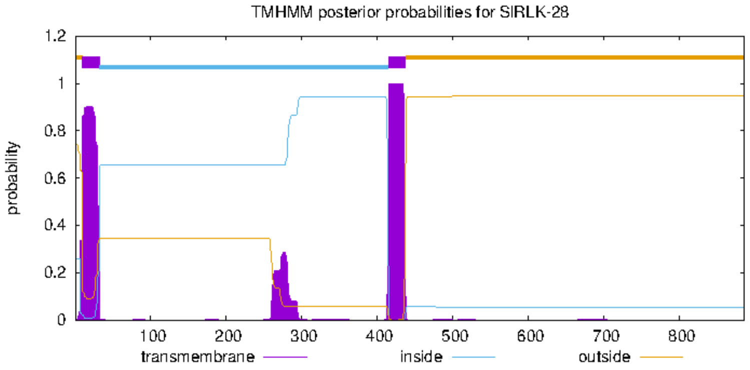

SlRLK-28

Fig. S13. The TMHMM results predicted the transmembrane helices

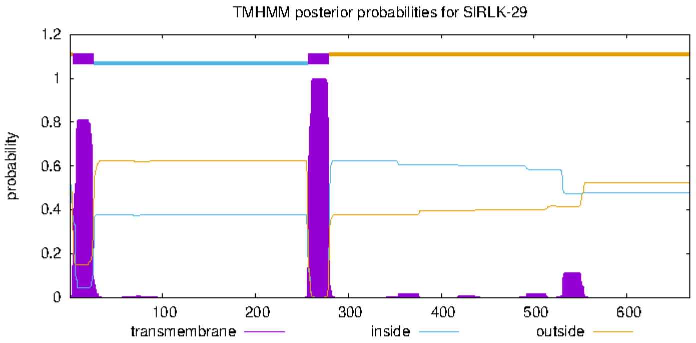

SlRLK-29

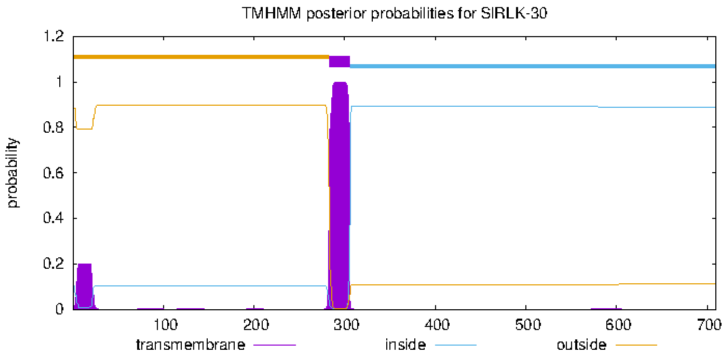

SlRLK-30

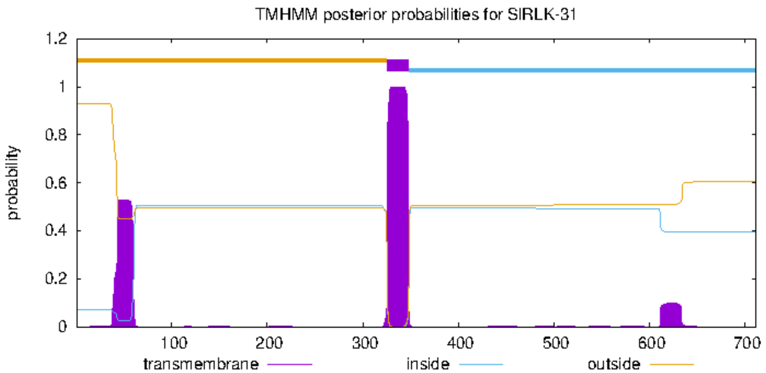

SlRLK-31

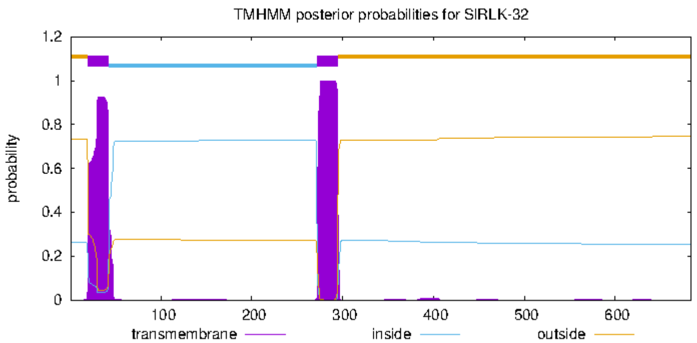

SlRLK-32

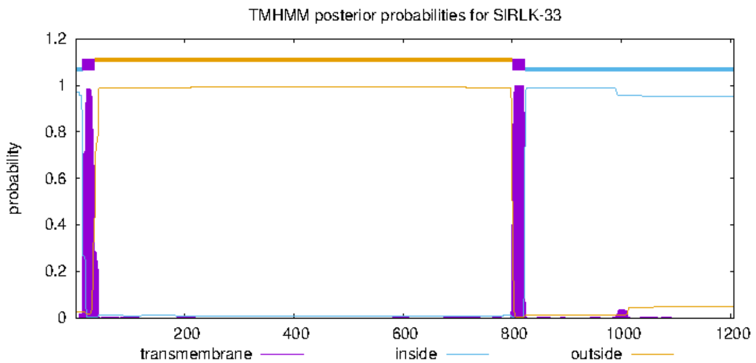

SlRLK-33

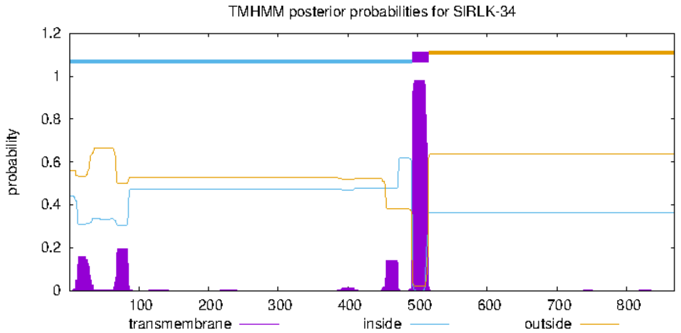

SlRLK-34

Fig. S14. The TMHMM results predicted the transmembrane helices

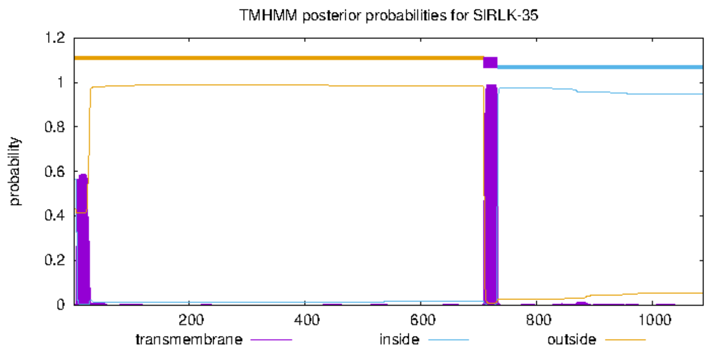

SlRLK-35

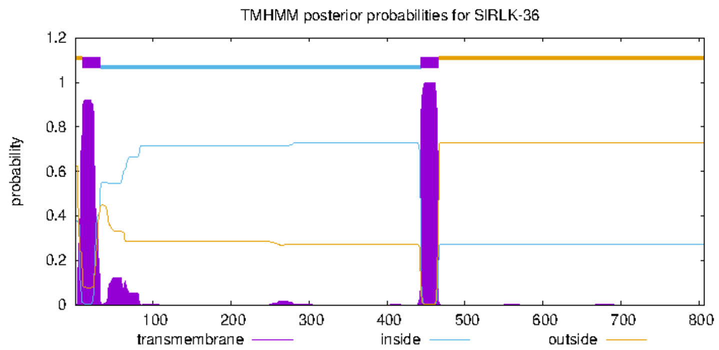

SlRLK-36

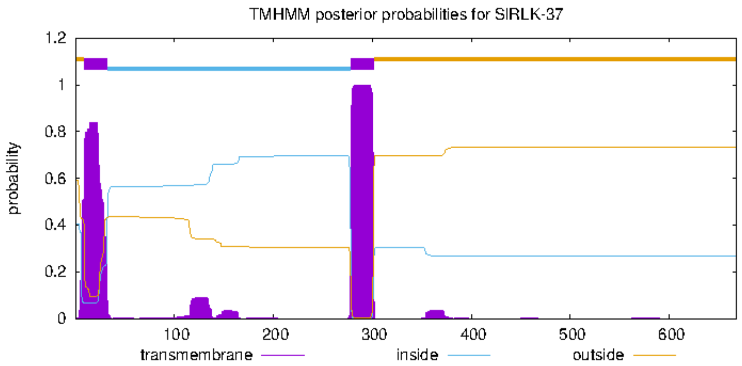

SlRLK-37

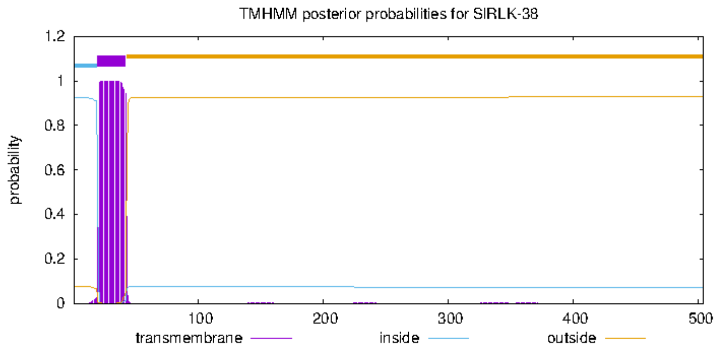

SlRLK-38

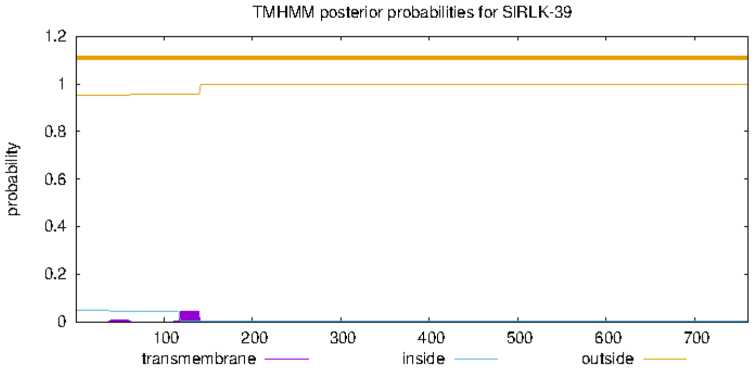

SlRLK-39

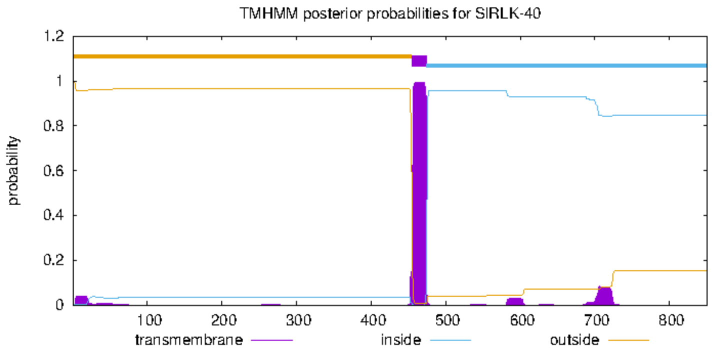

SlRLK-40

Fig. S15. The TMHMM results predicted the transmembrane helices

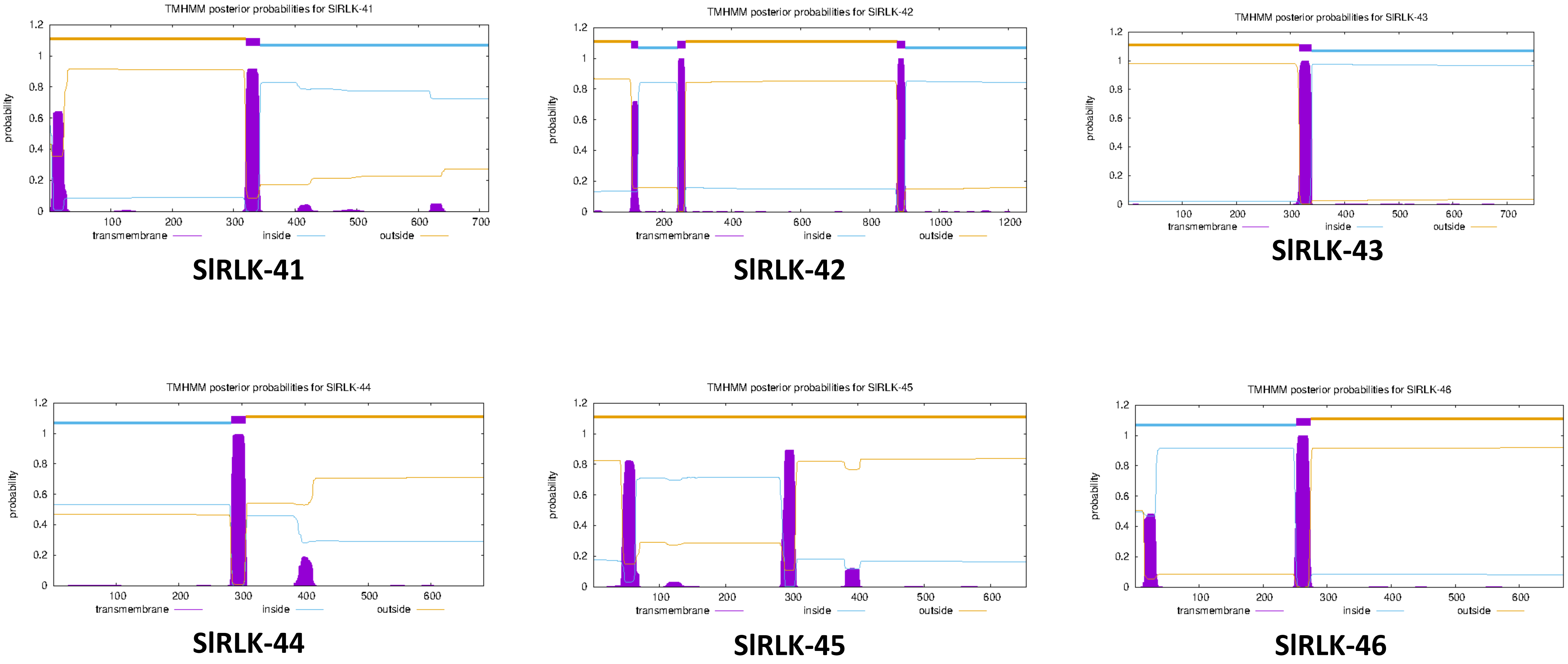

Fig. S16. The TMHMM results predicted the transmembrane helices

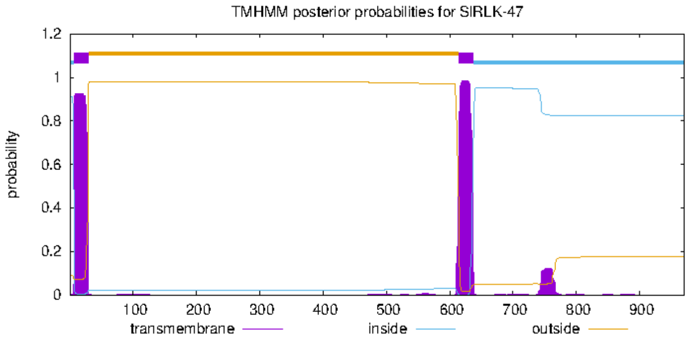

SlRLK-47

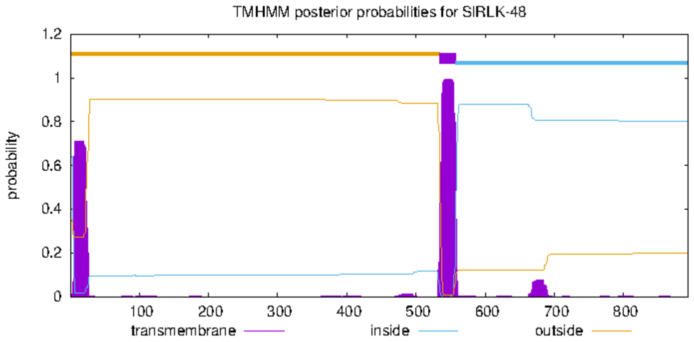

SlRLK-48

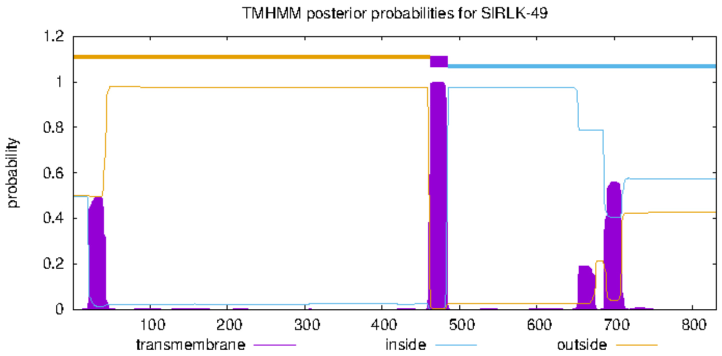

SlRLK-49

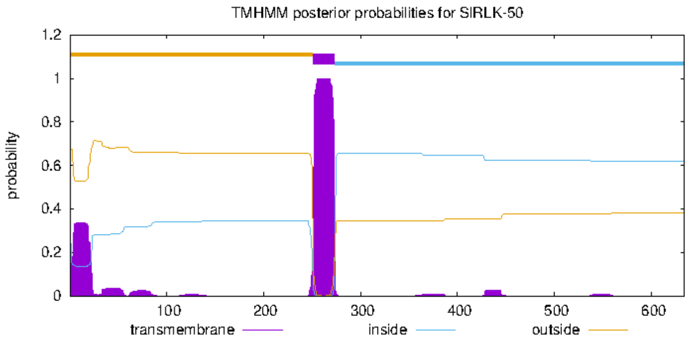

SlRLK-50

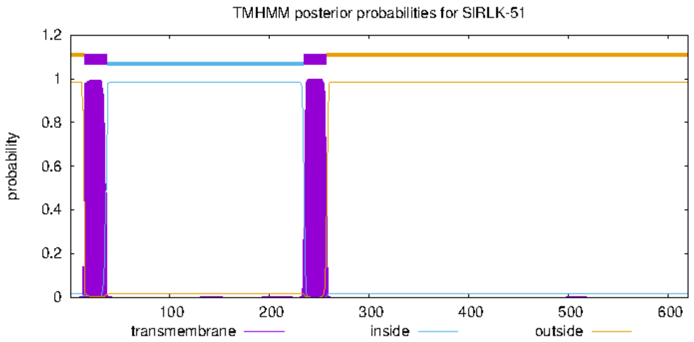

SlRLK-51

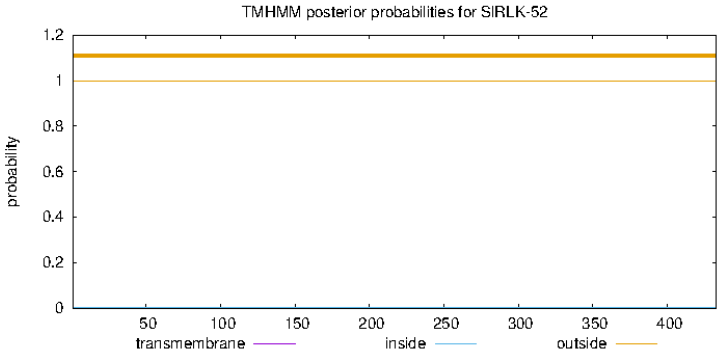

SlRLK-52

Fig. S17. The TMHMM results predicted the transmembrane helices

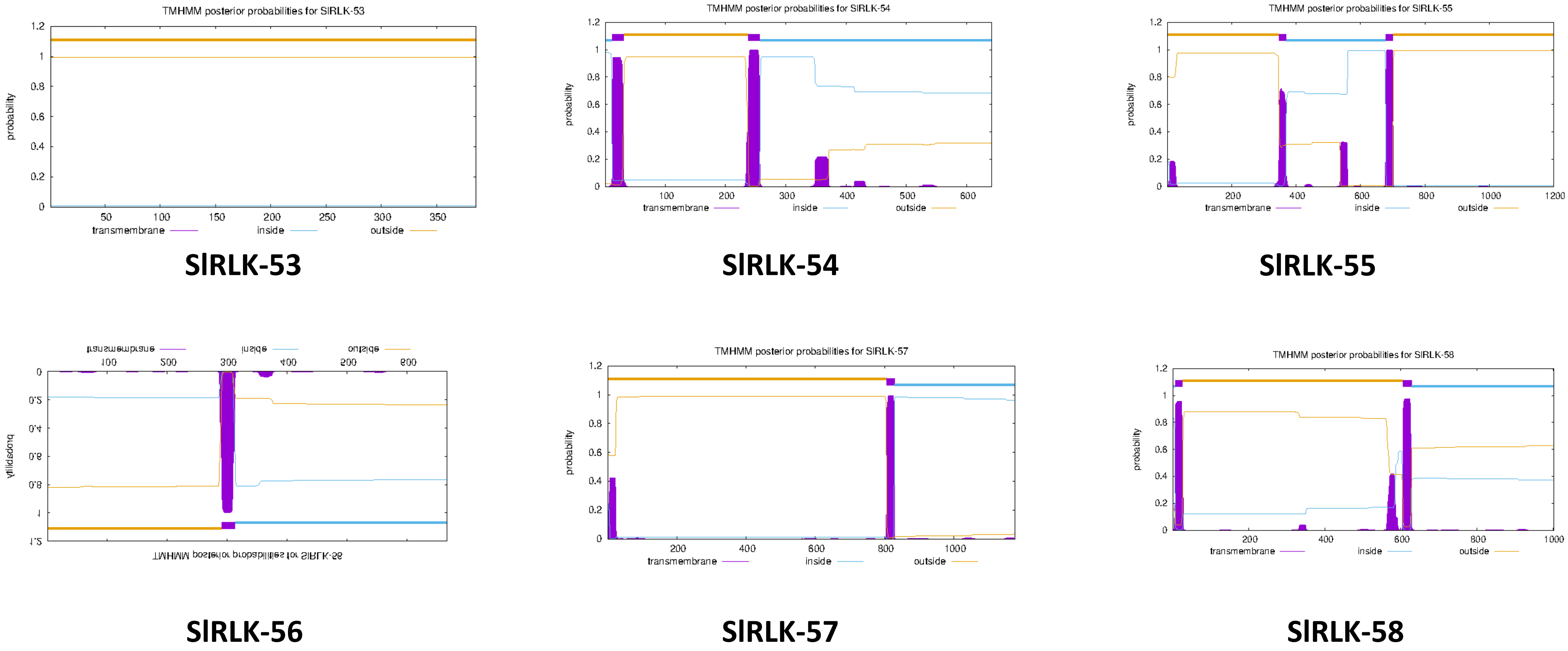

Fig. S18. The TMHMM results predicted the transmembrane helices

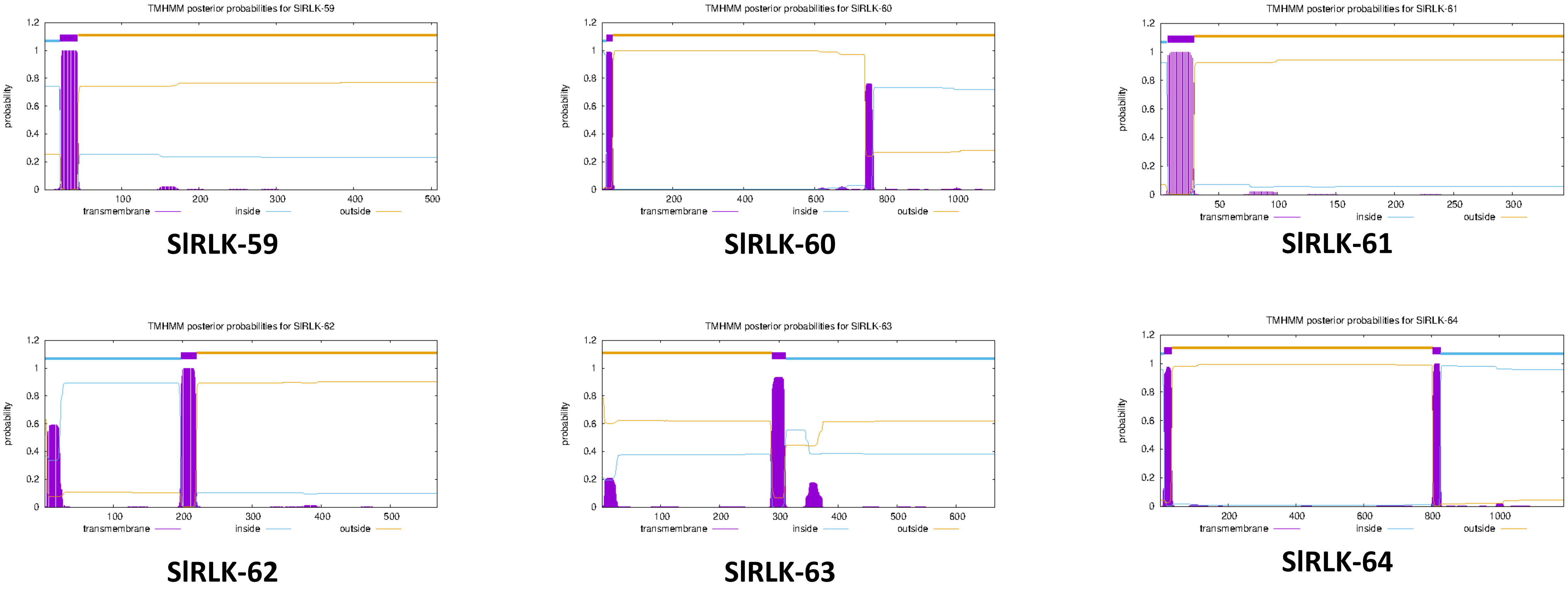

Fig. S19. The TMHMM results predicted the transmembrane helices

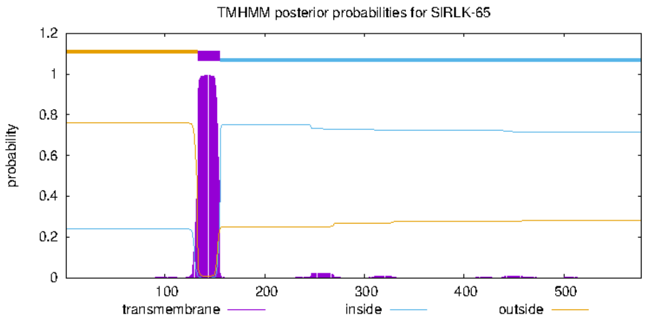

SlRLK-65

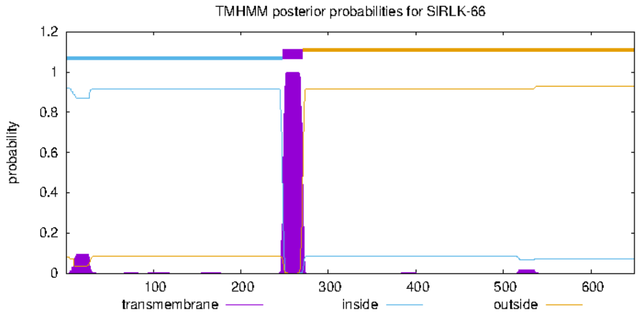

SlRLK-66

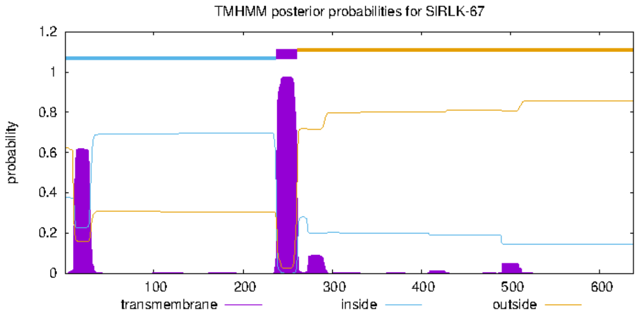

SlRLK-67

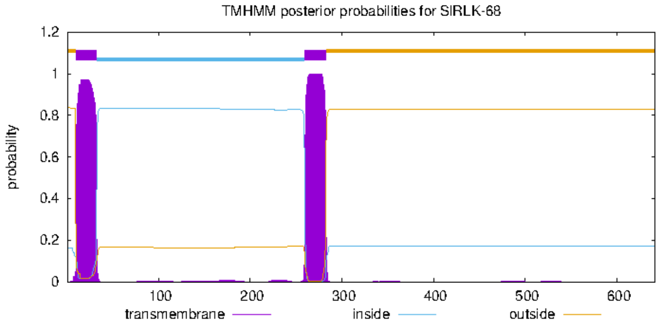

SlRLK-68

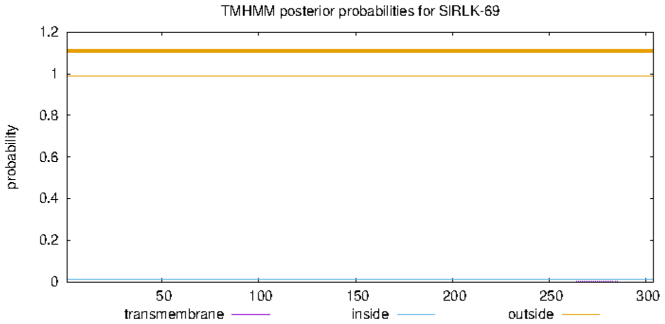

SlRLK-69

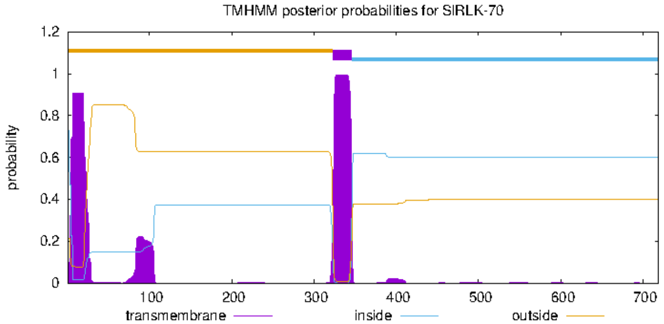

SlRLK-70

Fig. S20. The TMHMM results predicted the transmembrane helices

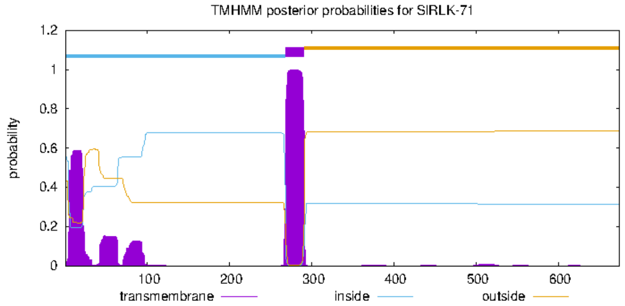

SlRLK-71

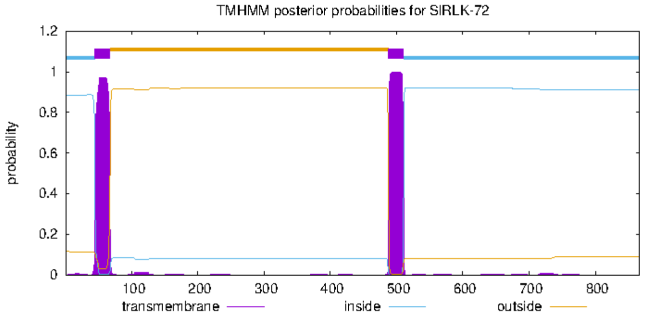

SlRLK-72

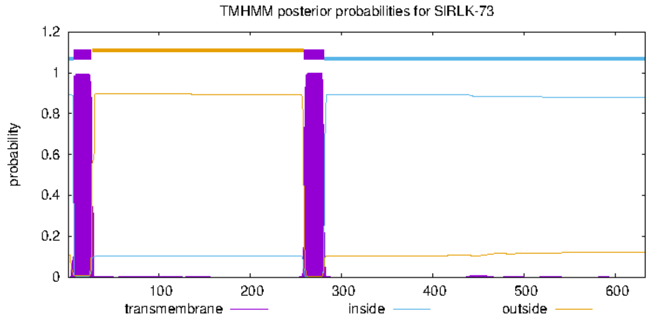

SlRLK-73

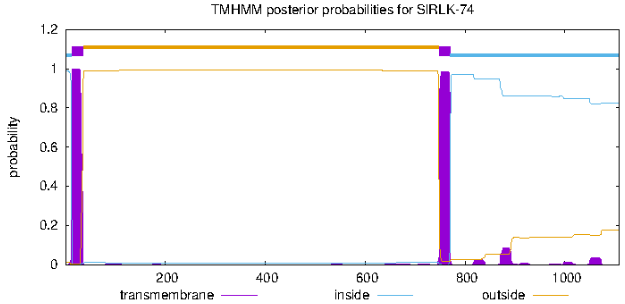

SlRLK-74

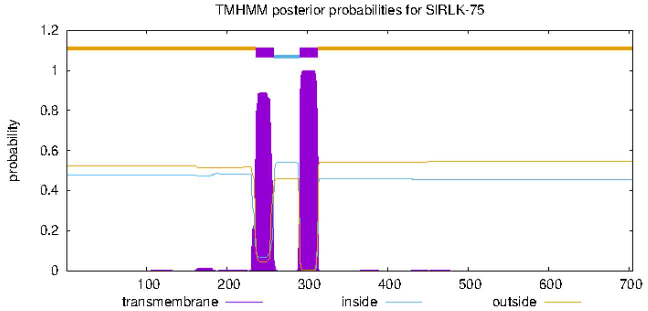

SlRLK-75

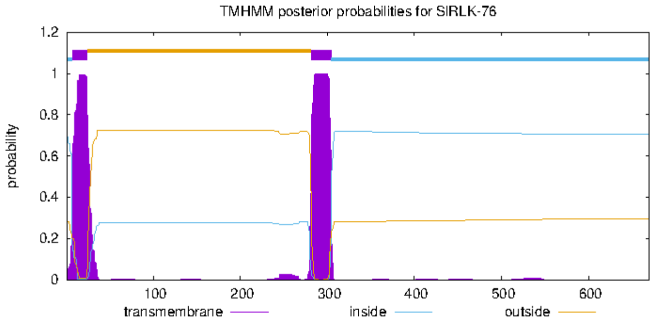

SlRLK-76

Fig. S21. The TMHMM results predicted the transmembrane helices

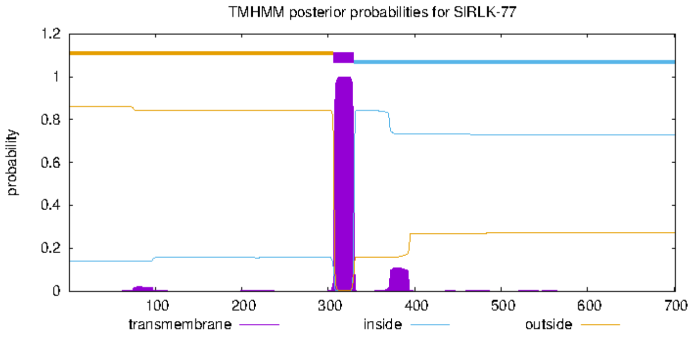

SlRLK-77

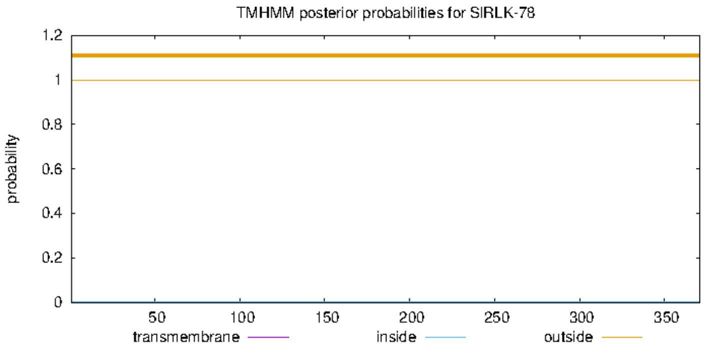

SlRLK-78

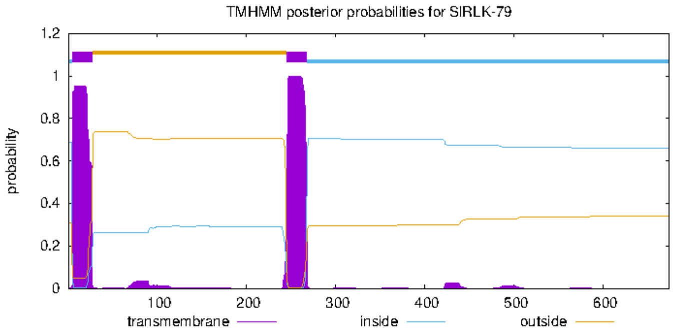

SlRLK-79

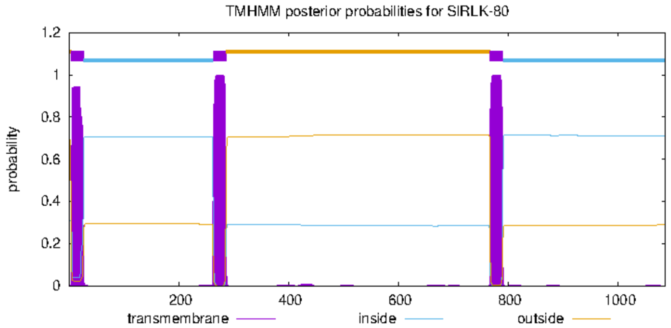

SlRLK-80

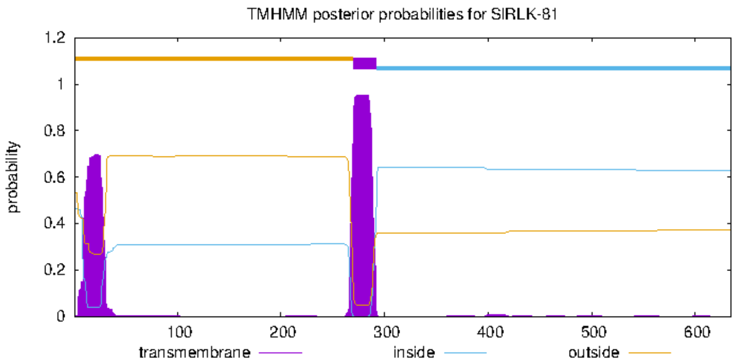

SlRLK-81

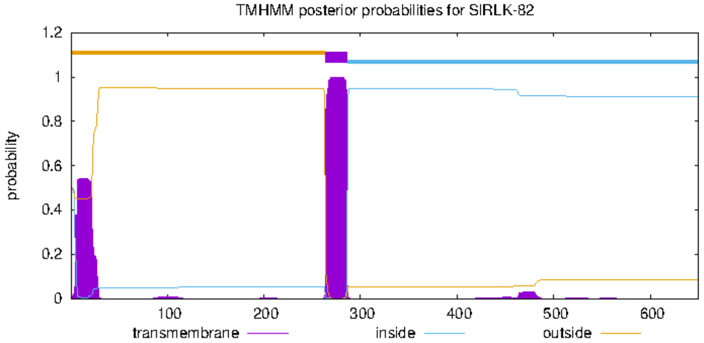

SlRLK-82

Fig. S22. The TMHMM results predicted the transmembrane helices

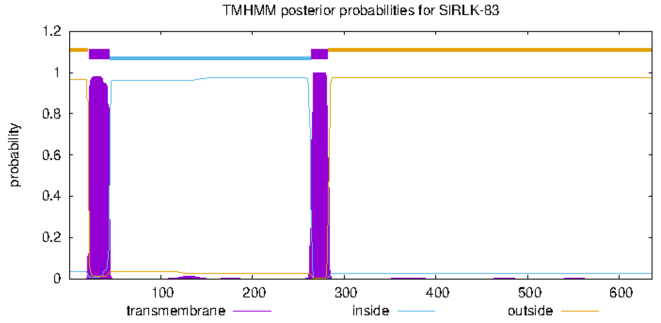

SlRLK-83

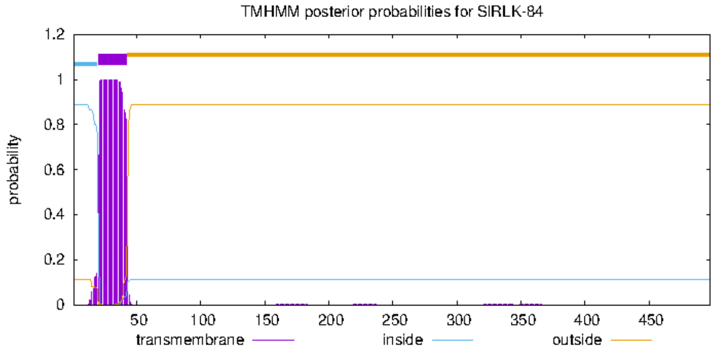

SlRLK-84

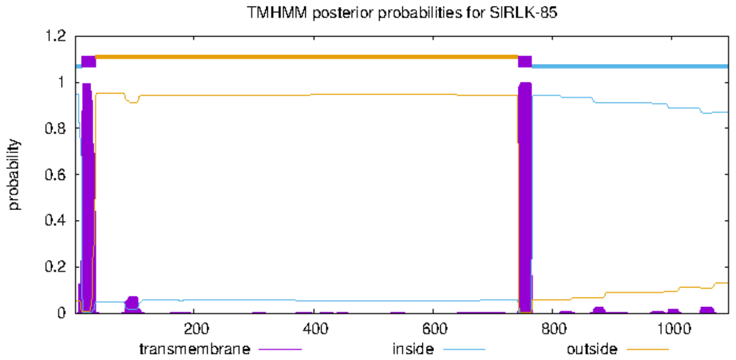

SlRLK-85

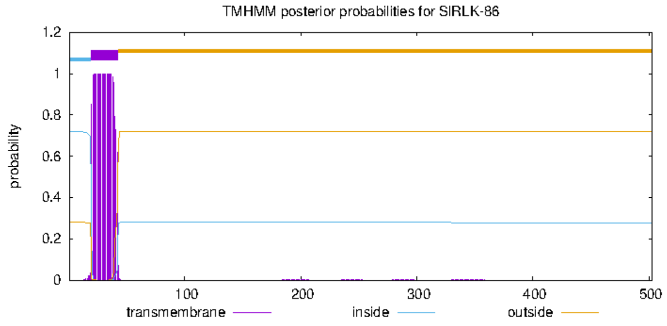

SlRLK-86

Fig. S23. The TMHMM results predicted the transmembrane helices

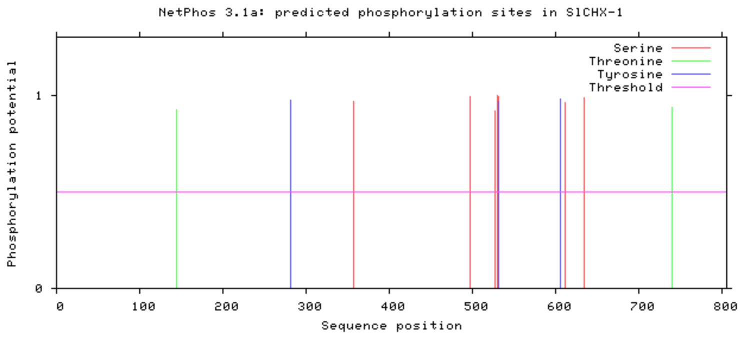

SlCHX-1

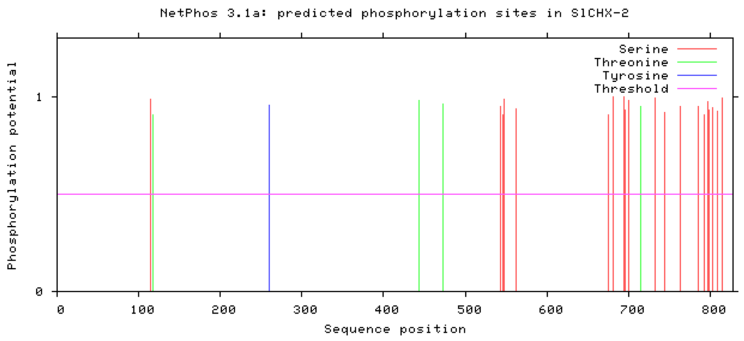

SlCHX-2

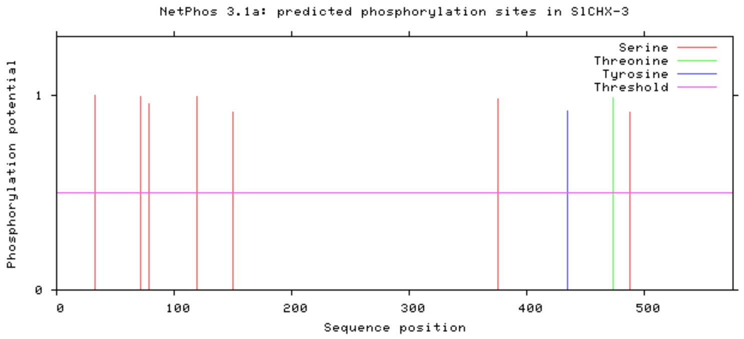

SlCHX-3

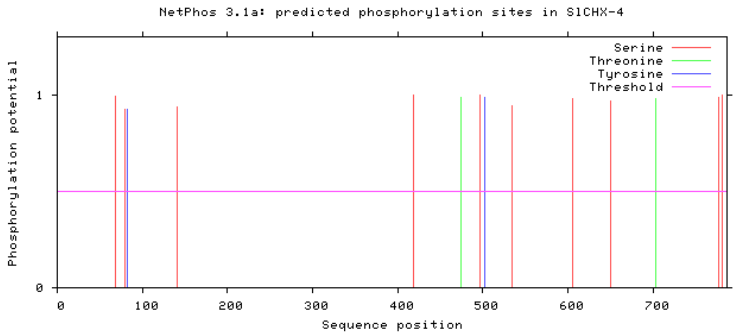

SlCHX-4

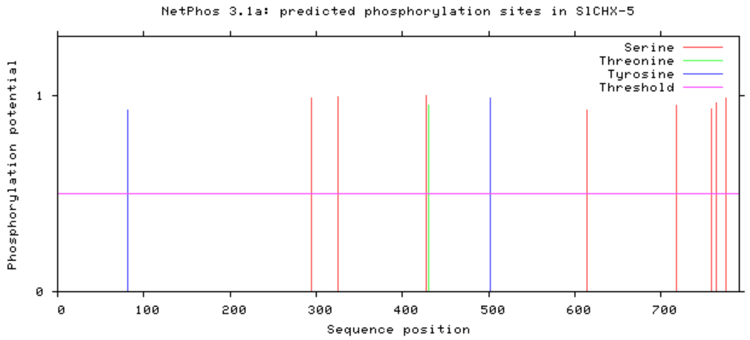

SlCHX-5

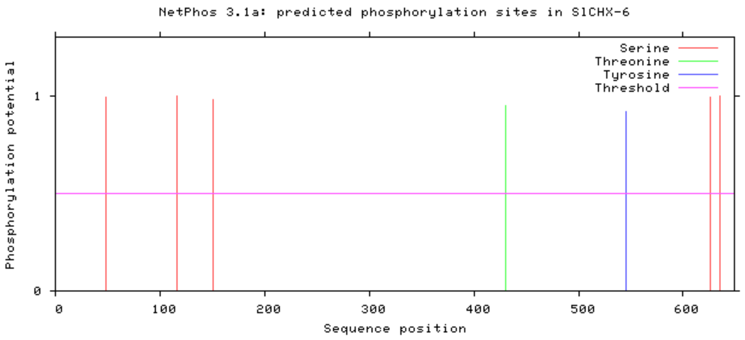

SlCHX-6

Fig. S24. Phosphorylation site prediction for kinases

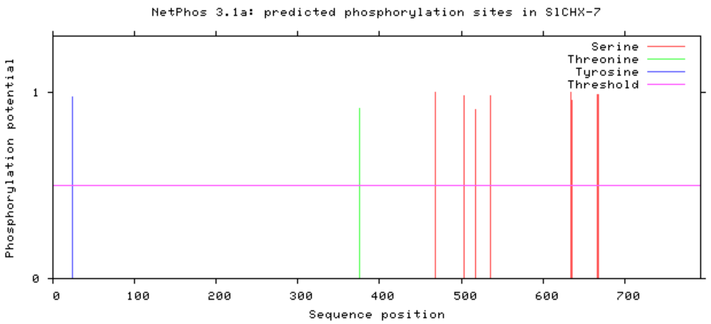

SlCHX-7

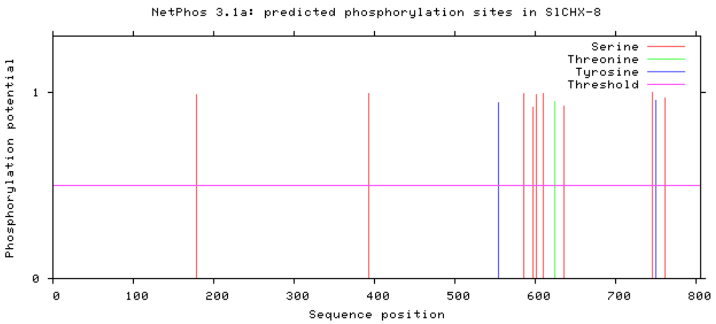

SlCHX-8

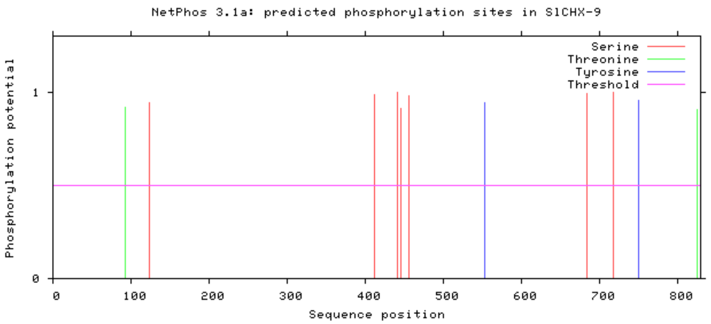

SlCHX-9

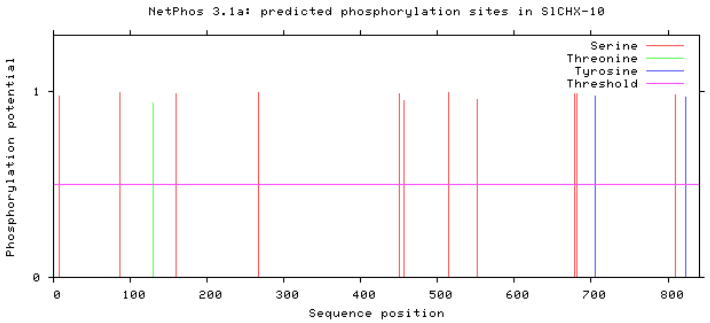

SlCHX-10

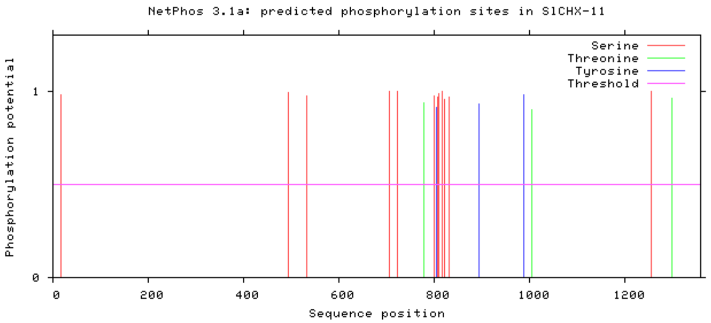

SlCHX-11

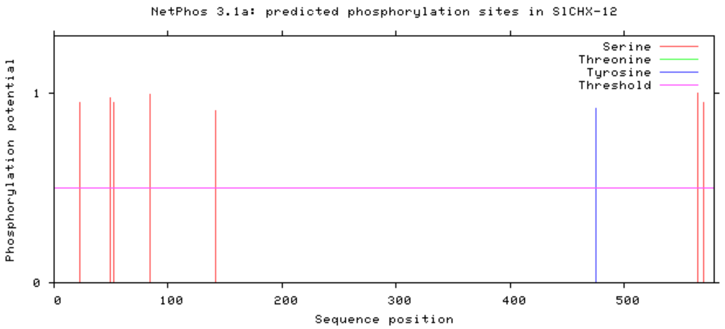

SlCHX-12

Fig. S25. Phosphorylation site prediction for kinases

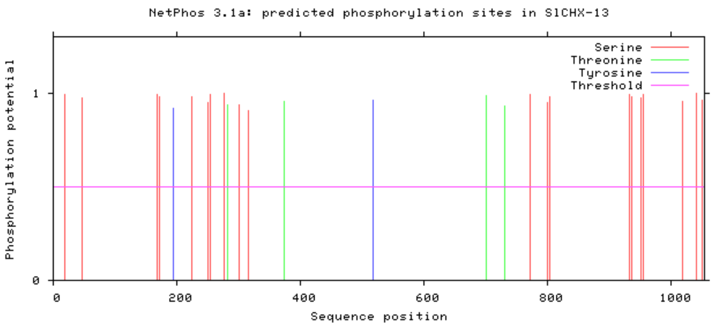

SlCHX-13

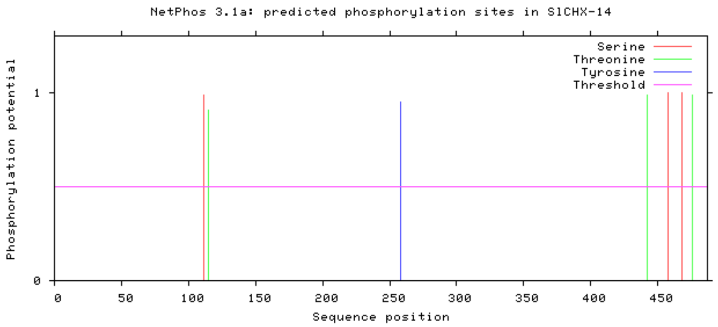

SlCHX-14

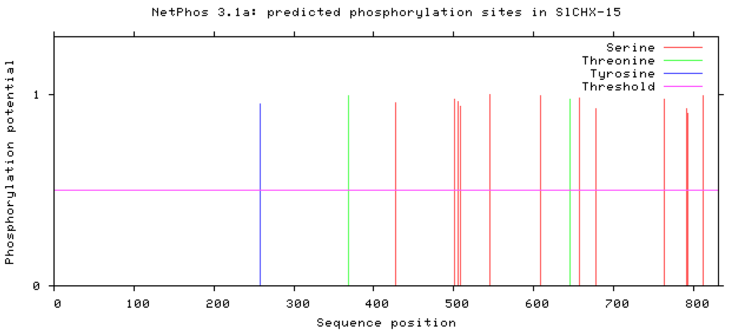

SlCHX-15

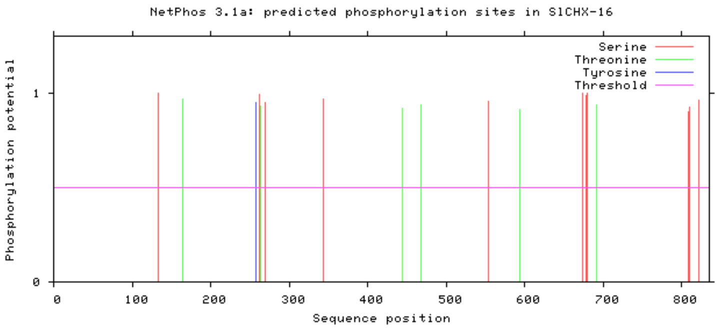

SlCHX-16

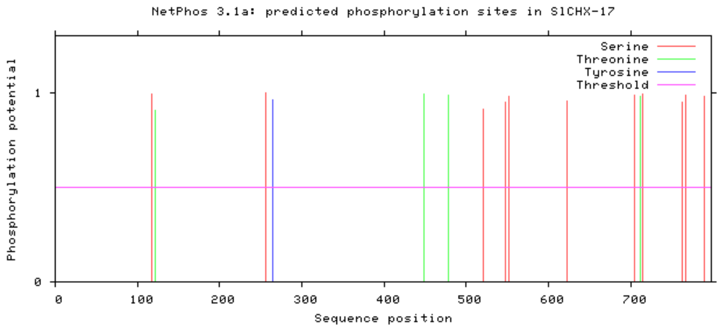

SlCHX-17

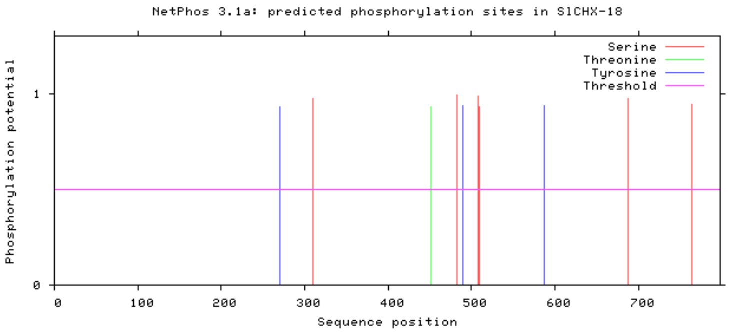

SlCHX-18

Fig. S26. Phosphorylation site prediction for kinases

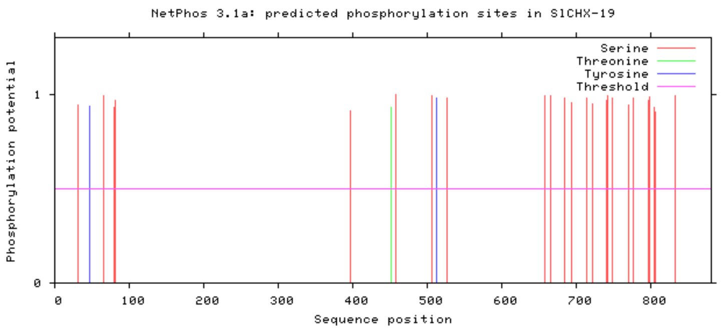

SlCHX-19

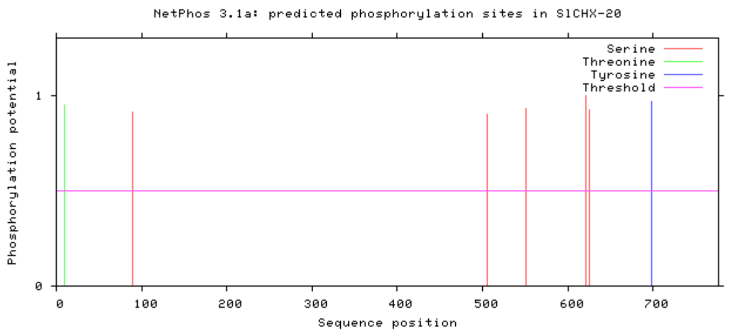

SlCHX-20

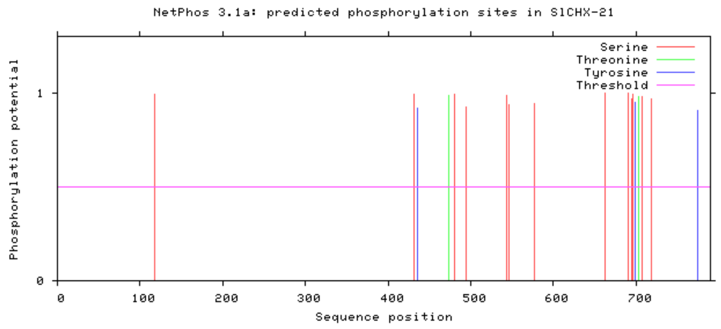

SlCHX-21

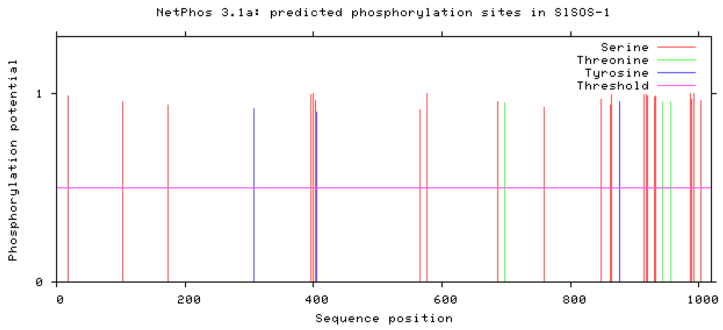

SlSOS-1

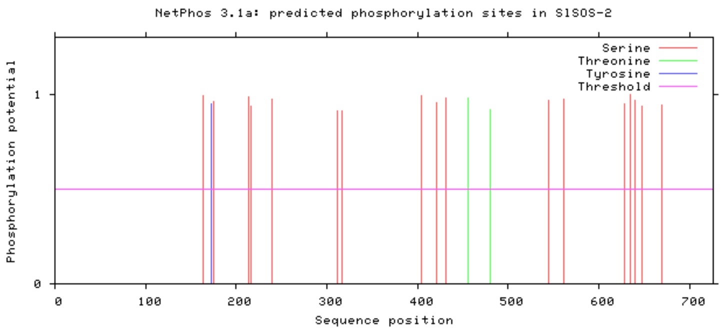

SlSOS-2

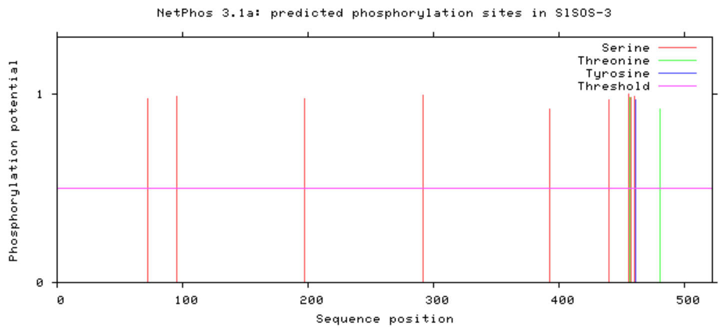

SlSOS-3

Fig. S27. Phosphorylation site prediction for kinases

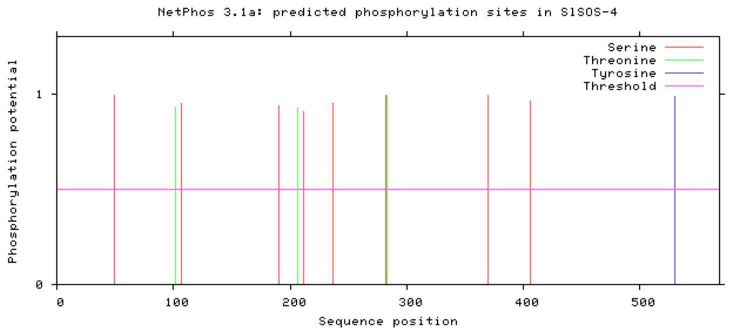

SlSOS-4

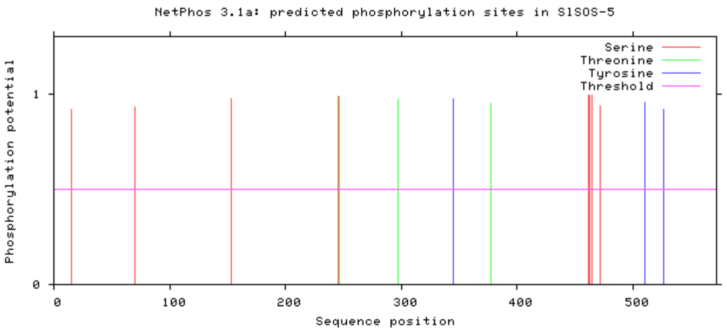

SlSOS-5

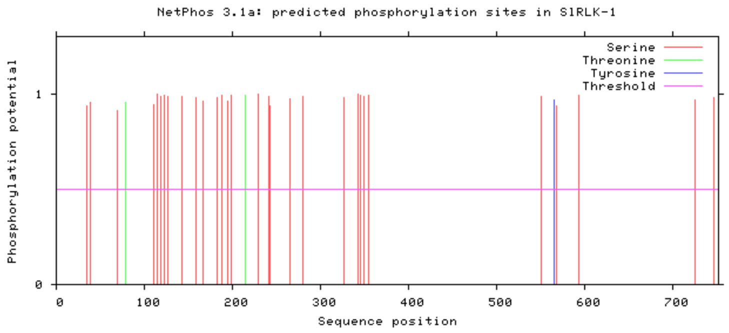

SlRLK-1

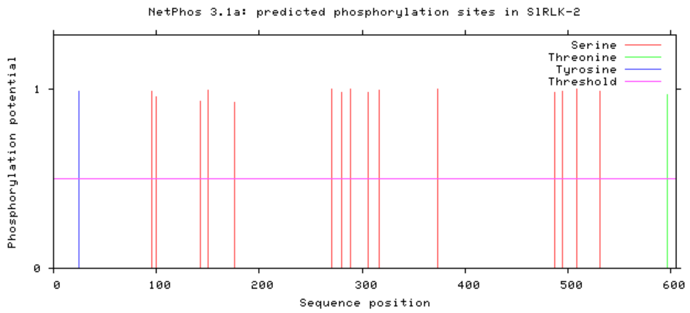

SlRLK-2

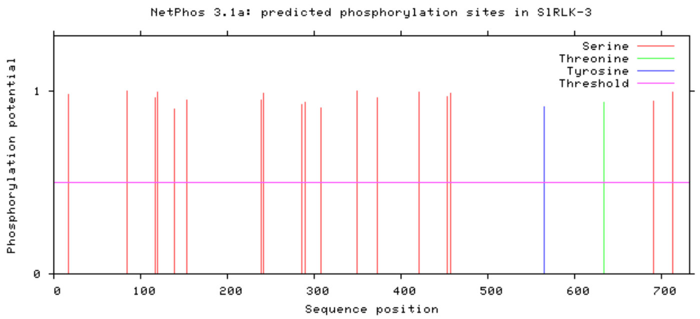

SlRLK-3

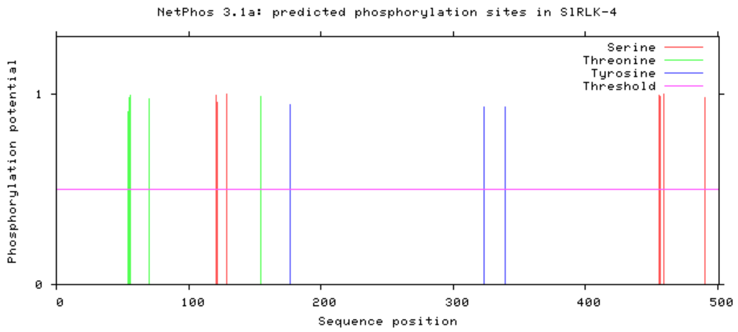

SlRLK-4

Fig. S28. Phosphorylation site prediction for kinases

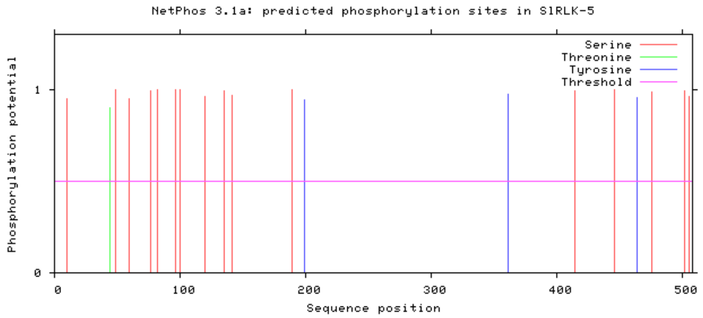

SlRLK-5

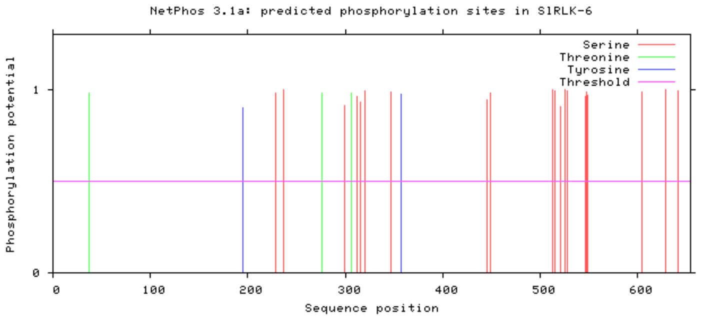

SlRLK-6

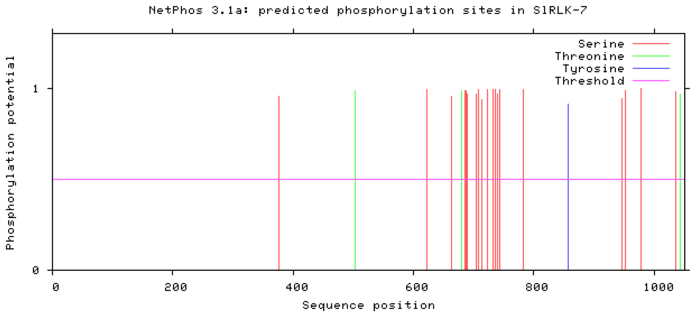

SlRLK-7

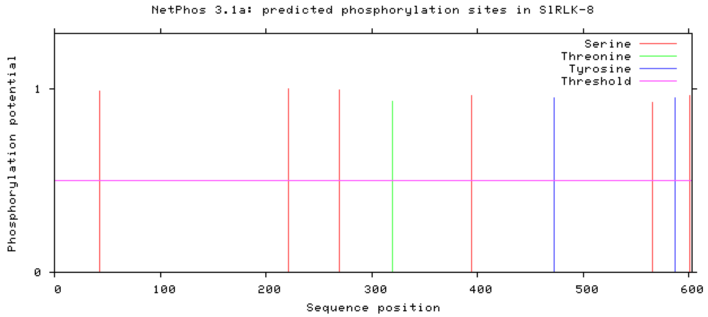

SlRLK-8

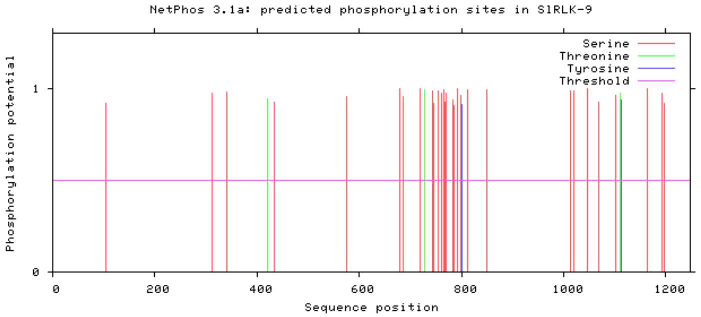

SlRLK-9

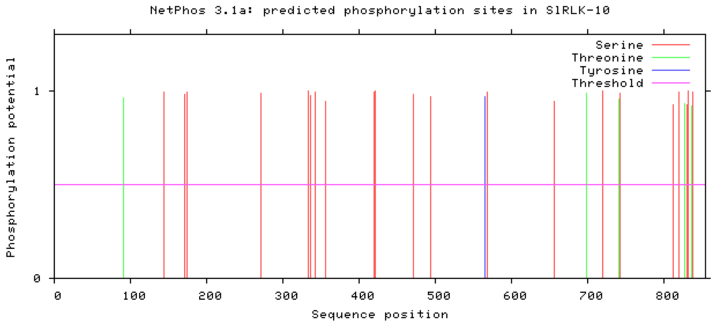

SlRLK-10

Fig. S29. Phosphorylation site prediction for kinases

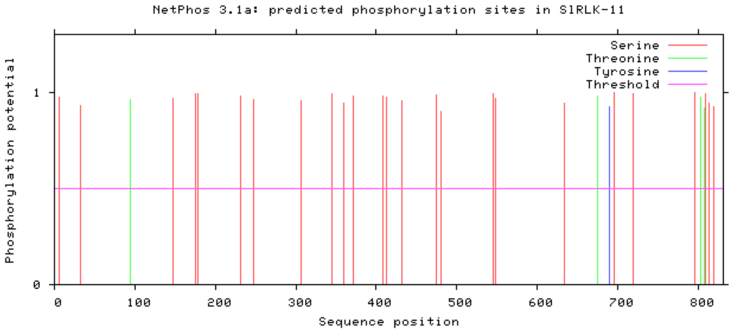

SlRLK-11

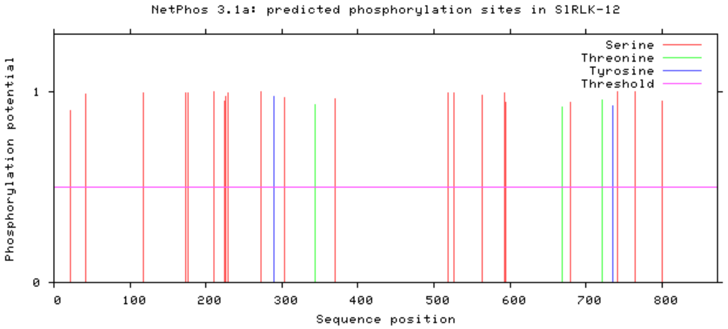

SlRLK-12

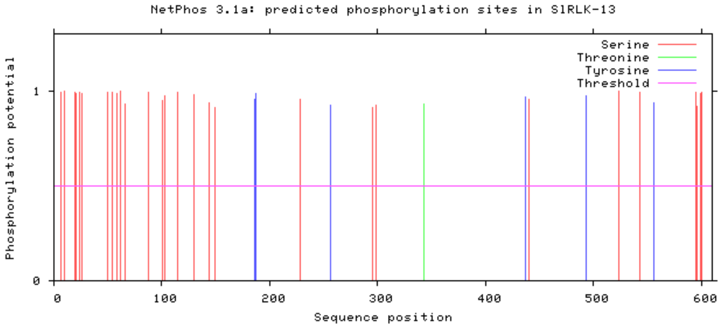

SlRLK-13

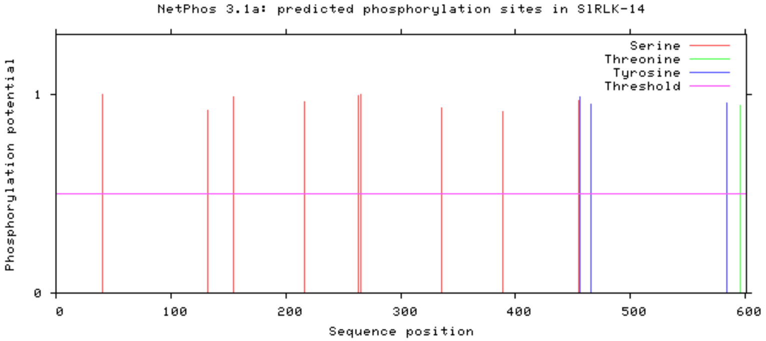

SlRLK-14

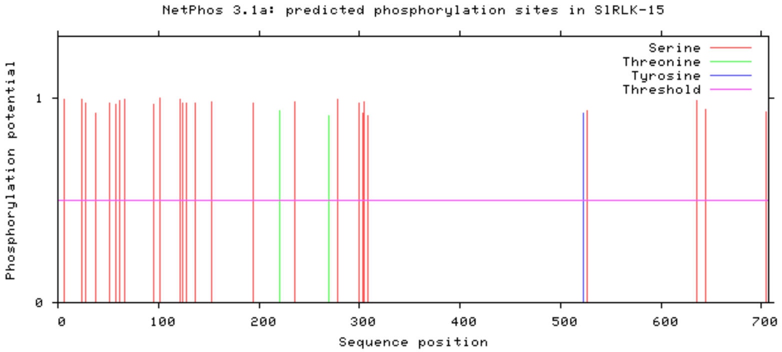

SlRLK-15

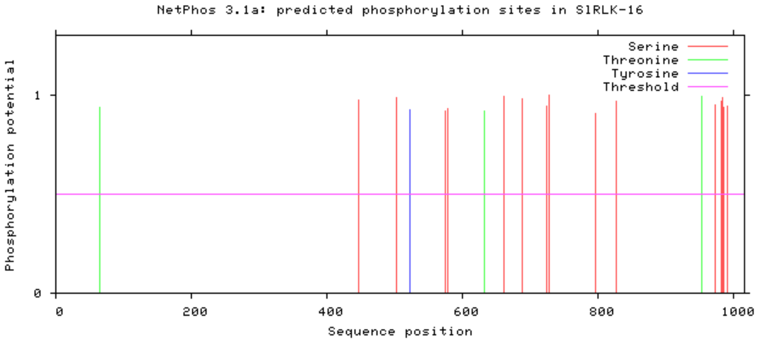

SlRLK-16

Fig. S30. Phosphorylation site prediction for kinases

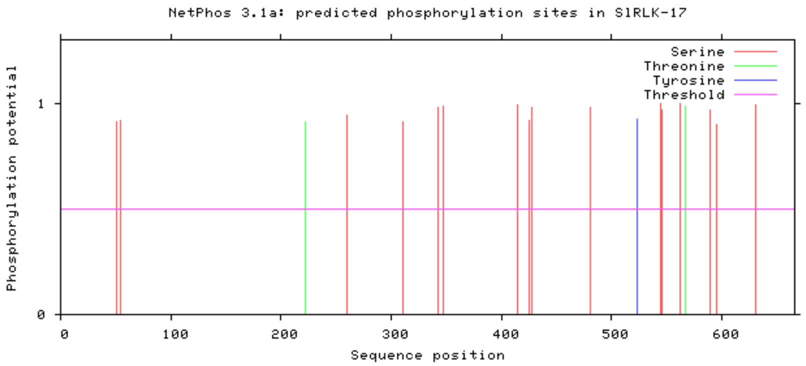

SlRLK-17

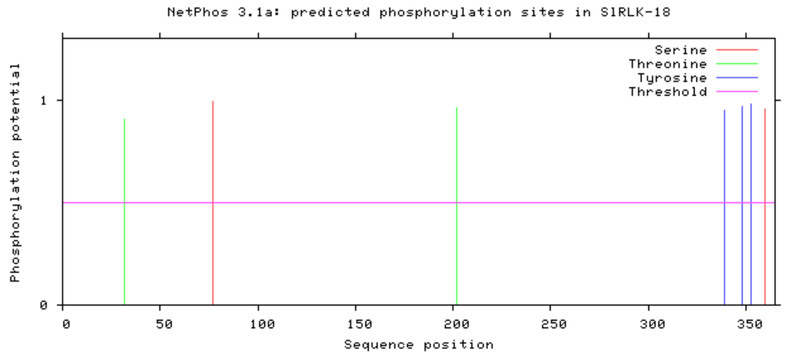

SlRLK-18

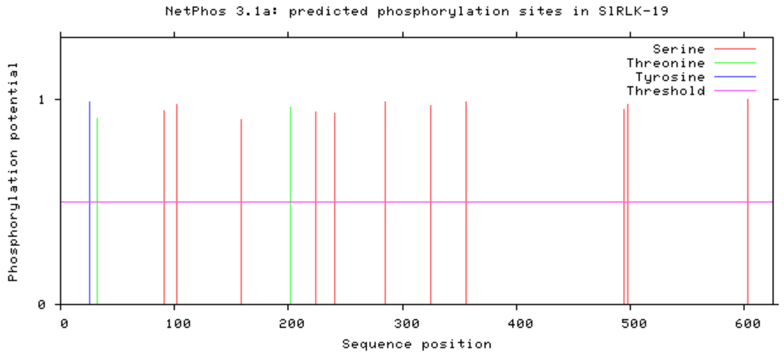

SlRLK-19

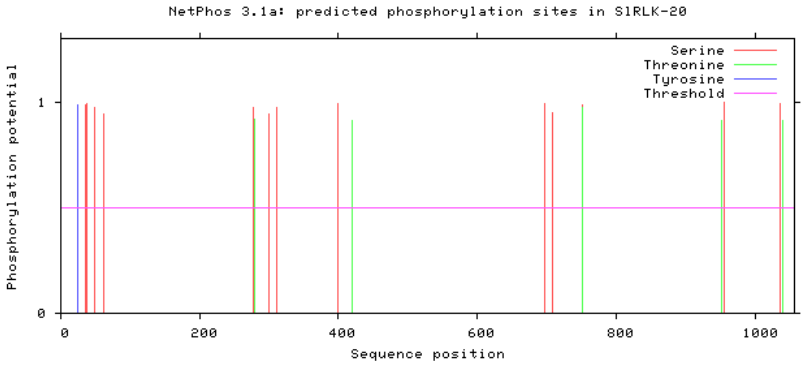

SlRLK-20

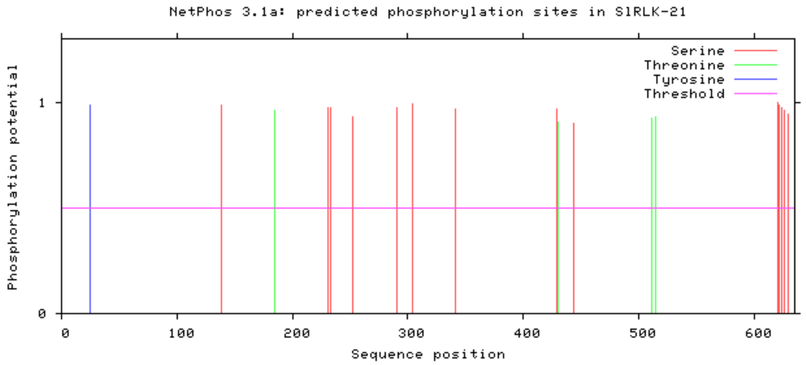

SlRLK-21

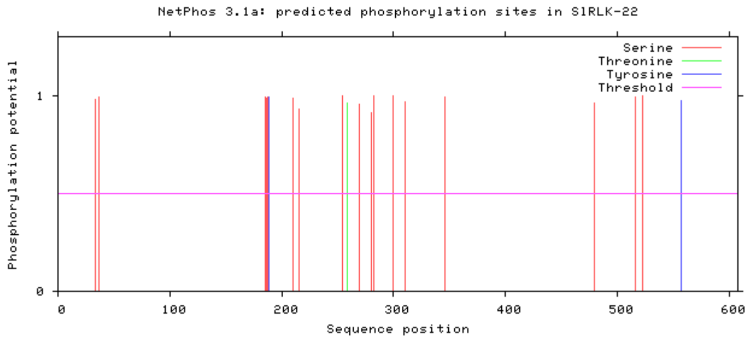

SlRLK-22

Fig. S31. The Phosphorylation site prediction for kinases

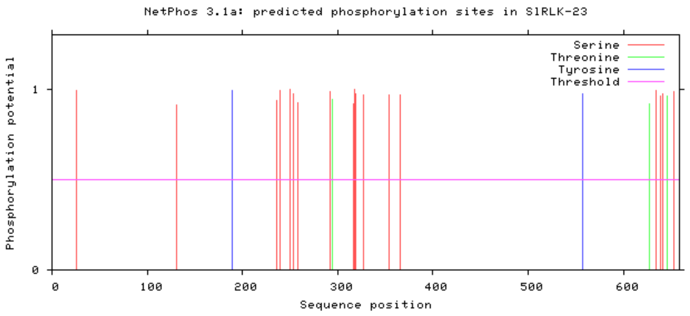

SlRLK-23

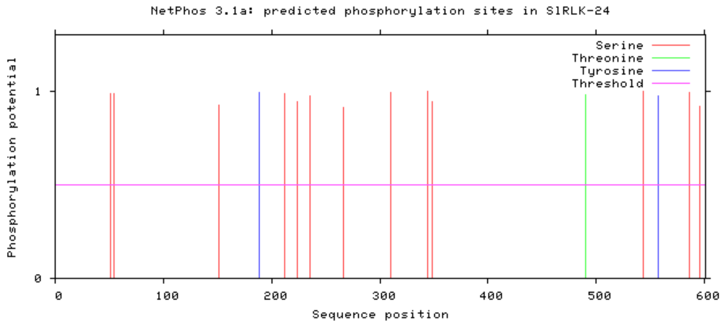

SlRLK-24

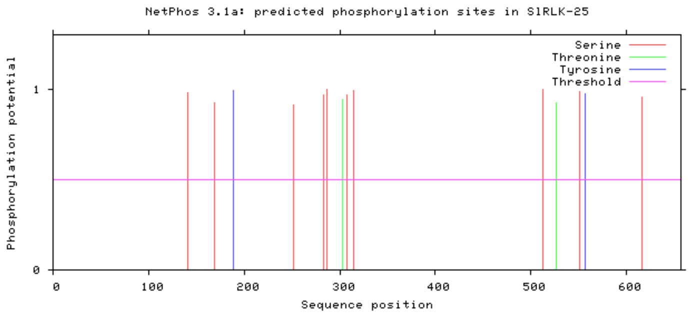

SlRLK-25

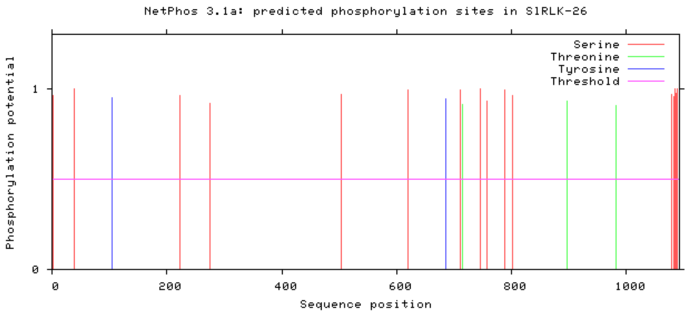

SlRLK-26

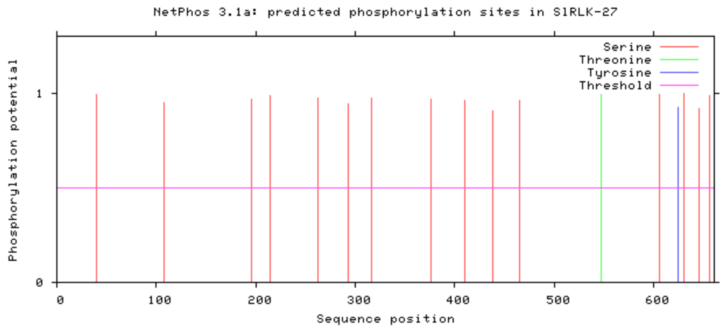

SlRLK-27

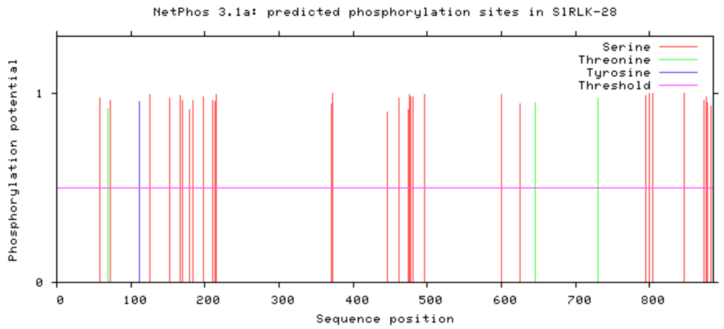

SlRLK-28

Fig. S32. Phosphorylation site prediction for kinases

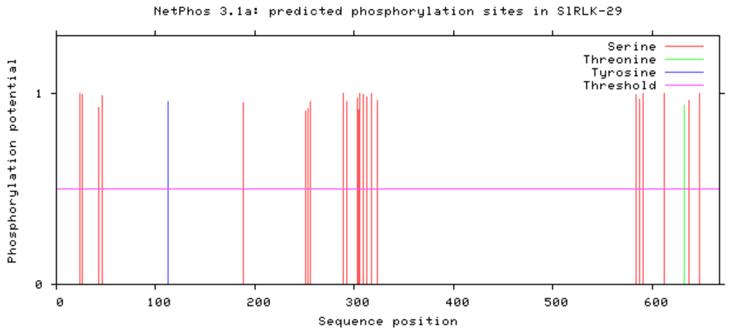

SlRLK-29

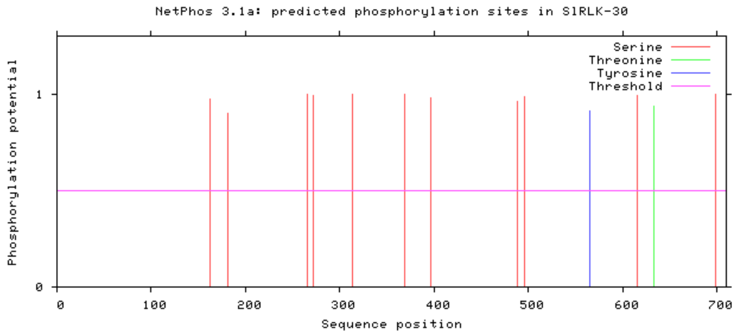

SlRLK-30

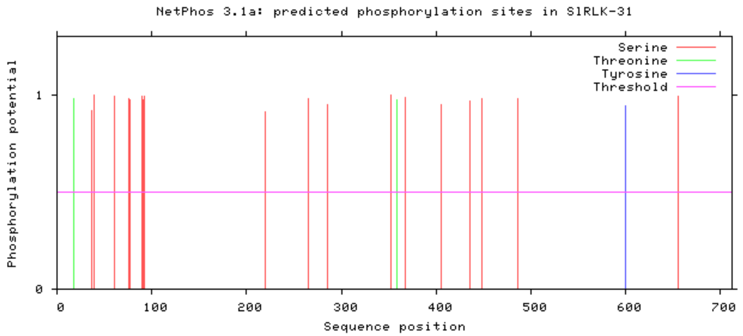

SlRLK-31

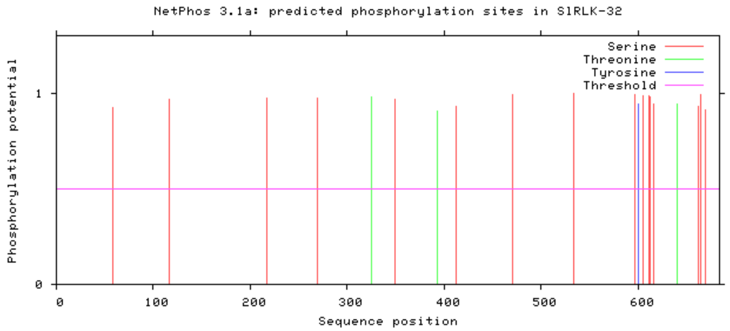

SlRLK-32

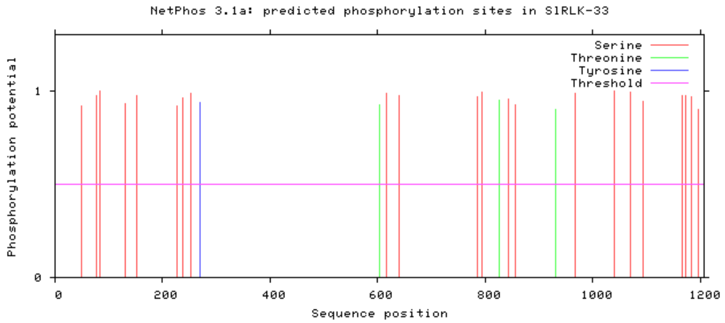

SlRLK-33

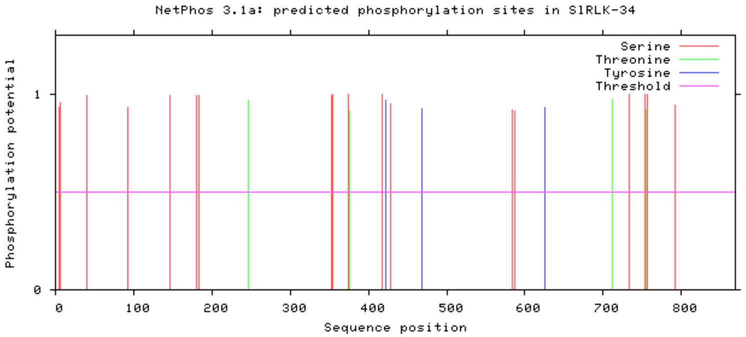

SlRLK-34

Fig. S33. Phosphorylation site prediction for kinases

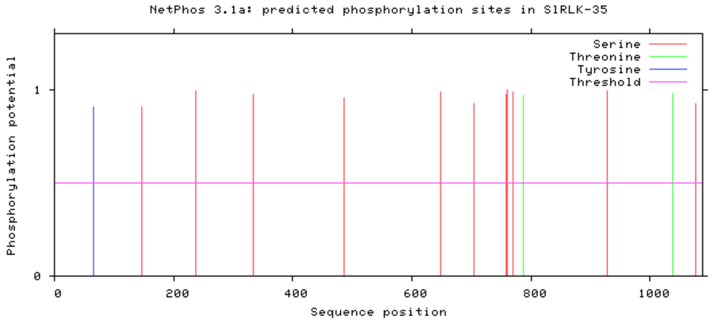

SlRLK-35

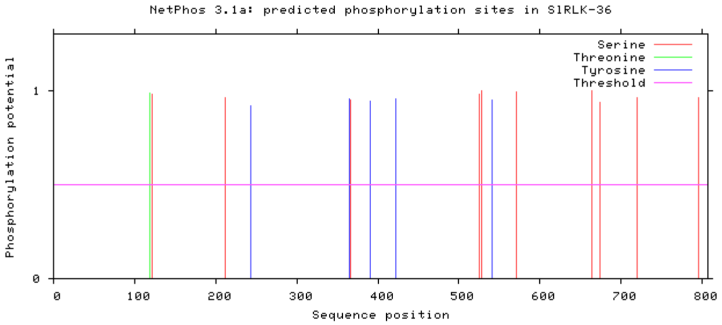

SlRLK-36

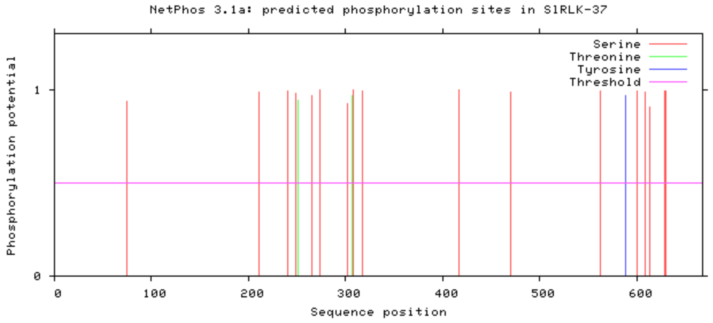

SlRLK-37

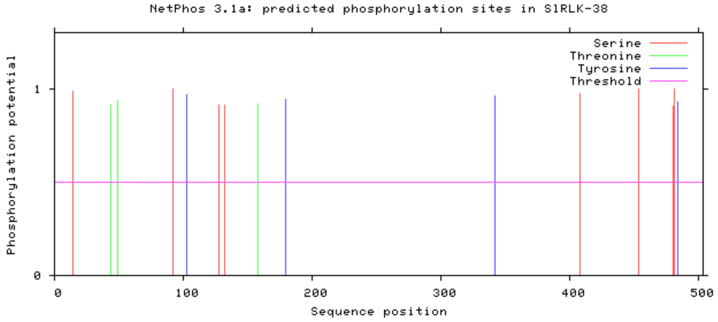

SlRLK-38

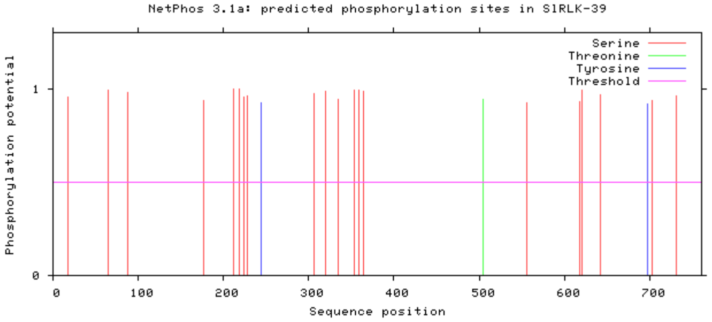

SlRLK-39

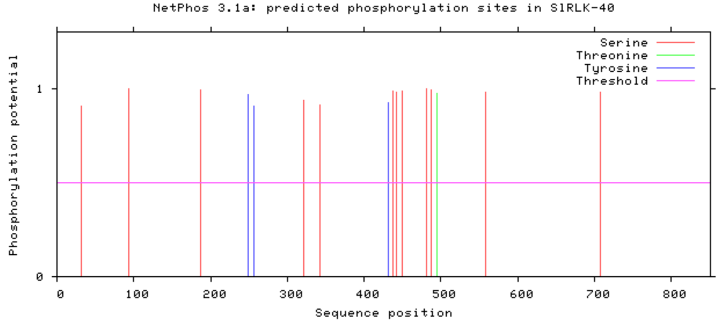

SlRLK-40

Fig. S34. Phosphorylation site prediction for kinases

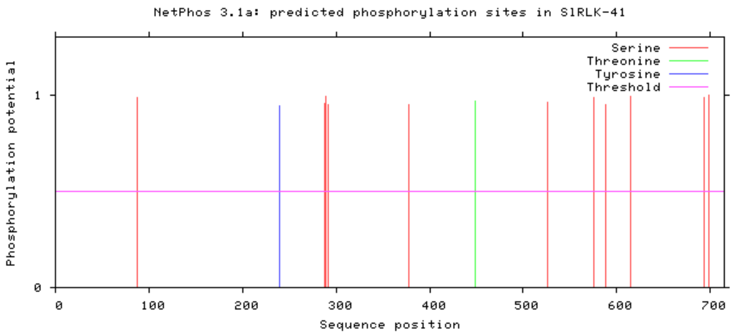

SlRLK-41

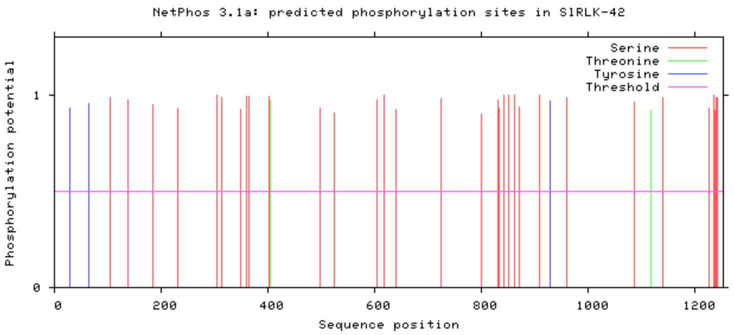

SlRLK-42

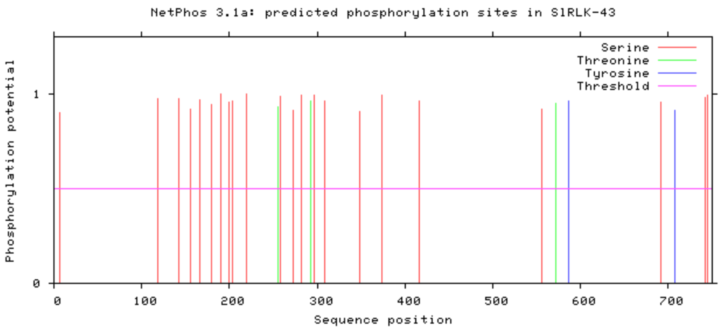

SlRLK-43

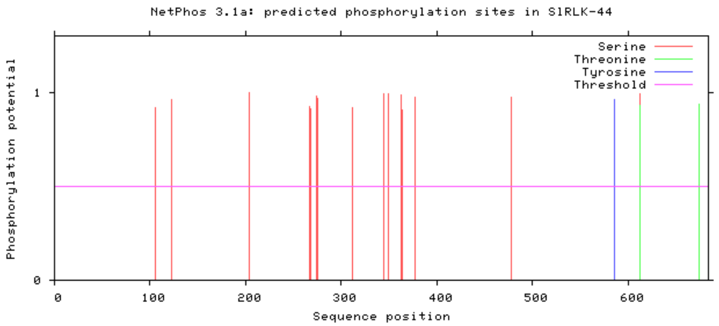

SlRLK-44

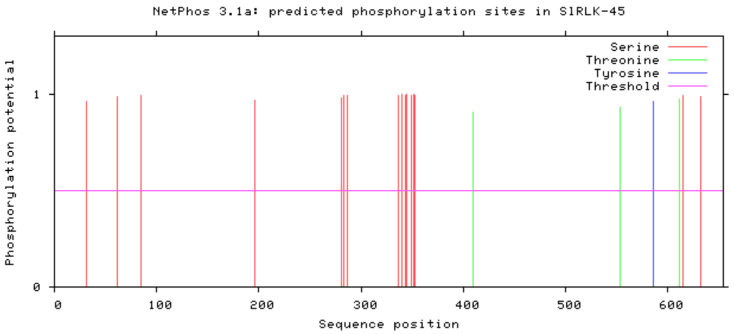

SlRLK-45

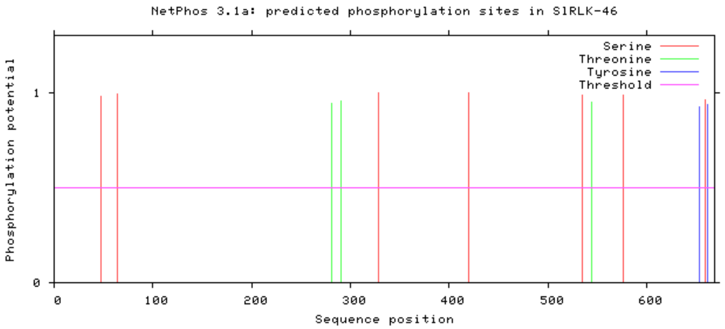

SlRLK-46

Fig. S35. Phosphorylation site prediction for kinases

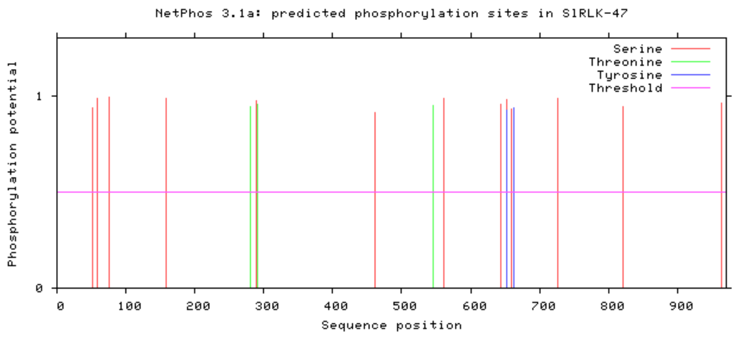

SlRLK-47

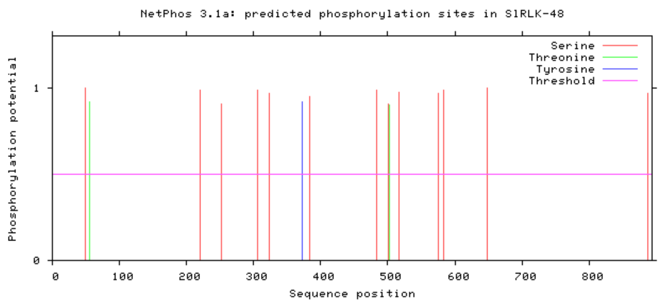

SlRLK-48

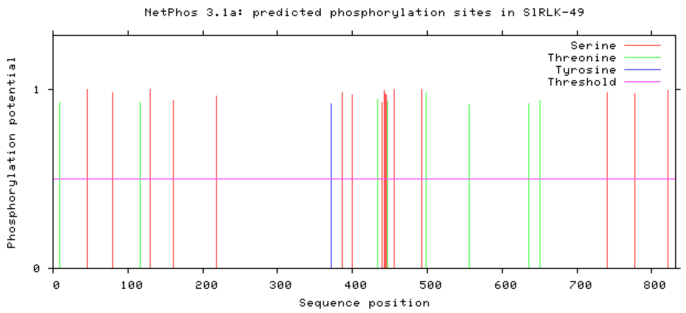

SlRLK-49

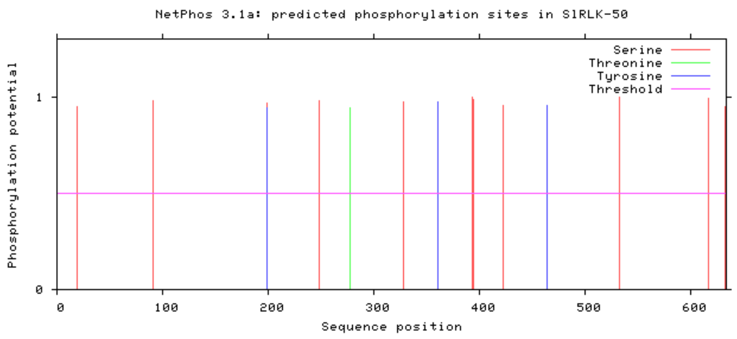

SlRLK-50

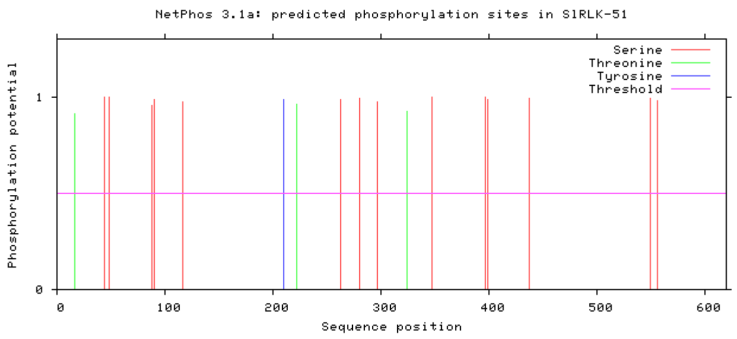

SlRLK-51

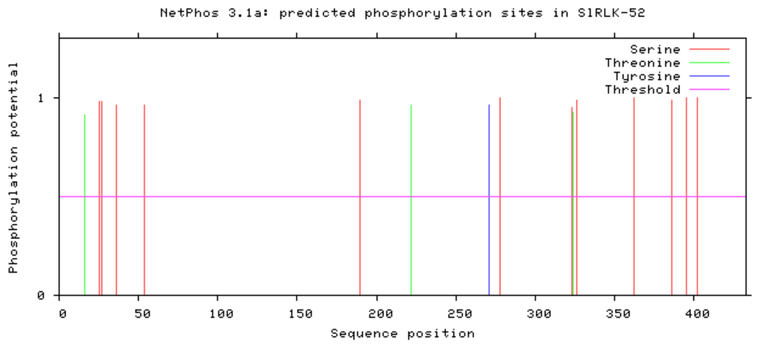

SlRLK-52

Fig. S36. Phosphorylation site prediction for kinases

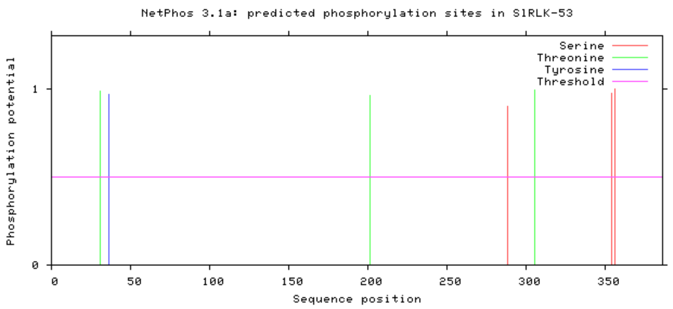

SlRLK-53

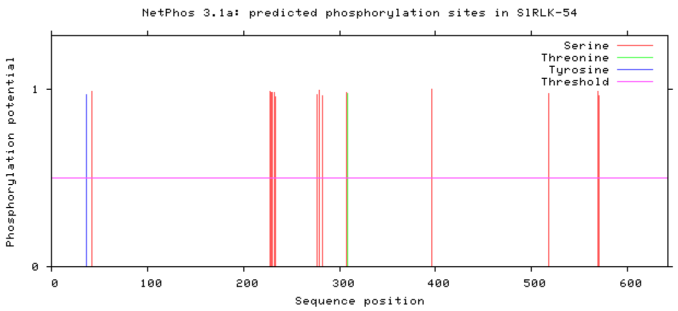

SlRLK-54

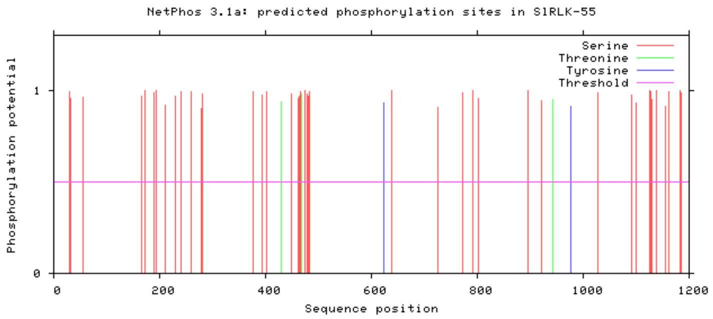

SlRLK-55

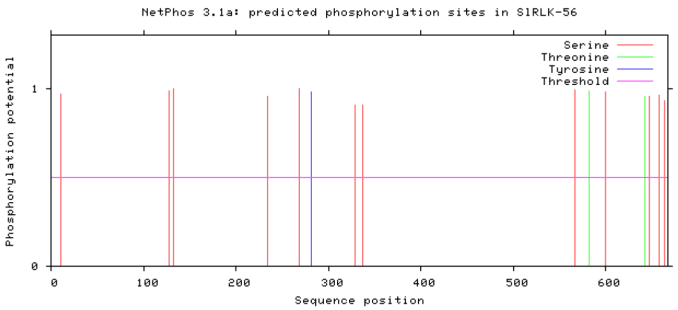

SlRLK-56

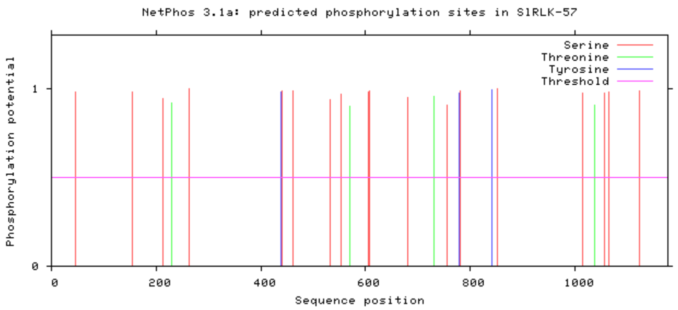

SlRLK-57

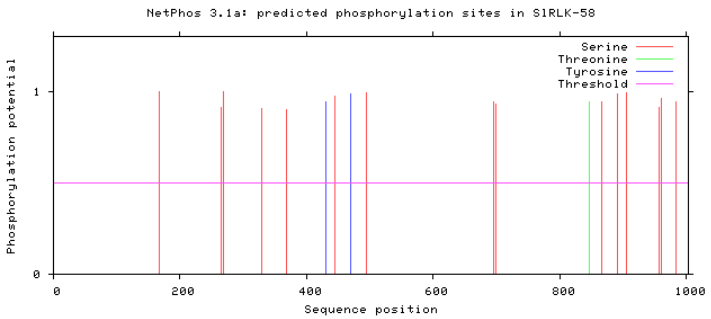

SlRLK-58

Fig. S37. Phosphorylation site prediction for kinases

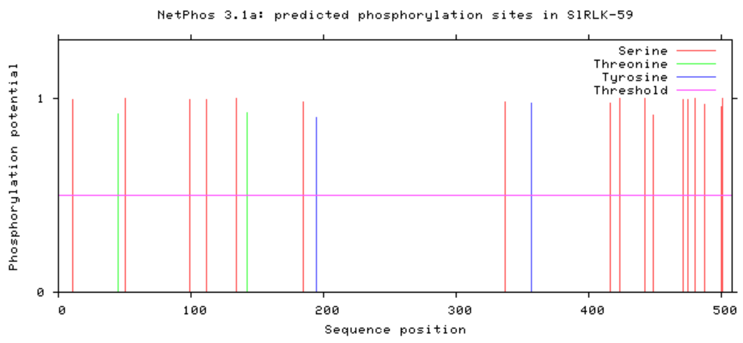

SlRLK-59

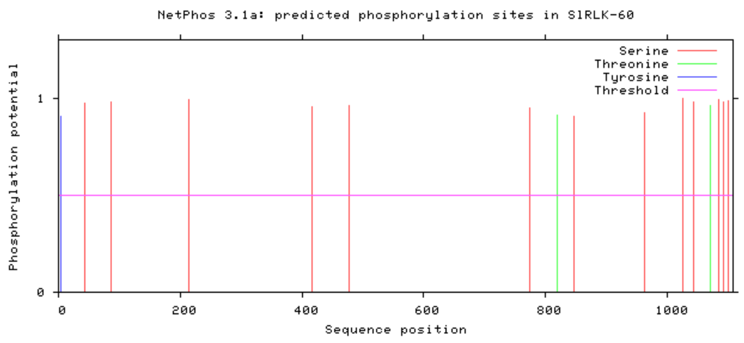

SlRLK-60

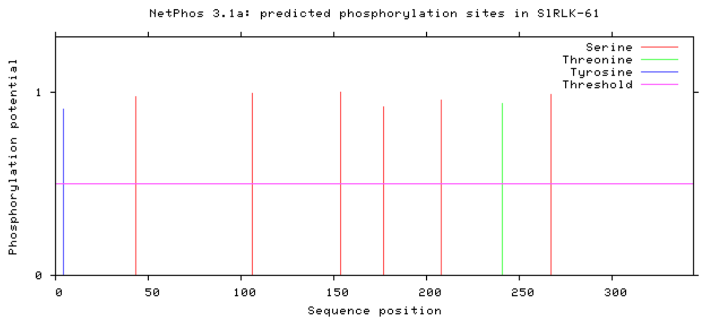

SlRLK-61

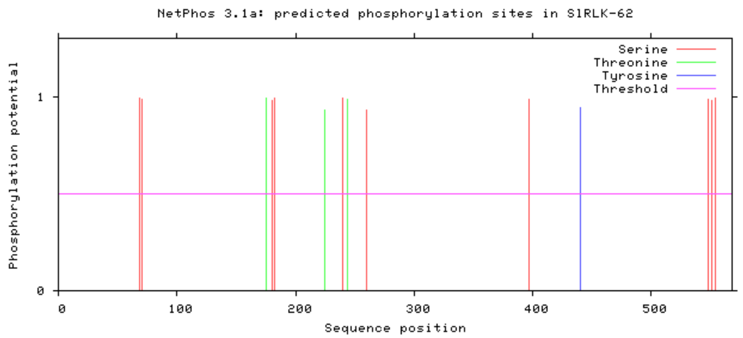

SlRLK-62

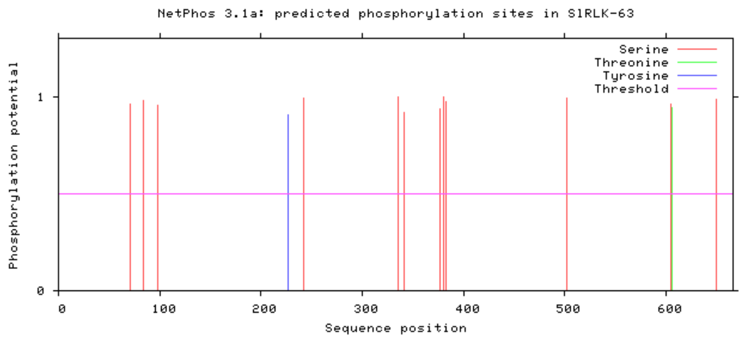

SlRLK-63

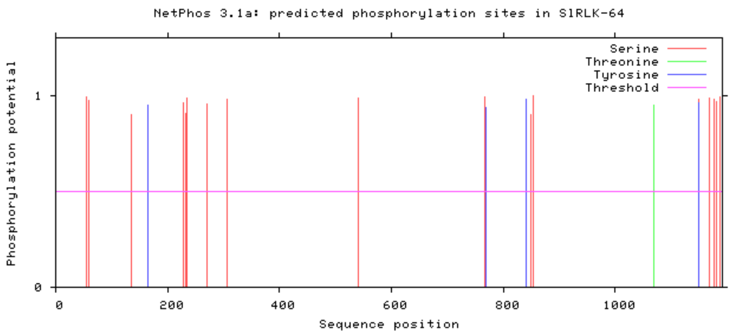

SlRLK-64

Fig. S38. Phosphorylation site prediction for kinases

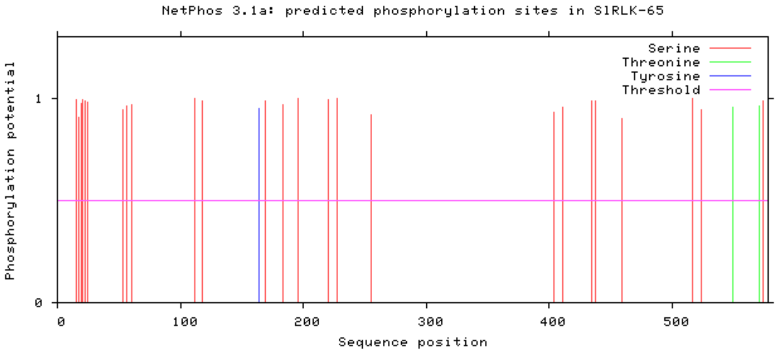

SlRLK-65

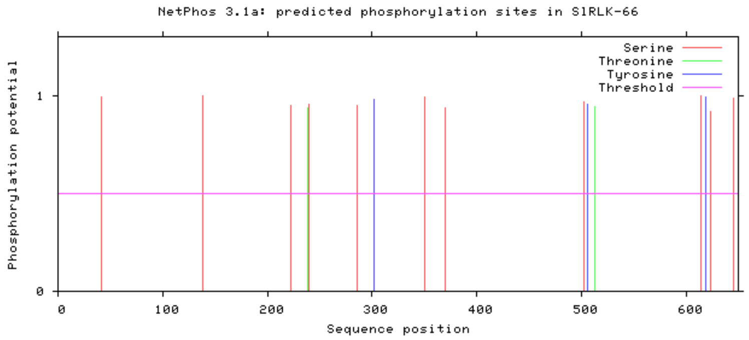

SlRLK-66

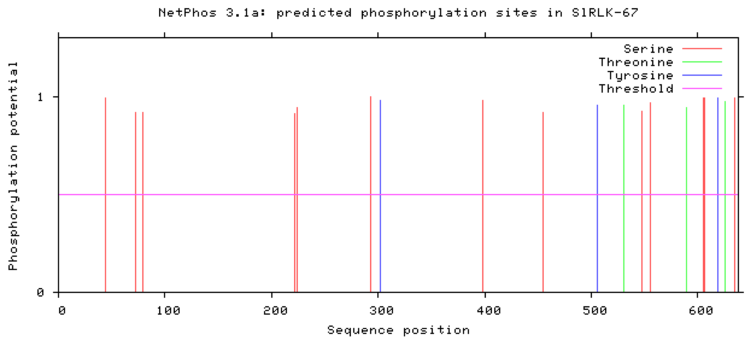

SlRLK-67

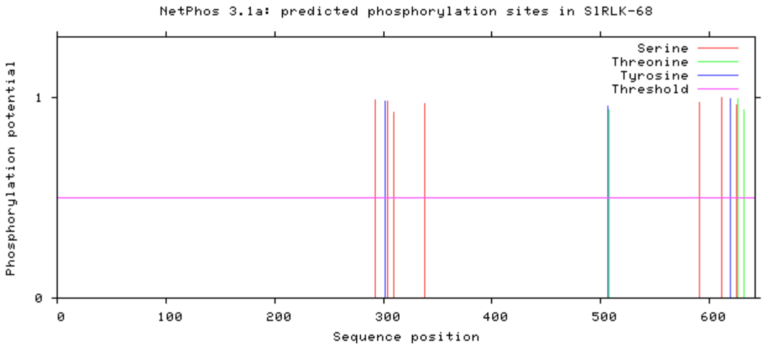

SlRLK-68

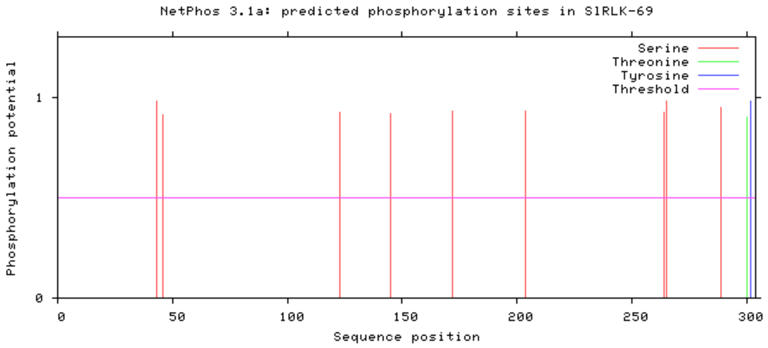

SlRLK-69

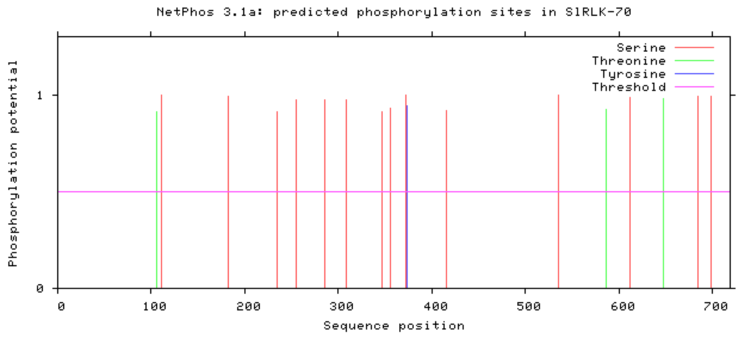

SlRLK-70

Fig. S39. Phosphorylation site prediction for kinases

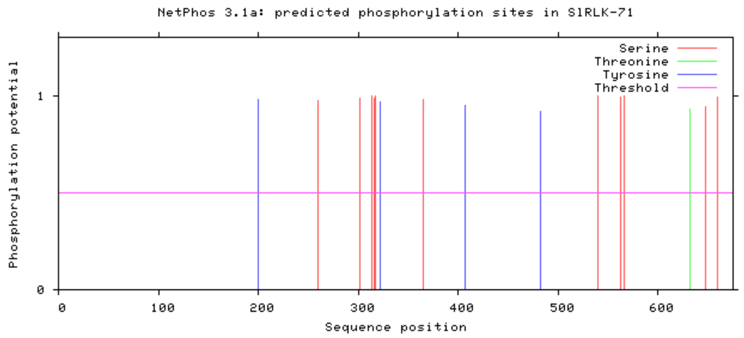

SlRLK-71

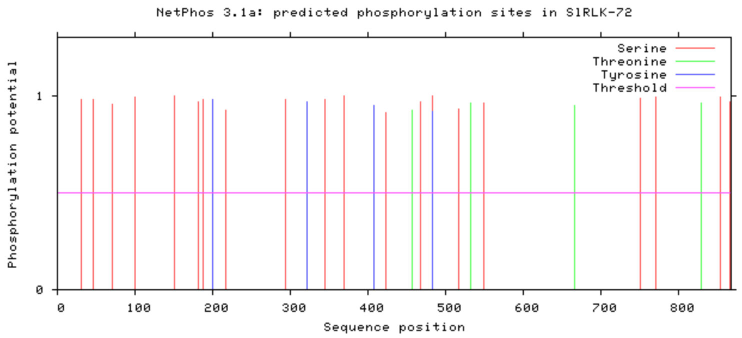

SlRLK-72

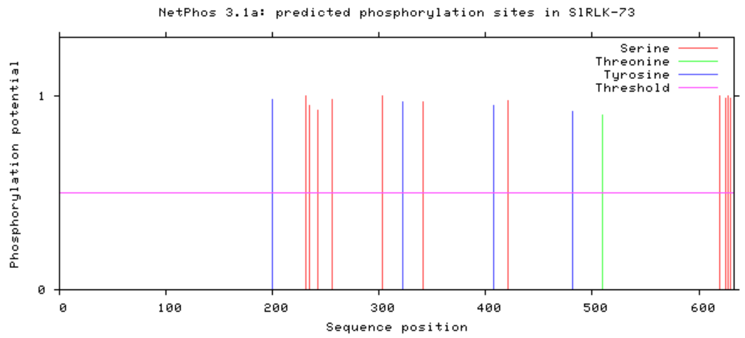

SlRLK-73

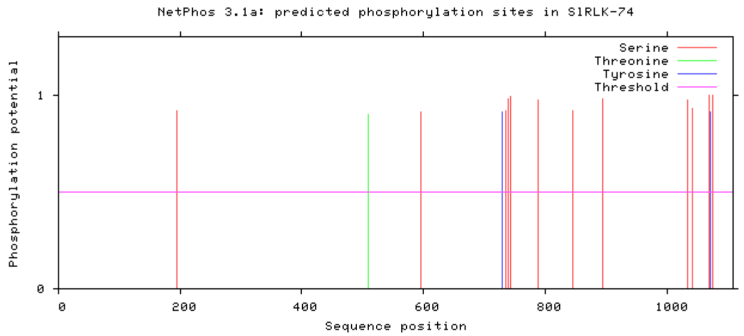

SlRLK-74

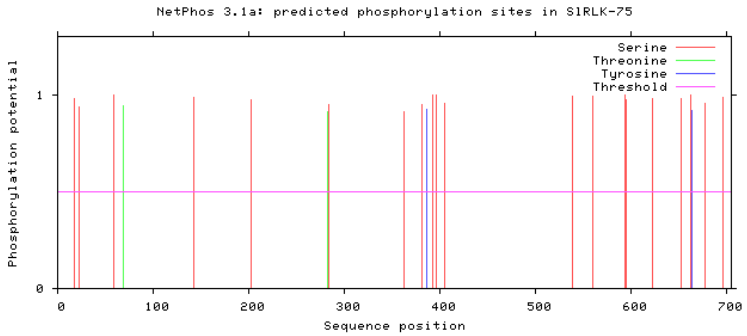

SlRLK-75

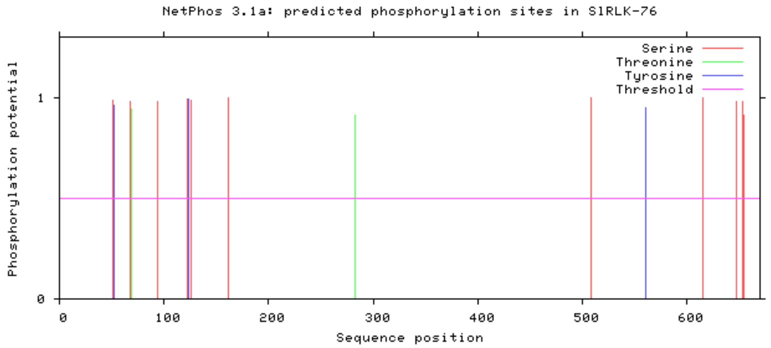

SlRLK-76

Fig. S40. Phosphorylation site prediction for kinases

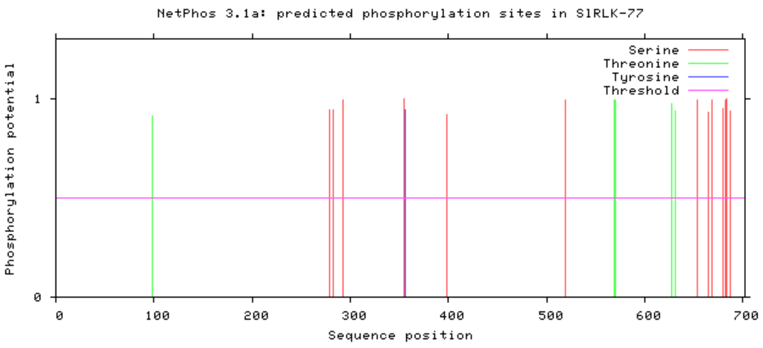

SlRLK-77

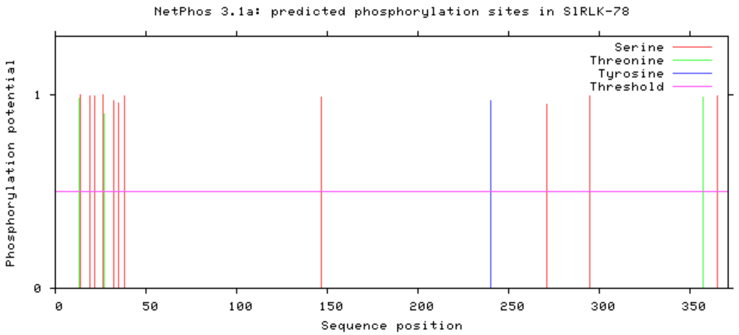

SlRLK-78

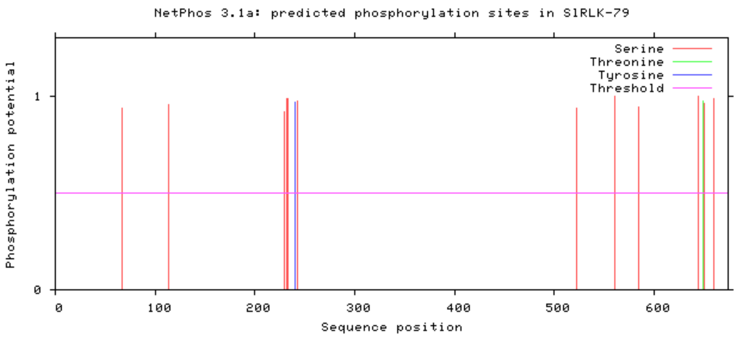

SlRLK-79

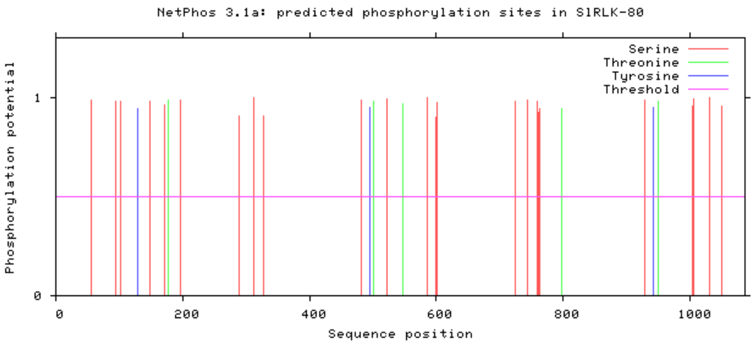

SlRLK-80

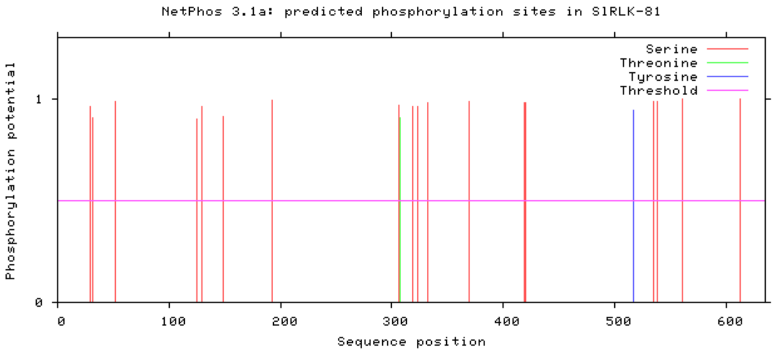

SlRLK-81

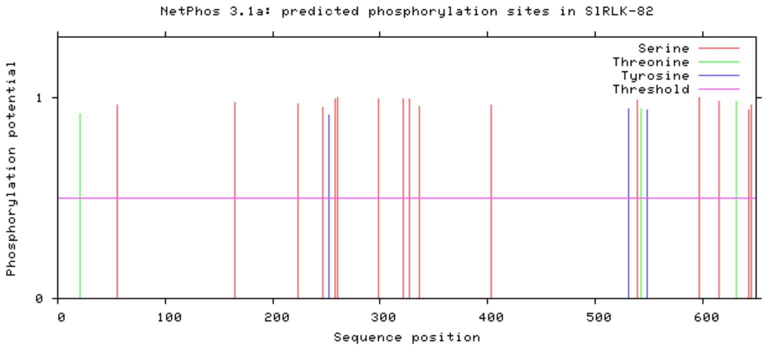

SlRLK-82

Fig. S41. Phosphorylation site prediction for kinases

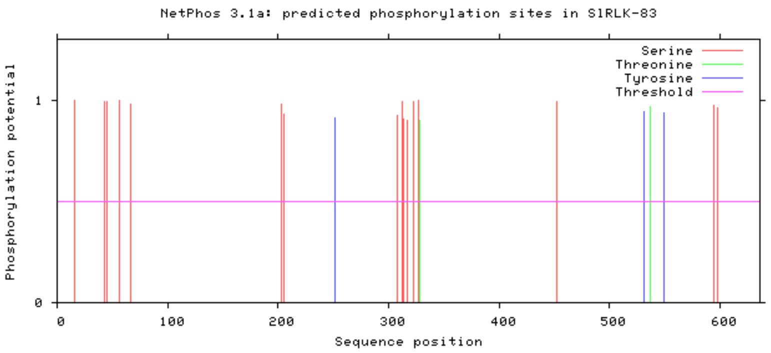

SlRLK-83

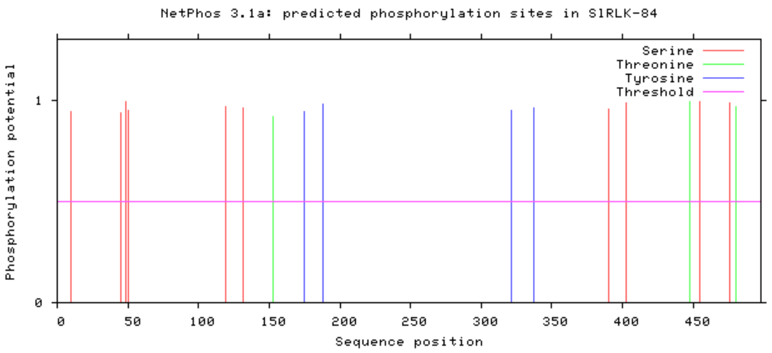

SlRLK-84

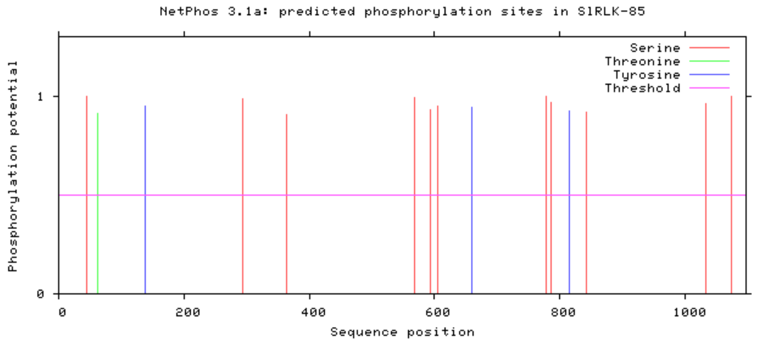

SlRLK-85

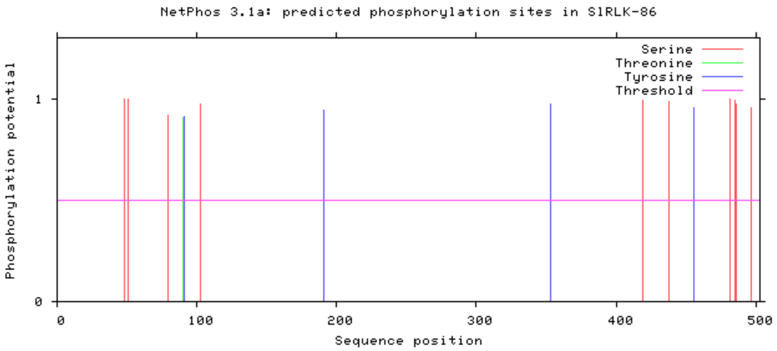

SlRLK-86

Fig. S42. Phosphorylation site prediction for kinases

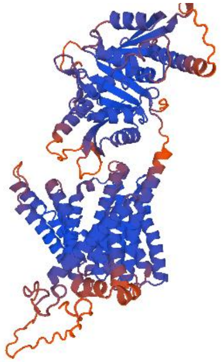

SlCHX-1

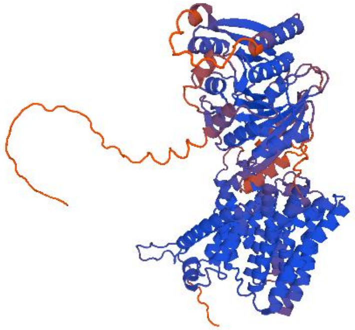

SlCHX-2

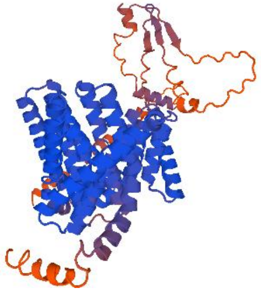

SlCHX-3

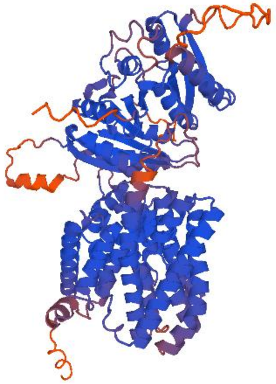

SlCHX-4

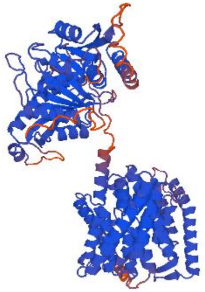

SlCHX-5

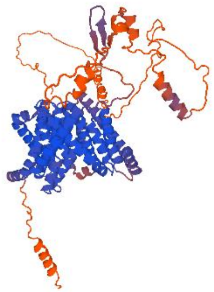

SlCHX-6

Fig. S43. Proteins modeled with swiss-model

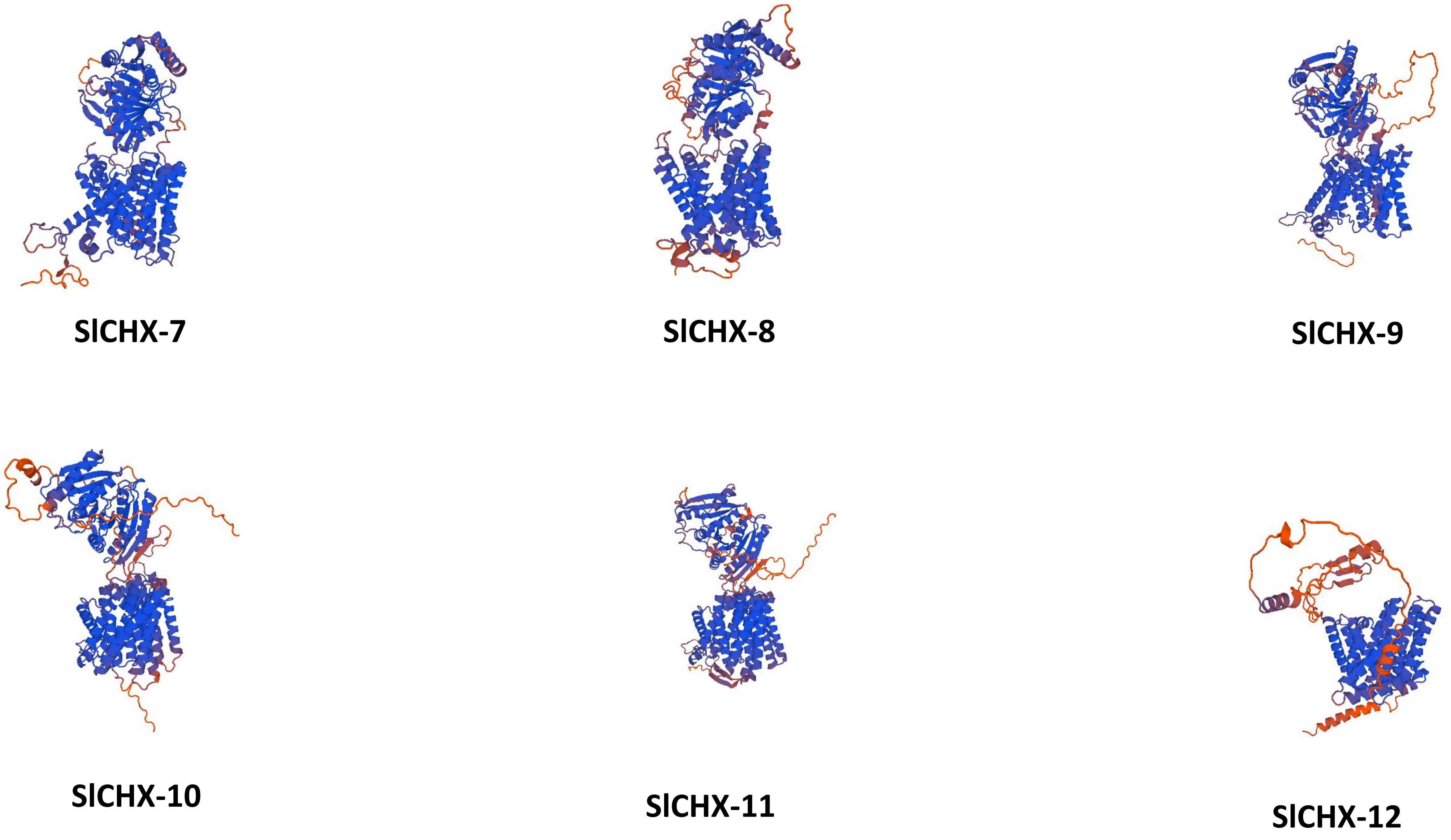

Fig. S44. Proteins modeled with swiss-model

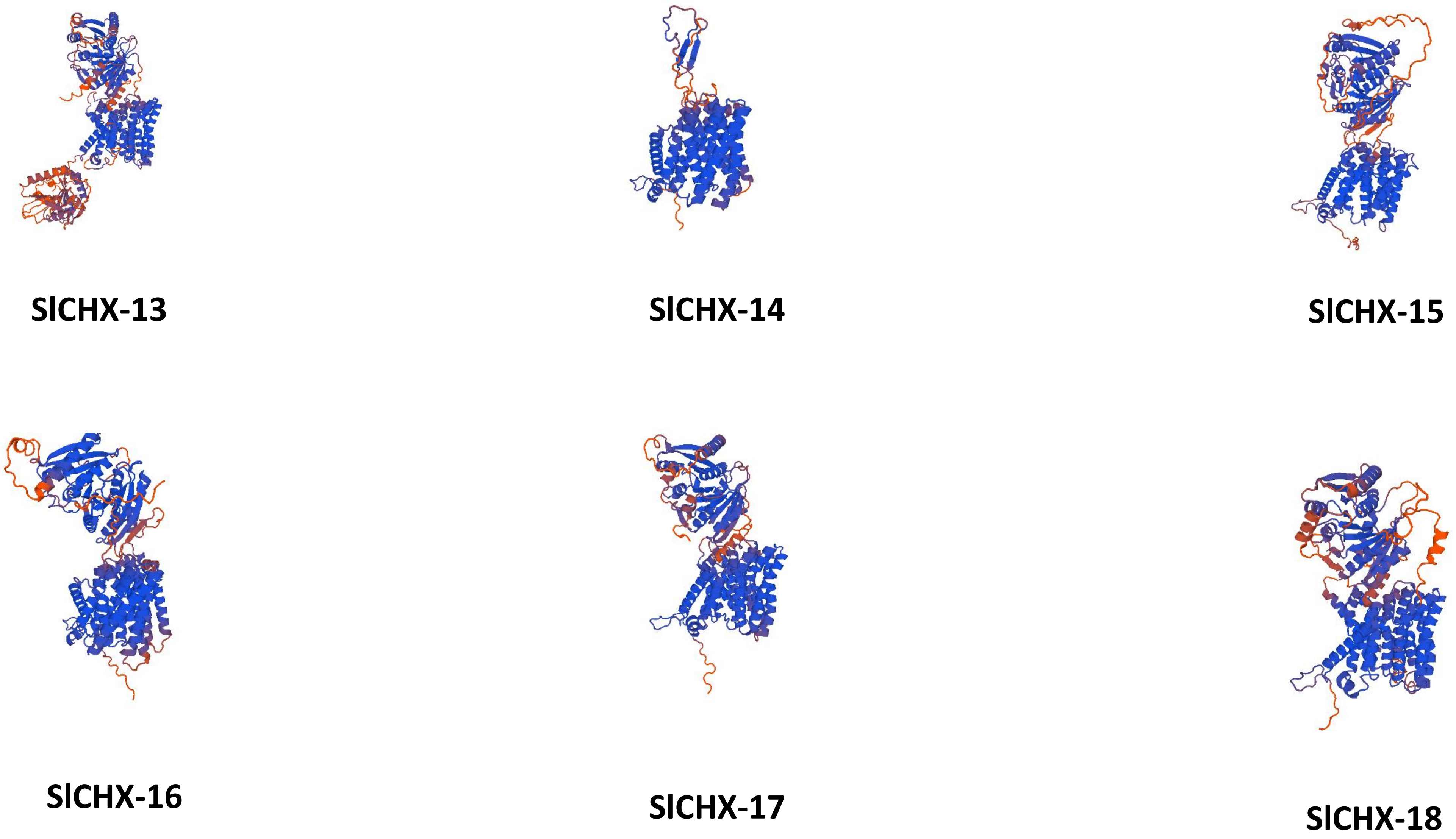

Fig. S45. Proteins modeled with swiss-model

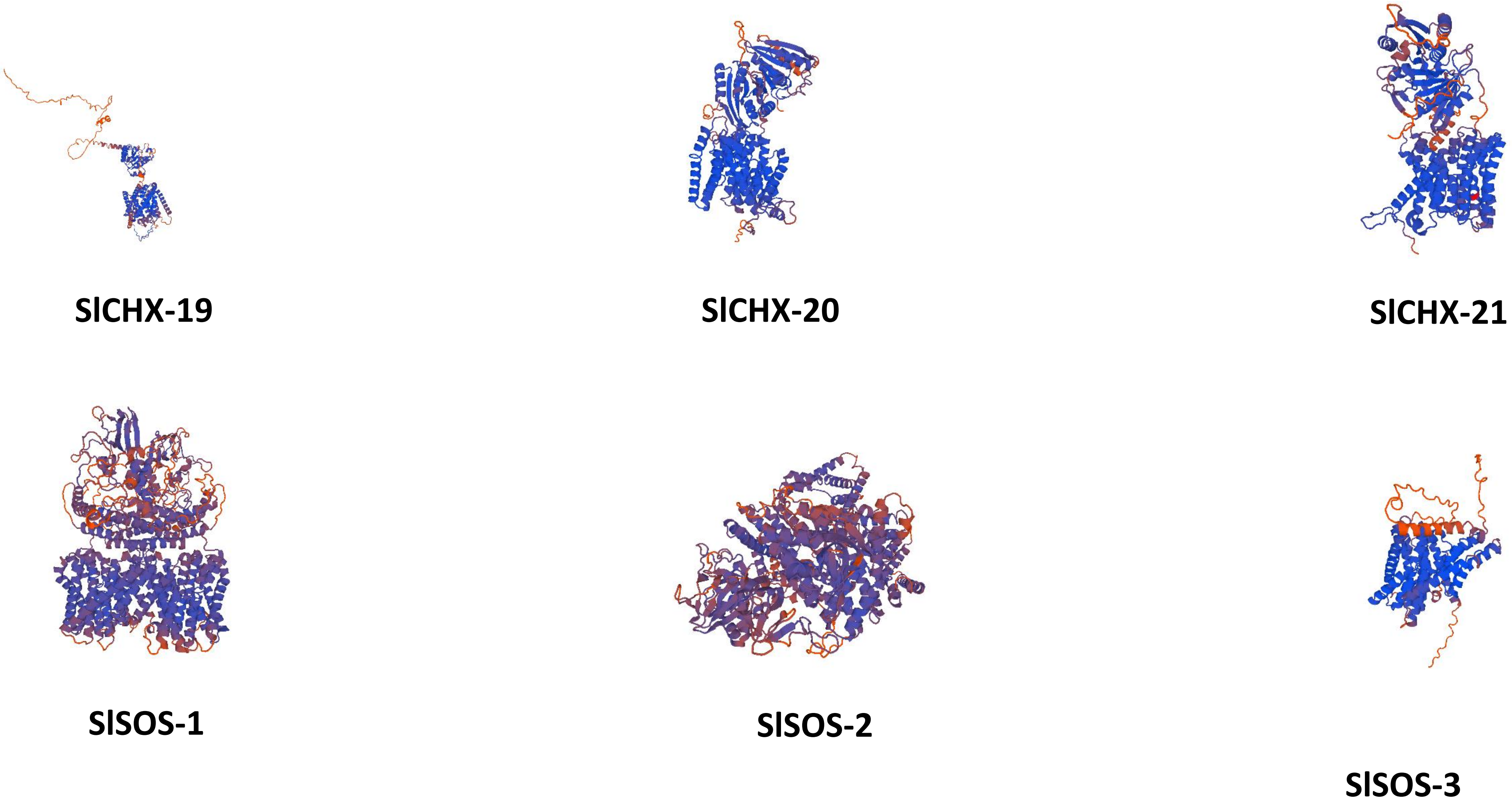

Fig. S46. Proteins modeled with swiss-model

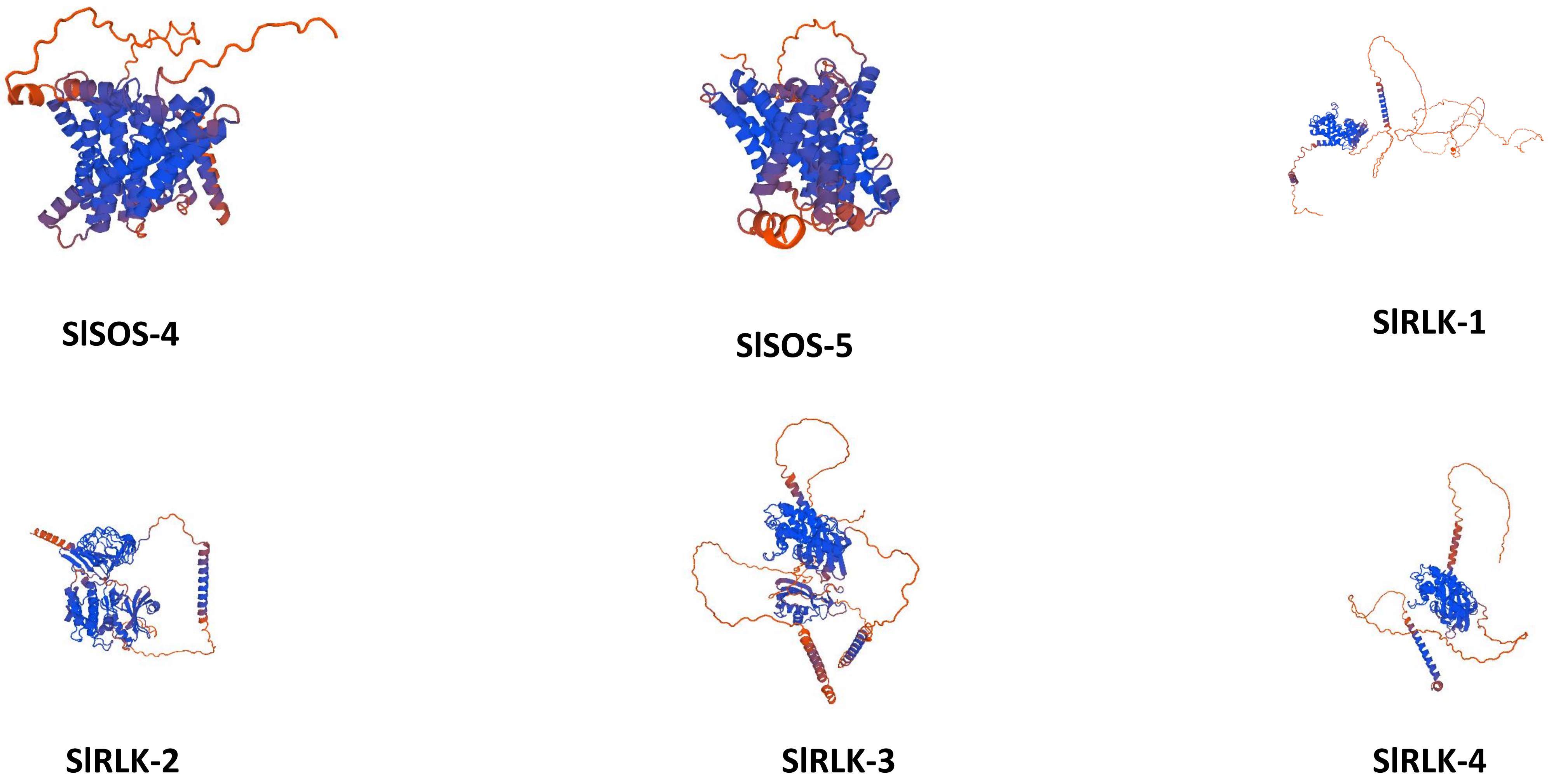

Fig. S47. Proteins modeled with swiss-model

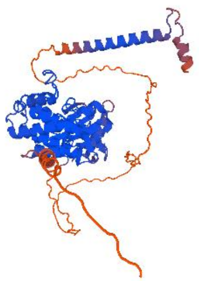

SlRLK-5

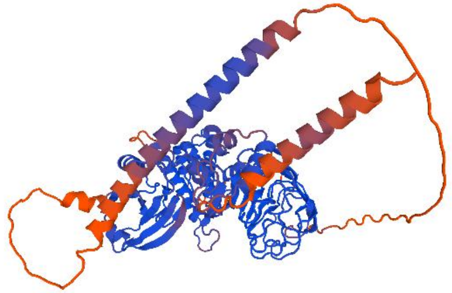

SlRLK-6

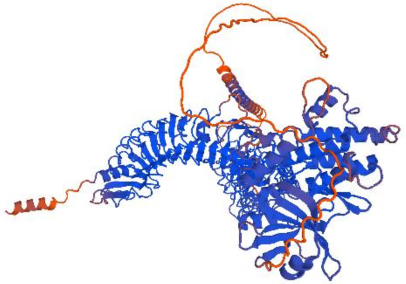

SlRLK-7

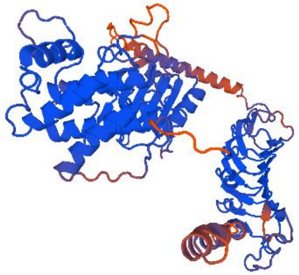

SlRLK-8

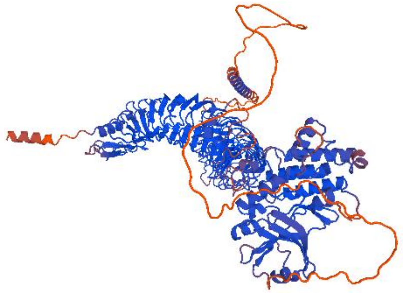

SlRLK-9

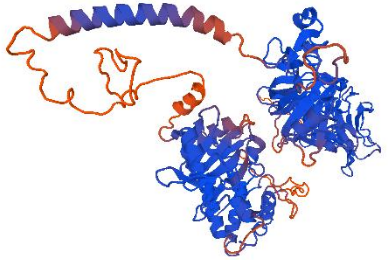

SlRLK-10

Fig. S48. Proteins modeled with swiss-model

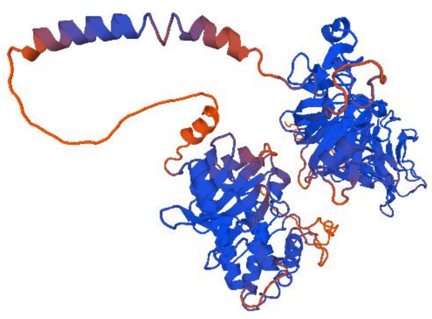

SlRLK-11

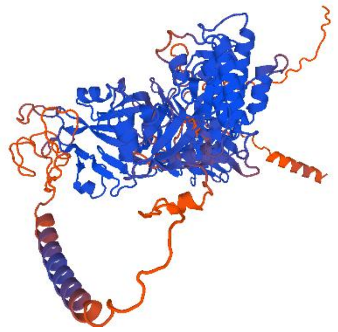

SlRLK-12

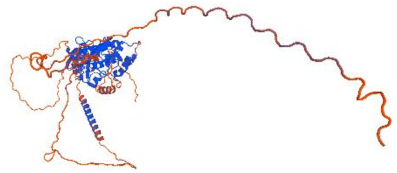

SlRLK-13

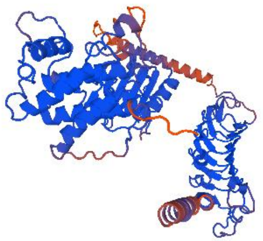

SlRLK-14

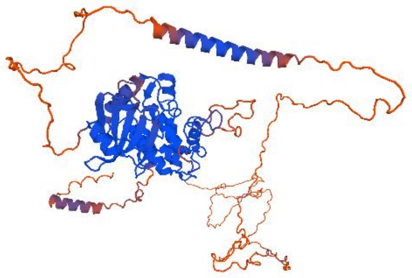

SlRLK-15

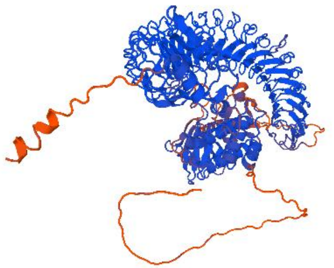

SlRLK-16

Fig. S49. Proteins modeled with swiss-model

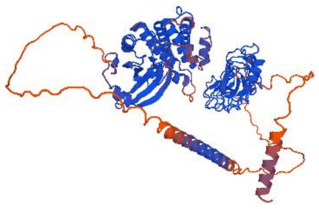

SlRLK-17

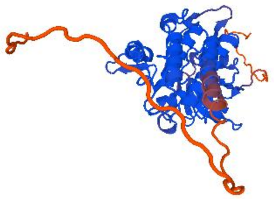

SlRLK-18

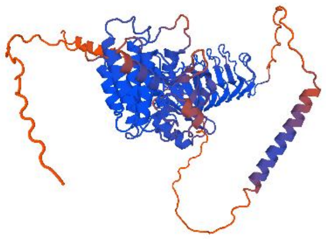

SlRLK-19

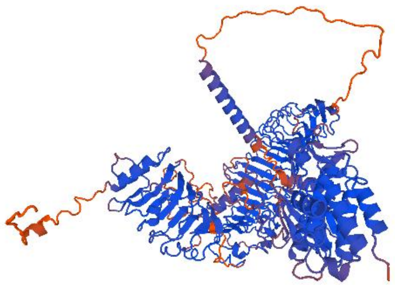

SlRLK-20

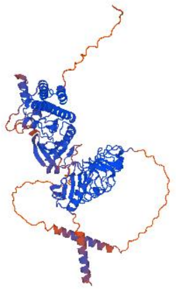

SlRLK-21

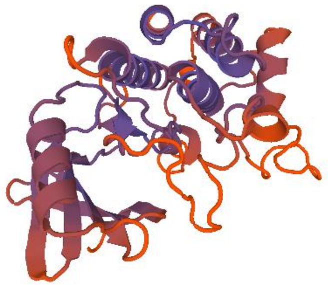

SlRLK-22

Fig. S50. Proteins modeled with swiss-model

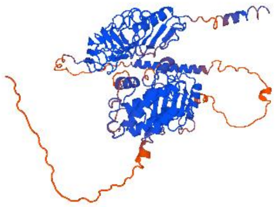

SlRLK-23

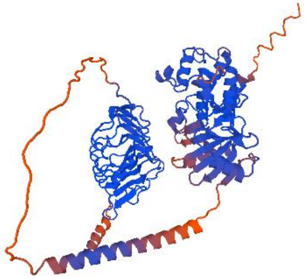

SlRLK-24

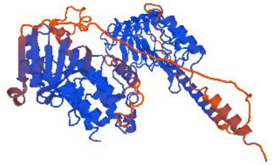

SlRLK-25

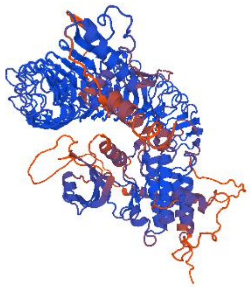

SlRLK-26

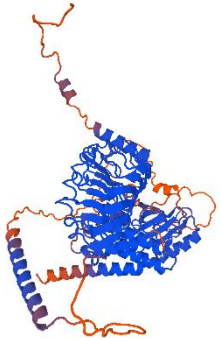

SlRLK-27

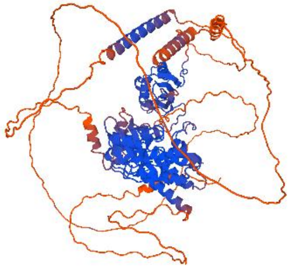

SlRLK-28

Fig. S51. Proteins modeled with swiss-model

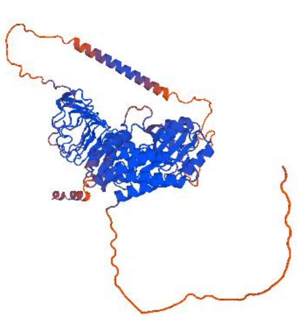

SlRLK-29

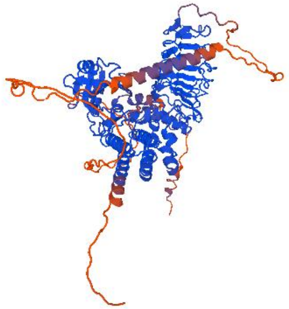

SlRLK-30

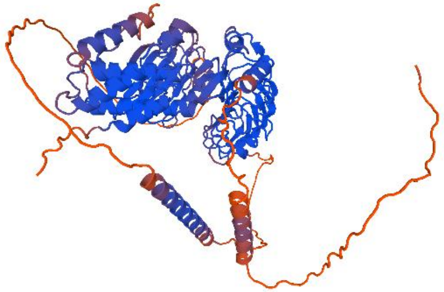

SlRLK-31

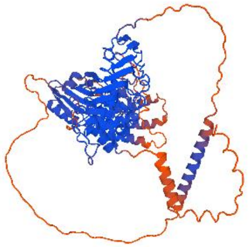

SlRLK-32

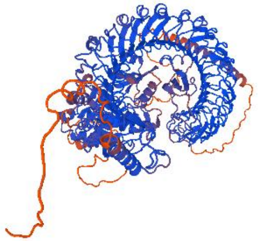

SlRLK-33

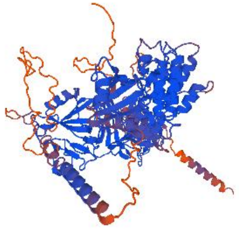

SlRLK-34

Fig. S52. Proteins modeled with swiss-model

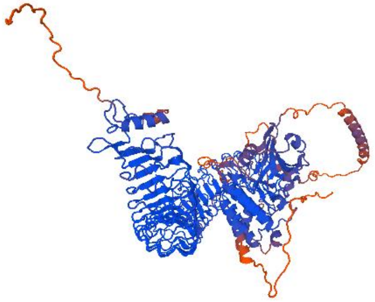

SlRLK-35

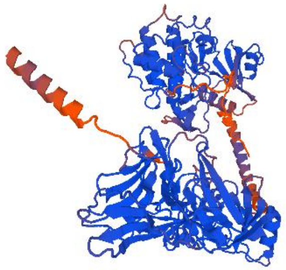

SlRLK-36

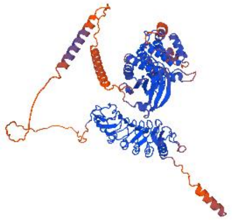

SlRLK-37

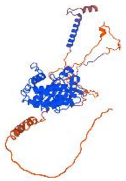

SlRLK-38

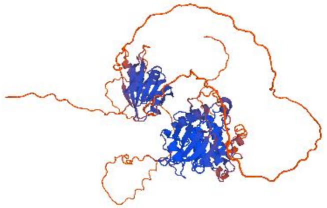

SlRLK-39

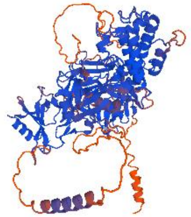

SlRLK-40

Fig. S53. Proteins modeled with swiss-model

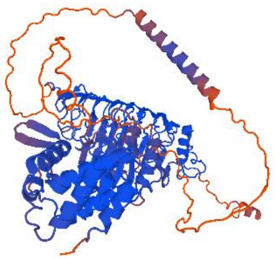

SlRLK-41

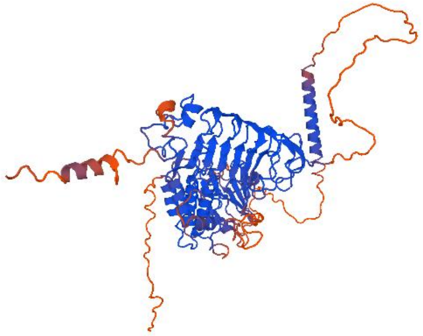

SlRLK-42

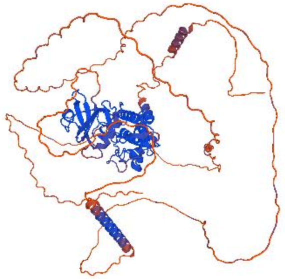

SlRLK-43

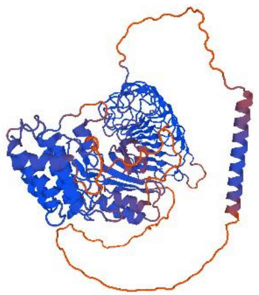

SlRLK-44

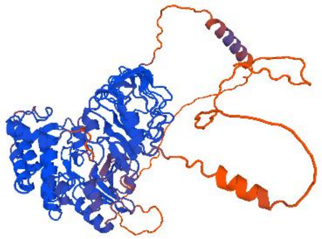

SlRLK-45

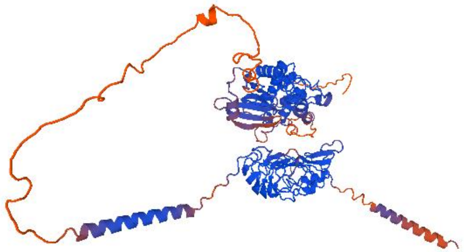

SlRLK-46

Fig. S54. Proteins modeled with swiss-model

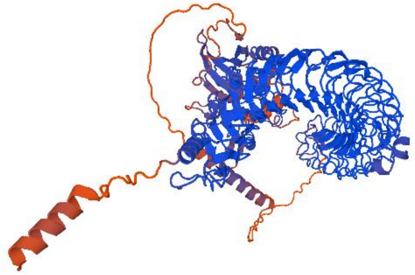

SlRLK-47

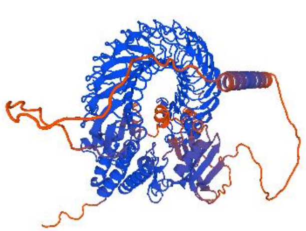

SlRLK-48

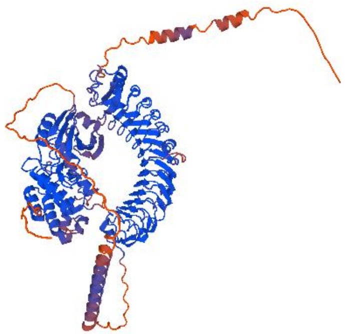

SlRLK-49

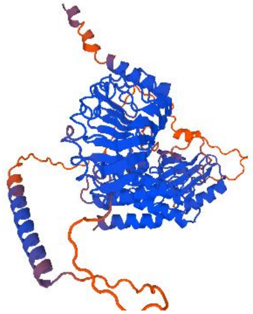

SlRLK-50

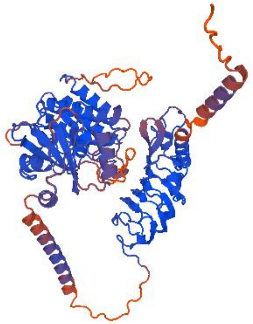

SlRLK-51

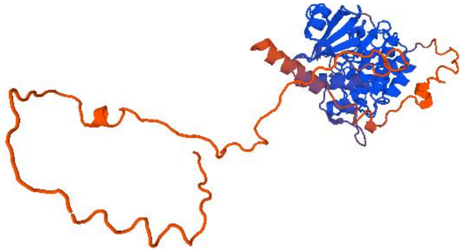

SlRLK-52

Fig. S55. Proteins modeled with swiss-model

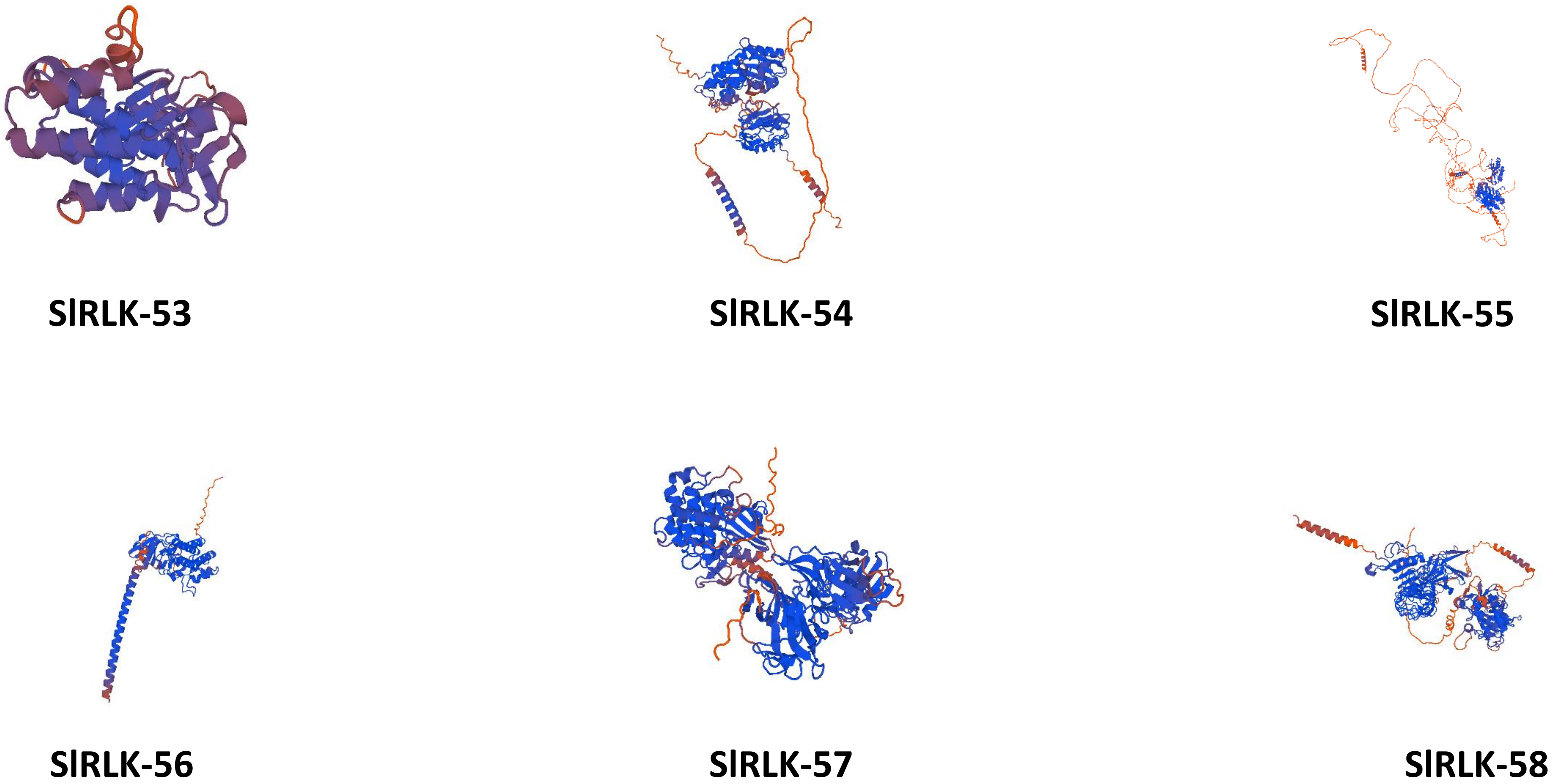

Fig. S56. Proteins modeled with swiss-model

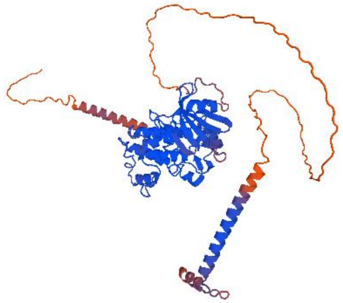

SlRLK-59

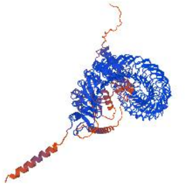

SlRLK-60

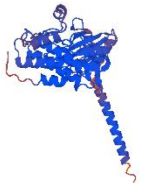

SlRLK-61

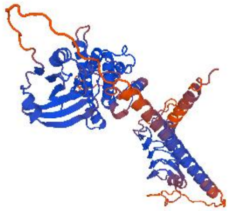

SlRLK-62

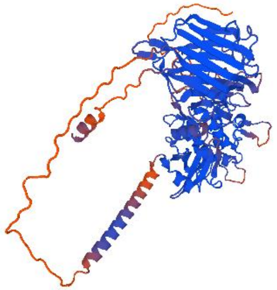

SlRLK-63

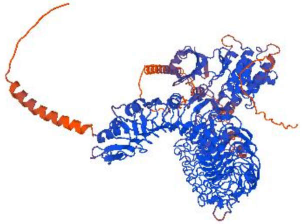

SlRLK-64

Fig. S57. Proteins modeled with swiss-model

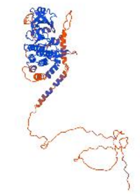

SlRLK-65

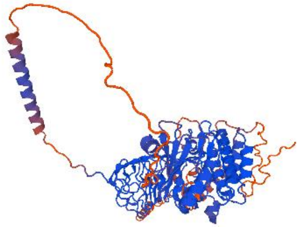

SlRLK-66

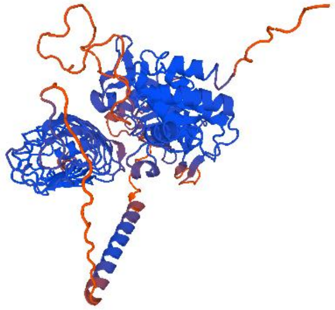

SlRLK-67

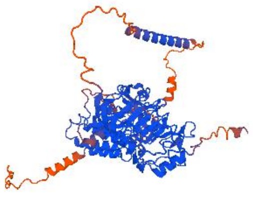

SlRLK-68

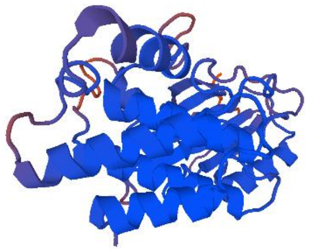

SlRLK-69

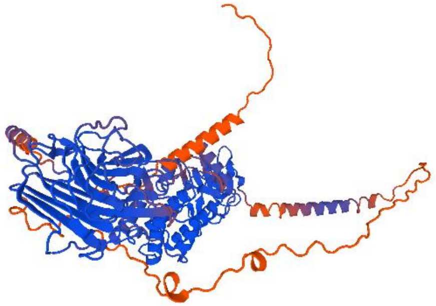

SlRLK-70

Fig. S58. Proteins modeled with swiss-model

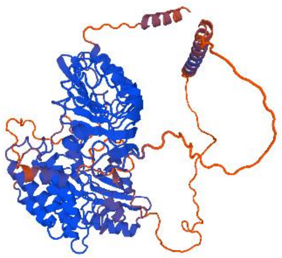

SlRLK-71

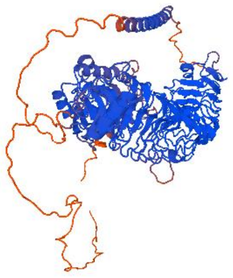

SlRLK-72

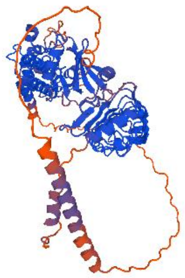

SlRLK-73

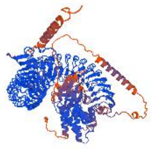

SlRLK-74

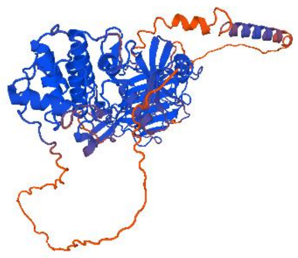

SlRLK-75

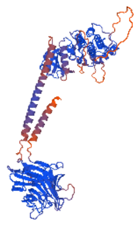

SlRLK-76

Fig. S59. Proteins modeled with swiss-model

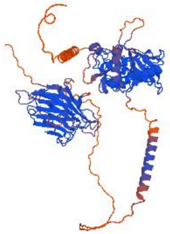

SlRLK-77

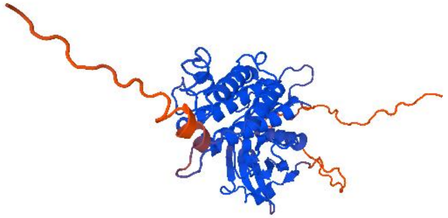

SlRLK-78

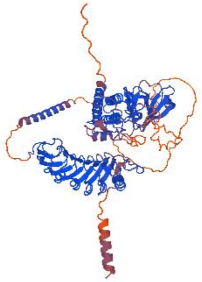

SlRLK-79

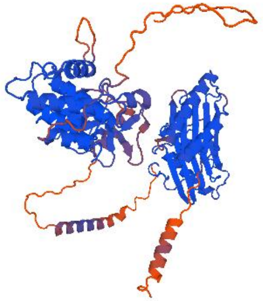

SlRLK-80

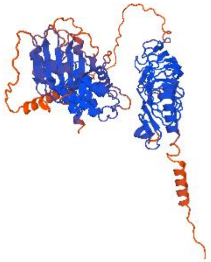

SlRLK-81

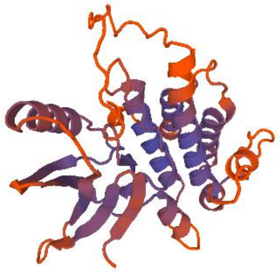

SlRLK-82

Fig. S60. Proteins modeled with swiss-model

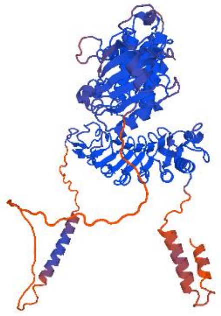

SlRLK-83

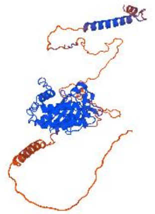

SlRLK-84

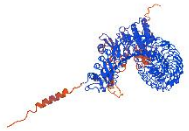

SlRLK-85

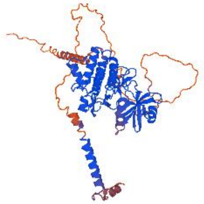

SlRLK-86

Fig. S61. Proteins modeled with swiss-model

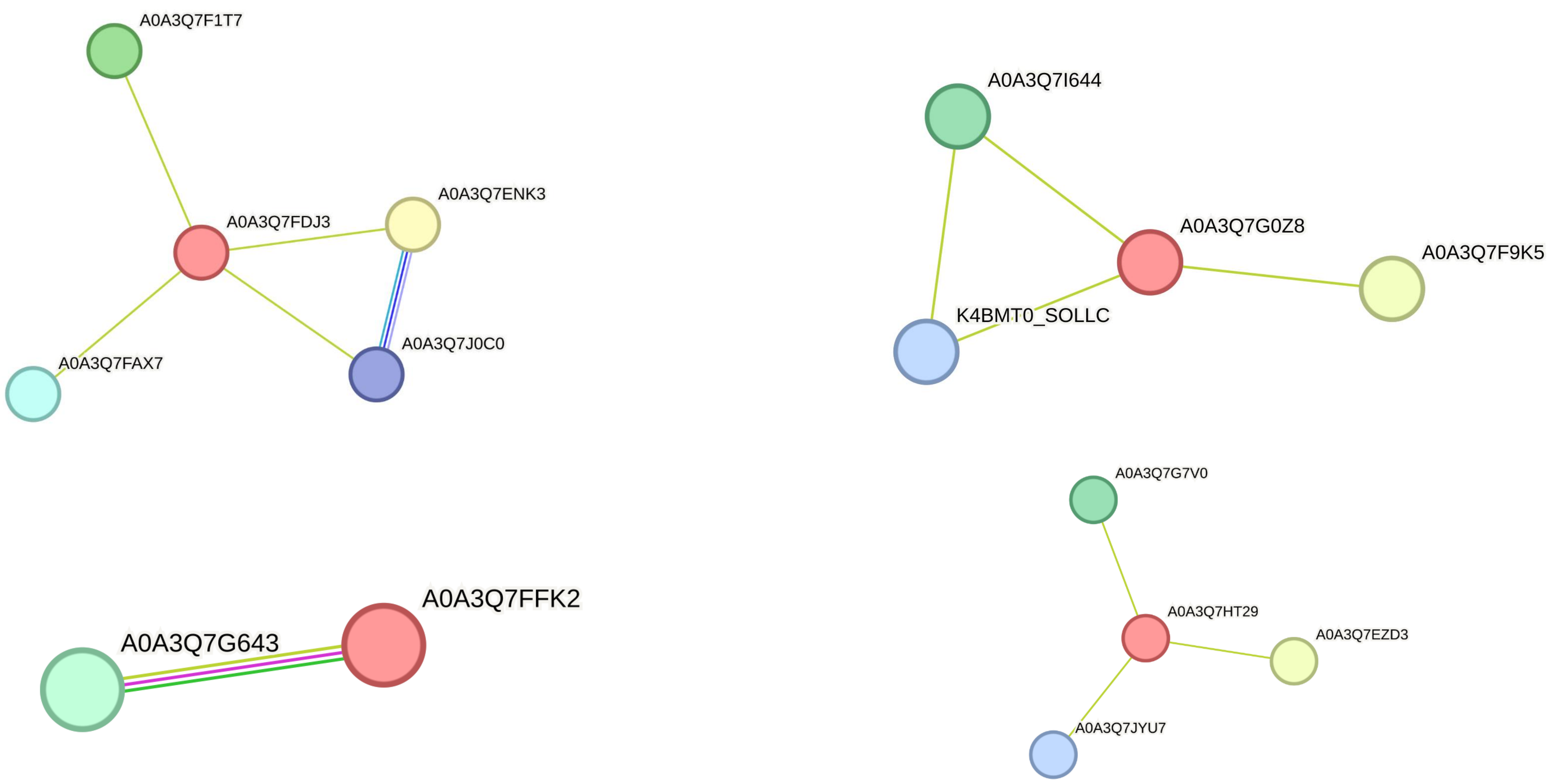

Fig. S62. CHX protein-protein interaction (PPI) network

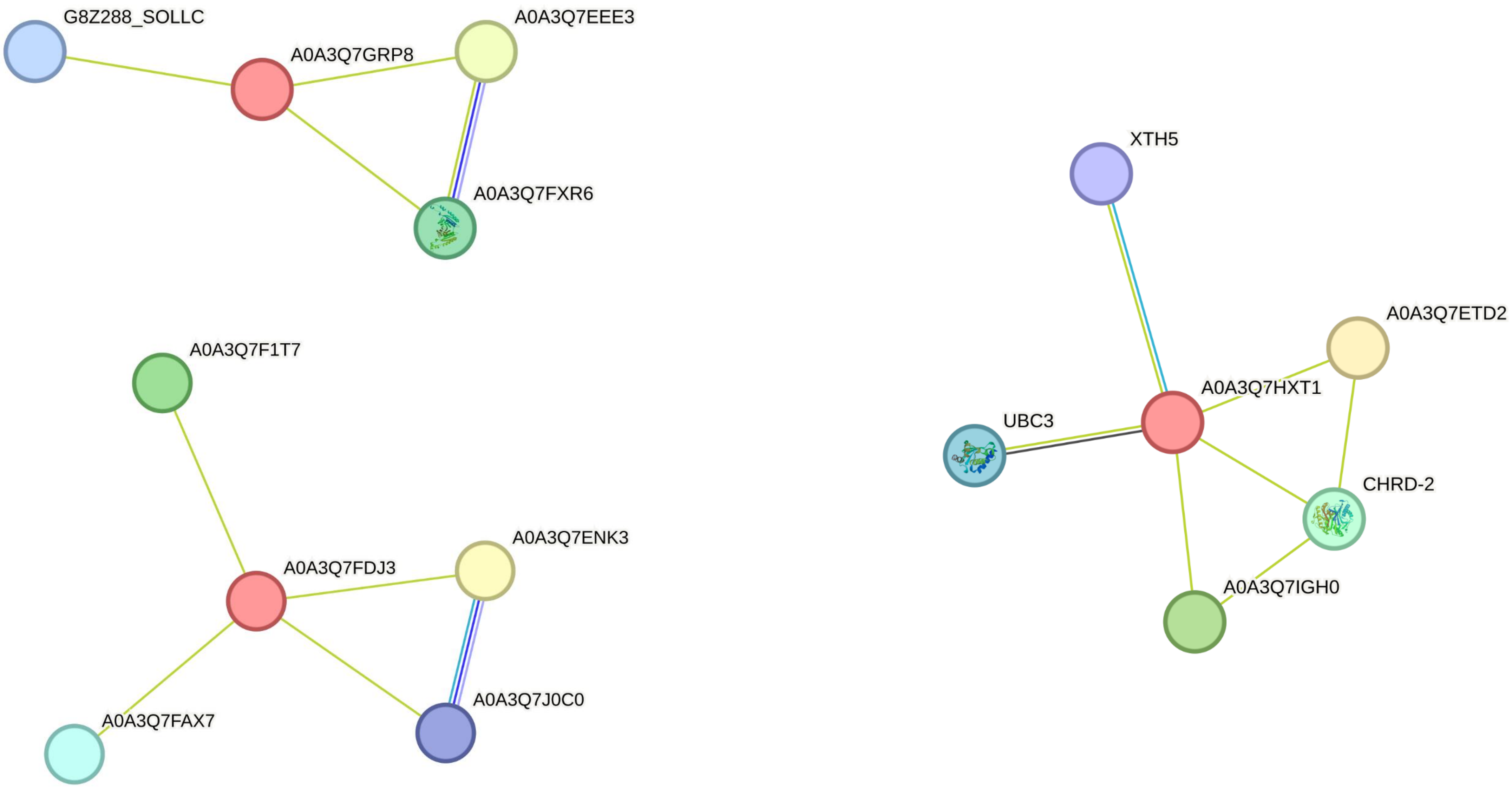

Fig. S63. CHX protein-protein interaction (PPI) network

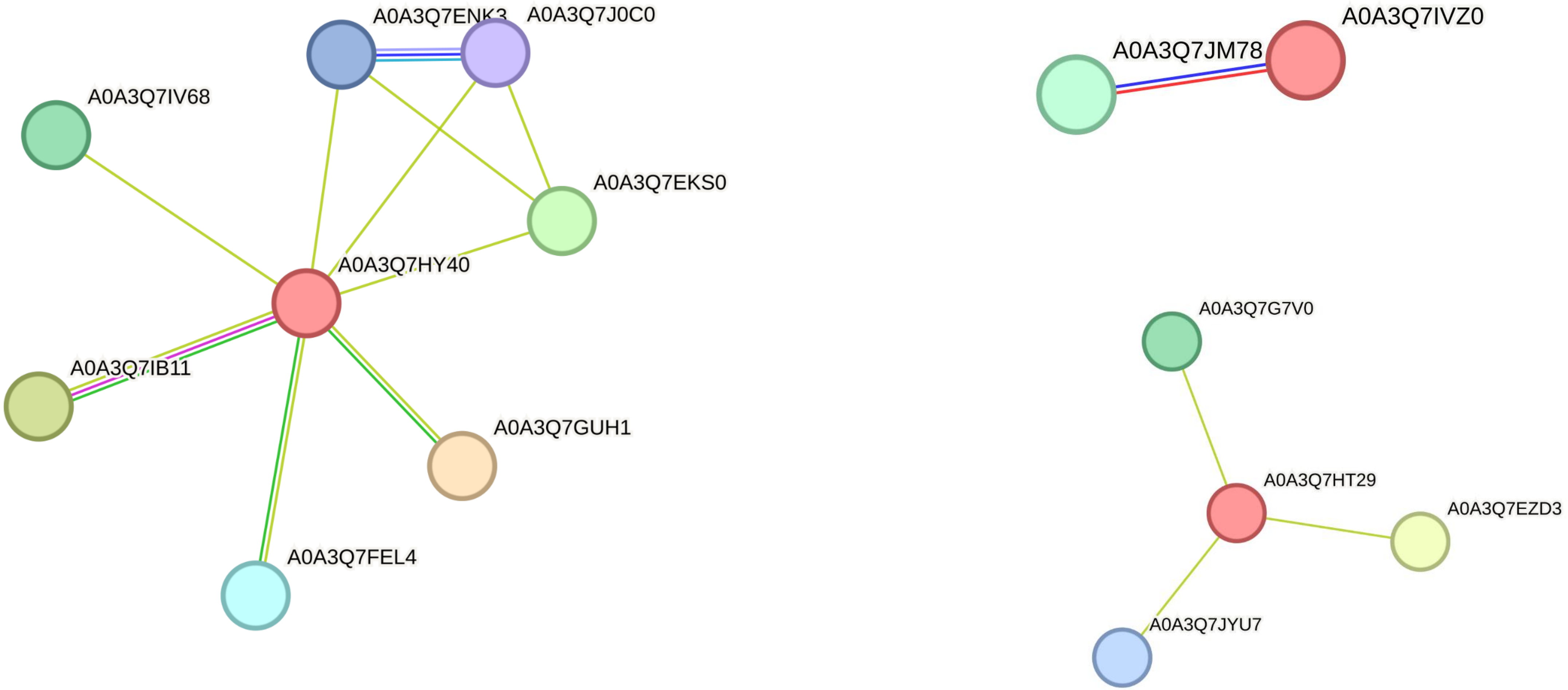

Fig. S64. CHX protein-protein interaction (PPI) network

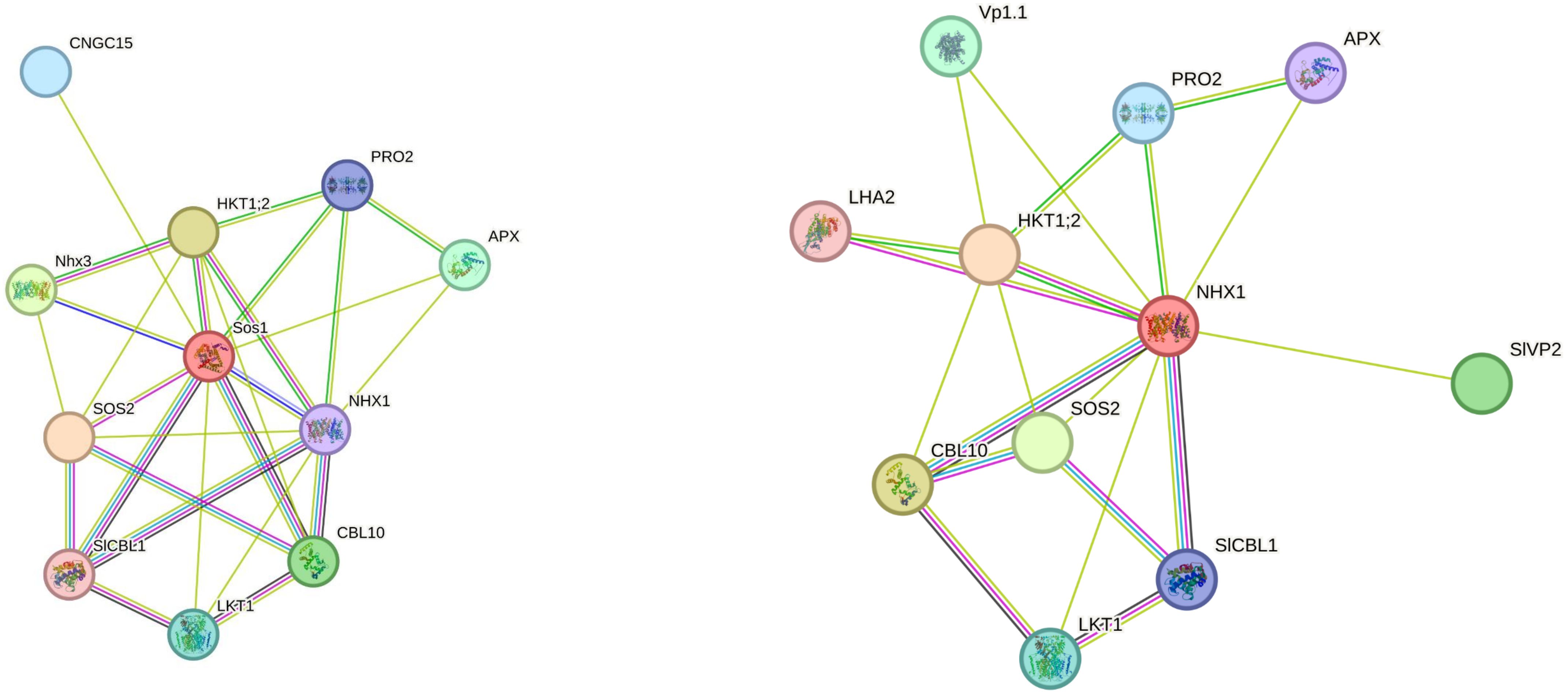

Fig. S65. SOS protein-protein interaction (PPI) network

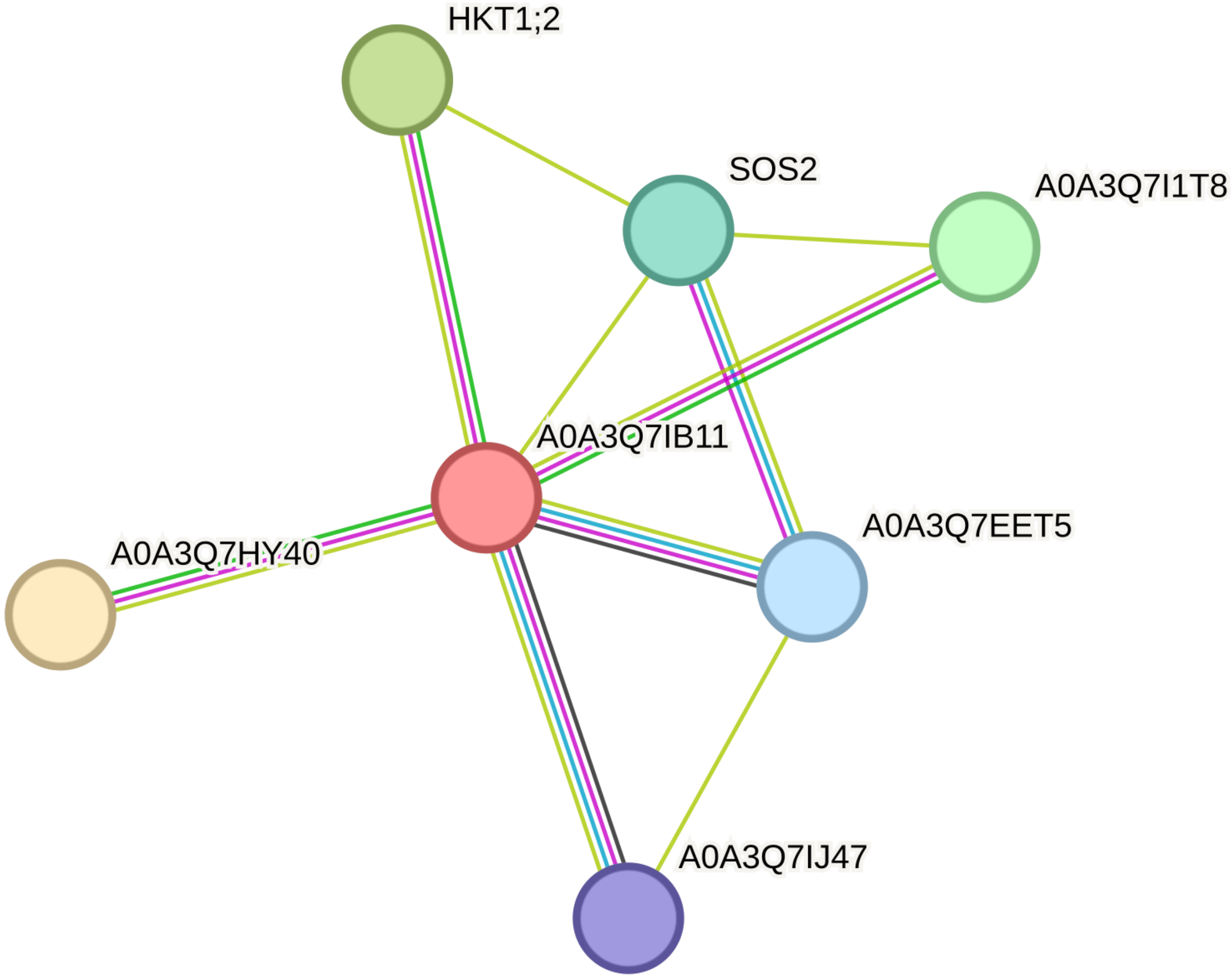

Fig. S66. SOS protein-protein interaction (PPI) network

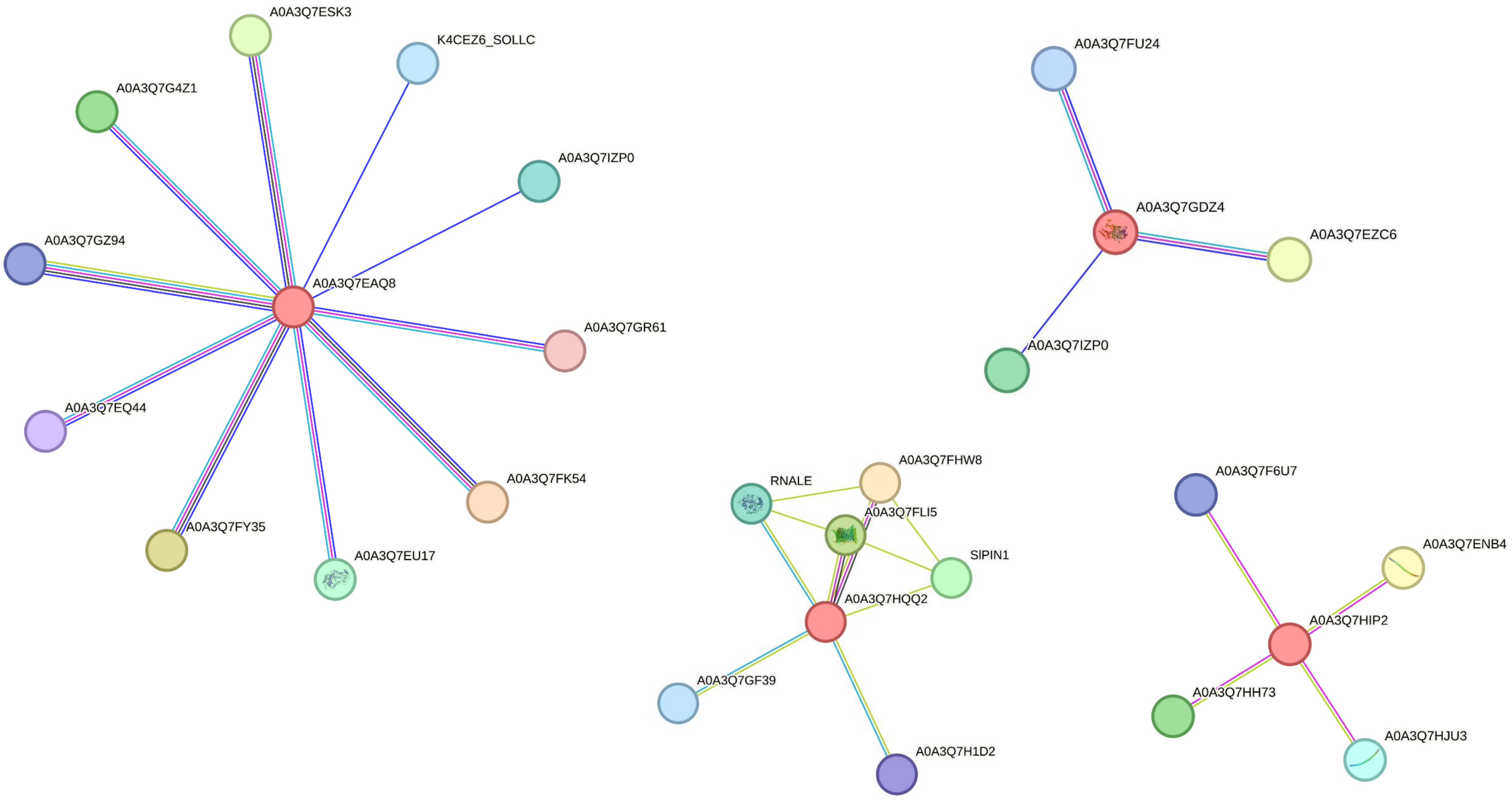

Fig. S67. RLK protein-protein interaction (PPI) network

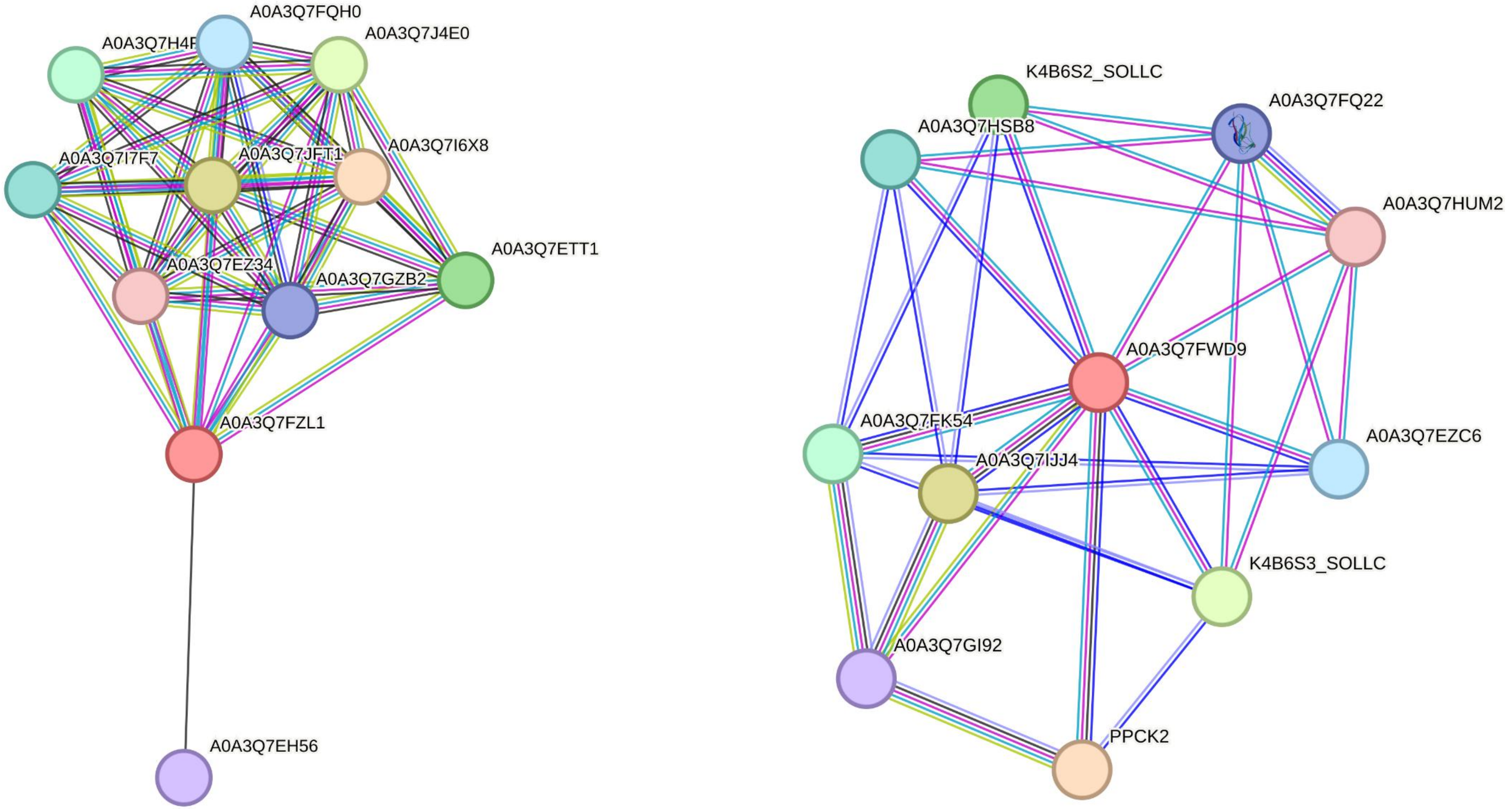

Fig. S68. RLK protein-protein interaction (PPI) network

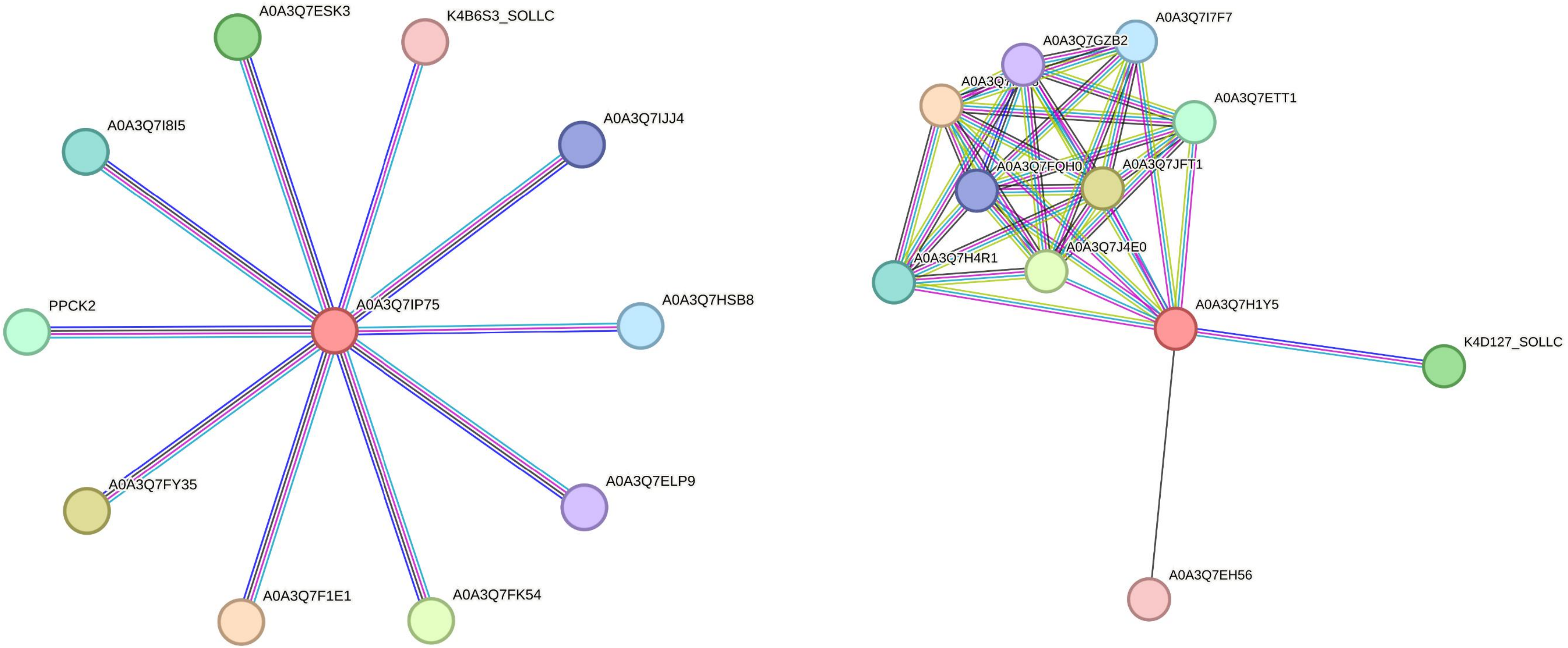

Fig. S69. RLK protein-protein interaction (PPI) network

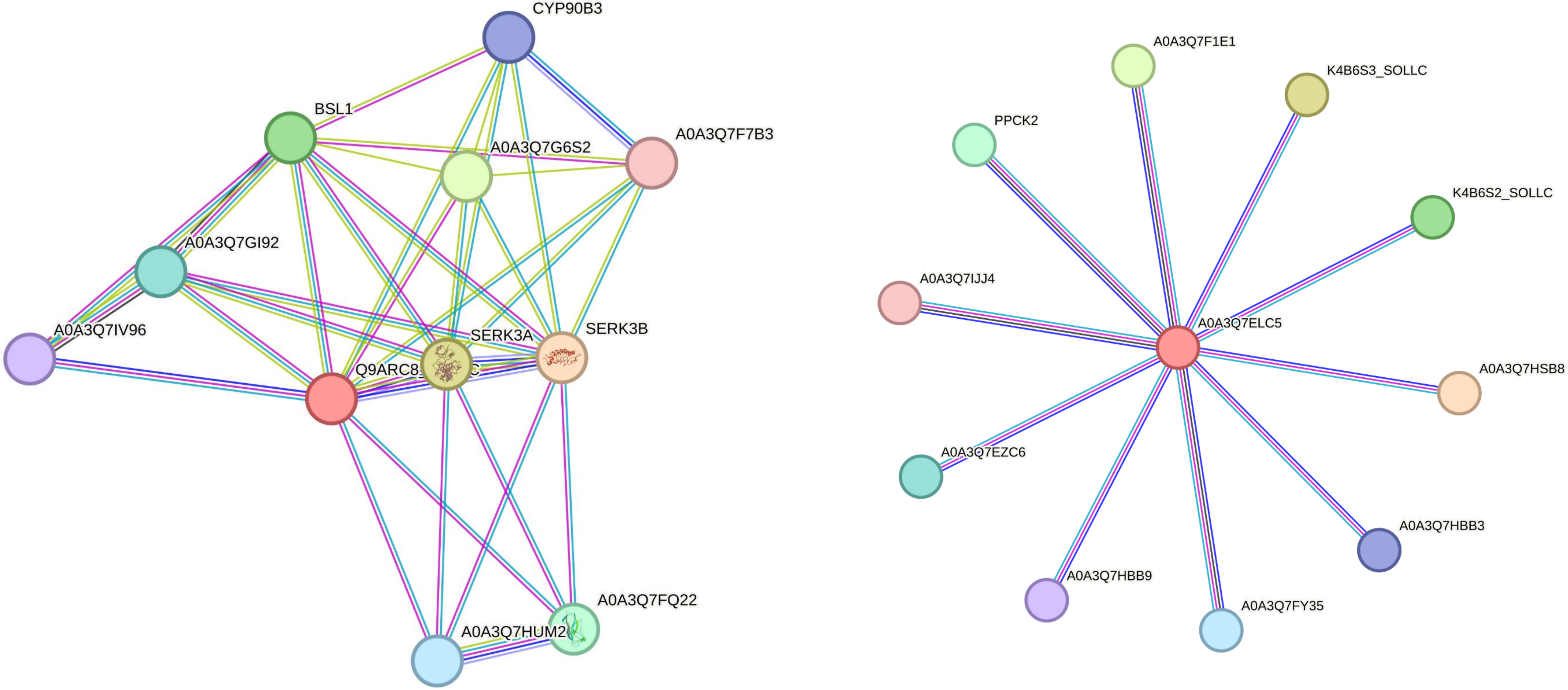

Fig. S70. RLK protein-protein interaction (PPI) network

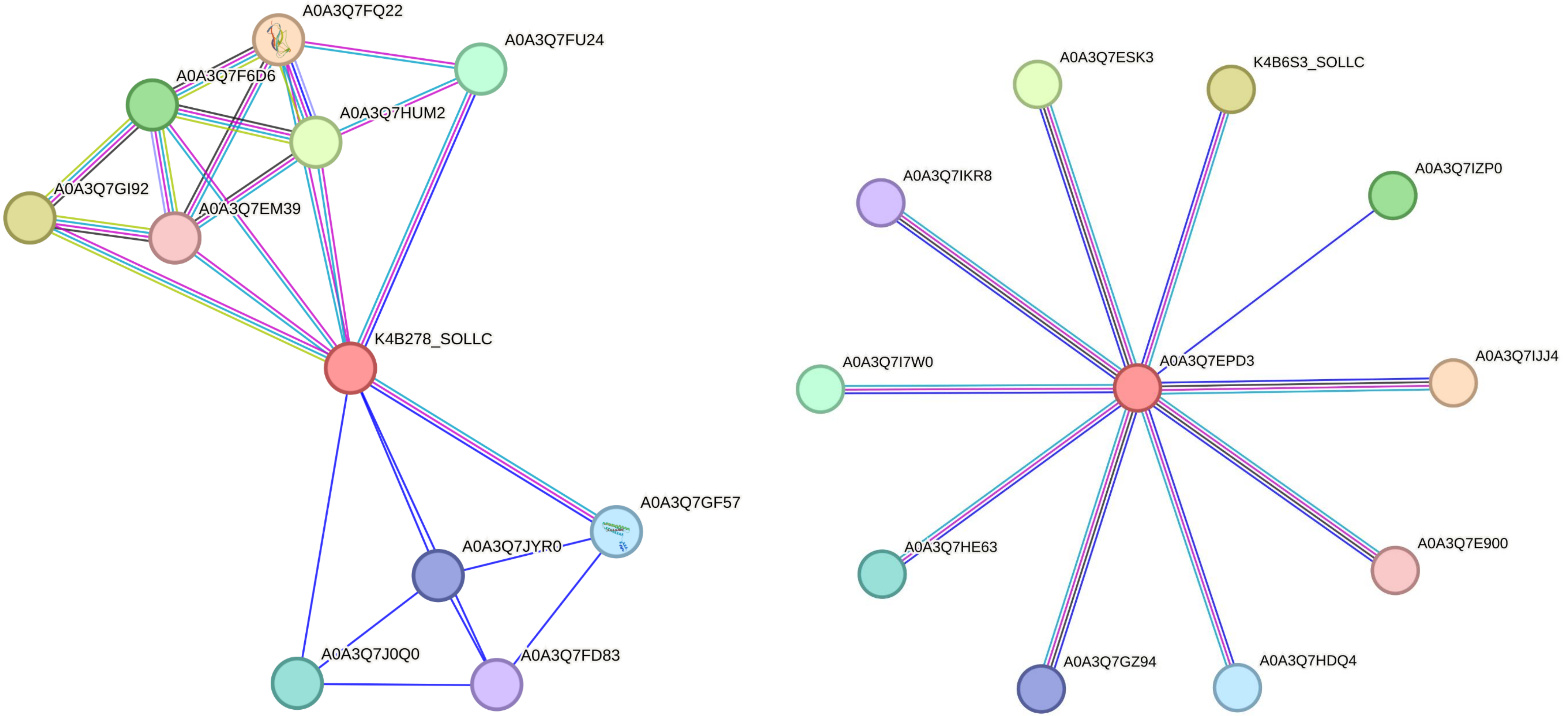

Fig. S71. RLK protein-protein interaction (PPI) network

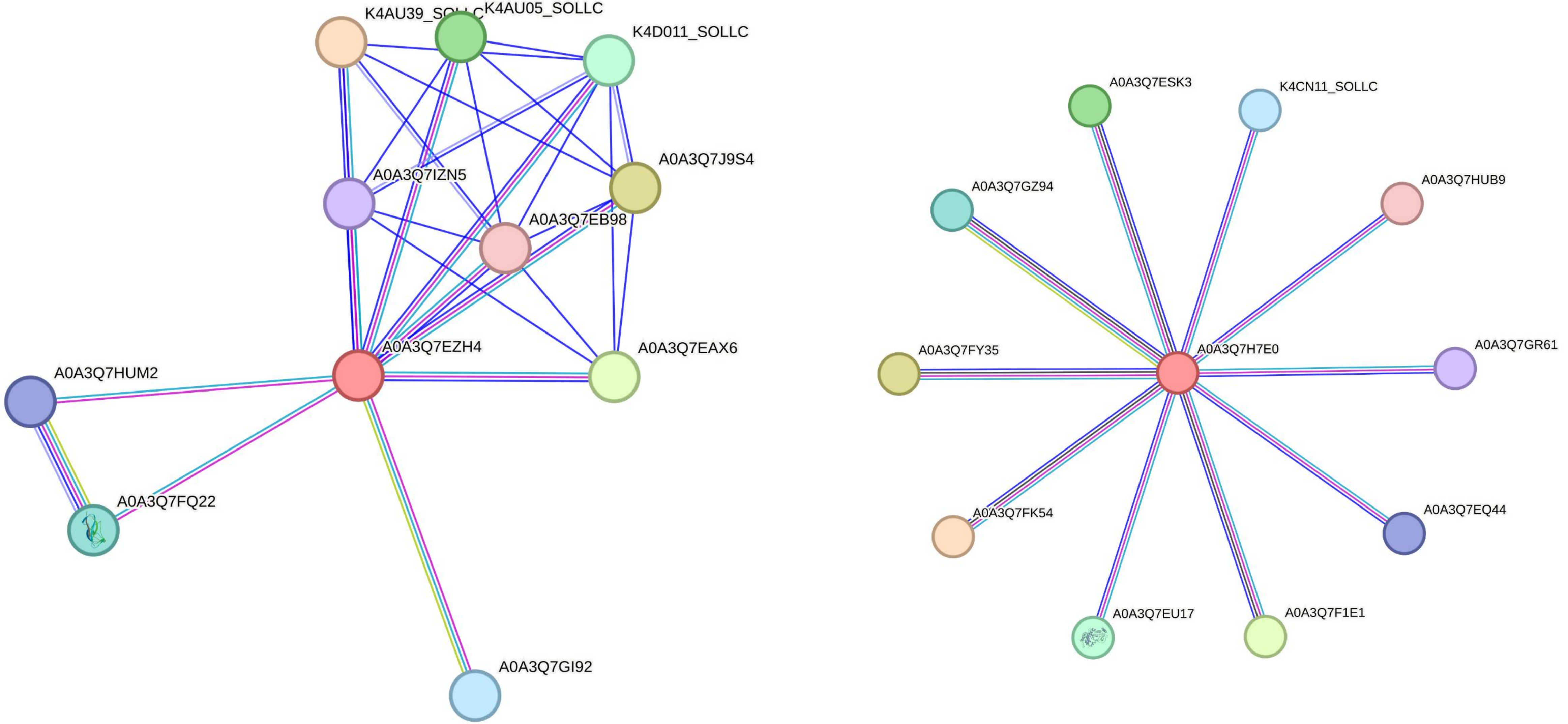

Fig. S72. RLK protein-protein interaction (PPI) network

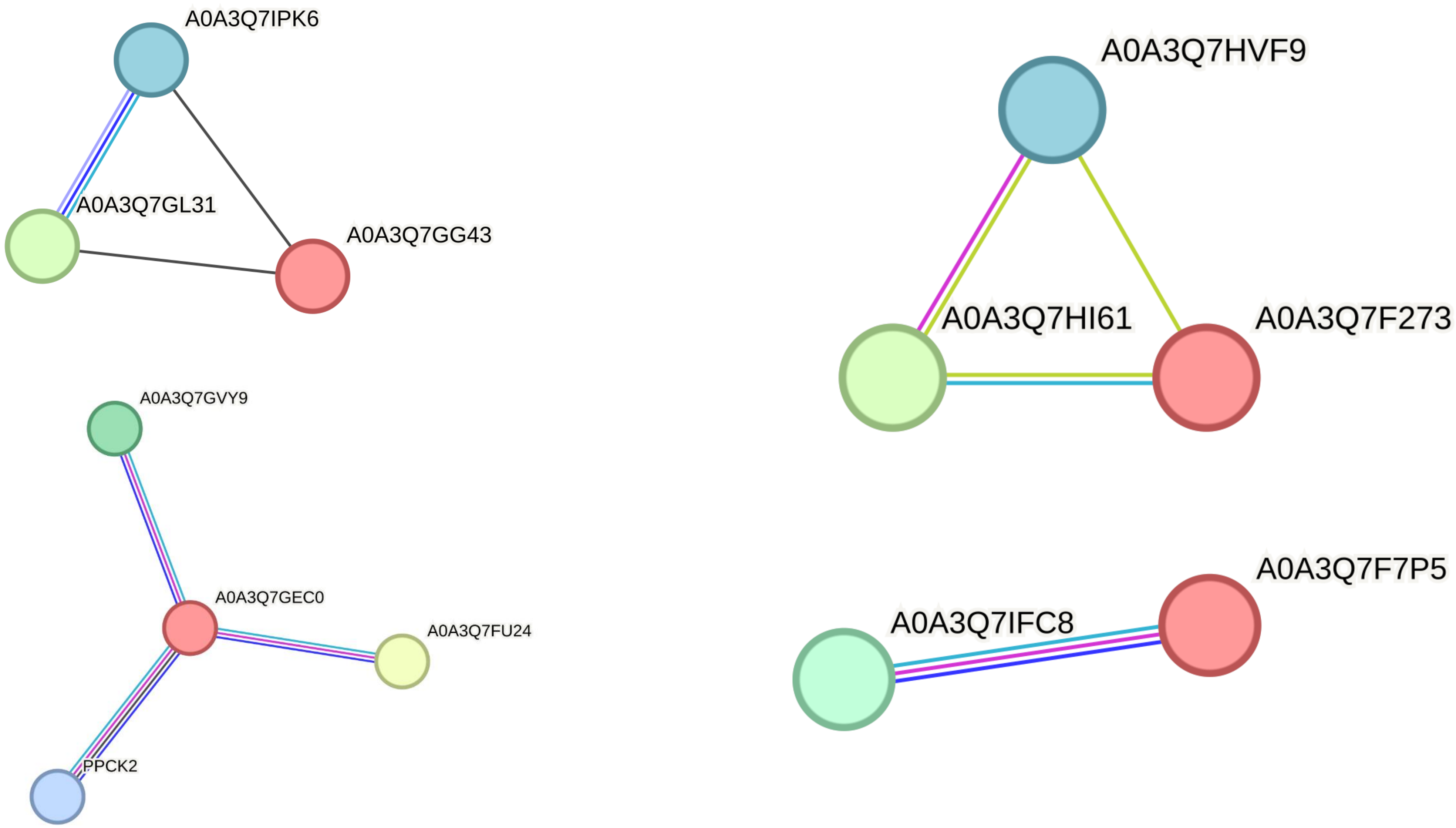

Fig. S73. RLK protein-protein interaction (PPI) network

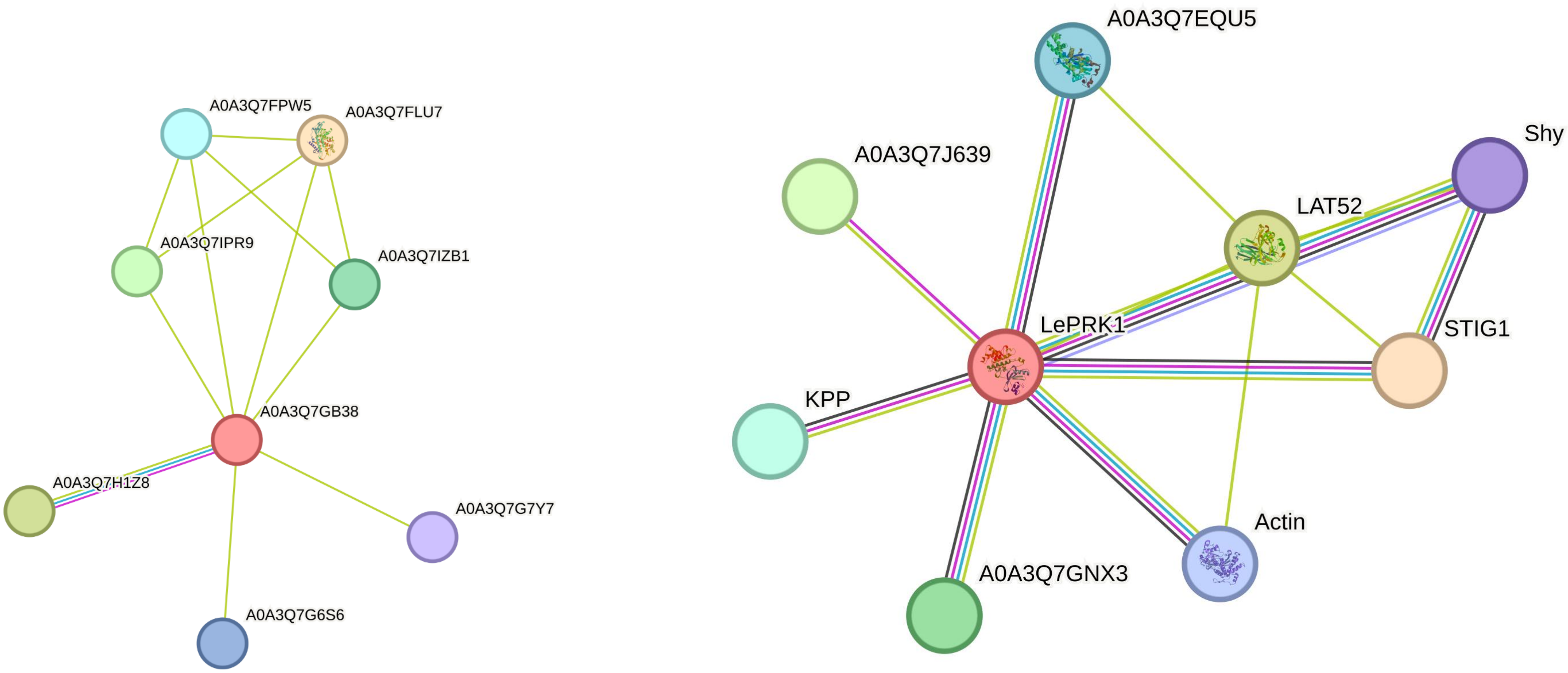

Fig. S74. RLK protein-protein interaction (PPI) network

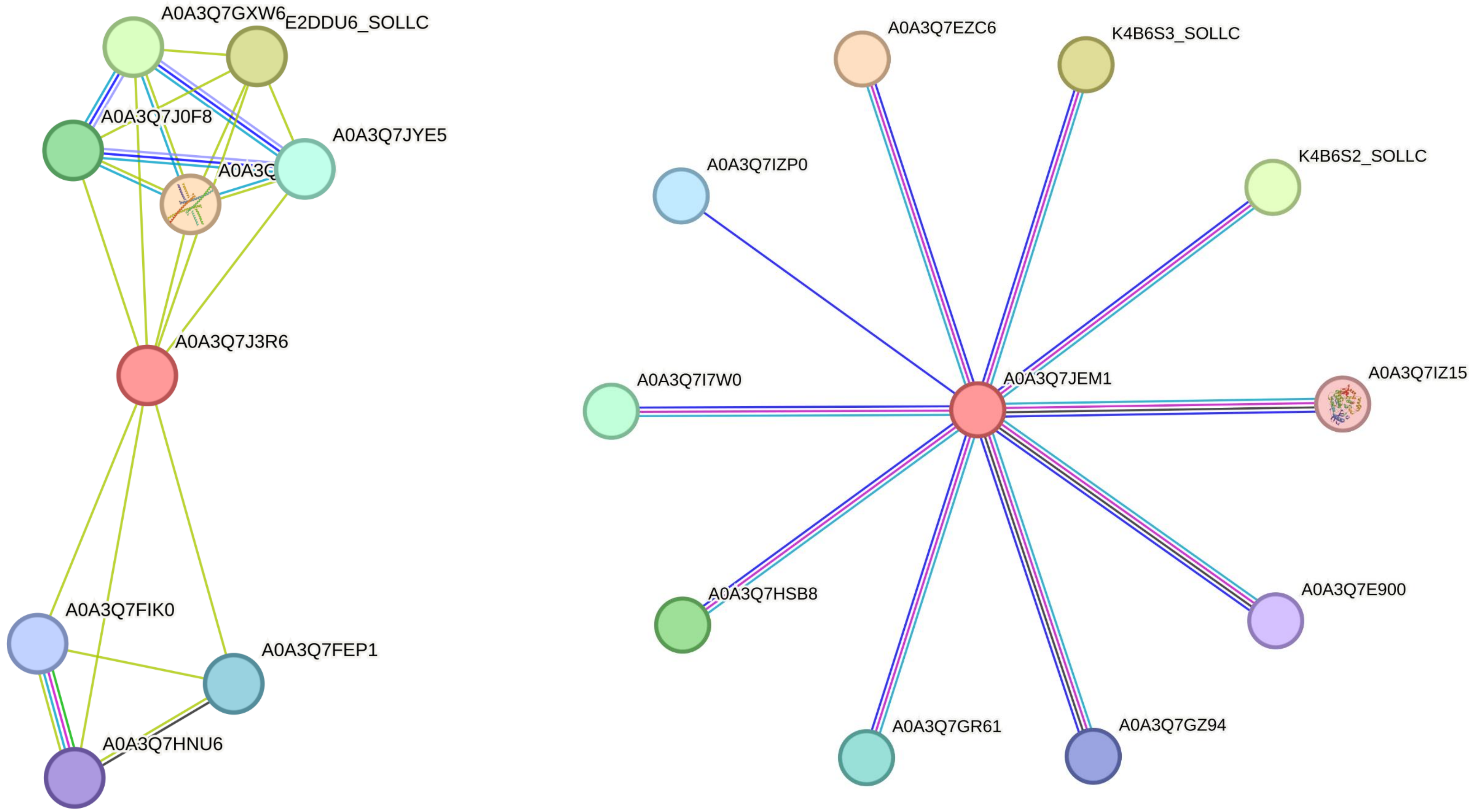

Fig. S75. RLK protein-protein interaction (PPI) network

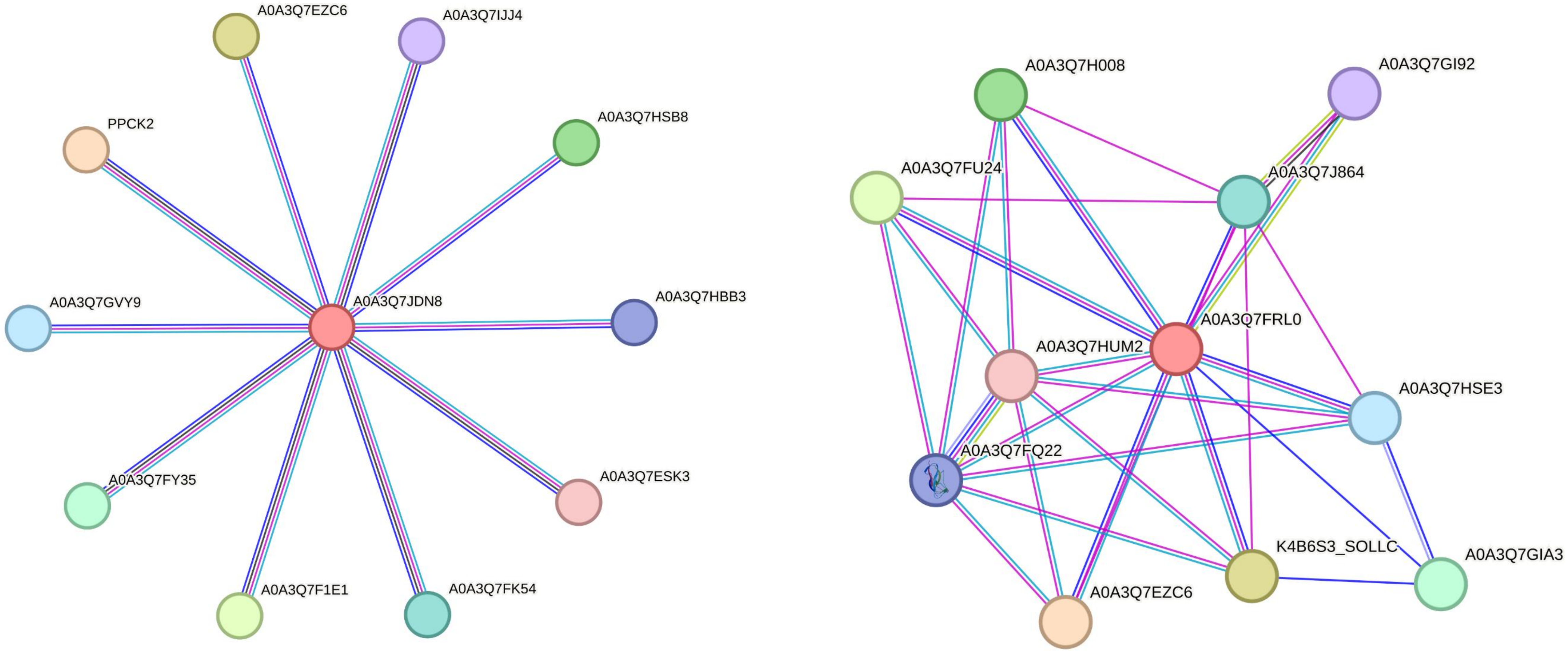

Fig. S76. RLK protein-protein interaction (PPI) network

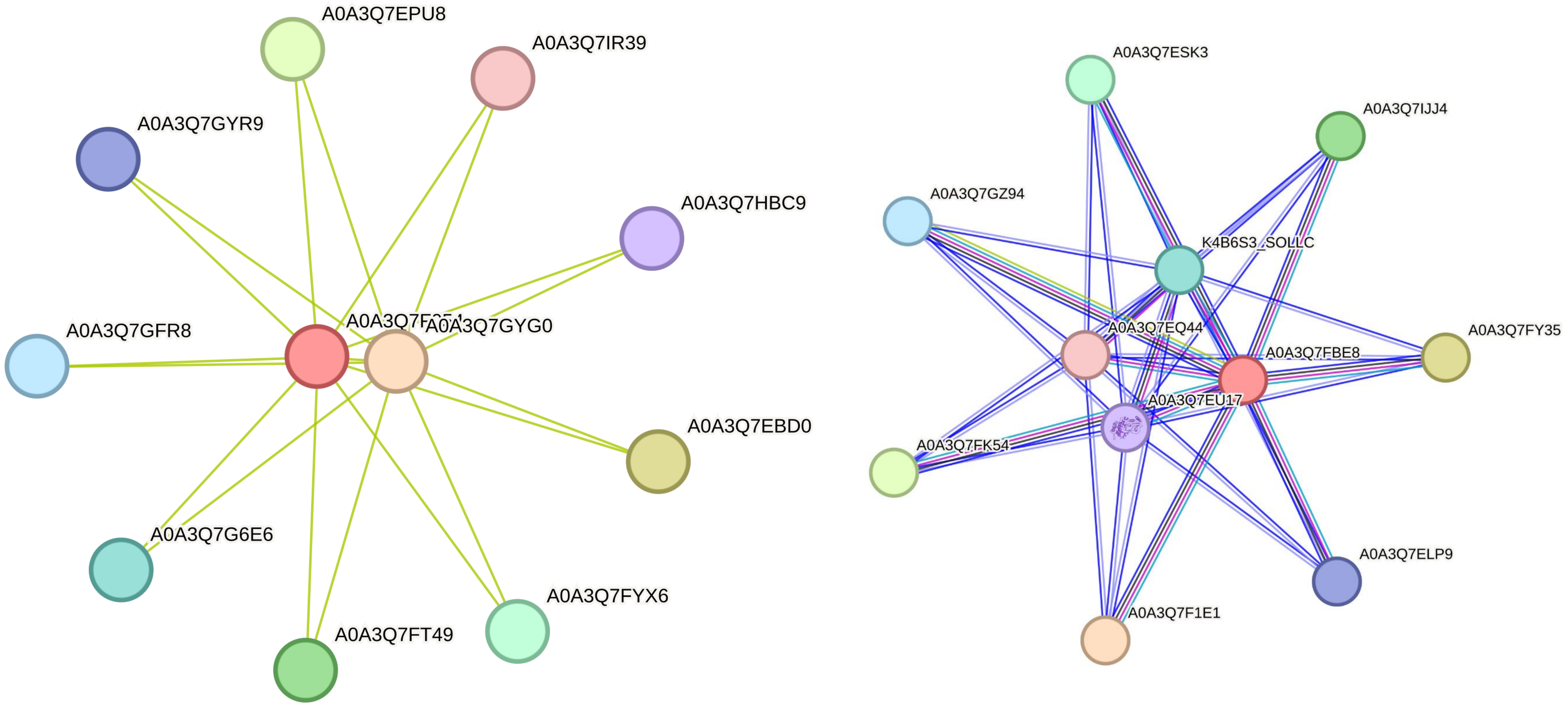

Fig. S77. RLK protein-protein interaction (PPI) network

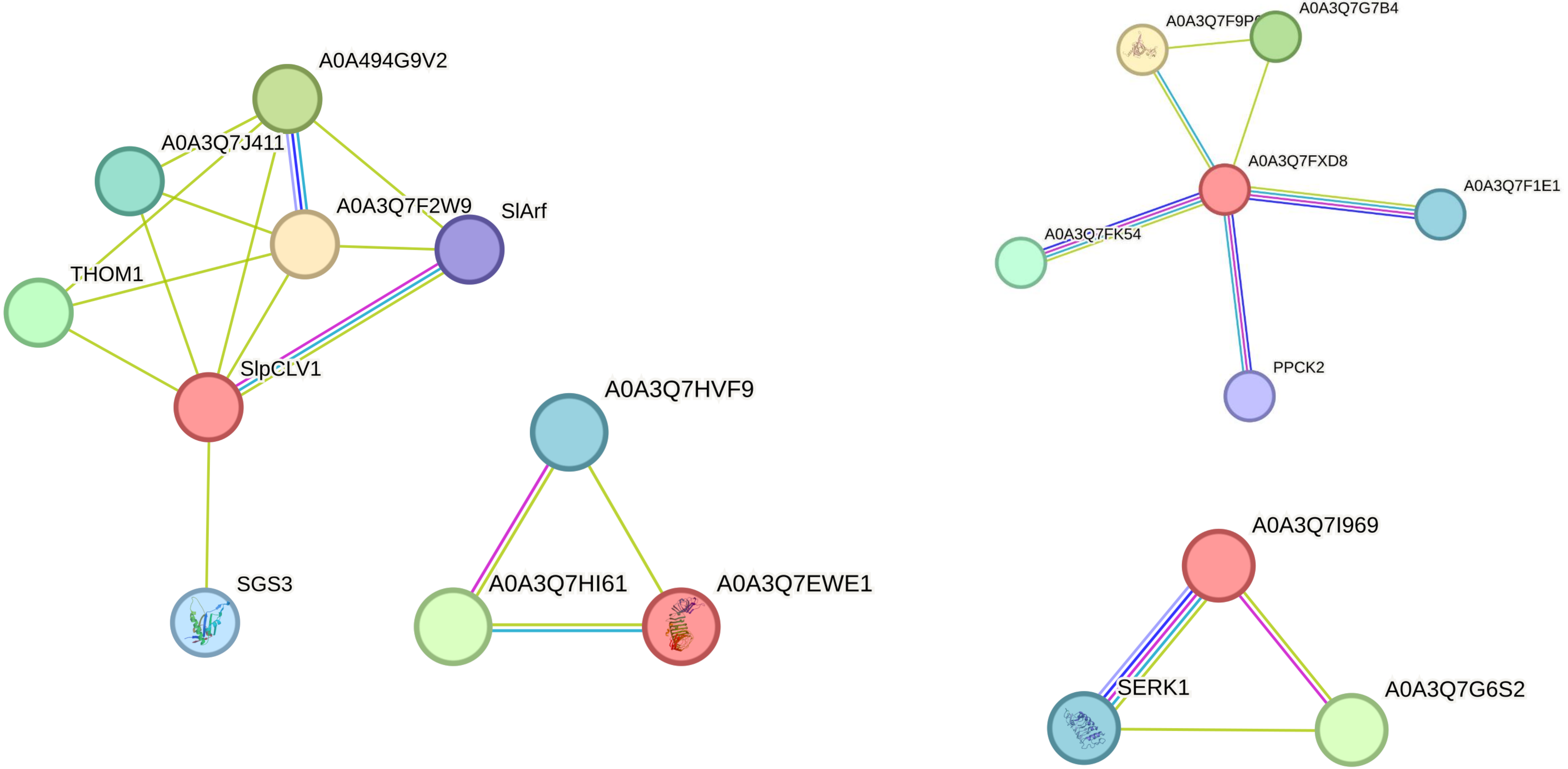

Fig. S78. RLK protein-protein interaction (PPI) network

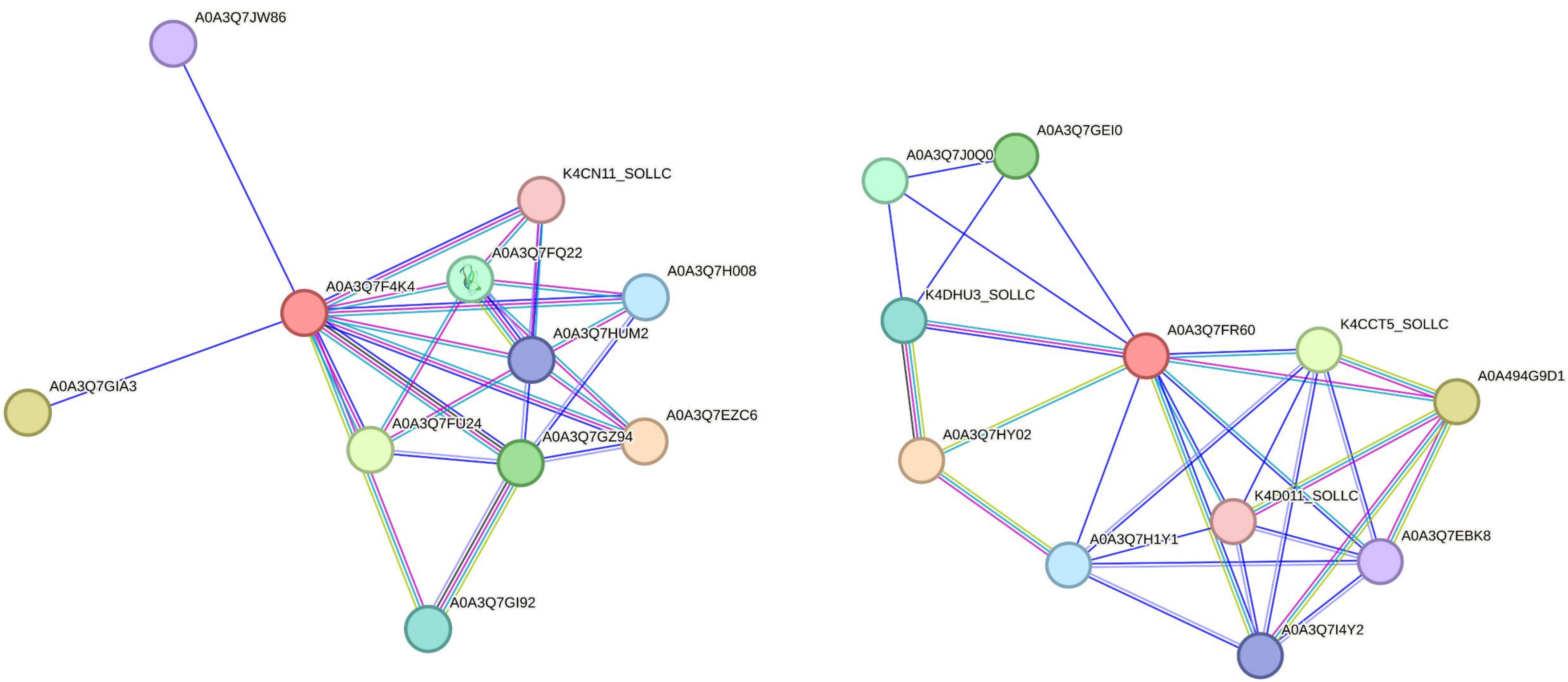

Fig. S79. RLK protein-protein interaction (PPI) network

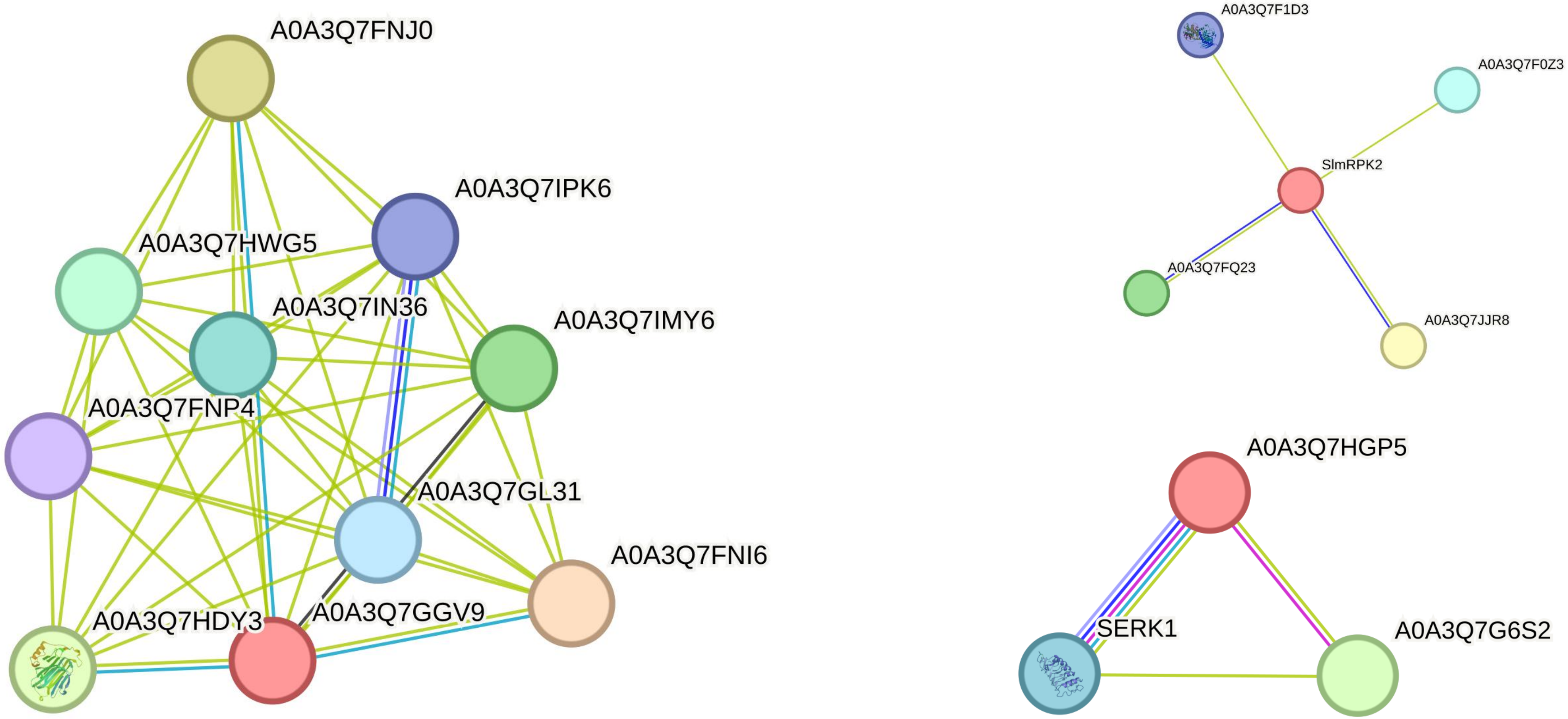

Fig. S80. RLK protein-protein interaction (PPI) network

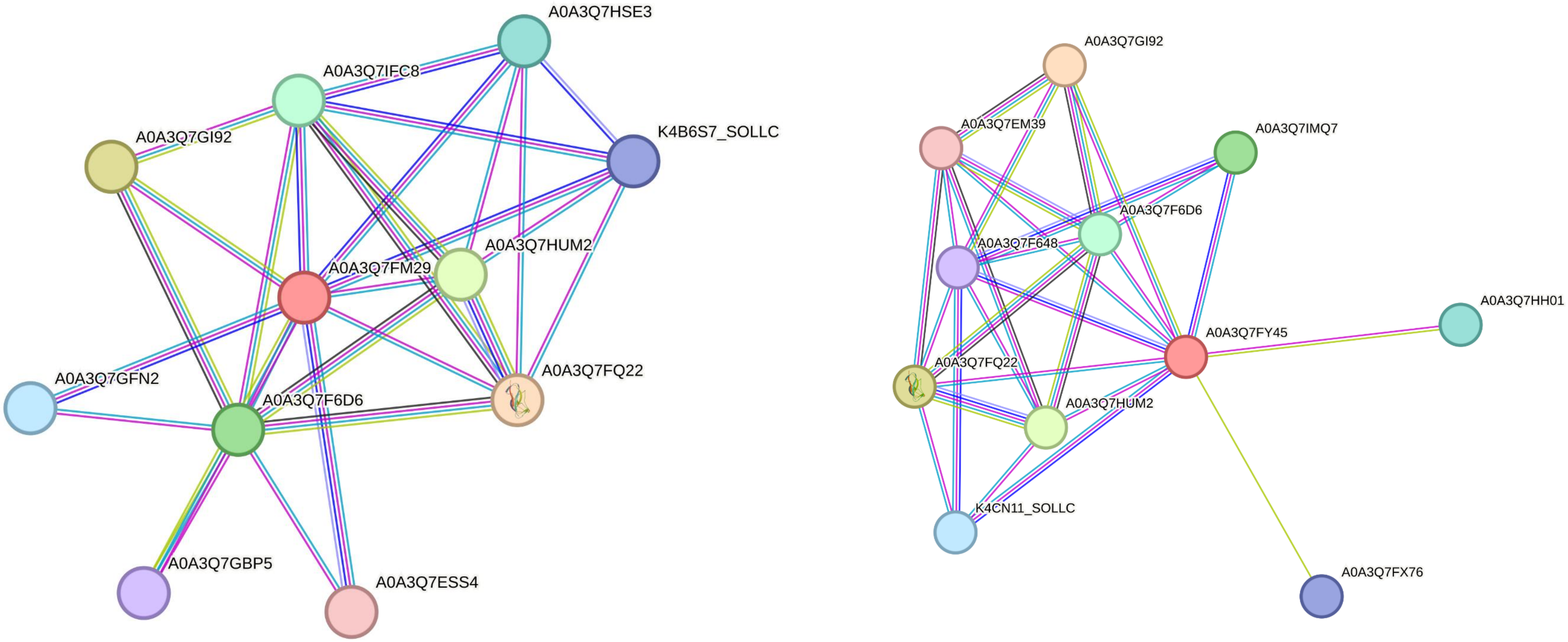

Fig. S81. RLK protein-protein interaction (PPI) network

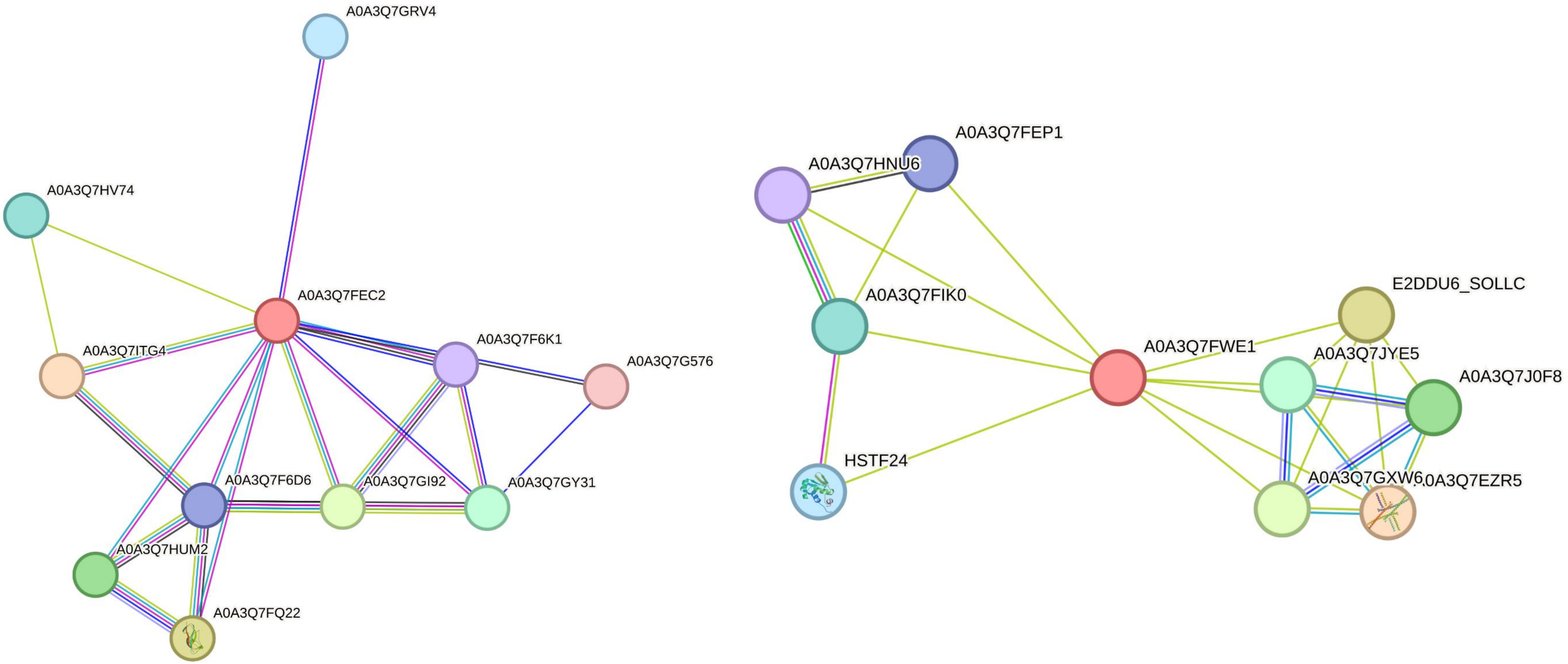

Fig. S82. RLK protein-protein interaction (PPI) network

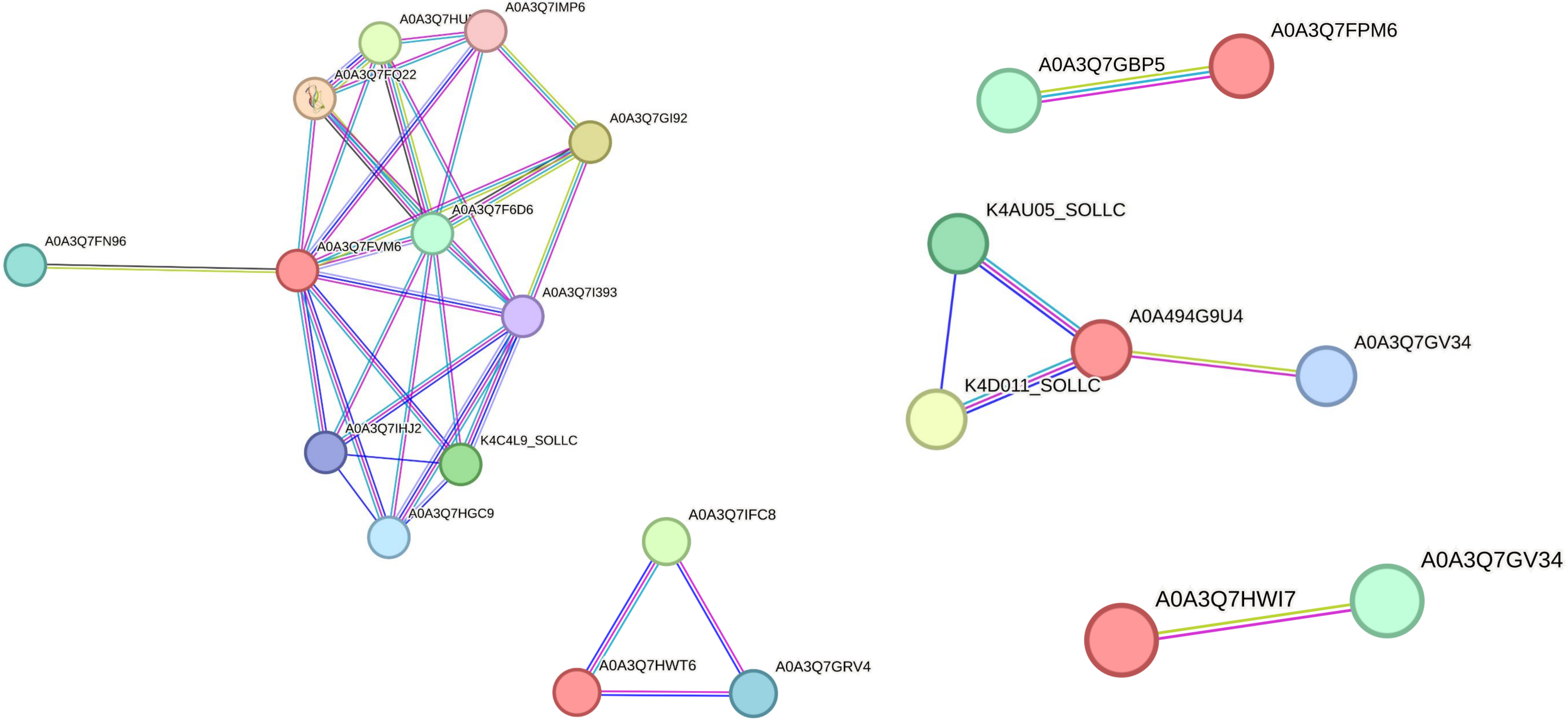

Fig. S83. RLK protein-protein interaction (PPI) network

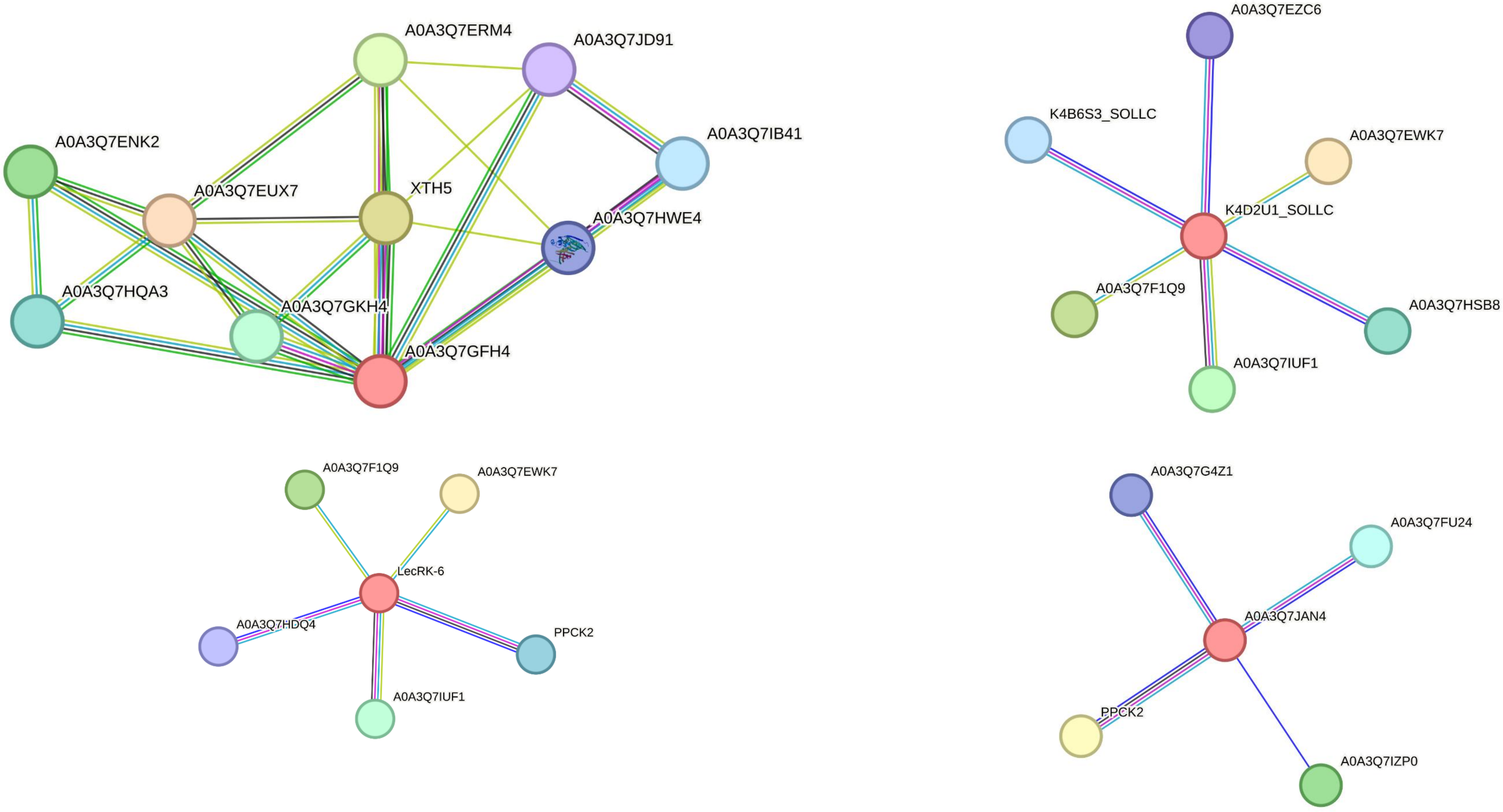

Fig. S84. RLK protein-protein interaction (PPI) network

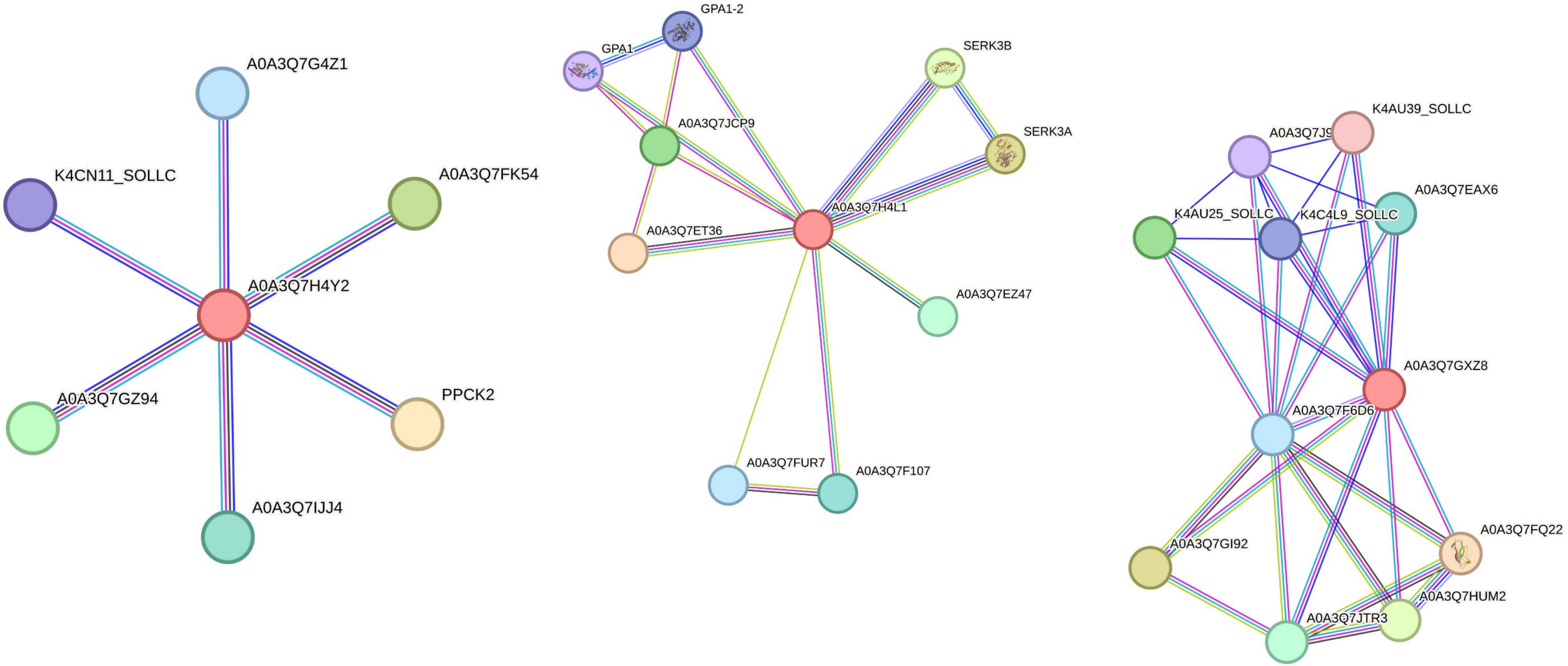

Fig. S85. RLK protein-protein interaction (PPI) network

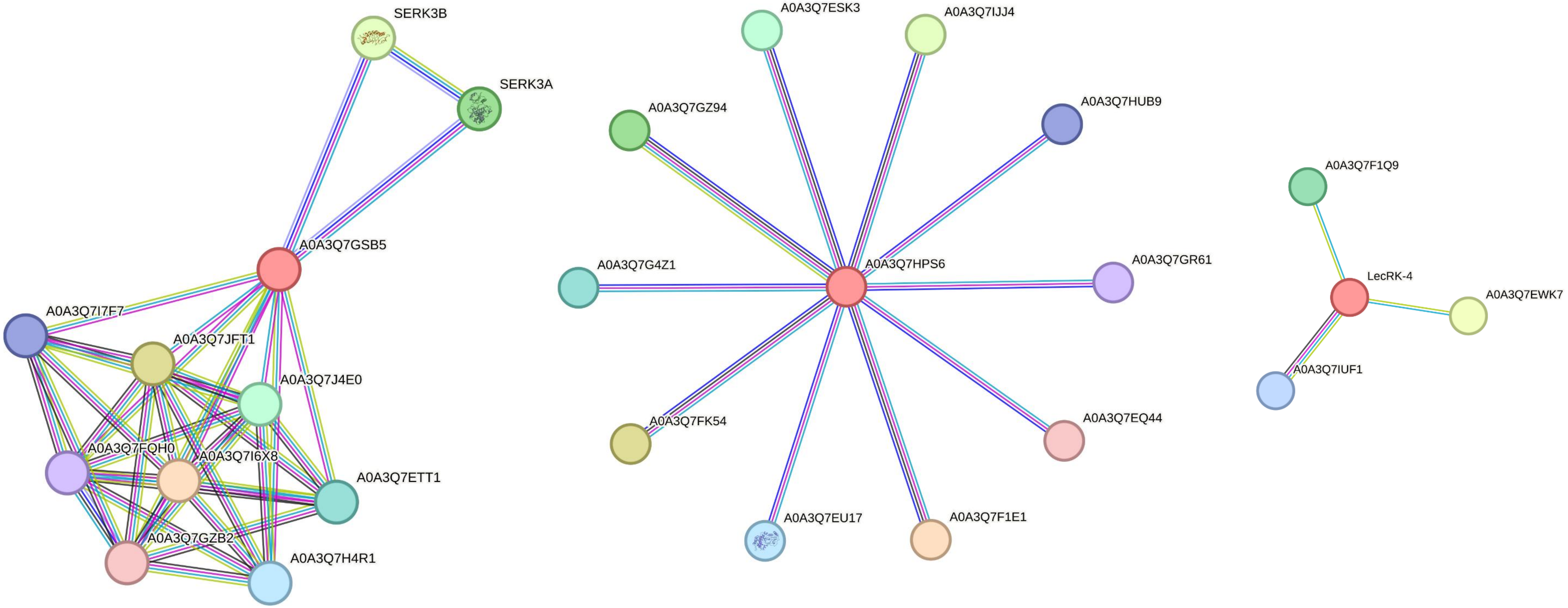

Fig. S86. RLK protein-protein interaction (PPI) network

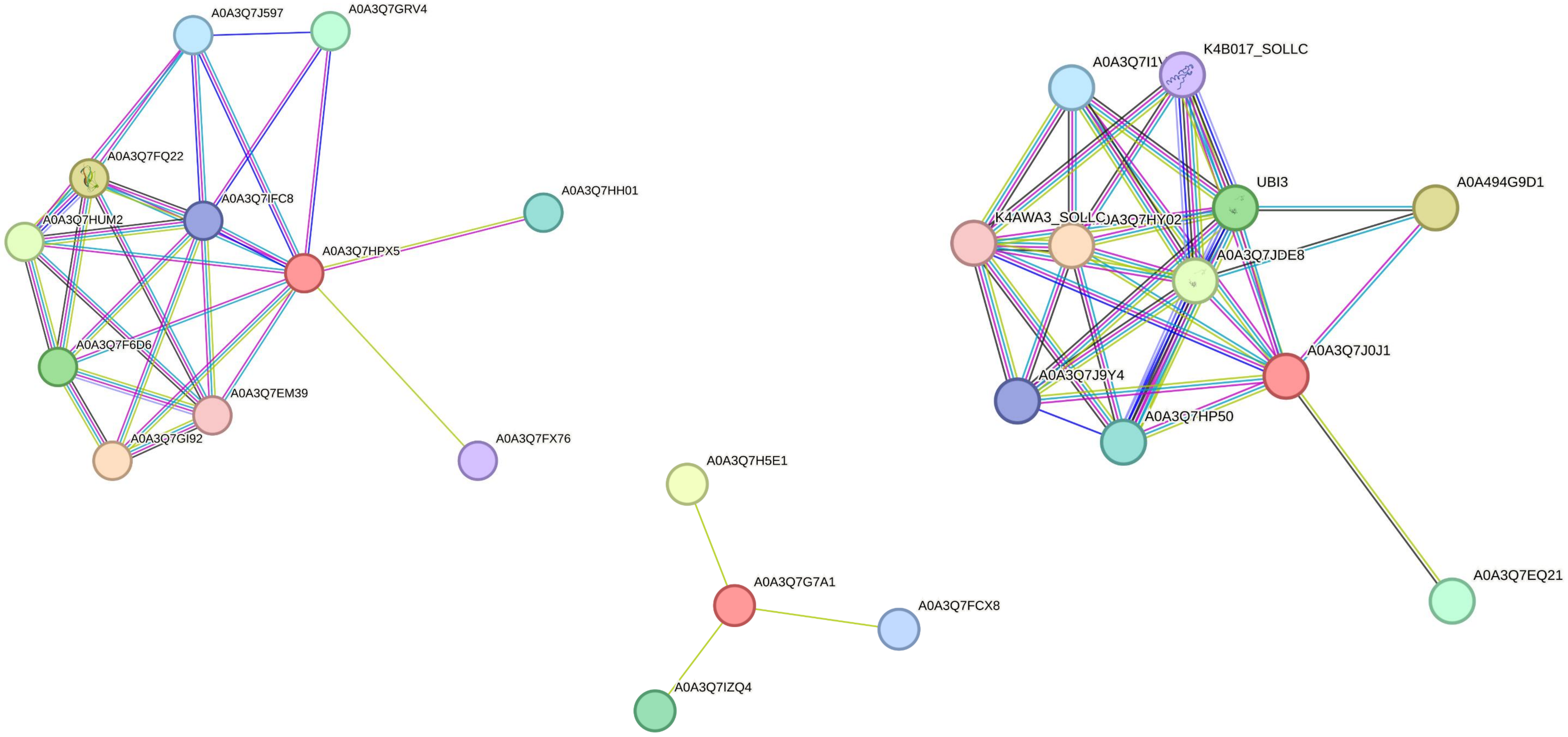

Fig. S87. RLK protein-protein interaction (PPI) network

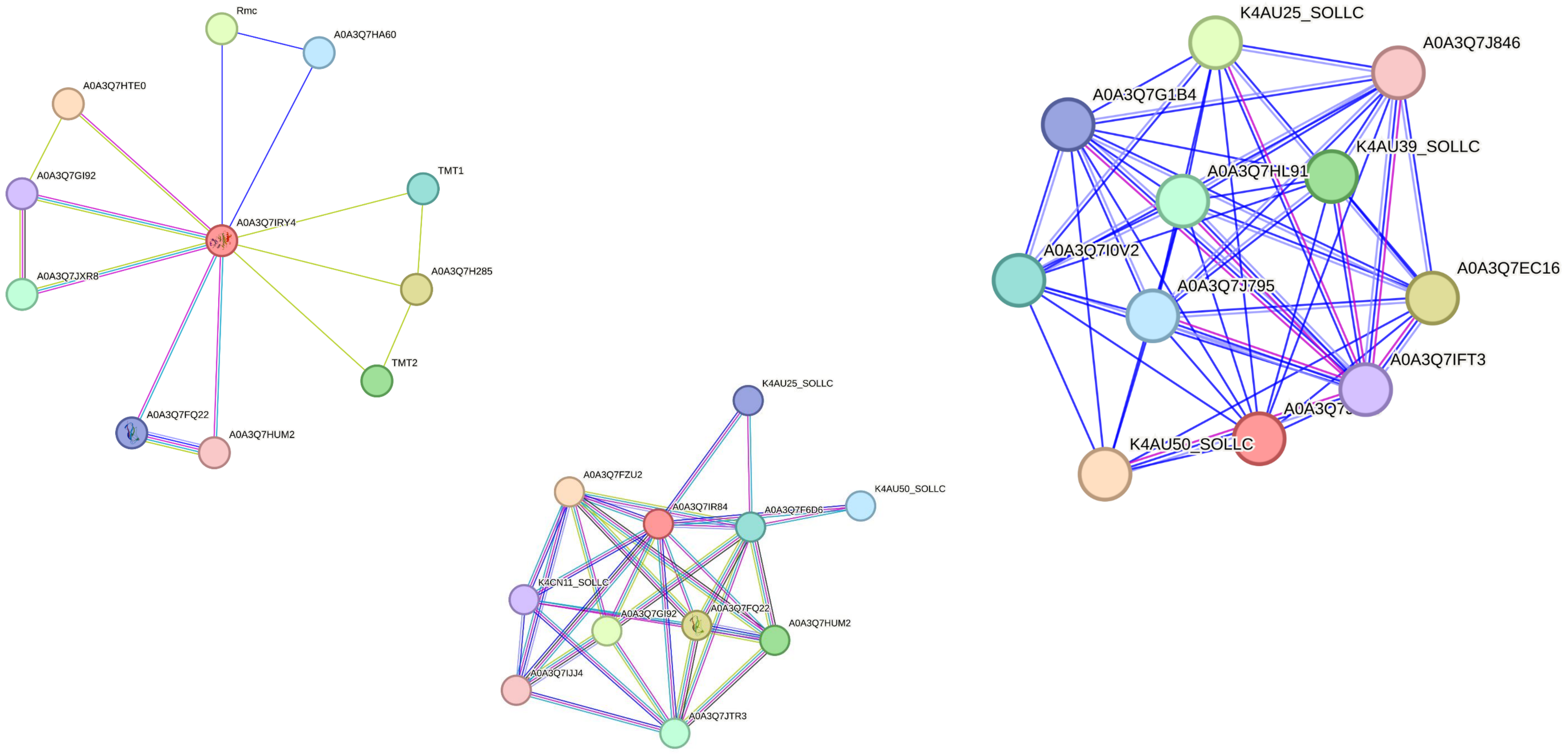

Fig. S88. RLK protein-protein interaction (PPI) network

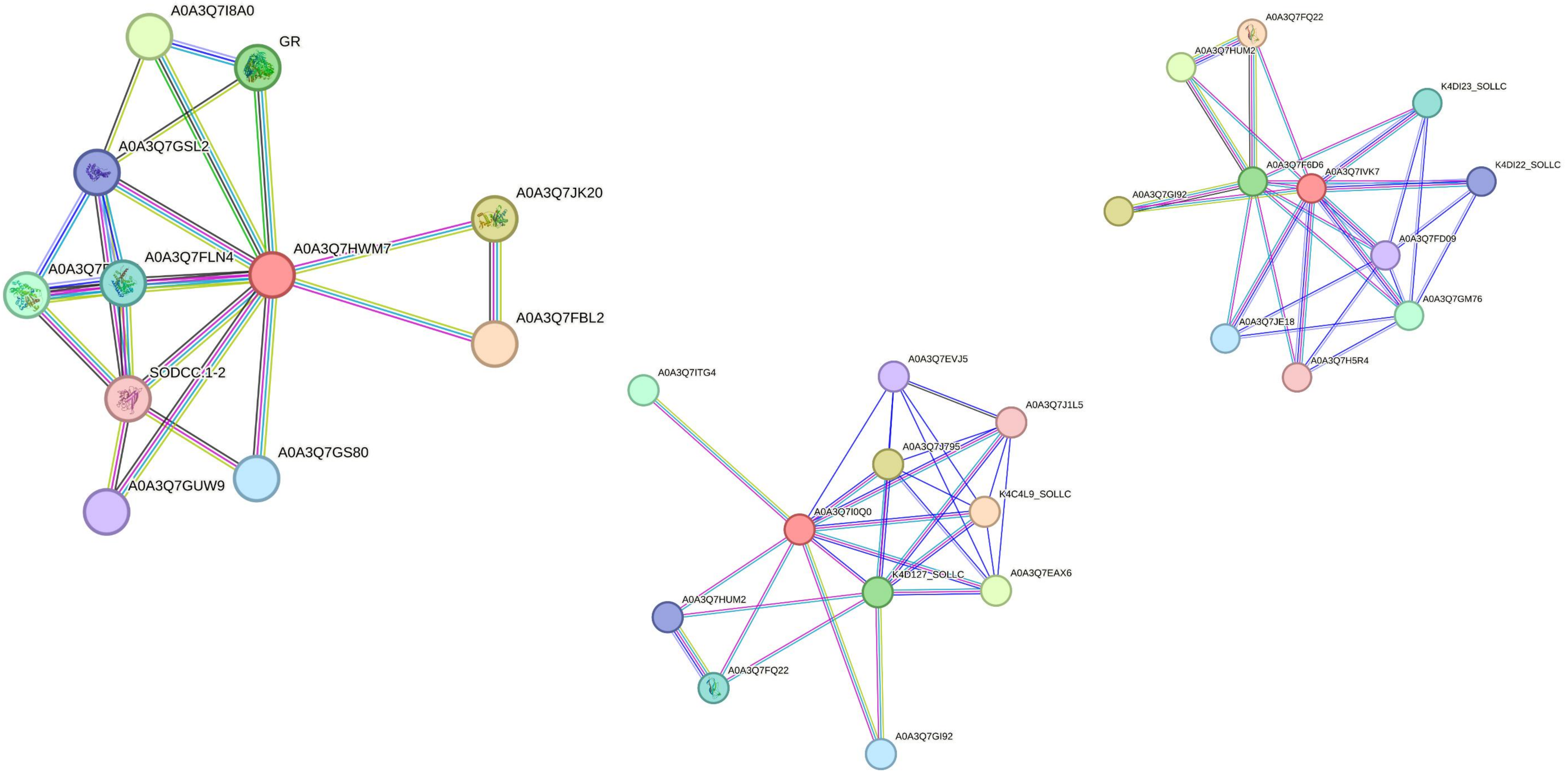

Fig. S89. RLK protein-protein interaction (PPI) network

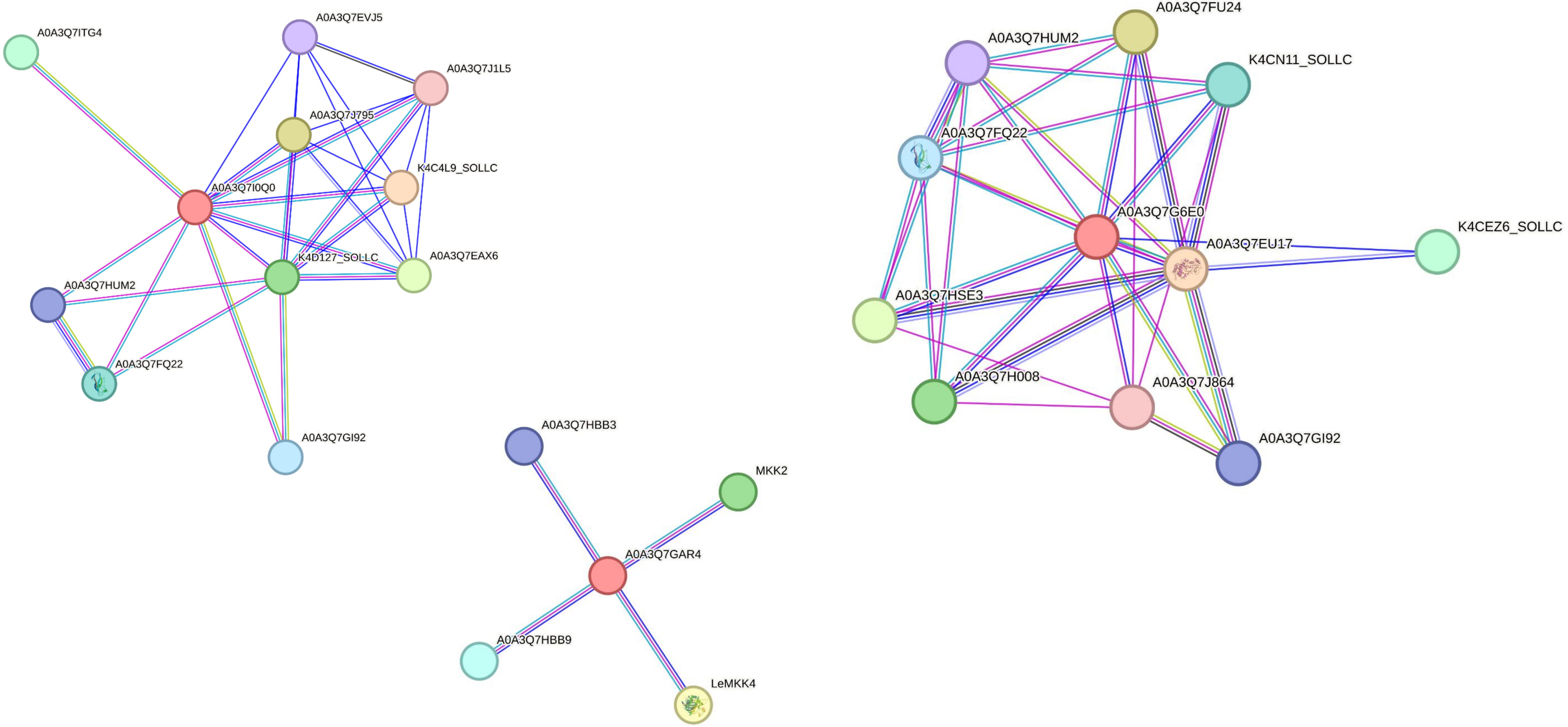

Fig. S90. RLK protein-protein interaction (PPI) network

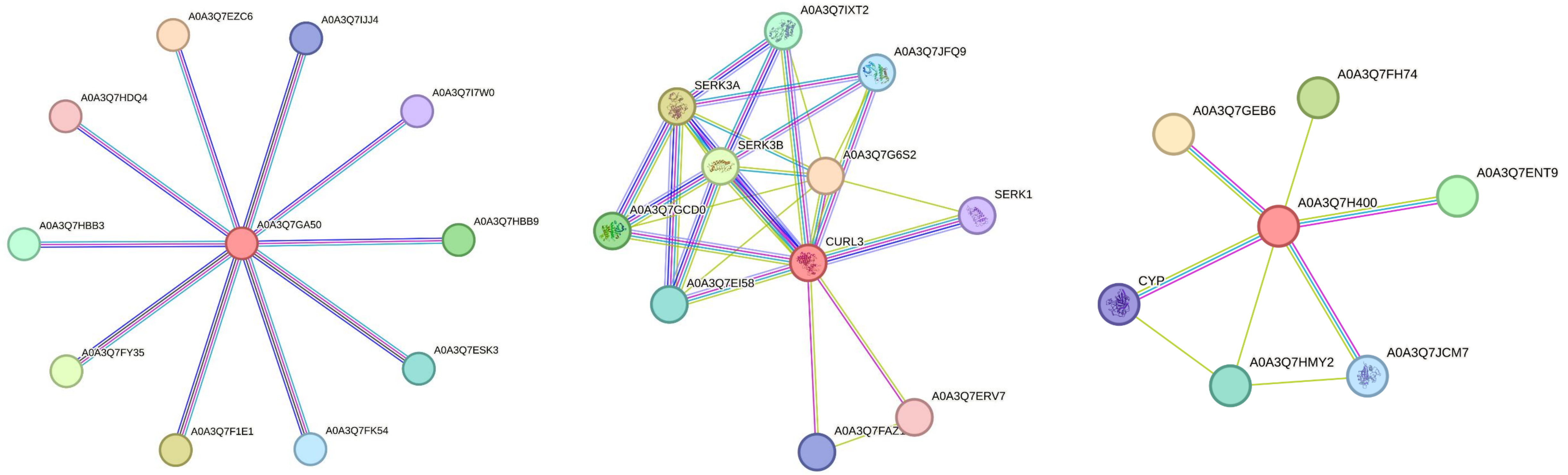

Fig. S91. RLK protein-protein interaction (PPI) network

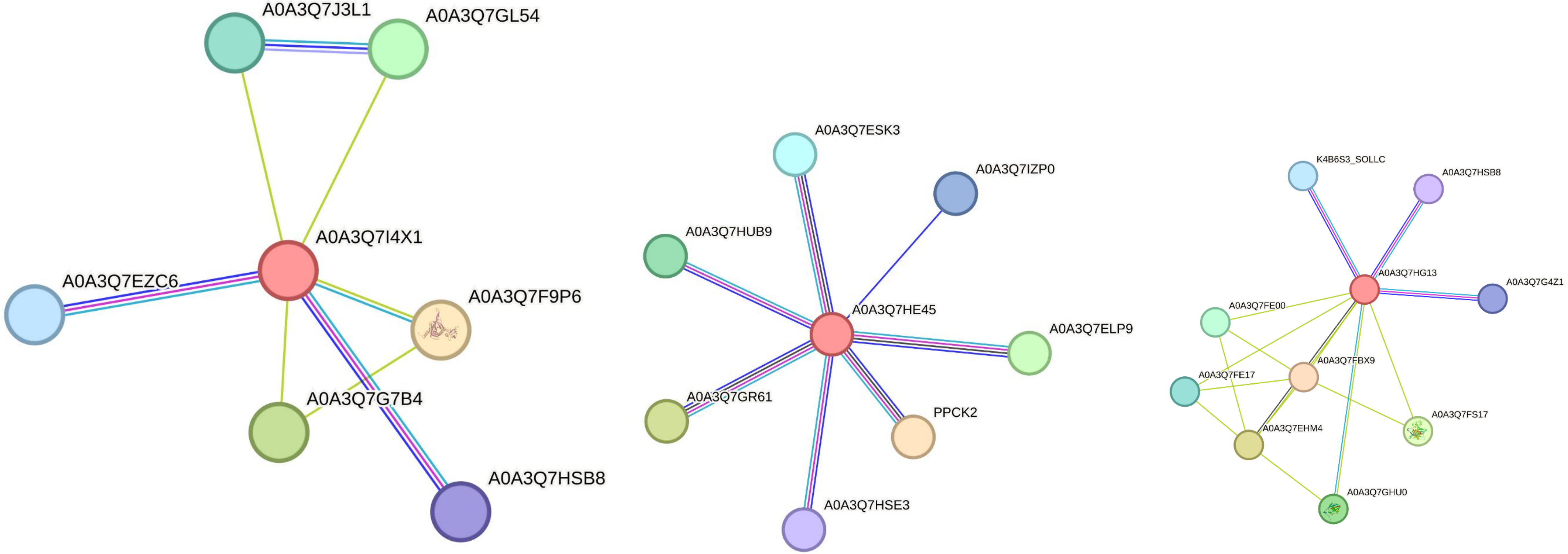

Fig. S92. RLK protein-protein interaction (PPI) network

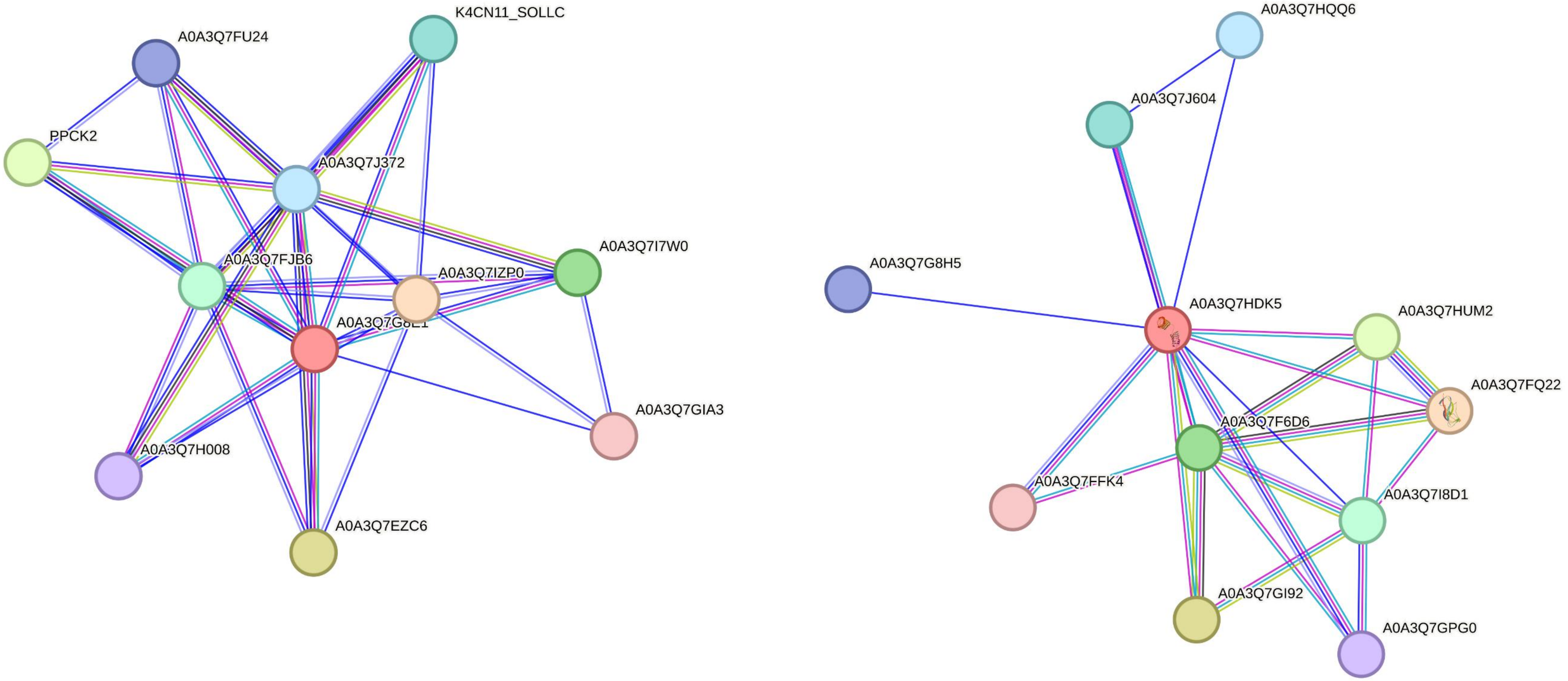

Fig. S93. RLK protein-protein interaction (PPI) network

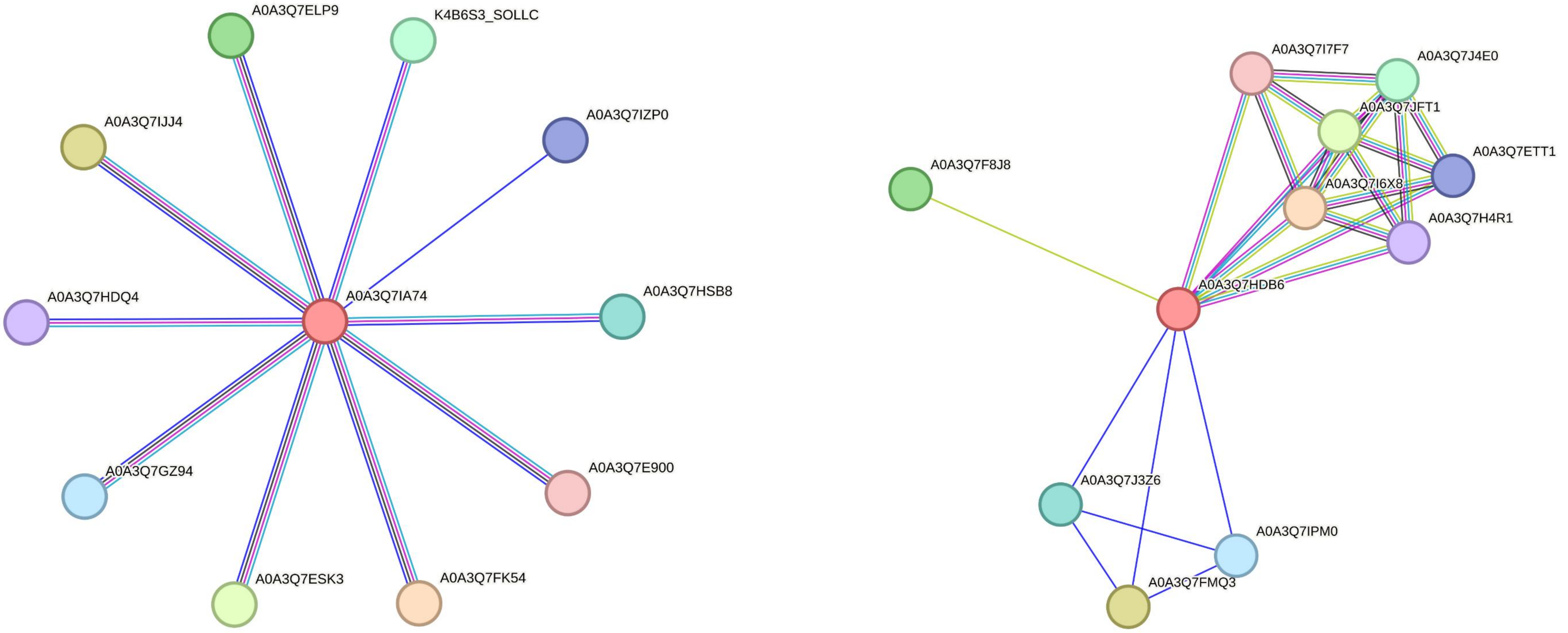

Fig. S94. RLK protein-protein interaction (PPI) network

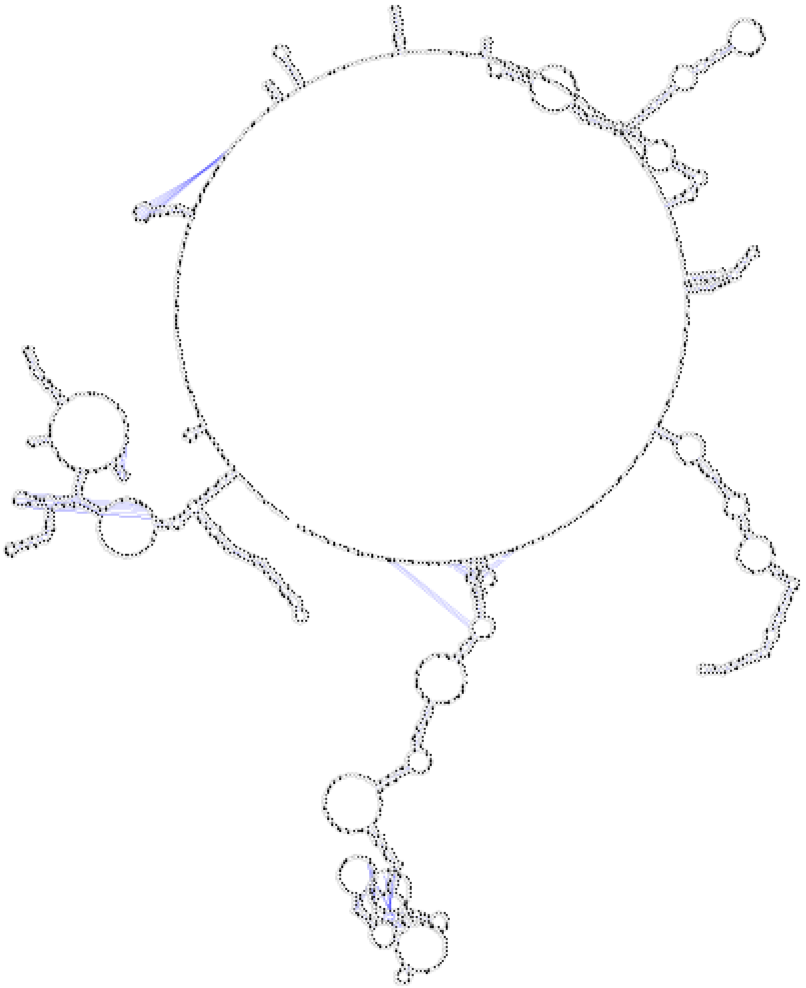

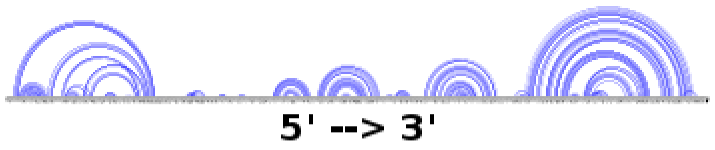

Fig. S95. Predicting RNA secondary structures of SlCHX-3

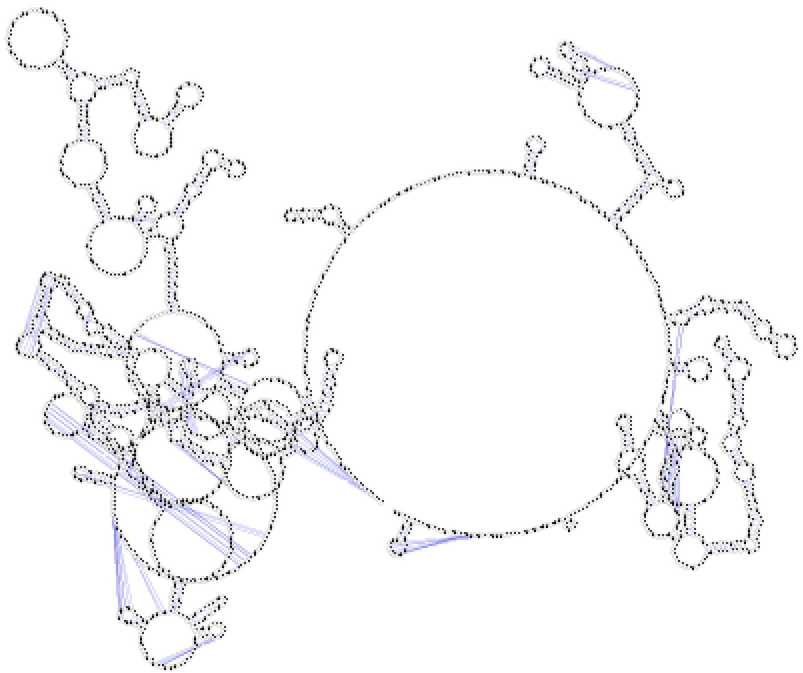

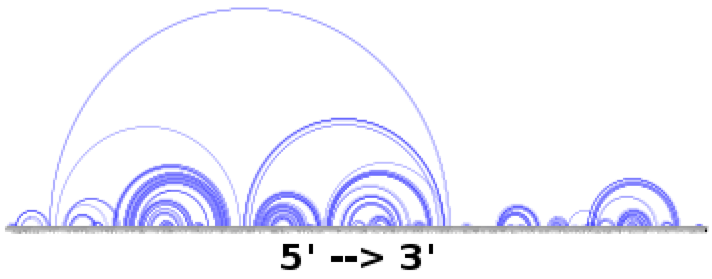

Fig. S96. Predicting RNA secondary structures of SlCHX-5

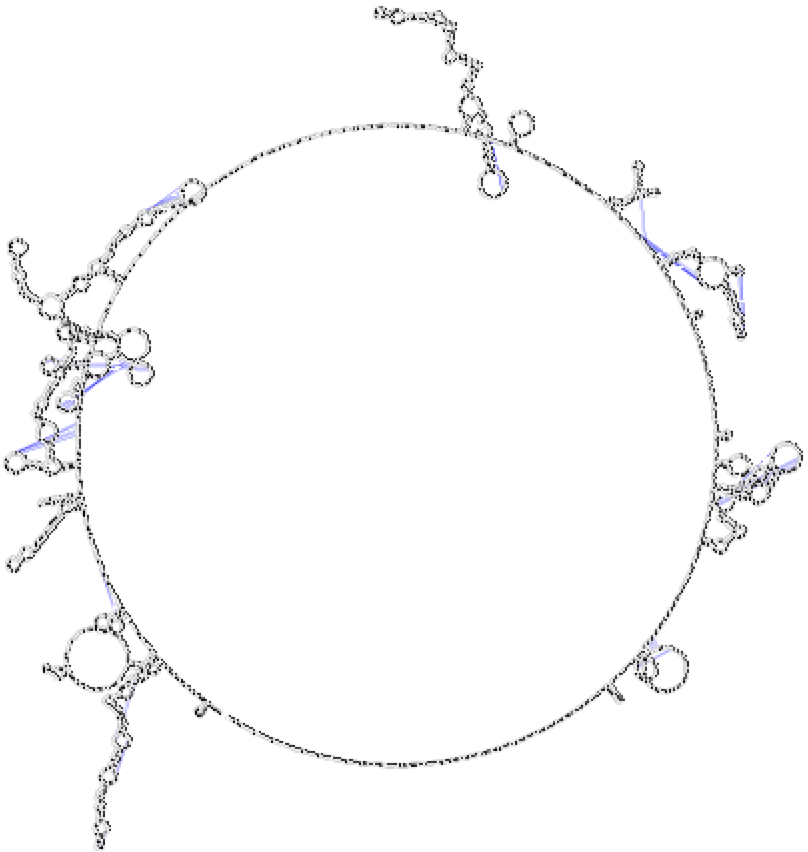

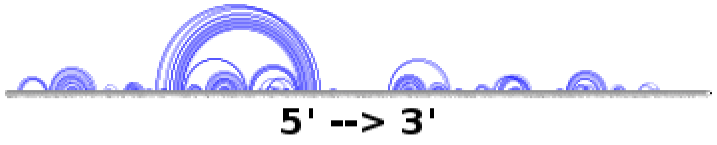

Fig. S97. Predicting RNA secondary structures of SlCHX-10

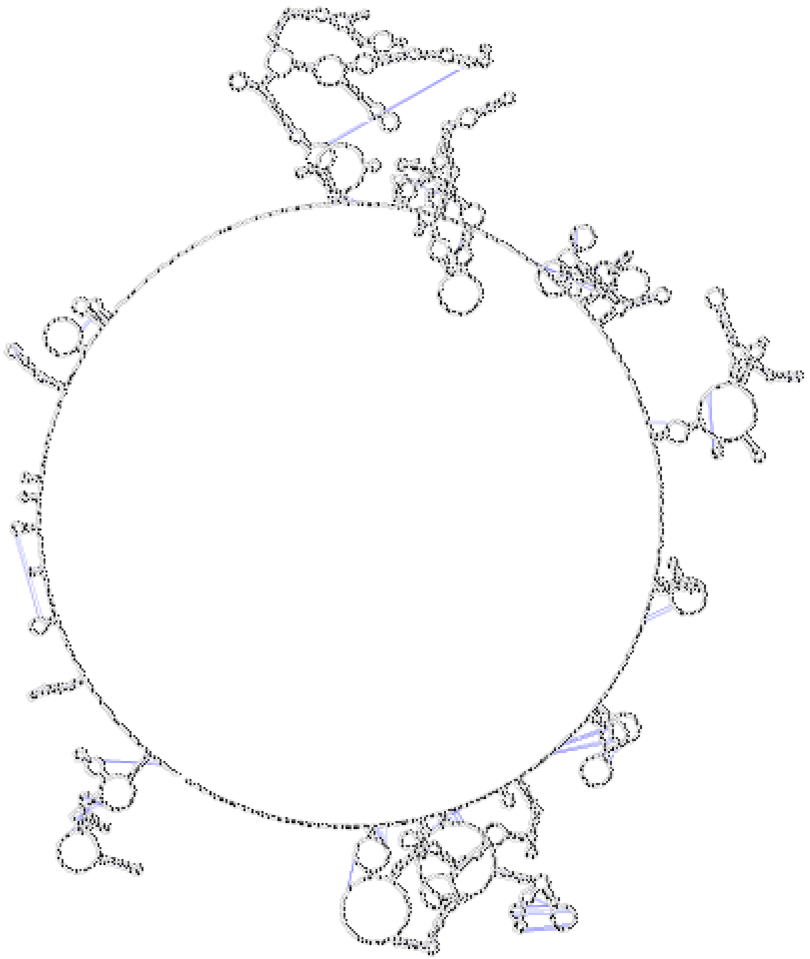

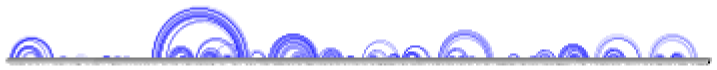

Fig. S98. Predicting RNA secondary structures of SlSOS-2

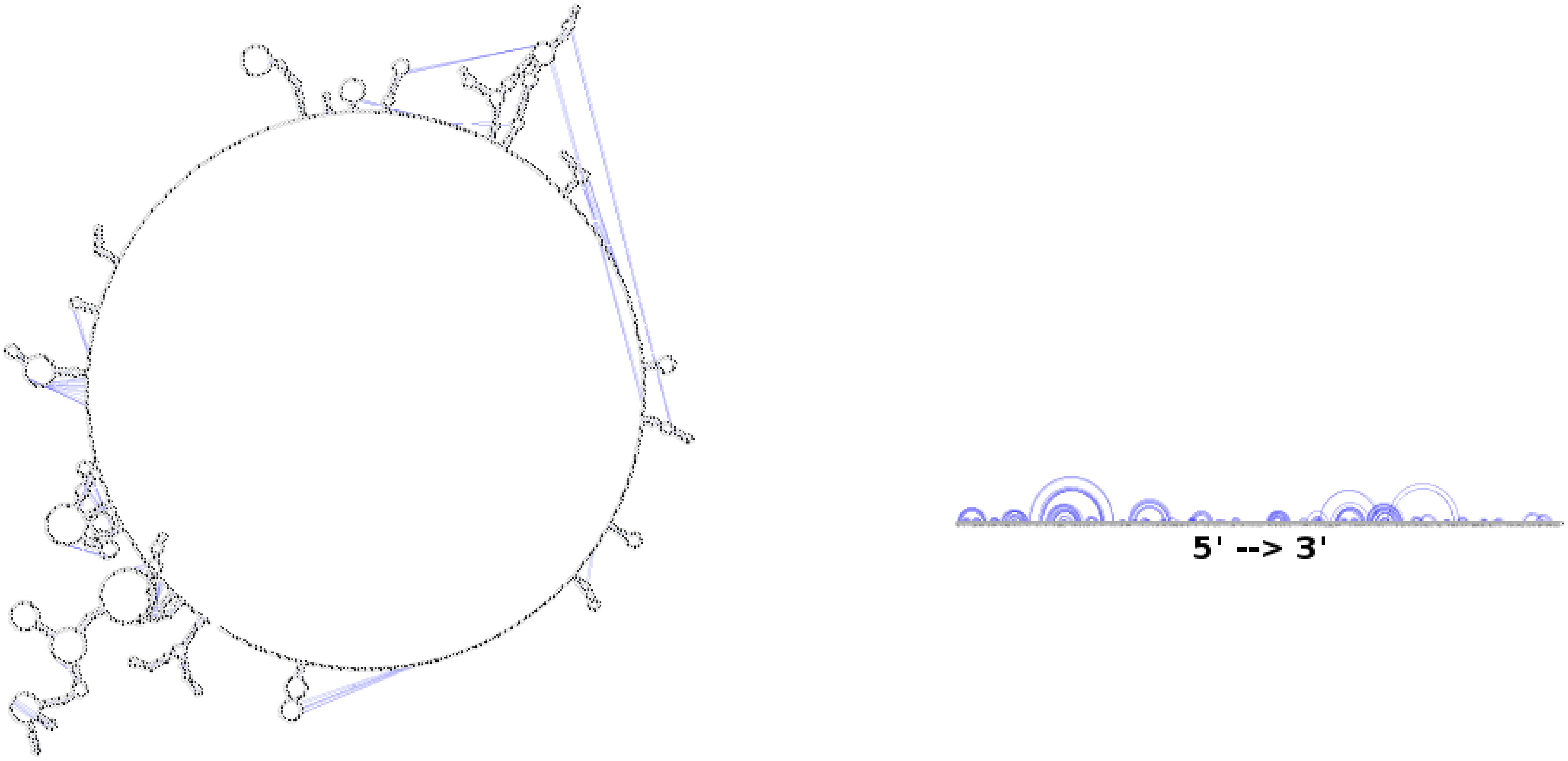

Fig. S99. Predicting RNA secondary structures of SlSOS-3

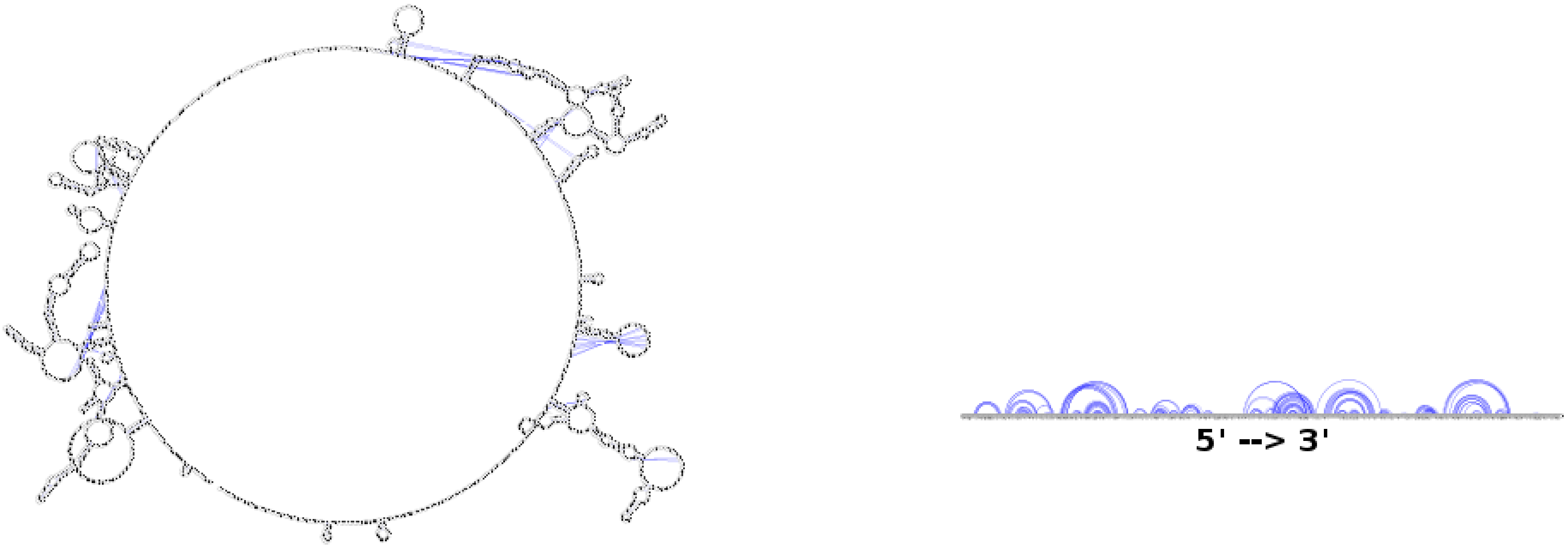

Fig. S100. Predicting RNA secondary structures of SlSOS-5

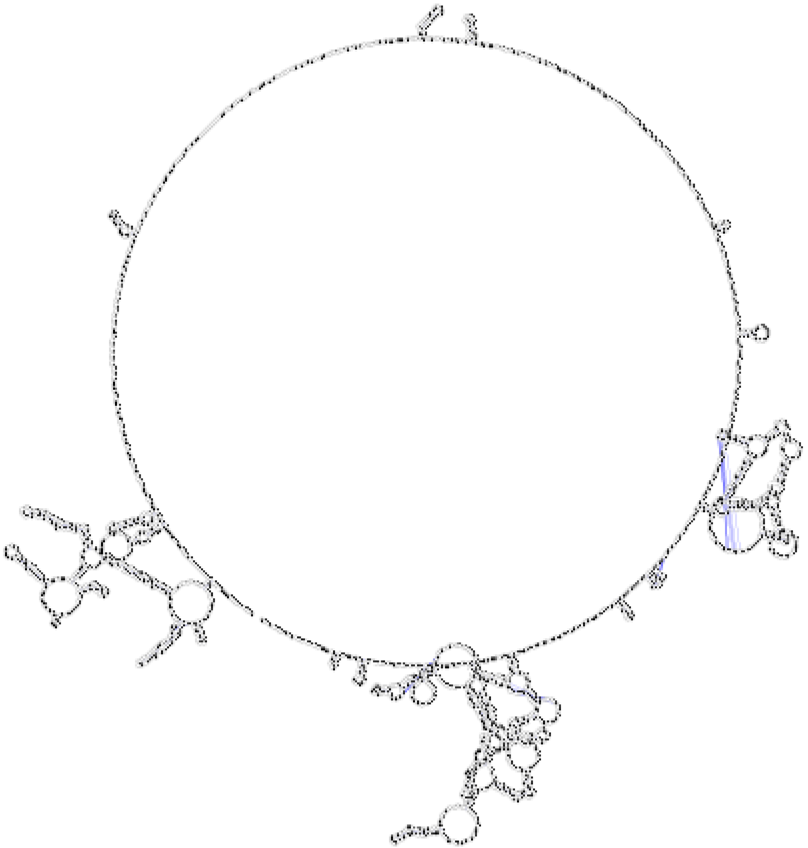

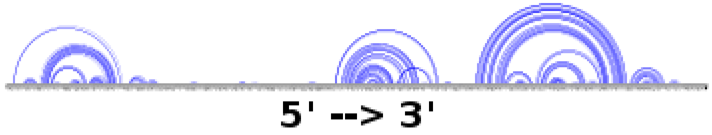

Fig. S101. Predicting RNA secondary structures of SlRLK-39

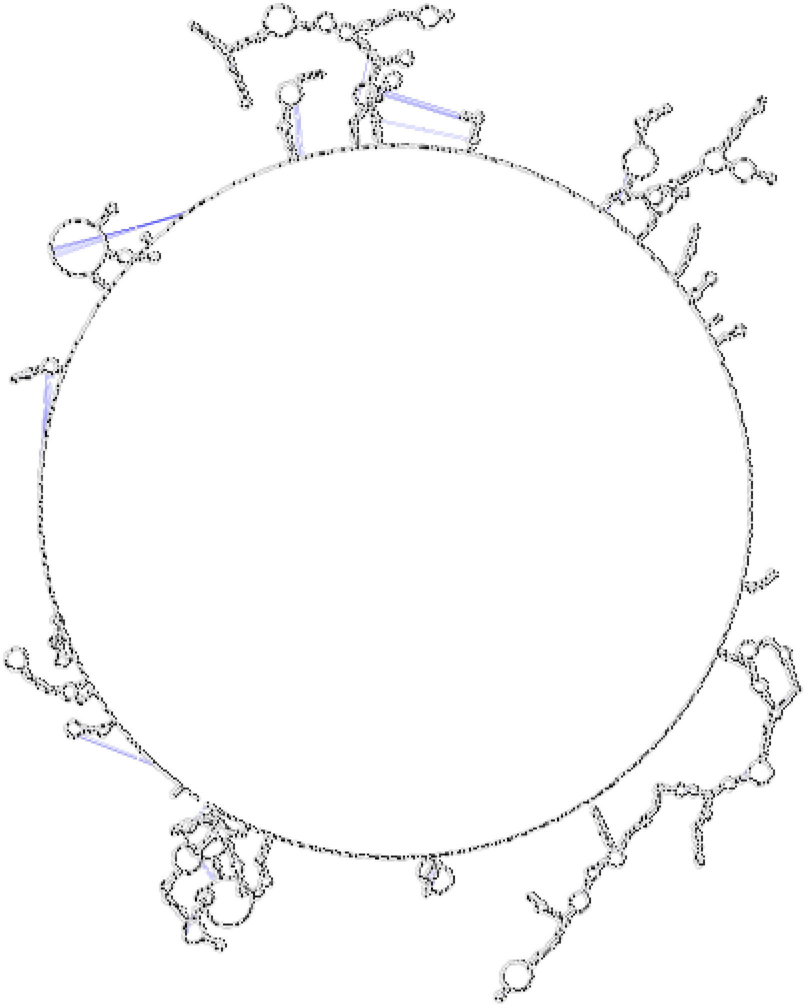

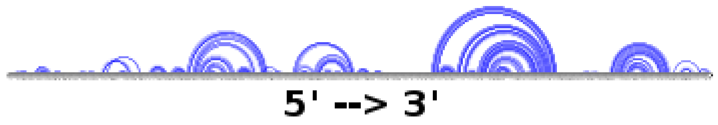

Fig. S102. Predicting RNA secondary structures of SlRLK-42

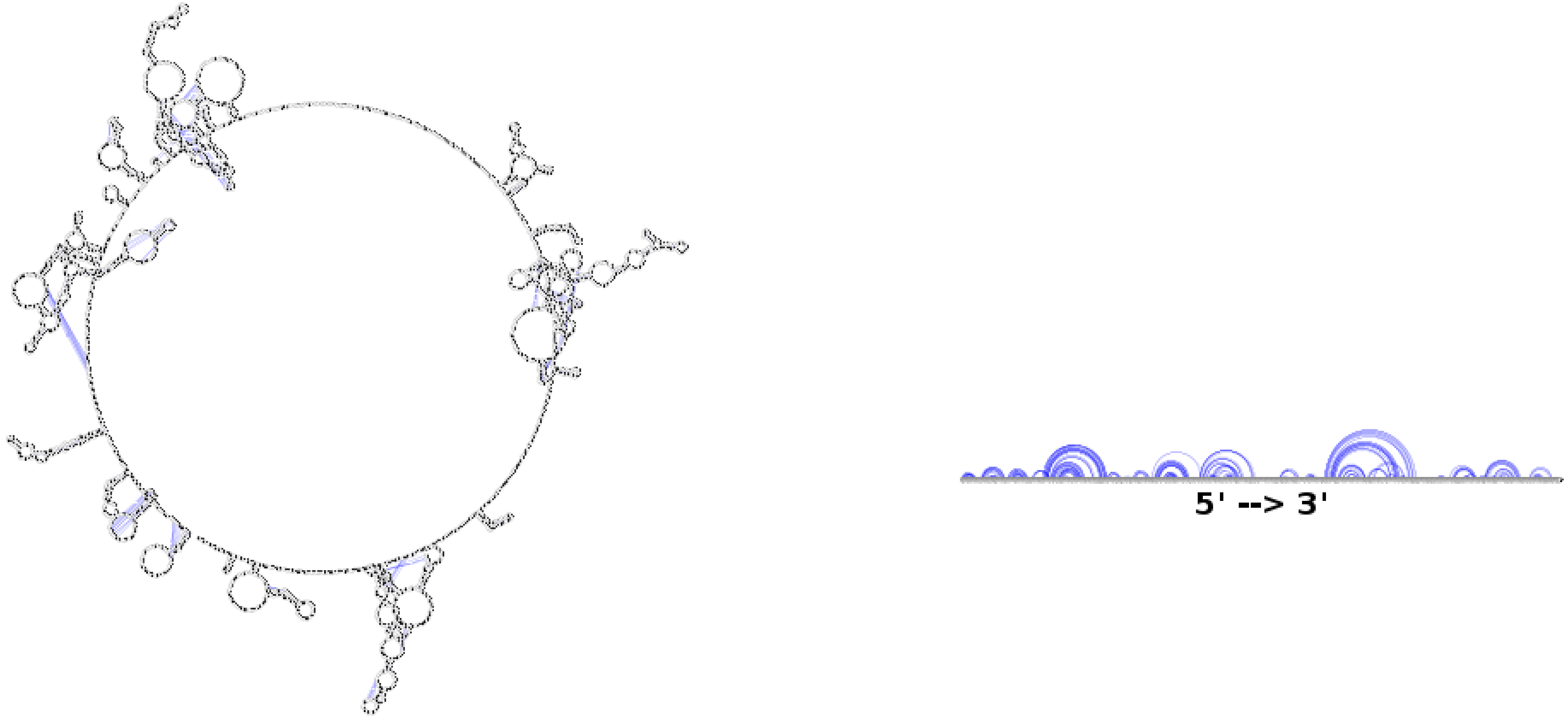

Fig. S103. Predicting RNA secondary structures of SlRLK-47

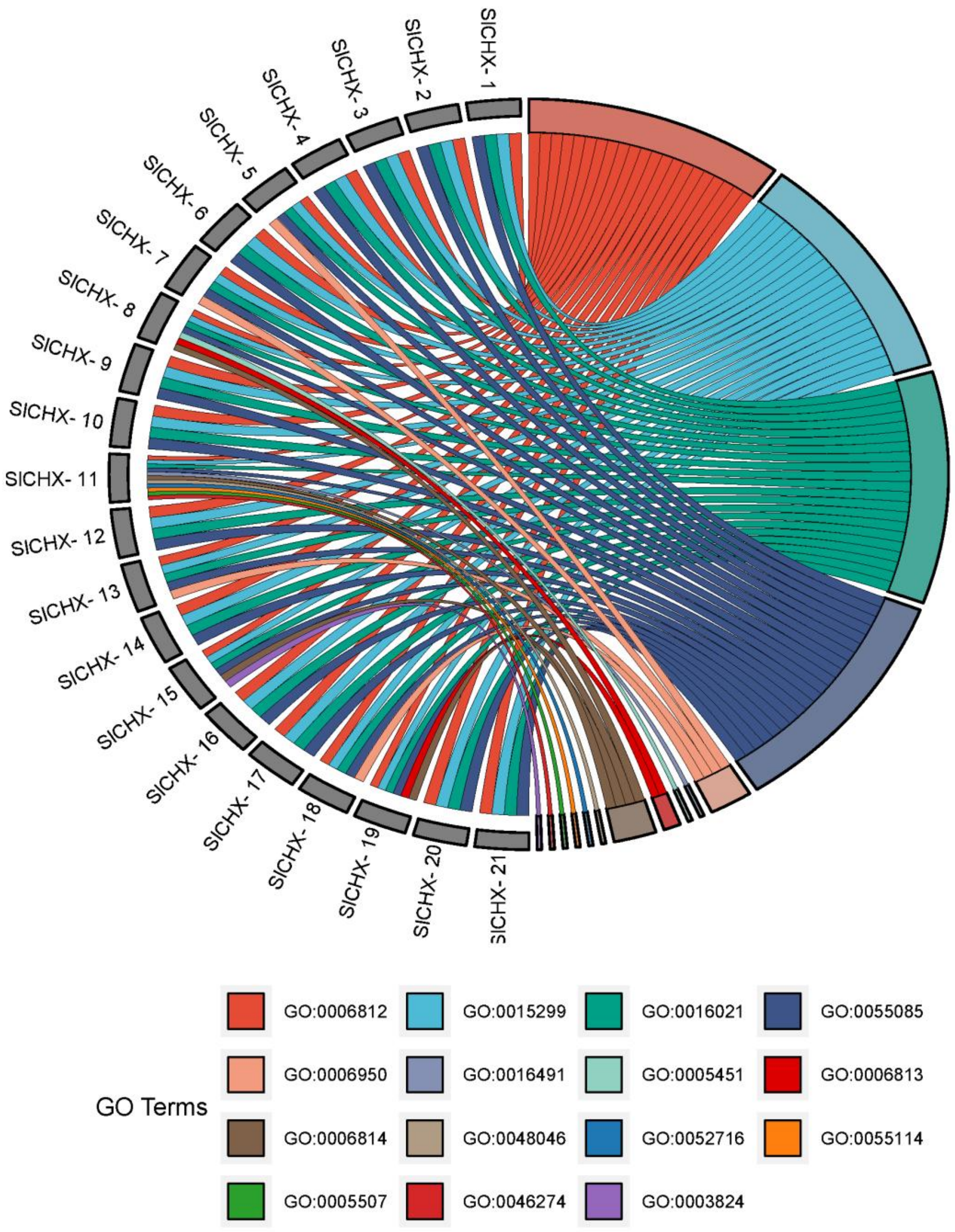

Fig. S104. GO terms for CHX

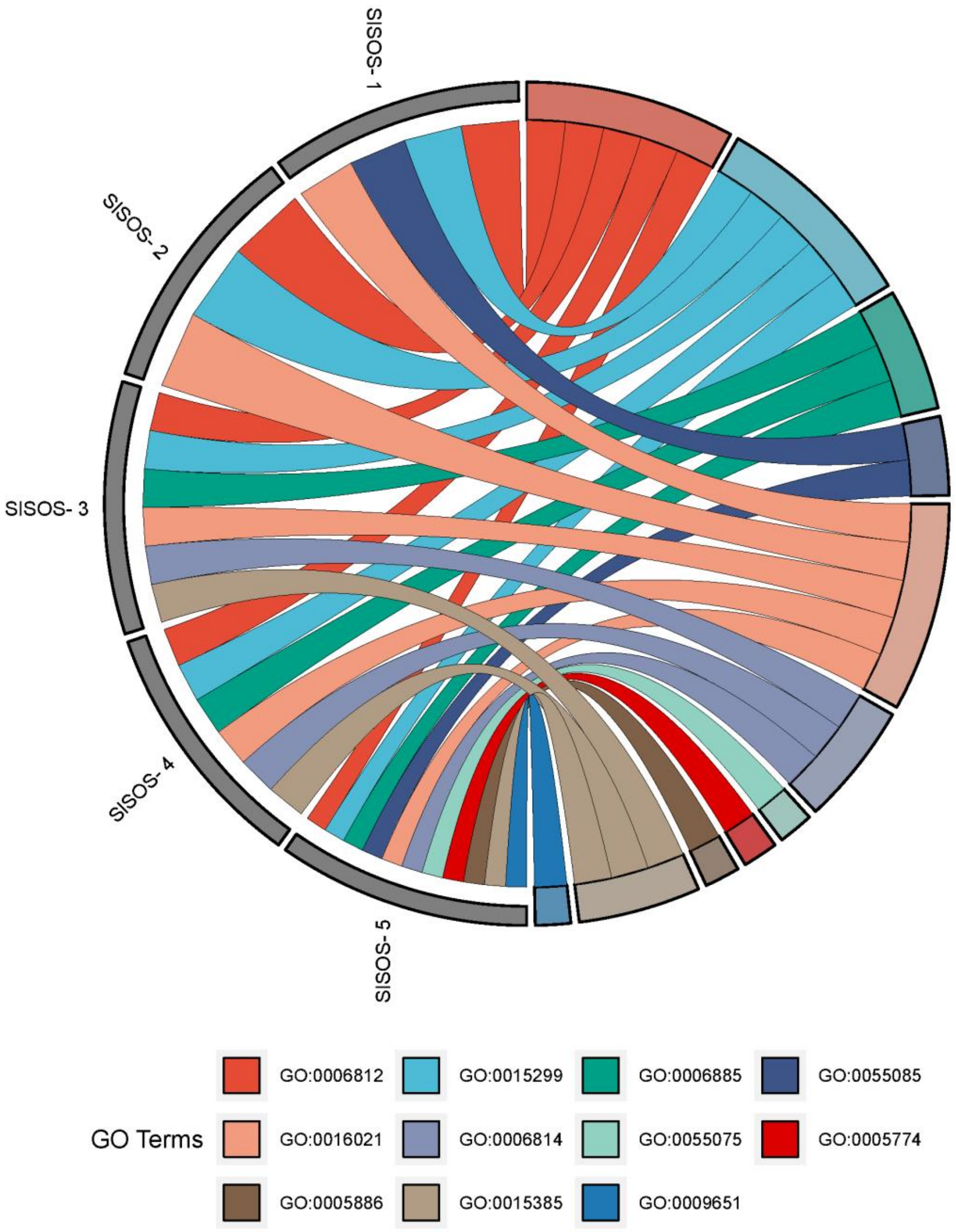

Fig. S105. GO terms for SOS

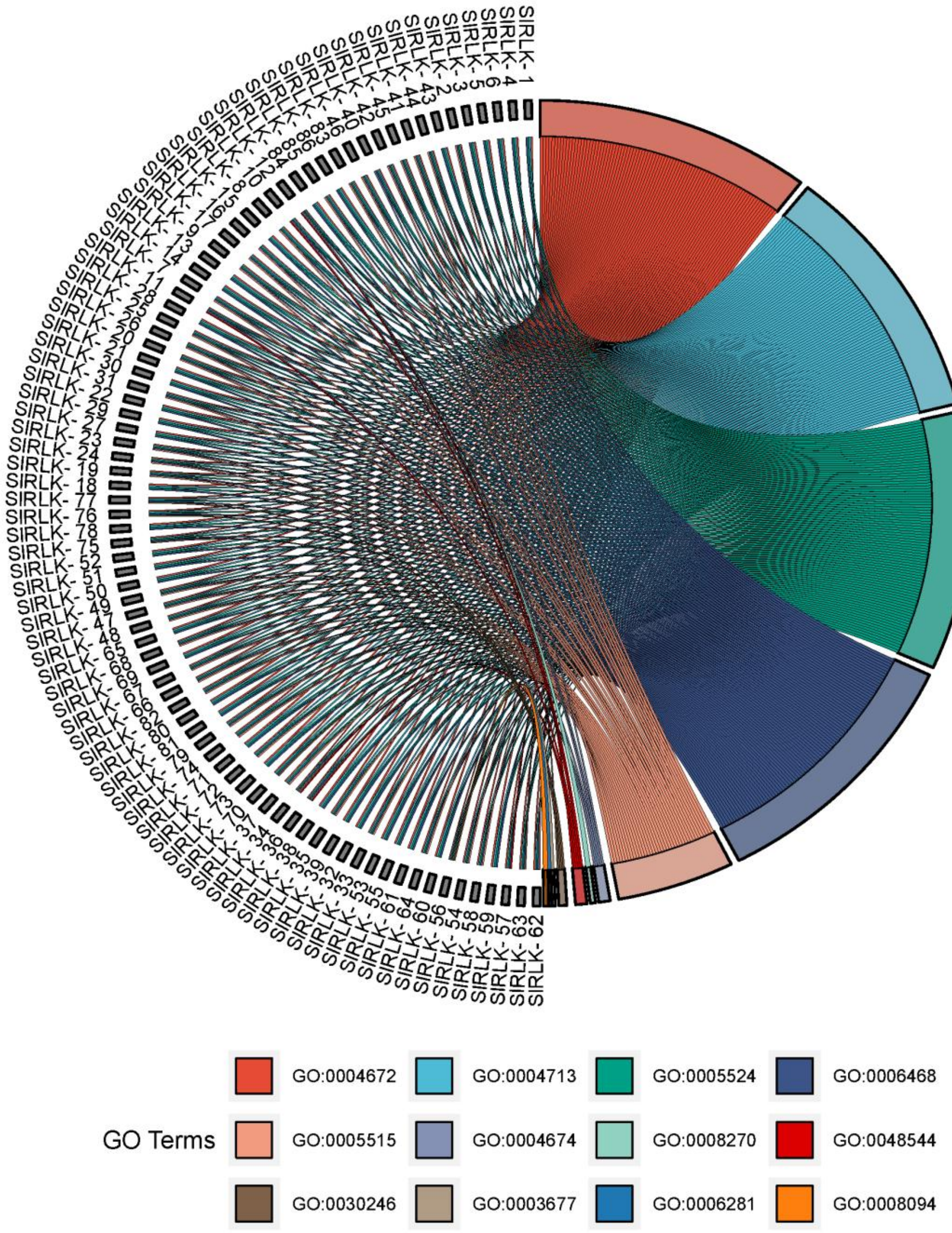

Fig. S106. GO terms for RLK

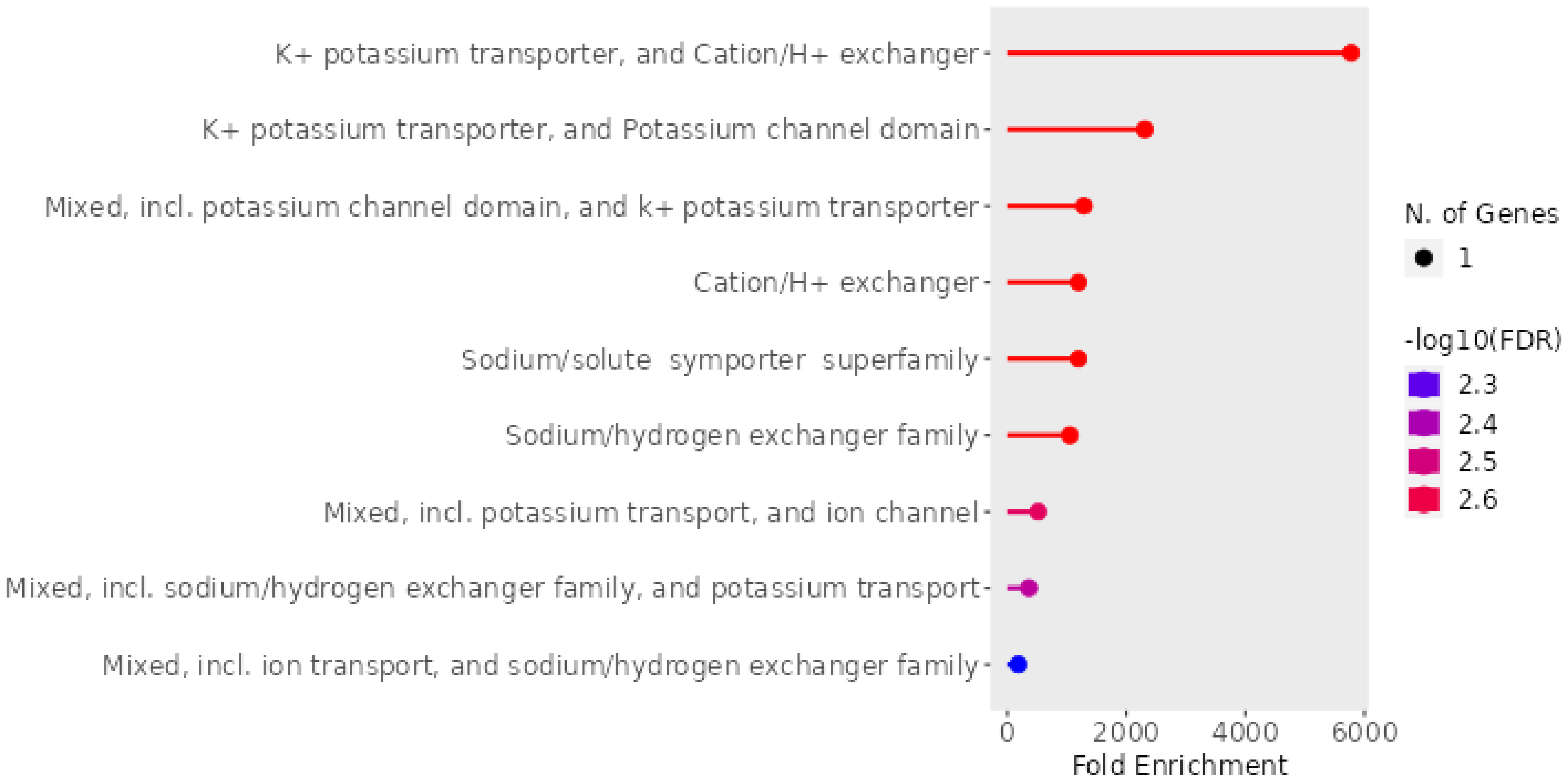

Fig. S107. Gene ontology enrichment analysis were confirmed the functional role of CHX as stress-responsive

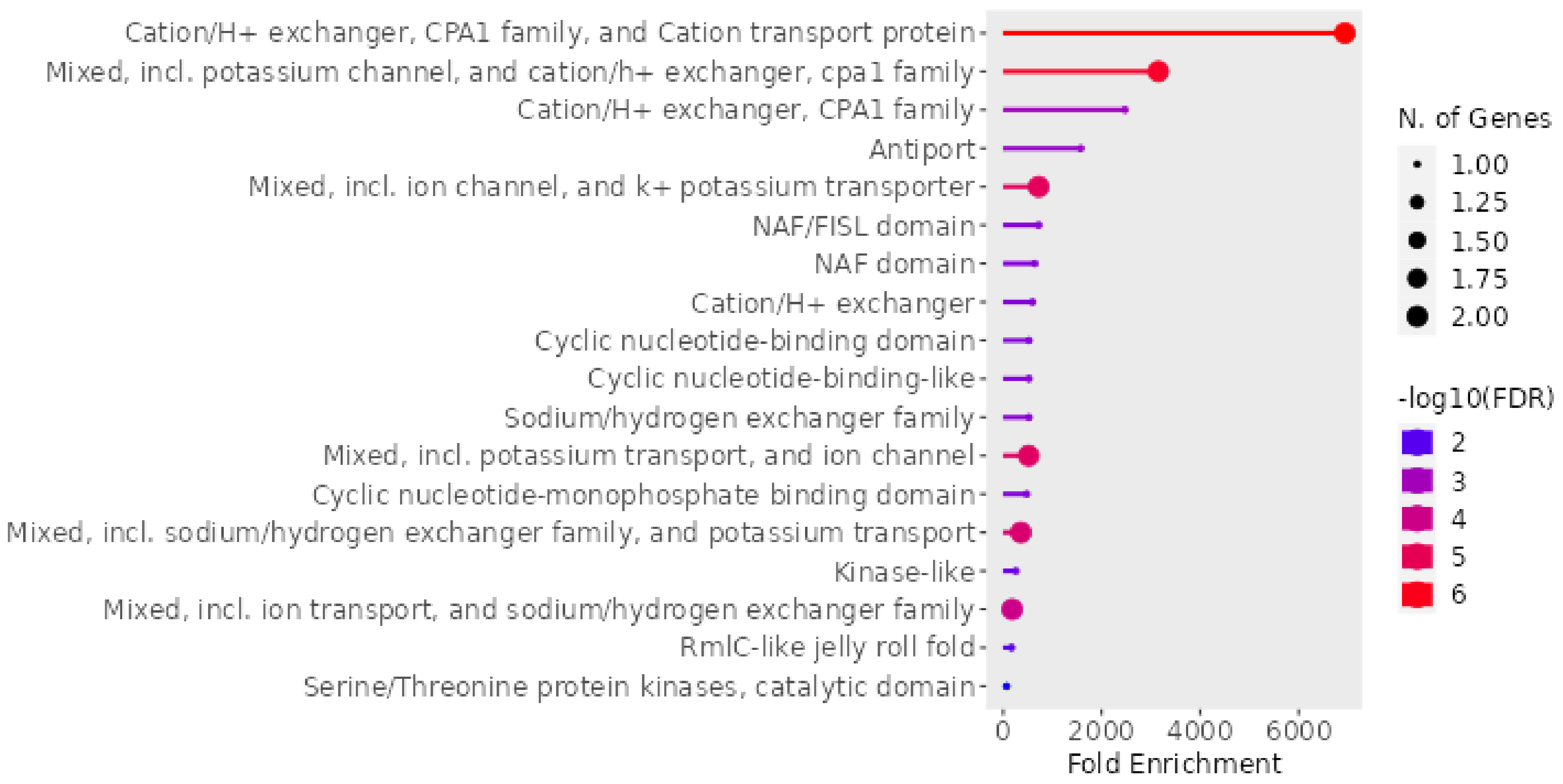

Fig. S108. Gene ontology enrichment analysis were confirmed the functional role of SOS as a stress-responsive

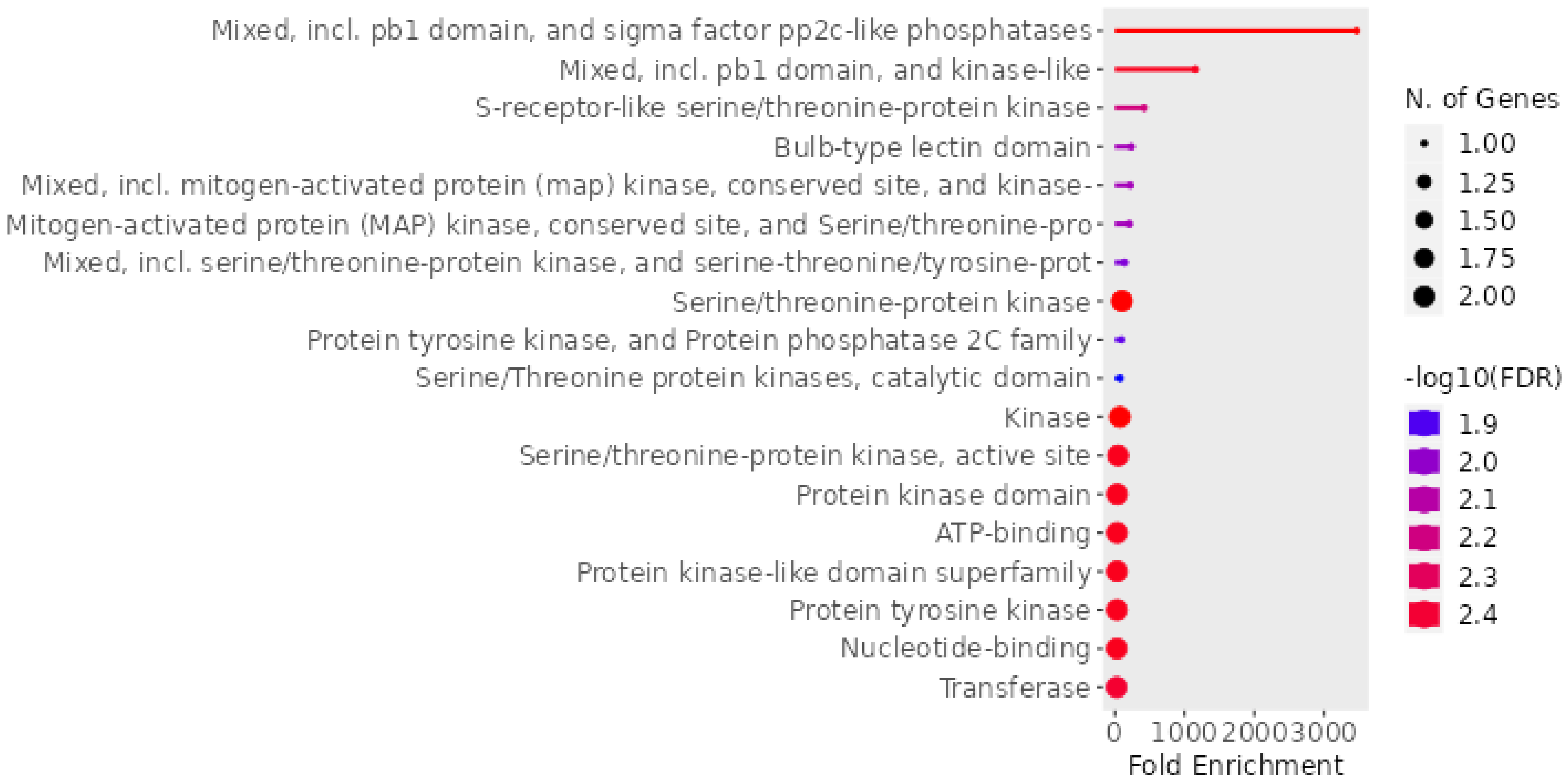

Fig. S109. Gene ontology enrichment analysis were confirmed the functional role of RLK as a stress-responsive
